# 5-Hydroxy-pyrrolone based building blocks as maleimide alternatives for protein bioconjugation and single-site multi-functionalization†

**DOI:** 10.1039/d0sc05881e

**Published:** 2021-03-04

**Authors:** Ewout De Geyter, Eirini Antonatou, Dimitris Kalaitzakis, Sabina Smolen, Abhishek Iyer, Laure Tack, Emiel Ongenae, Georgios Vassilikogiannakis, Annemieke Madder

**Affiliations:** Organic and Biomimetic Chemistry Research Group OBCR, Department of Organic and Macromolecular Chemistry, Faculty of Sciences, Ghent University Krijgslaan 281 S4 9000 Ghent Belgium annemieke.madder@ugent.be; Department of Chemistry, University of Crete Vasilika Vouton 71003 Iraklion Crete Greece vasil@uoc.gr

## Abstract

Recent dramatic expansion in potential uses of protein conjugates has fueled the development of a wide range of protein modification methods; however, the desirable single-site multi-functionalization of proteins has remained a particularly intransigent challenge. Herein, we present the application of 5-hydroxy-1,5-dihydro-2*H*-pyrrol-2-ones (5HP2Os) as advantageous alternatives to widely used maleimides for the chemo- and site-selective labeling of cysteine residues within proteins. A variety of 5HP2O building blocks have been synthesized using a one-pot photooxidation reaction starting from simple and readily accessible furans and using visible light and oxygen. These novel reagents display excellent cysteine selectivity and also yield thiol conjugates with superior stability. 5HP2O building blocks offer a unique opportunity to introduce multiple new functionalities into a protein at a single site and in a single step, thus, significantly enhancing the resultant conjugate's properties.

## Abstract 2

Recent expansion in potential uses of protein conjugates has fueled the development of a range of protein modification methods; however, the desirable single-site multi-functionalization of proteins has remained a particularly intransigent challenge.

## Graphical abstract

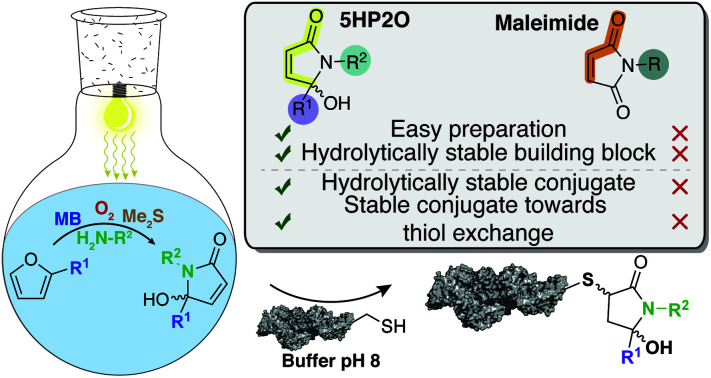

## Introduction

Chemo- and site-selective protein modifications are cornerstones of chemical biology as they offer a powerful strategy to both alter and study the properties of proteins. In recent years, a rapid increase in the demand for modified proteins for use in a range of applications (from fluorescent labeling to new conjugate-based therapeutic technologies) has led to increased attention being focused on the development of novel methods for protein modification.^1–4^ Site-selective modifications can be achieved *via* different strategies, including by incorporation of unnatural amino acids possessing distinct biorthogonal reactivities or by using enzymatic methods to modify a target tag or recognition pattern.^5^ The modifications of proteinogenic amino acid side-chains and the C- and N-termini of a protein are other widely used methods.^6^

Despite recent advances in modifying Lys,^7^ Met,^8–10^ His,^11^ Tyr^12–14^ and Trp^15,16^ residues, cysteine is by far the most attractive target for chemo- and site-selective protein conjugation. Cysteine is considered the most robustly nucleophilic amino acid of the 20 canonical amino acids since it possesses a sulfhydryl group, which, under physiological conditions, has a high propensity to form the thiolate anion (p*K*_a_ of 8.3). In addition, cysteine residues are low in natural abundance and can be introduced easily in a specific position into proteins by mutagenesis, thus allowing for site-selective modification. These innate properties mean that cysteine has the ideal profile for participation in site-specific protein manipulations targeting clinically useful protein conjugates, such as antibody–drug conjugates (ADCs).^17^

**Scheme 1:**
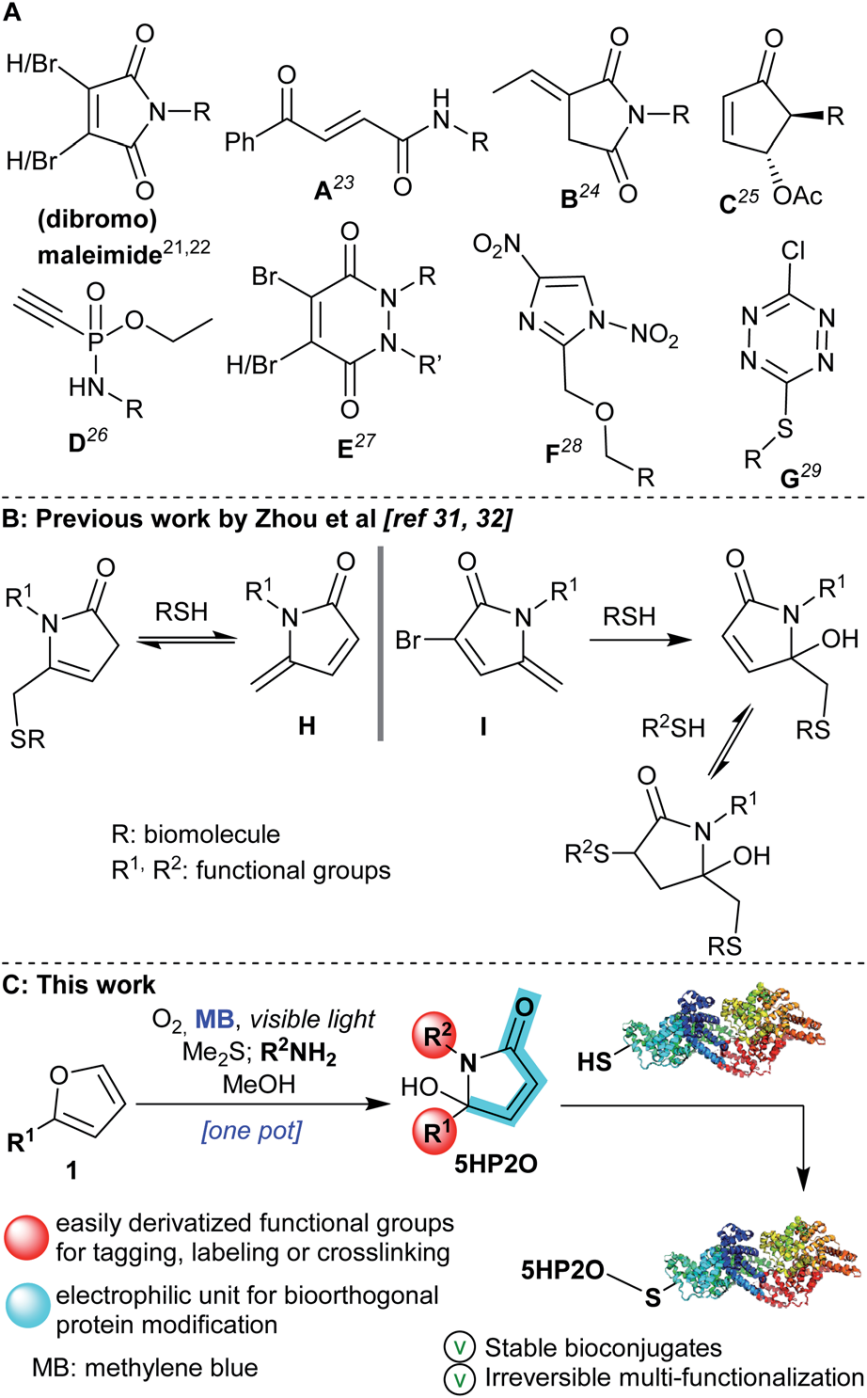
(A) Maleimide and next-generation reagents^21–29^ for the labeling of cysteine residues. (B) Previously studied thiol conjugations with pyrrolone **H** and **I** by Zhou *et al.*^31,32^ (C) Singlet oxygen-induced formation of 5HP2O as a candidate for cysteine multi-functionalization.

Within the toolbox of selective conjugation methods, maleimides are the most widely used cysteine modifiers (acting *via* a Michael addition reaction). However, maleimides are susceptible to hydrolysis, as well as having a tendency to undergo retro-Michael reactions and/or thiol exchange reactions leading to protein conjugates with poor stability.^18–20^ In response to these issues, a number of research groups have developed next-generation cysteine modifiers,^21^ with an emphasis on improving reactivity, specificity, stability and biocompatibility. Recent alternatives are included in Scheme 1A: dibromomaleimides,^22^ carbonylacrylic reagents (**A**),^23^ exocyclic olefinic succinimides (**B**),^24^ 2-cyclopentenones (**C**),^25^ phosphonamidates (**D**),^26^ (di-)bromopyridazinediones (**E**),^27^ dinitroimidazoles (**F**)^28^ and chlorotetrazines (**G**).^29^ However, not all of these compounds are readily accessible nor do they have all the requisite characteristics to act as single-site multi-functionalization mediators that form protein conjugates. Efforts have been made to address the multi-functionalization issue with a recent one-pot thiol-amine bioconjugation strategy using dibromomaleimides for dual functionalisation in two steps.^30^

Recently, two elegant studies were published by Zhou *et al.*,^31,32^ where methylene pyrrolone derivatives (**H** and **I**, Scheme 1B) were applied for cysteine modification. In the case of mono-substituted pyrrolone **H**, the reaction with thiols was characterised by its reversibility, a feature exploited in bioconjugation protocols where the removal of modifiers was desired.^31^ Insertion of a bromine atom at the 3-position of the pyrrolone backbone (**I**) proved crucial to achieving an irreversible thiol bioconjugation. It also provided the opportunity for a second thiol addition reaction, and, consequently, to access dual-functionalization. However, the second addition was reversible giving unstable double addition adducts.^32^

Over the last few years, our groups have established expertise in singlet oxygen-based synthetic chemistry^33–36^ and its application in DNA^37,38^ and peptide cross-linking.^39,40^ Driven by the facile nature of these protocols, we sought to develop a new and highly versatile maleimide alternative using the 5-hydroxy-1-(R^2^)-5-(R^1^)-1,5-dihydro-2*H*-pyrrol-2-one motif which we could gain ready access to (5HP2O, Scheme 1C). We anticipated that these easily synthesized new building blocks might offer not only excellent site selectivity but also previously unattainable irreversible multi-functionalization.

The facile preparation of 5HP2O building blocks relies on the use of simple and readily available starting materials (such as substituted furans and primary amines) and requires just one synthetic operation (Scheme 1C).^41^ This one-pot procedure provides the opportunity for the direct and controlled introduction of two functional groups of choice (R^1^ and R^2^ groups). In combination with the α,β-unsaturated moiety of the 5HP2O unit, a highly selective tag and modification procedure for cysteine residues should result. Moreover, the introduction of an alkyl group (R^1^) at the 5-position of the lactam's scaffold was considered crucial for attaining the desired enhanced stability, to hydrolysis and retro-Michael reaction, of the resultant thiol conjugates in comparison with their maleimide counterparts.

## Results and discussion

### Synthesis of 5HP2O building blocks and their reactivity towards thiols

**Scheme 2:**
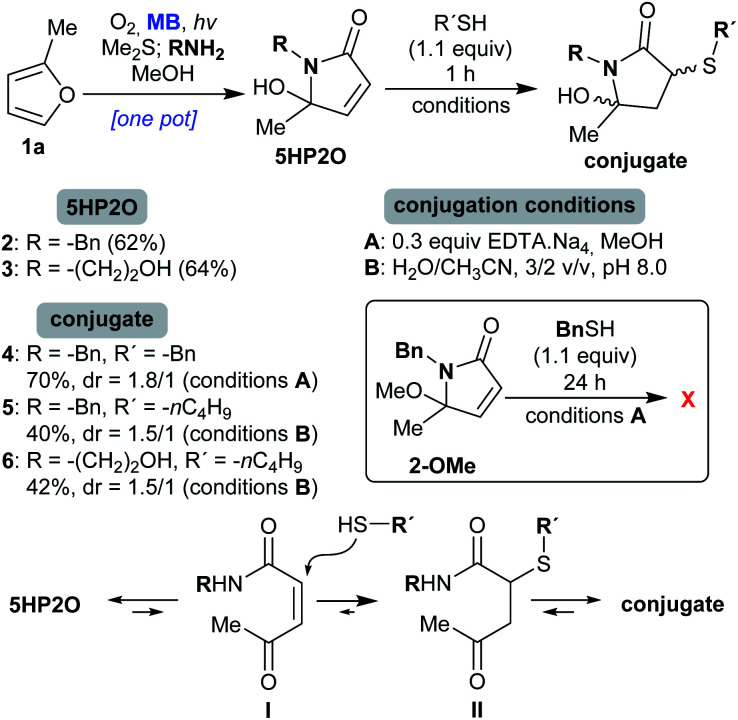
Conditions for the conjugation reaction of 5HP2O **2** and **3** with thiols and the proposed mechanistic explanation for the observed regioselectivity.

We started our investigation with the γ-lactams **2** and **3** which were easily synthesized from furan **1a** (Scheme 2).^41^ Specifically, 2-methylfuran (**1a**) was photooxidised in the presence of molecular oxygen and catalytic amounts of methylene blue (MB), followed by reduction with dimethylsulfide (Me_2_S). After the addition of the corresponding amine (benzylamine or ethanolamine), the desired 5HP2Os **2** and **3** were obtained in 62 and 64% isolated yields, respectively (Scheme 2). In order to study 5HP2O’s reactivity toward thiol conjugation, compound **2** was subjected to 1.1 equiv. of benzyl mercaptan in methanol in the presence of 0.3 equiv. of EDTA.Na_4_ (conditions A, Scheme 2). After 1 hour at room temperature, lactam **2** was fully converted to the conjugated product **4**. The same results were obtained when either lactam **2** or **3** was subjected to 1-butanethiol in a mixture of water/acetonitrile (3/2) and at pH 8.0 (conditions B). Interestingly, in every case the conjugation proceeded exclusively at the 3 position of the lactam's backbone (products **4** and **5** were fully characterized by NMR experiments after separation of the diastereomers by flash-chromatography, see the ESI†). The regioselectivity of the reaction can be explained by the existence of an equilibrium between the 5HP2O and its open keto-form I, wherein the Michael addition proceeds at the position directed by the keto group (I → II, Scheme 2). Strong evidence for the proposed mechanism comes from the fact that the reaction of the methoxy-analogue of compound **2** (2-OMe, Scheme 2) with benzyl mercaptan, under conditions A, did not proceed even after 24 h.

To synthesize a variety of 5HP2Os, different furan substrates (**1a–1e**, variability of the R^1^ group) were combined with various amines (variability of the R^2^ group) to give a series of functionalized derivatives (**7–20**, Table 1). Many of the R^1^ and R^2^ groups were carefully selected to allow for further derivatization: for instance, the use of propargyl amine (products **7**, **12** and **13**) results in a handle that can be subsequently modified *via* the alkyne–azide click reaction.^42^ The direct incorporation of a pyrene or a dansylcadaverine group results in products (**8** and **11**, respectively) with fluorescent properties. Additionally, compound **10** represents an alternative for the widely known commercially available SMCC linker. Ethyl-3-(furan-2-yl)propionate (**1c**) was combined with different amines to give bi-functionalized moieties consisting of an ester functionality at the R^1^ position and an alkyne (compound **13**), PEG (compound **15**) or a long chain unsaturated alkyl functionality (compound **16**) at the R^2^ position. To further demonstrate the versatility of the 5HP2O synthesis, multi-functionalized building blocks were synthesized (**17–20**). Notably, building block **19** bears a long alkyl chain and the drug doxorubicin, while **20** contains a dansyl fluorophore and a short PEG moiety. Representative 5HP2O compounds were then tested in a simple thiol addition reaction with β-mercapto-ethanol under conditions A or B; in every case, the expected conjugates were obtained as a mixture of diastereomers (**21–26**, Table 2).

**Table 1:** Singlet oxygen induced preparation of various functionalized 5HP2Os

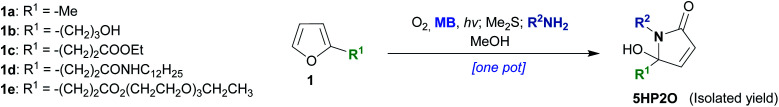
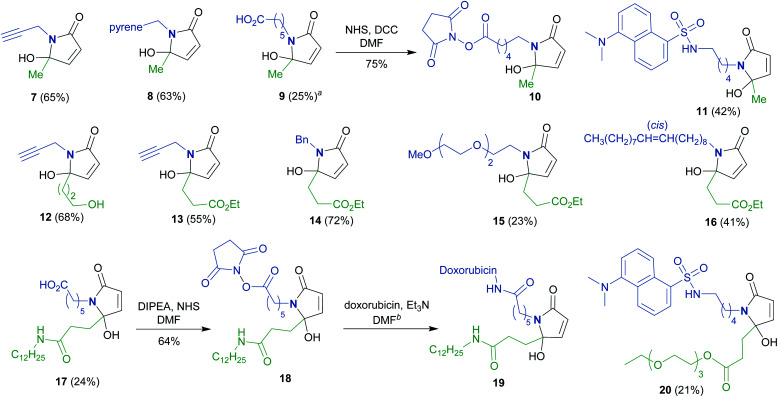

a Isolated as the triethylammonium salt.

b The reaction was monitored by RP-HPLC (conversion > 95% after 24 h).

**Table 2:** Conjugation of various 5HP2O building blocks towards β-mercapto-ethanol

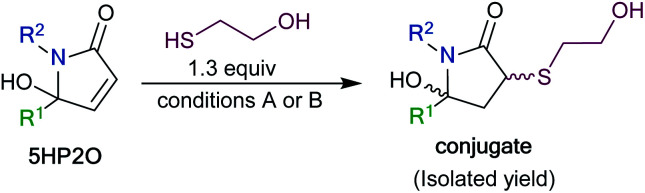
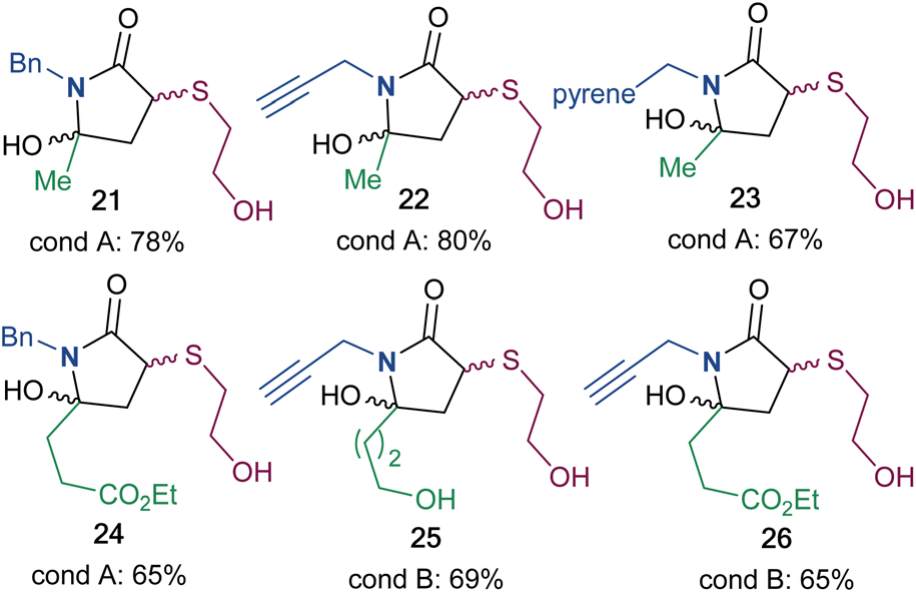

### Building block and conjugate stability

The major drawback when using maleimides is their limited stability towards hydrolysis. To assess the hydrolytic stability of 5HP2Os, pyrrolone **3** was selected as the model compound and studied in different buffer solutions at pH 6, 7 and 8. After 24 hours of incubation at 37 °C in the appropriate buffer, no hydrolysed products were observed (see the ESI†). Unlike maleimides (we also tested the hydrolytic stability of *N*-benzyl maleimide – see the ESI†), 5HP2Os proved inert towards hydrolysis, giving them a considerable advantage with respect to shelf-life. We also investigated the hydrolytic stability of the resulting thiol conjugates. Specifically, a 2 mM solution of a selected conjugate (**21**) was incubated in different buffers at pH 7, 8 and 9 (37 °C, 24 h, see the ESI†). In any case **21** proved stable towards hydrolysis.

Another drawback of maleimide-based protein conjugation is the fact that maleimide–thiol adducts can undergo thiol exchange reactions at physiological pH and temperature.^43^ Therefore, the stability of **21** towards thiol exchange was tested using 10 equiv. of glutathione at 37 °C (glutathione is found in high concentrations in cells). We found that trace amounts of the exchanged product were observed only after 4 days (see the ESI†). This observation contrasts with the behavior of the thiol conjugates coming from pyrrolones of type **I** (Scheme 1B),^32^ where thiol exchange was observed, most probably due to the presence of the sulfur group at the exocyclic position. As a consequence, 5HP2O building blocks fulfill all the criteria in terms of hydrolytic stability and Michael-addition irreversibility to be able to answer the previously unmet call for a selective method for dual-functionalization of cysteine residues.

### Conjugations to peptides and proteins

**Fig. 1:**
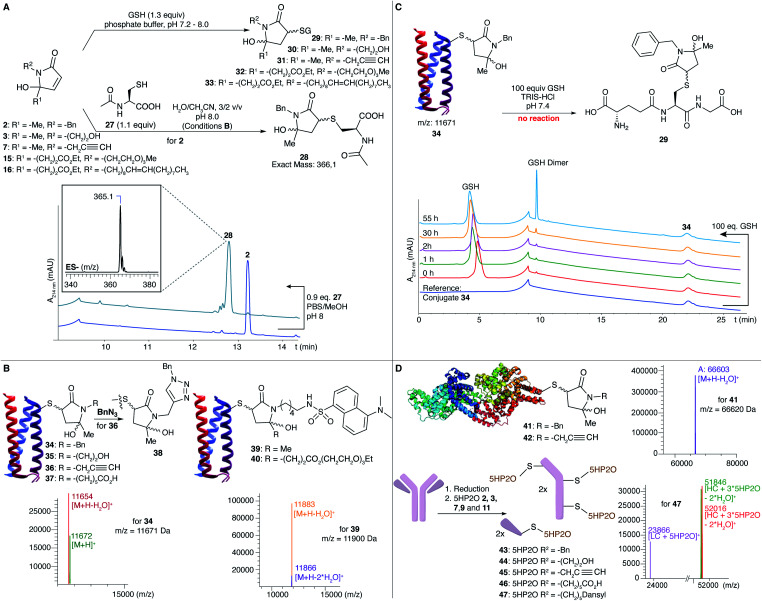
(A) *N*-Acetyl-l-cysteine and glutathione (GSH) conjugation to 5HP2O **2**, **3**, **7**, **15** and **16**. Conversion of **2** to **28** was observed by RP-HPLC and the product was analysed by LC-MS ([M − H]^−^ = 365.1). (B) Conjugation of different 5HP2Os (using 10 equiv.) is achieved on alphabody (**34–37**, **39** and **40**) at a pH of 8. (C) Conjugate **34** was incubated with 100 equiv. of GSH and the reaction was monitored by RP-HPLC. No thiol-exchanged product **29** was found. (D) Protein conjugates were efficiently prepared for BSA (**41** and **42**) and for the antibody trastuzumab (**43–47**) using 100 equiv. of 5HP2O at a pH of 8.

Following the efficient conjugation of 5HP2Os with simple thiols, we embarked on extending the reactivity testing using *N*-acetyl-l-cysteine (NAC) (**27**) and glutathione (GSH) as model nucleophiles. Full conversion of **2** into the desired conjugate **28** was observed within 2.5 h under conditions B (Fig. 1A, see also the ESI†). Additionally, a direct comparison with benzyl-maleimide was made using, respectively, 5 and 20 equiv. of building block and following the kinetics of conjugation over time. Upon addition of the maleimide, immediate and full conversion was seen at a pH of 8 in accordance with the known fast reaction kinetics for maleimides. However, also with 20 equiv. of 5HP2O full conversion was reached after only 30 min. The expected GSH conjugates (**29–33**) with different 5HP2O building blocks (**2**, **3**, **7**, **15** and **16**) were also prepared in phosphate buffered solution (Fig. 1A). The superior hydrolytic stability of our 5HP2O building blocks was illustrated *via* a direct comparison between NAC-conjugates with, respectively, benzyl-maleimide and our benzyl-5HP2O (**2**). NAC-conjugates were incubated for 24 hours in buffered aqueous media at pH of 7, 8 and 9 ^44^ and at a concentration of 0.4 mM and analysed *via* HPLC. While for our 5HP2O conjugates no hydrolysis could be observed after 24 hours in all studied buffered media, maleimide conjugates hydrolysed up to 50% after 24 hours at a pH of 7 and more than 99% after 24 hours at a pH of 9 (see the ESI†). Following the hydrolytic stability studies, GSH exchange experiments were performed. NAC-conjugates were incubated in buffered media at a pH of 7.5 with 10 mM GSH at a concentration of 0.1 mM. Degradation of the NAC-conjugates was followed *via* HPLC (see the ESI†). Even with 100 equivalents of GSH, 70% of intact NAC–5HP2O conjugate is left after 5 days, while for maleimides only 20% of intact conjugate is left. The degradation of the NAC–maleimide conjugate is the result of the hydrolysis of the NAC-conjugate combined with GSH exchange as well as the hydrolysis of the exchanged GSH-conjugate.^45^ Detailed investigations on the influence of the nature of the *N*-substituent as well as the role of the exact structure and p*K*_a_ of the thiol are currently ongoing and form the subject of a separate detailed study, which will be reported in due course.

To evaluate the applicability of 5HP2Os as genuine protein modifiers, bioconjugation experiments were performed using a model protein, MB23-Cys alphabody obtained from Complix N.V. (Fig. 1B). Alphabody proteins are of therapeutic interest because of their capacity to target protein–protein interfaces.^46^ The alphabody possesses one surface-exposed free cysteine residue and is an ideal model protein. The reaction was conducted using 10 equiv. of different 5HP2O building blocks (**2**, **3**, **7** and **9**) under Tris buffer conditions (10 mM, pH 8) at 25 °C. Efficient formation of the corresponding conjugates **34–37** was observed after 6–12 h. Additionally, protein conjugation conditions were screened in the pH range between 6 and 9 using 10 equiv. of **2**. Limited conjugation was observed at a pH of 6, while full conjugation was seen in the pH range 7–9 (see the ESI†).

Notably, we could efficiently modify the alphabody with a fluorescent label using dansyl 5HP2O **11** (conjugate **39**, Fig. 1B). For one-site bi-functionalization, an alphabody was treated with bi-functional 5HP2O **20** to yield an alphabody conjugate consisting of a dansyl fluorophore and a PEG moiety (**40**). Additionally, as a proof-of-concept, an initial experiment was performed to click benzyl-azide onto an alkyne-functionalised alphabody according to the well-known CuAAC reaction (product **38**, Fig. 1B), illustrating the potential for versatile tandem 5HP2O–CuAAC modification protocols. Conjugate **34** was found to be hydrolytically stable in 10 mM Tris buffer at pH 7, 8 or 9 for up to 6 days. In contrast, when an alphabody was treated with 6-maleimidohexanoic acid under the same conditions, ring-opened hydrolysis products were observed (see the ESI†). Finally, the stability of the new alphabody conjugate towards thiol exchange was tested using 100 equivalents of glutathione. The RP-HPLC analysis showed no thiol exchange, with no peak for product **29** observed even after several days at 35 °C (Fig. 1C and see the ESI†).

To further illustrate the potential of 5HP2O, modification reactions were successfully performed on bovine serum albumin (BSA). When the protein was treated with 100 equiv. of building blocks **2** and **7**, the mono-conjugates **41** and **42** were observed (Fig. 1D). Finally, exemplary application of the current conjugation protocol in a therapeutic context was obtained by conjugation of **2**, **3**, **7**, **9** and **11** to the Her2-addressing IgG monoclonal antibody, trastuzumab. Trastuzumab (Fig. 1D) was first reduced using 20 equiv. of TCEP followed by the addition of 12.5 equiv. of 5HP2O per free cysteine. Labeling was confirmed by LC-MS analysis (conjugates **43–47**), which showed a shift in the signal with a mass difference corresponding to one or more 5HP2O moieties in accordance with the number of solvent-exposed cysteine residues present in trastuzumab (Fig. 1D), illustrating the high degree of chemo selectivity.

## Conclusions

To conclude, we have presented an efficient one-pot synthesis of a novel class of 5HP2O protein modifying agents. Functional groups at two different positions of these building blocks can be varied readily and at will (opening up further multi-functionalization possibilities, such as click reactions). The decorated 5HP2Os can be efficiently conjugated to thiols (cysteine modifiers). We have also established that the new 5HP2O modifiers have characteristics which offer significant performance enhancement when compared to the alternative maleimide-based bioconjugation methods. We have shown that the resulting conjugates are hydrolytically stable in buffered media and that the protein conjugate is stable towards thiol-exchange over prolonged periods of time which represent considerable advantages when compared to maleimide derivatives or the thiol conjugates of the 3-bromo-5-methylene pyrrolones^32^ which degrade through exchange. Furthermore, protein labeling with 5HP2Os resulted in conjugates with high stability. Therefore, we propose our 5HP2O building blocks as highly advantageous maleimide alternatives for protein modification and single-step multi-functionalization.

## Author contributions

E. D. G. was responsible for conceptualization, data curation, formal analysis, lead investigation (ESI chapters 1–8†), methodology, project administration, visualization and writing of the original draft. E. A. was responsible for data curation, lead investigation (ESI chapters 1, 4 and 6†), methodology and visualization. D. K. was responsible for conceptualization, data curation, lead investigation (ESI chapters 1 and 4†), methodology, project administration, visualization, supportive supervision and writing, reviewing and editing of the manuscript. S. S. was responsible for conceptualization, data curation, investigation (ESI chapters 1, 2 and 4–6†), methodology and visualization. A. I. was responsible for writing the original draft. L. T and E. O. were responsible for supporting investigation in the synthesis of compounds **18**, **19**, **32** and **33**. G. V. and A. M. were responsible for conceptualization and supervision, funding acquisition, project administration, resources and reviewing and editing of the manuscript.

## Conflicts of interest

The technology described in the manuscript is part of a pending patent application (EP 19160048.5) by A. M., G. V., E. A., E. D. G., D. K. and S. S.

## Supplementary Material

SC-012-D0SC05881E-s001
