# Supplementary material for: 5-Hydroxy-pyrrolone based building blocks as maleimide alternatives for protein bioconjugation and single-site multi-functionalization
Source: Chem Sci. 2021 Mar 4;12(14):5246–52. doi: 10.1039/d0sc05881e (PMC8179572; doi:10.1039/d0sc05881e)

Electronic Supplementary Material (ESI) for Chemical Science.
This journal is © The Royal Society of Chemistry 2021

5-Hydroxy-Pyrrolone Based Building Blocks as Maleimide Alternatives for Protein Bioconjugation and Single-Site Multi-Functionalization

Ewout De Geyter+,[a] Eirini Antonatou+,[a] Dimitris Kalaitzakis+,[b] Sabina Smolen, [a] Abhishek Iyer, [a] Laure Tack, [a] Emiel Ongenae, [a] Georgios Vassilikogiannakis*,[b] Annemieke Madder*[a]

[a] E. De Geyter, Dr. E. Antonatou, Dr. A. Iyer, Dr. S. Smolen, Prof. Dr. A. Madder
Organic and Biomimetic Chemistry Research group OBCR, Department of Organic and Macromolecular Chemistry, Faculty of Sciences, Ghent University, Krijgslaan 281 S4, 9000 Ghent, Belgium
Email: Annemieke.Madder@UGent.be

[b] Dr. D. Kalaitzakis, Prof. Dr. G. Vassilikogiannakis
Department of Chemistry, University of Crete
Vasilika Vouton, 71003, Iraklion, Crete, Greece
E-mail: vasil@uoc.gr
Homepage: www.chemistry.uoc.gr/vassilikogiannakis

[+] These authors contributed equally to this work.

Table of Contents

1. PREPARATION OF 5-HYDROXY-1,5-DIHYDRO-2H-PYRROL- 2(5H)-ONE BUILDING BLOCKS 4

2. HYDROLYTIC STABILITY STUDIES OF THE 5-HYDROXY-1H-PYRROL- 2(5H)-ONE BUILDING BLOCK (3) 12

3. HYDROLYTIC STABILITY OF N-BENZYLMALEIMIDE 16

4. REACTIVITY OF 5HP2O BUILDING BLOCKS TOWARDS THIOLS 17

4.1. Conjugation to thiols in MeOH 17

4.1.1. General procedure for the preparation of 5-hydroxy-3-((2- hydroxyethyl)thio)-pyrrolidin-2-ones 17

4.2. Conjugation to thiols in H_2O/MeCN 20

5. STABILITY OF 5-HYDROXY-3-((2- HYDROXYETHYL)THIO)-PYRROLIDIN-2-ONE (21) 24

5.1. Hydrolytic stability studies 24

5.2. Glutathione competition experiment 26

6. REACTIVITY OF 5HP2O BUILDING BLOCKS TOWARDS PEPTIDE-LIKE COMPOUNDS 28

6.1. N-Acetyl-L-cysteine (NAC) conjugation 28

6.1.1. Kinetic analysis 31

6.2. Stability of the NAC conjugates 33

6.2.1. Hydrolytic stability 33

6.2.2. Glutathione competition experiment 36

6.3. General procedure for the conjugation of α,β-unsaturated γ-lactam with GSH 39

6.3.1. Conjugation of lactam 2 with GSH 40

6.3.2. Conjugation of lactam 3 with GSH 42

6.3.3. Conjugation of lactam 7 with GSH 44

6.3.4. Conjugation of lactam 15 with GSH 45

6.3.5. Conjugation of lactam 16 with GSH 46

7. REACTIVITY OF 5HP2O BUILDING BLOCKS TOWARDS PROTEINS 48

7.1. MB23 Alphabody Protein 48

7.1.1. MB23 pre-reduction 48

7.1.2. Verification of free thiol functionality by reaction of MB23 with Ellman’s reagent 49

7.1.3. Alphabody conjugation to 5HP2O building blocks 49

7.1.4. Alphabody conjugation to 6-maleimidohexanoic acid 63

7.1.5. Hydrolytic stability of the alphabody conjugate 64

7.1.6. CuAAC reaction on Alphabody conjugate 68

7.1.7. Glutathione competition experiment 69

7.2. Bovine Serum Albumin (BSA) conjugation 72

7.2.1. Verification of free thiol functionality by reaction of BSA with Ellman’s reagent 73

7.2.2. BSA conjugation to 5HP2O building blocks 74

8. REACTIVITY OF 5HP2O BUILDING BLOCKS TOWARDS TRASTUZUMAB ANTIBODY 77

8.1. Trastuzumab reduction 77

8.2. Trastuzumab conjugation to 5HP2O building block 2 78

8.3. Trastuzumab conjugation to 5HP2O building block 3 79

8.4. Trastuzumab conjugation to 5HP2O building block 7 80

8.5. Trastuzumab conjugation to 5HP2O building block 9 81

8.6. Trastuzumab conjugation to 5HP2O building block 11 83

9. COPIES OF NMR SPECTRA 84

1. Preparation of 5-hydroxy-1,5-dihydro-2H-pyrrol- 2(5H)-one building blocks

Synthesis of *N*-dodecyl-3-(furan-2-yl)propenamide (1d)

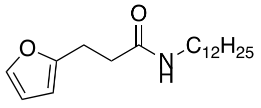

Substituted furan 3-(2-furyl)propionic acid (147.7 mg, 1 mmol) and HBTU (569 mg, 1.5 mmol) were dissolved in dry DMF (10 mL) under argon atmosphere. Then a solution of diisopropylethylamine (696 μL, 4 mmol) and dodecylamine (378.8 mg, 2 mmol) in dry DMF (1 mL) was added to the solution. The solution was stirred overnight at room temperature. The reaction was monitored by tlc analysis. After completion of the reaction, brine was added (50 ml) and the mixture was extracted with EtOAc (2x 50 mL). The combined organic phases were washed with brine (2x 50 mL), dried over $MgSO_4$, concentrated under reduced pressure and purified via reverse-phase prep-HPLC.

Yield 70% (224 mg). ^{1}H NMR (400 MHz, $CDCl_3$): δ = 7.29 (dd, J_1 = 1.8 Hz, J_2 = 0.8 Hz, 1H), 6.27 (dd, J_1 = 3.1 Hz, J_2 = 1.9 Hz, 1H), 6.02 (dd, J_1 = 3.1 Hz, J_2 = 0.8 Hz, 1H), 3.21 (m, 2H), 2.98 (t, J = 7.5 Hz, 2H), 2.49 (t, J = 7.5 Hz, 2H), 1.44 (m, 2H), 1.25 (s, 18H), 0.88 (t, J = 6.9 Hz, 3H) ppm; ^{13}C NMR (101 MHz, $CDCl_3$): δ =171.7, 154.5, 141.3, 110.4, 105.7, 39.7, 35.3, 32.1, 29.8 (2C), 29.7 (3C), 29.5, 29.4, 27.0, 24.3, 22.8, 14.3 ppm; HRMS (TOF ESI): calculated for $C_{19}H_{33}NO_2$: 308.2511[M+H]$^+$; found: 308.2574.

Synthesis of 2-(2-(2-ethoxyethoxy)ethoxy)ethyl 3-(furan-2-yl)propanoate (1e)

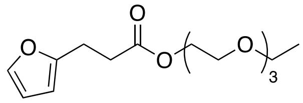

3-(2-furyl)propionic acid (110 mg, 0.78 mmol) was dissolved in tri(ethylene glycol) monoethyl ether (14 mL) with 1% H_2SO_4. The reaction was stirred for 24 hours. After ompletion of the reaction the product was purified by reverse phase prep-HPLC.

Yield 63% (147.6 mg). ^{1}H NMR (400 MHz, CD_3OD): δ = 7.35 (d, J = 1.9 Hz, 1H), 6.28 (dd, J_1 = 3.0 Hz, J_2 = 1.9 Hz, 1H), 6.06 (d, J = 3.0 Hz, 1H), 4.22 (m, 2H), 3.68 (m, 2H), 3.64-3.60 (m, 6H), 3.59-3.49 (m, 4H), 2.93 (m, 2H), 2.67 (t, J = 7.5 Hz, 2H), 1.18 (t, J = 7.0 Hz, 3H) ppm; ^{13}C NMR (101 MHz, CD_3OD): δ =174.2, 155.6, 142.6, 111.3, 106.4, 71.7 (3C), 71.0, 70.2, 67.7, 65.0, 33.7, 24.5, 15.6 ppm; HRMS (TOF ESI): Calculated for $C_{15}H_{24}O_6$: 301.1573 [M+H]$^+$; mass found: 301.1639 [M+H]$^+$.

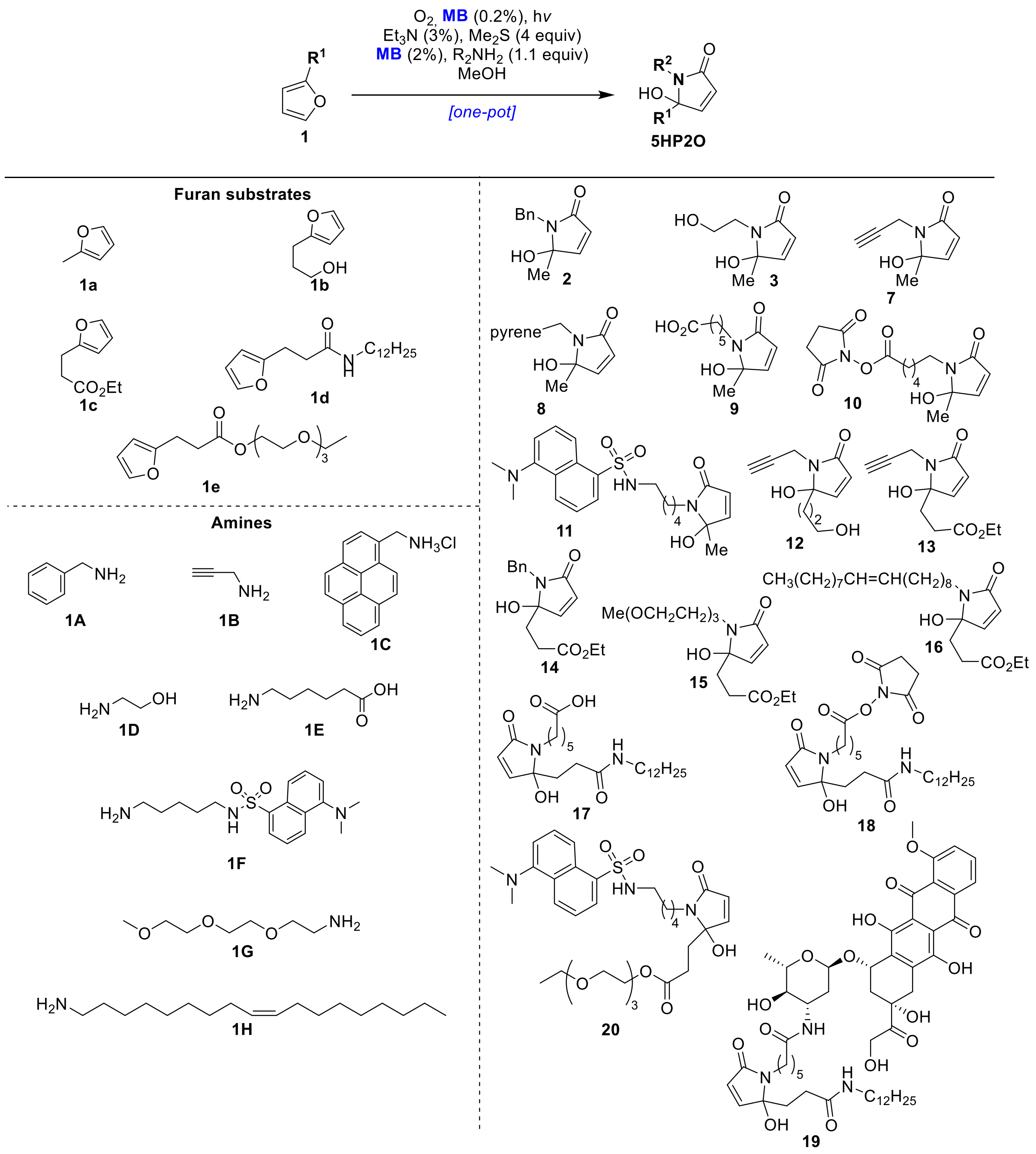

Scheme S 1: General procedure for the synthesis of 5HP2O building blocks.

General procedure for the synthesis of 5HP2O building blocks

2-Substituted furan of type **1** (1.0 mmol, 90 µL for **1a**, 126 mg for **1b**,[1] 168 mg for **1c**) were dissolved in methanol (12 mL) containing catalytic amounts of methylene blue (0.2 mol%, 0.64 mg, 0.002 mmol). The solutions were cooled with an ice bath. Oxygen was gently bubbled through the solutions while they were irradiated with a xenon Variac Eimac Cermax 300 W lamp. The reactions were monitored by TLC. After completion of the reactions (7 min), the solution was warmed to room temperature and Et_3N (4.2 µL, 0.03 mmol) followed by Me_2S (291 µL, 4.0 mmol) was added. After completion of the reduction (45 min), an

[1] D. Kalaitzakis, E. Antonatou, G. Vassilikogiannakis, *Chem. Commun.*, 2014, ***50***, 400-402.

additional amount of methylene blue was added (2 mol%, 6.4 mg, 0.02 mmol) followed by the corresponding amine [1.1 mmol, 120 μL of **1A**, 70.5 μL of **1B**, 66.5 μL of **1D**, 144 mg of **1E** (which was first neutralized in MeOH with 2 equiv of Et_3N, 278 μL, 2.0 mmol), 368.5 mg of **1F**, 180 mg of **1G**, 0.362 mL **1H** or a 2 mL of a methanolic solution containing 294.5 mg of **1C** and Et_3N, 230 μL, 1.65 mmol], was added and the solutions were stirred at rt. After completion of the reactions, as indicated by tlc analysis (3 h), the solutions were concentrated *in vacuo* and the final 5-hydroxy-1H-pyrrol-2(5H)-ones were purified by flash column chromatography (silica gel, petroleum ether:EtOAc, 5:1 → 2:1→ 1:1 for **2**, **8**, **13** and **14** or 2:1 → 0:1 for **3**, **7** and **12**) or by preparative reverse-phase HPLC for **9**, **11, 15** and **16**.

1-benzyl-5-hydroxy-5-methyl-1,5-dihydro-2H-pyrrol-2-one (2)

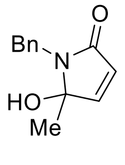

Yield 62% (126 mg). ^{1}H NMR (500 MHz, $CDCl_3$): δ = 7.28 (m, 4H), 7.23 (m, 1H), 6.90 (d, *J* = 6.0 Hz, 1H), 6.02 (d, *J* = 6.0 Hz, 1H), 4.68 (d, *J* = 15.6 Hz, 1H), 4.39 (d, *J* = 15.6 Hz, 1H), 3.65 (brs, 1H), 1.31 (s, 3H) ppm; ^{13}C NMR (125 MHz, $CDCl_3$): δ = 169.6, 150.8, 138.2, 128.5 (2C), 127.8 (2C), 127.2, 125.6, 90.3, 41.5, 23.2 ppm; HRMS (TOF ESI): calculated for $C_{12}H_{13}NO_2$: 204.1019 $[M+H]^+$; found: 204.1026.

5-hydroxy-1-(2-hydroxyethyl)-5-methyl-1,5-dihydro-2H-pyrrol-2-one (3)

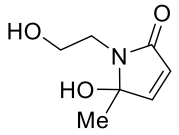

Yield 64% (100.5 mg). ^{1}H NMR (500 MHz, $CDCl_3$): δ = 6.95 (d, *J* = 5.9 Hz, 1H), 6.01 (d, *J* = 5.9 Hz, 1H), 3.81-3.71 (m, 3H), 3.25 (m, 1H), 1.52 (s, 3H); ^{13}C NMR (125 MHz, $CDCl_3$): δ = 170.3, 151.1, 125.3, 89.7, 61.5, 41.1, 22.6. HRMS (TOF ESI): calculated for $C_7H_{11}NO_3$: 158.0739 $[M+H]^+$; mass found: 158.0810$[M+H]^+$.

5-hydroxy-5-methyl-1-(prop-2-yn-1-yl)-1,5-dihydro-2H-pyrrol-2-one (7)

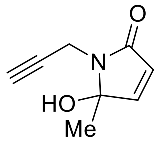

Yield 65% (98 mg). ^{1}H NMR (500 MHz, $CDCl_3$): δ = 6.96 (d, *J* = 6.0 Hz, 1H), 6.00 (d, *J* = 6.0 Hz, 1H), 4.15 (dd, J_1 = 17.8 Hz, J_2 = 2.5 Hz, 1H), 4.05 (dd, J_1 = 17.8 Hz, J_2 = 2.5 Hz, 1H), 3.55 (brs, 1H), 2.16 (t, *J* = 2.5 Hz, 1H), 1.62 (s, 3H) ppm; ^{13}C NMR (125 MHz, $CDCl_3$): δ = 168.6, 151.1, 125.5, 90.0, 79.2, 70.8, 26.6, 22.6 ppm; HRMS (TOF ESI): calculated for $C_8H_9NO_2$: 152.0706 $[M+H]^+$; found: 152.0704.

5-hydroxy-5-methyl-1-(pyren-1-ylmethyl)-1,5-dihydro-2H-pyrrol-2-one (8)

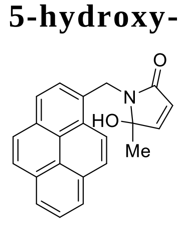

Yield 63% (206 mg). ^{1}H NMR (500 MHz, $CHCl_3$): 8.41 (d, *J* = 9.2 Hz, 1H), 8.15 (t, *J* = 7.2 Hz, 2H), 8.09 (d, *J* = 7.7 Hz, 1H), 8.08 (d, *J* = 9.2 Hz, 1H), 8.03-7.96 (m, 4H), 6.83 (d, *J* = 6.0 Hz, 1H), 6.06 (d, *J* = 6.0 Hz, 1H), 5.31 (d, *J* = 15.6 Hz, 1H), 5.24 (d, *J* = 15.6 Hz, 1H), 1.17 (s, 3H) ppm; ^{13}C NMR (125 MHz, $CDCl_3$): δ = 169.5, 151.0, 131.2, 130.9, 130.8, 130.6, 128.6, 127.9, 127.3, 127.2, 127.0, 125.9, 125.5, 125.2, 125.1, 124.8, 124.6, 122.9, 90.6, 39.7, 23.0 ppm.

6-(2-hydroxy-2-methyl-5-oxo-2,5-dihydro-1H-pyrrol-1-yl)hexanoic acid (9)

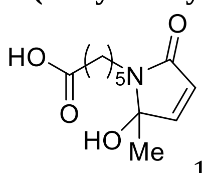

Compound **9** was isolated as a thiethylammonium salt. Yield 25% (82 mg). ^{1}H NMR (400 MHz, DMSO-d_6): δ = 7.02 (d, *J* = 6.0 Hz, 1H), 5.98 (d, *J* = 6.0 Hz, 1H), 3.20 (m, 1H), 3.11 (m, 1H), 2.17 (t, *J* = 7.4 Hz, 2H), 1.50 (m, 4H), 1.39 (s, 3H), 1.28 (m, 2H); ^{13}C NMR (101 MHz, DMSO-d_6): δ = 174.7, 168.3, 151.5, 124.8, 89.0, 37.4, 34.0, 28.4, 26.2, 24.4, 23.5; HRMS (TOF ESI): calculated for $C_{11}H_{17}NO_4$: 228.1158 $[M+H]^+$; mass found: 228.1230 $[M+H]^+$.

5-(dimethylamino)-N-(5-(2-hydroxy-2-methyl-5-oxo-2,5-dihydro-1H-pyrrol-1-yl)pentyl)naphthalene-1-sulfonamide (11)

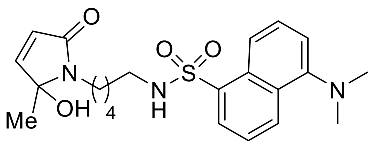

Yield 42% (181.1 mg). ^{1}H NMR (400 MHz, CD_3OD): δ = 8.56 (dt, J_1 = 8.5 Hz, J_2 = 1.1 Hz, 1H), 8.36 (dt, J_1 = 8.5 Hz, J_2 = 0.9 Hz, 1H), 8.19 (dd, J_1 = 7.3 Hz, J_2 = 1.3 Hz, 1H), 7.58 (m, 2H), 7.28 (dd, J_1 = 7.6 Hz, J_2 = 0.7 Hz, 1H), 6.99 (d, J = 6.0 Hz, 1H), 5.98 (d, J = 6.0 Hz, 1H), 3.18 (m, 1H), 3.04 (m, 1H), 2.88 (s, 6H), 2.85 (t, J = 6.9 Hz, 2H), 1.42 (s, 3H), 1.36 (m, 4H), 1.17 (m, 2H); ^{13}C NMR (101 MHz, CD_3OD): δ = 171.4, 153.2, 153.0, 137.2, 131.2, 131.1, 131.0, 130.2, 129.1, 125.9, 124.3, 120.6, 116.4, 91.4, 45.8 (2C), 43.8, 39.1, 30.2, 29.4, 25.1, 23.4; HRMS (TOF ESI): calculated for $C_{22}H_{29}N_3O_4S$: 432.1879 $[M+H]^+$; mass found: 432.1950 $[M+H]^+$.

5-hydroxy-5-(3-hydroxypropyl)-1-(prop-2-yn-1-yl)-1,5-dihydro-2H-pyrrol-2-one (12)

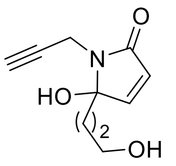

Yield 68% (132.6 mg). ^{1}H NMR (500 MHz, CD_3OD): δ = 7.06 (d, J = 6.0 Hz, 1H), 6.11 (d, J = 6.0 Hz, 1H), 4.21 (dd, J_1 = 17.9 Hz, J_2 = 2.5 Hz, 1H), 3.97 (dd, J_1 = 17.9 Hz, J_2 = 2.5 Hz, 1H), 3.53 (m, 2H), 2.55 (t, J = 2.5 Hz, 1H), 2.03 (ddd, J_1 = 14.0 Hz, J_2 = 11.1 Hz, J_3 = 5.8 Hz, 1H), 1.96 (ddd, J_1 = 14.0 Hz, J_2 = 10.6 Hz, J_3 = 5.7 Hz, 1H), 1.45 (m, 2H) ppm; ^{13}C NMR (125 MHz, CD_3OD): δ = 169.8, 151.0, 125.5, 92.3, 78.7, 70.5, 61.2, 31.9, 26.6, 25.9 ppm.

Ethyl 3-(2-hydroxy-5-oxo-1-(prop-2-yn-1-yl)-2,5-dihydro-1H-pyrrol-2-yl)propanoate (13)

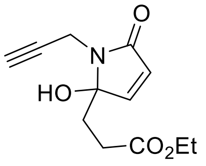

Yield 55% (130.3 mg). ^{1}H NMR (300 MHz, $CDCl_3$): δ = 6.91 (d, J = 6.0 Hz, 1H), 6.07 (d, J = 6.0 Hz, 1H), 4.19 (dd, J_1 = 17.9 Hz, J_2 = 2.5 Hz, 1H), 4.10 (d, J = 7.1 Hz, 1H), 3.99 (dd, J_1 = 17.9 Hz, J_2 = 2.5 Hz, 1H), 2.36 (m, 2H), 2.25 (m, 2H), 2.16 (t, J = 2.5 Hz, 1H), 1.22 (t, J = 7.1 Hz, 3H) ppm; ^{13}C NMR (75 MHz, $CDCl_3$): δ = 172.8, 168.8, 149.1, 126.9, 91.8, 78.6, 71.0, 60.8, 30.1, 28.9, 26.6, 14.1 ppm.

Ethyl 3-(1-benzyl-2-hydroxy-5-oxo-2,5-dihydro-1H-pyrrol-2-yl)propanoate (14)

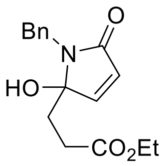

Yield 72% (208 mg). ^{1}H NMR (500 MHz, $CDCl_3$): δ = 7.35 (d, J = 7.2 Hz, 2H), 7.27 (t, J = 7.2 Hz, 2H), 7.22 (t, J = 7.2 Hz, 1H), 6.84 (d, J = 6.0 Hz, 1H), 6.06 (d, J = 6.0 Hz, 1H), 4.63 (d, J = 15.3 Hz, 1H), 4.34 (d, J = 15.3 Hz, 1H), 4.00 (q, J = 7.1 Hz, 2H), 2.16 (ddd, J_1 = 13.8 Hz, J_2 = 8.3 Hz, J_3 = 6.8 Hz, 1H), 1.99 (m, 1H), 1.94-1.81 (m, 2H), 1.17 (t, J = 7.2 Hz, 3H) ppm; ^{13}C NMR (125 MHz, $CDCl_3$): δ = 172.6, 169.8, 148.7, 137.8, 128.5 (2C), 128.3 (2C), 127.4, 127.0, 92.2, 60.6, 41.6, 30.5, 28.5, 14.0 ppm; HRMS (TOF ESI): calcd for $C_{16}H_{20}NO_4$: 290.1387 $[M+H]^+$; found: 290.1383.

Ethyl 3-(2-hydroxy-1-(2-(2-(2-methoxyethoxy)ethoxy)ethyl)-5-oxo-2,5-dihydro-1H-pyrrol-2-yl)propanoate (15)

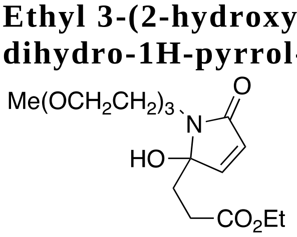

Yield 23% (80.1 mg). ^{1}H NMR (400 MHz, CD_3OD): δ = 7.00 (d, J = 6.0 Hz, 1H), 6.07 (d, J = 6.0 Hz, 1H), 4.10 (m, 2H), 3.63-3.51 (m, 11H), 3.38 (m, 1H), 3.35 (s, 3H), 2.21 (m, 4H), 1.23 (t, J = 7.1 Hz, 3H) ppm; ^{13}C NMR (101 MHz, CD_3OD): δ =174.5, 172.2, 151.6, 127.1, 93.2, 72.9, 71.4, 71.3 (2C), 69.7, 61.7, 59.1, 39.3, 31.7, 29.7,

14.5 ppm; Calculated mass for $C_{16}H_{28}NO_7$: 346.2 $[M+H]^+$; mass found: 346.1 and 328.1 $[M+H-H_2O]^+$.

Ethyl (*Z*)-3-(2-hydroxy-1-(octadec-9-en-1-yl)-5-oxo-2,5-dihydro-1H-pyrrol-2-yl)propanoate (16)

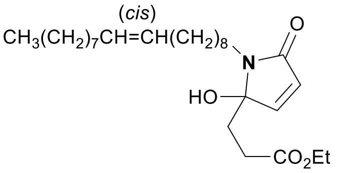

Yield 41% (186 mg). ^{1}H NMR (400 MHz, DMSO-d$_6$): δ = 6.96 (d, *J* = 6.0 Hz, 1H), 6.04 (d, *J* = 6.0 Hz, 1H), 5.32 (m, 2H), 4.02 (q, *J* = 7.1 Hz, 2H), 3.18 (m, 1H), 3.00 (m, 1H), 2.11 (m, 3H), 2.00-1.88 (m, 5H), 1.50 (m, 2H), 1.31-1.24 (m, 22H), 1.16 (t, *J* = 7.1 Hz, 3H), 0.85 (t, *J* = 7.0 Hz, 3H) ppm; ^{13}C NMR (101 MHz, DMSO-d$_6$): δ =172.1, 168.8, 149.4, 129.6 (2C), 126.2, 90.9, 59.9, 37.6, 31.3, 30.7, 29.1 (2C), 29.0, 28.9, 28.8 (2C), 28.7 (2C), 28.6, 28.5, 26.7, 26.6, 26.5, 22.1, 14.0, 13.9 ppm; Calculated mass for $C_{27}H_{48}NO_4$: 450.3 $[M+H]^+$; mass found: 450.3 $[M+H]^+$ and 432.3 $[M+H-H_2O]^+$.

2-(2-(2-ethoxyethoxy)ethoxy)ethyl 3-(1-(5-((5(dimethylamino)naphthalene)-1-sulfonamido)pentyl)-2-hydroxy-5-oxo-2,5-dihydro-1H-pyrrol-2-yl)propanoate (20)

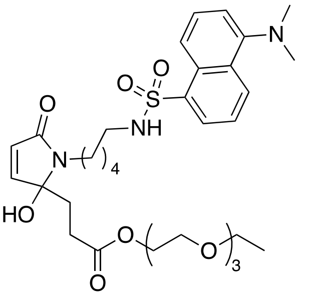

Compound **20** was prepared from **1e** (123.1 mg, 0.41 mmol) according to the general experimental procedure described above for the synthesis of 5HP2O using the dansylcadaverine amine **1F** (151 mg, 0.45 mmol). The resulting product was purified by reverse-phase preparative-HPLC.

Yield 21% (56 mg). ^{1}H NMR (400 MHz, CD_3CN): δ = 8.53 (d, *J* = 8.5 Hz, 1H), 8.28 (d, *J* = 8.7 Hz, 1H), 8.17 (dd, J_1 = 7.3 Hz, J_2 = 1.2 Hz, 1H), 7.60 (d, *J* = 7.6 Hz, 1H), 7.56 (d, *J* = 7.6 Hz, 1H) 7.25 (d, *J* = 7.6 Hz, 1H), 6.86 (d, *J* = 6.0 Hz, 1H), 5.97 (d, *J* = 6.0 Hz, 1H), 4.12 (m, 2H), 3.60 (m, 2H), 3.56-3.47 (m, 8H), 3.44 (m, 2H), 3.14 (m, 1H), 2.92 (m, 1H), 2.85 (s, 6H), 2.80 (t, *J* = 6.8 Hz, 2H), 2.19-2.10 (m, 3H), 1.98 (m, 1H), 1.41-1.27 (m, 4H), 1.16-1.06 (m, 5H) ppm; ^{13}C NMR (101 MHz, CD_3CN): δ = 173.5, 170.3, 153.1, 149.8, 136.7, 131.0, 130.7, 130.5, 130.1, 129.1, 127.7, 124.5, 120.1, 116.3, 92.6, 71.3, 71.2, 71.1, 70.6, 69.7, 67.0, 64.7, 45.8 (2C), 43.8, 38.7, 31.4, 29.7, 29.6, 29.2, 24.8, 15.6 ppm; HRMS (TOF ESI): calculated for $C_{32}H_{48}N_3O_9S$: 650.3033 $[M+H]^+$; mass found: 650.3092 $[M+H]^+$.

Synthesis of compounds 10, 18 and 19

2,5-dioxopyrrolidin-1-yl 6-(2-hydroxy-2-methyl-5-oxo-2,5-dihydro-1H-pyrrol-1-yl)hexanoate (10)

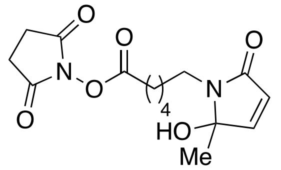

Under argon atmosphere, 5HP2O building block **9** (18 mg, 0.08 mmol) and NHS (27.6 mg, 0.24 mmol) were dissolved in DMF (5mL) and the solution was cooled to 0 °C. A solution of DCC (49.5 mg, 0.24 mmol) in DMF (1mL) was added dropwise and the reaction was stirred for 2 h at 0 °C followed by 10 h at room temperature. The reaction mixture was concentrated *under high vacuo* and the dicyclohexylurea (DCU) was precipitated in MeCN (5 mL). The mixture was filtered and the filtrate was concentrated under reduced pressure. The product was purified by flash column chromatography (silica gel, Hexane:EtOAc, 1:4 → 0:1).

Yield 75% (19.5 mg). ^{1}H NMR (400 MHz, CD_3CN): δ = 6.97 (d, *J* = 5.9 Hz, 1H), 5.97 (d, *J* = 5.9 Hz, 1H), 3.36 (m, 1H), 3.21 (m, 1H), 2.80 (s, 4H), 2.66 (t, *J* = 7.4 Hz, 2H), 1.80-1.72 (m, 2H), 1.69-1.62 (m, 2H) 1.49 (s, 3H), 1.44 (m, 2H) ppm; ^{13}C NMR (101 MHz, CD_3CN): δ = 171.2, 170.1 (2C), 169.9, 151.8, 126.2, 90.7, 38.5, 31.3, 29.3, 26.9, 26.4 (2C), 25.1, 23.4

ppm; HRMS (TOF ESI): calculated for $C_{15}H_{21}N_2O_6$: 325.1321 $[M+H]^+$; masses found: 325.1381 $[M+H]^+$.

2,5-dioxopyrrolidin-1-yl 6-(2-(3-(dodecylamino)-3-oxopropyl)-2-hydroxy-5-oxo-2,5-dihydro-1*H*-pyrrol-1-yl)hexanoate (18)

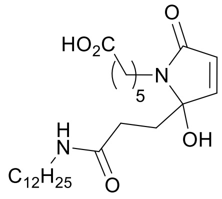

17

Intermediate compound **17** was prepared from furan **1d** (211.6 mg, 0.688 mmol) according to the general experimental procedure described above for the synthesis of 5HP2O using amine **1E** (100 mg, 0.757 mmol, which was first neutralized in MeOH with 2 equiv of Et_3N, 192 µL, 1.38 mmol). The resulting product was purified by reverse-phase preparative HPLC. Yield 24% (75.5mg). ^{1}H NMR (400 MHz, DMSO-d$_6$): δ = 7.74 (t, *J* = 5.5 Hz, 1H), 6.91 (d, *J* = 6.0 Hz, 1H), 6.01 (d, *J* = 6.0 Hz, 1H), 3.24-3.15 (m, 2H), 3.00-2.94 (m, 2H), 2.18 (t, *J* = 7.3 Hz, 2H), 1.91-1.80 (m, 2H), 1.50 (m, 4H) 1.23 (m, 22H), 0.84 (t, *J* = 7.0 Hz, 3H) ppm; Calculated mass for $C_{25}H_{43}N_2O_5$: 451.3 $[M-H]^-$; mass found: 451.3.

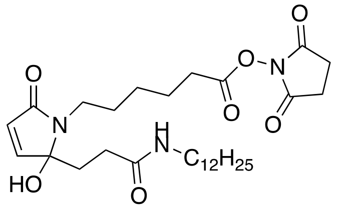

For the synthesis of **18**, intermediate **17** (75.5 mg, 0.167 mmol) and HBTU (95 mg, 0.250 mmol) were dissolved in dry DMF (2 mL) under argon atmosphere. Then a solution of diisopropylethylamine (117 µL, 0.668 mmol) and NHS (38 mg, 0.334 mmol) in DMF (0.5 mL), was added and the solution was stirred overnight at room temperature. The reaction was monitored by tlc analysis. After completion of the reaction, brine was added (10 ml) and the mixture was extracted with EtOAc (2x 10 mL). The combined organic phases were washed with brine (2x 10 mL), dried over $MgSO_4$ and concentrated under reduced pressure. The residue was purified by reverse-phase preparative-HPLC.

Yield 64% (59 mg). ^{1}H NMR (400 MHz, CD_3OD): δ = 6.97 (d, *J* = 6.0 Hz, 1H), 6.05 (d, *J* = 6.0 Hz, 1H), 3.21-3.10 (m, 4H), 2.83 (s, 4H), 2.65 (t, *J* = 7.4 Hz, 2H), 2.07 (m, 2H), 1.81-1.74 (m, 2H), 1.47 (m, 4H) 1.29 (s, 22H), 0.89 (t, *J* = 7.1 Hz, 3H) ppm; ^{13}C NMR (101 MHz, CD_3OD): δ =174.6, 172.0 (2C), 170.3, 170.2, 151.3, 127.4, 93.8, 40.6, 39.5, 33.2, 32.4, 31.6, 31.4, 30.9 (3C), 30.8, 30.6 (2C), 30.5, 29.6, 28.2, 27.5, 26.6 (2C), 25.5, 23.9, 14.6 ppm; Calculated mass for $C_{29}H_{46}N_3O_6$: 532.3 $[M+H-H_2O]^+$; mass found: 532.2.

6-(2-(3-(dodecylamino)-3-oxopropyl)-2-hydroxy-5-oxo-2,5-dihydro-1*H*-pyrrol-1-yl)-*N*-((2*S*,3*R*,4*S*,6*R*)-3-hydroxy-2-methyl-6-(((1*S*,3*S*)-3,5,12-trihydroxy-3-(2-hydroxyacetyl)-10-methoxy-6,11-dioxo-1,2,3,4,6,11-hexahydrotetracen-1-yl)oxy)tetrahydro-2*H*-pyran-4-yl)hexanamide (19)

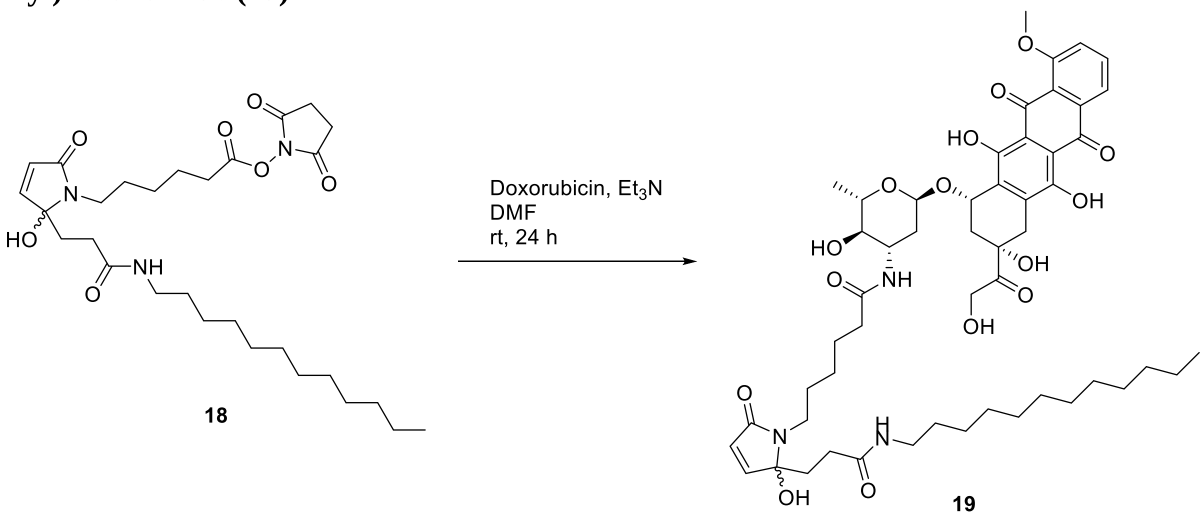

*Scheme S 2: Reaction scheme for the synthesis of compound **19**.*

Compound **19** was prepared by vigorously shaking **18** (2.1 mg, 0.0038 mmol) and an excess of doxorubicin (2.5 mg, 0.00460 mmol) in DMF (1mL), containing Et_3N (2 µL, 0.014 mmol). Progress was followed by RP-HPLC.

LC-MS: m/z $[M-H]^-$ calculated for compound **19**: 976.4885, found: 976.35.

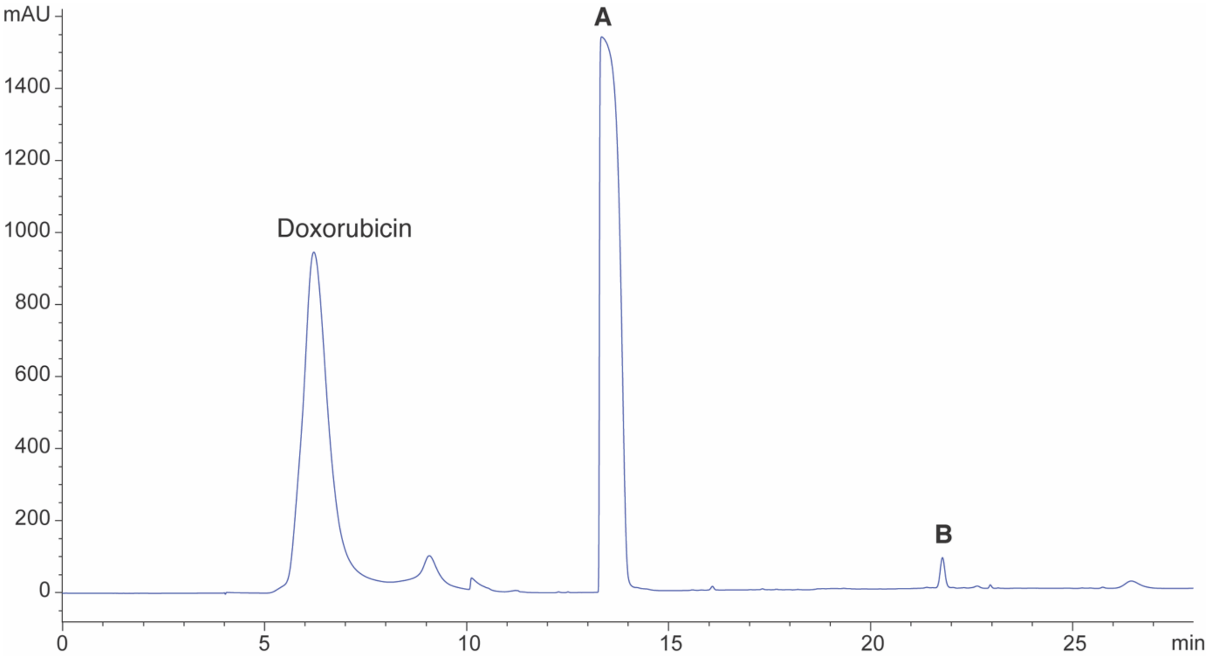

*Figure S 1: RP-HPLC analysis 24 h after the addition of doxorubicin. Product peak **A** (**19**) and starting material peak **B** (**18**).*

Synthesis of 1-benzyl-5-methoxy-5-methyl-1,5-dihydro-2H-pyrrol-2-one (2-OMe)

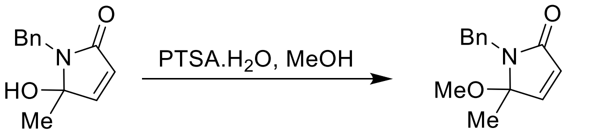

2 **2-OMe**

To a solution of lactam **2** (0.2 mmol, 40.6 mg) in MeOH (2 mL), PTSA·H_2O (38 mg, 0.2 mmol) was added and the solution was stirred at rt until full consumption of the starting material was indicated by tlc analysis (3 h). After completion of the reaction, a saturated aquatic solution of $NaHCO_3$ was added (3 mL) and the mixture was extracted with EtOAc (2x 4 mL). The combined organic layers were dried over Na_2SO_4, filtered and concentrated *in vacue*. The product was used without further purification.

Yield 92% (126 mg). ^{1}H NMR (500 MHz, $CDCl_3$): δ = 7.35 (d, *J* = 7.3 Hz, 2H), 7.29 (m, 2H), 7.23 (m, 1H), 6.76 (d, *J* = 6.0 Hz, 1H), 6.27 (d, *J* = 6.0 Hz, 1H), 4.66 (d, *J* = 15.3 Hz, 1H), 4.26 (d, *J* = 15.3 Hz, 1H), 2.84 (s, 3H), 1.34 (s, 3H) ppm; ^{13}C NMR (125 MHz, $CDCl_3$): δ = 169.5, 148.2, 137.9, 128.4, 128.3 (2C), 128.2 (2C), 127.1, 93.9, 50.5, 41.5, 23.3 ppm.

2. Hydrolytic stability studies of the 5-hydroxy-1H-pyrrol- 2(5H)-one building block (3)

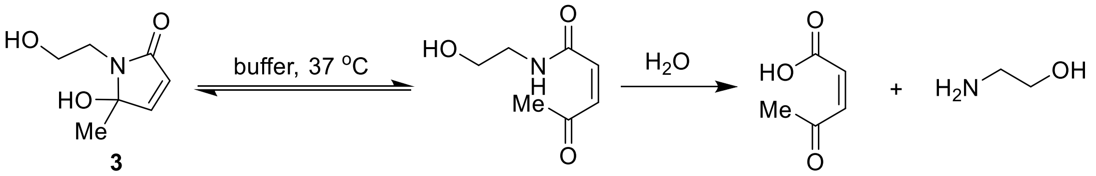

*Scheme S 3: Reaction scheme for the possible hydrolysis of **3** after ring opening.*

The water soluble α,β-unsaturated γ-hydroxylactam **3** was selected as the model compound for the hydrolytic stability study. Compound **3** was dissolved at a 6.75 mM concentration using the following buffers: A) 2-(*N*-morpholino)ethanesulfonic acid (MES) (pH = 6.0; 0.1 M), B) sodium phosphate buffer (pH = 7.0; 0.1 M), C) sodium borate buffer (pH = 8.0; 0.1 M) incubated at 37 °C and was shaken in dark for 24 h. The stability was checked using RP-HPLC. Analysis was performed on Phenomenex Luna C18 column with a flow rate of 1 mL/min. The following gradient was employed: 3 min 100% H_2O followed by 0 to 100% MeCN in 20 min and 5 minutes at 100% MeCN.

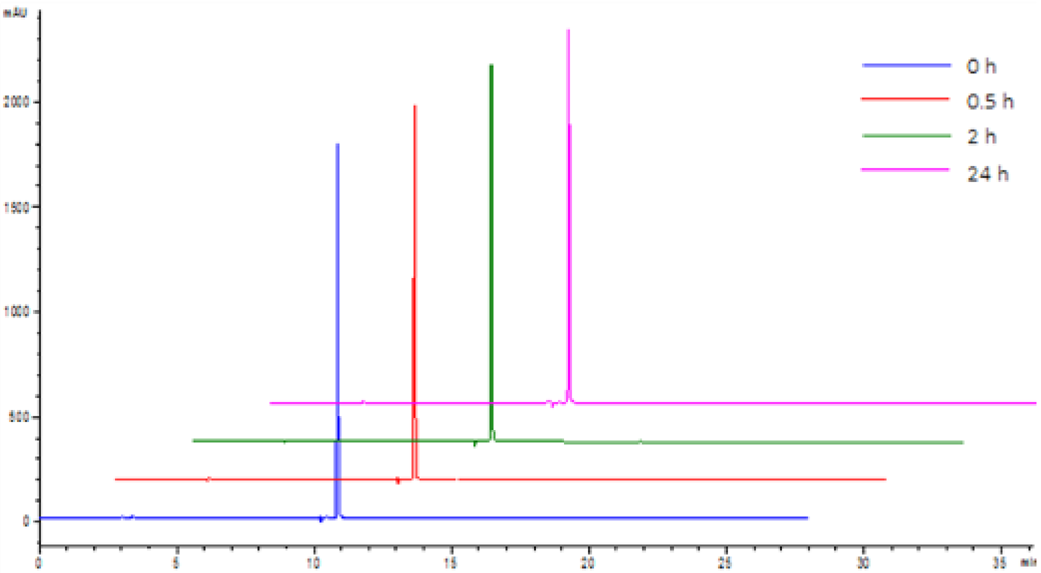

*Figure S 2: Hydrolytic stability test of 5HP2O building block **3** in buffer A (pH 6.0). HPLC traces (214 nm) are shown at different reaction time points. No hydrolysis product was observed.*

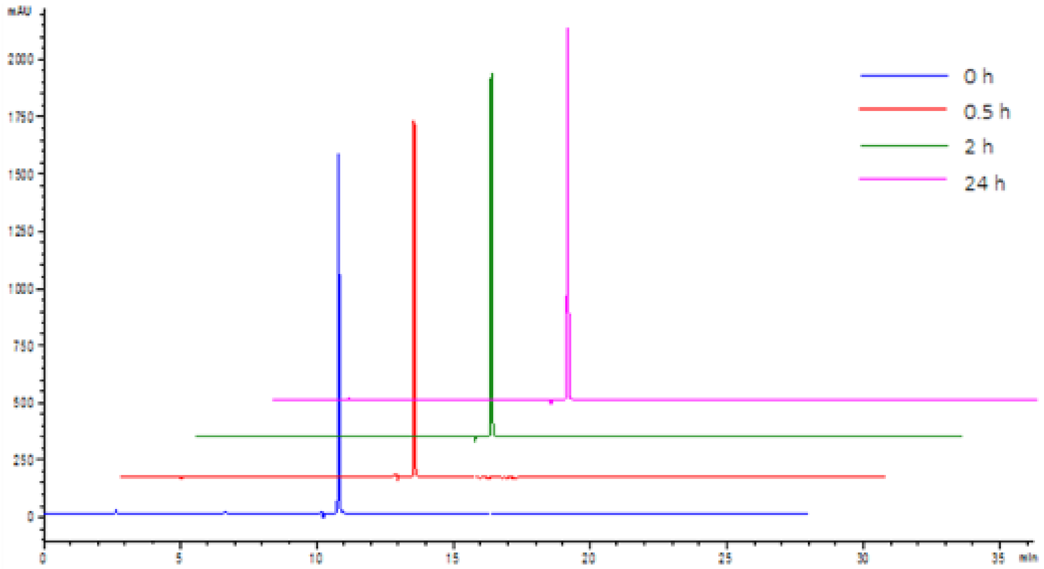

*Figure S 3: Stability test of 5HP2O building block **3** in buffer B (pH 7.0). HPLC traces (214 nm) are shown at different reaction time points. No hydrolysis product was observed.*

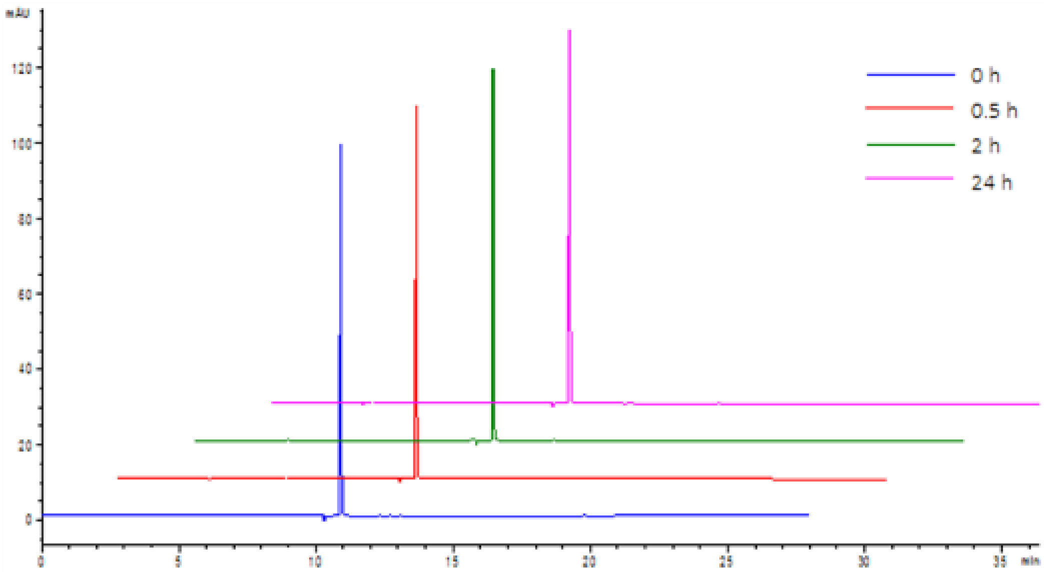

*Figure S 4: Stability test of 5HP2O building block **3** in buffer C (pH 8.0). HPLC traces (214 nm) are shown at different reaction time points. No hydrolysis product was observed.*

NMR analysis

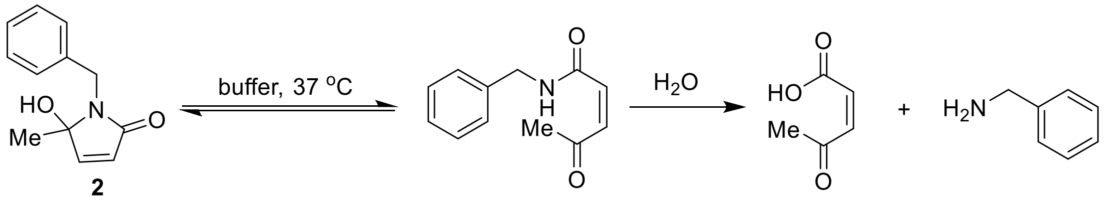

2

*Scheme S 4: Reaction scheme for the possible hydrolysis of **2** after ring opening.*

Compound **2** was dissolved at a 5 mM concentration using PBS pH8 and MeOH mixture (1/1). After 6 hours structure of **2** was determined by NMR.

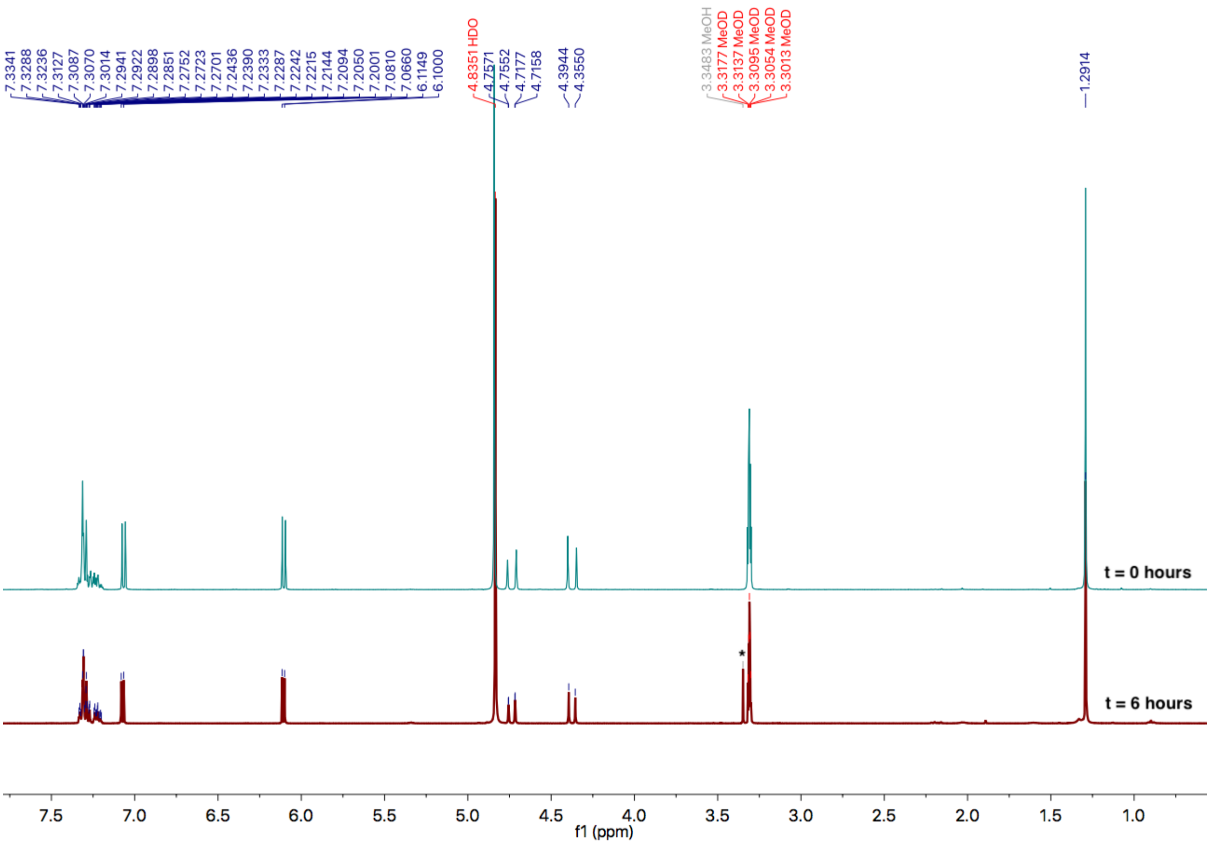

*Figure S 5: Overlay ^{1}H-NMR from before (green) and after (red) incubation in buffer/MeOH mixture. Residual MeOH peak indicated with *.*

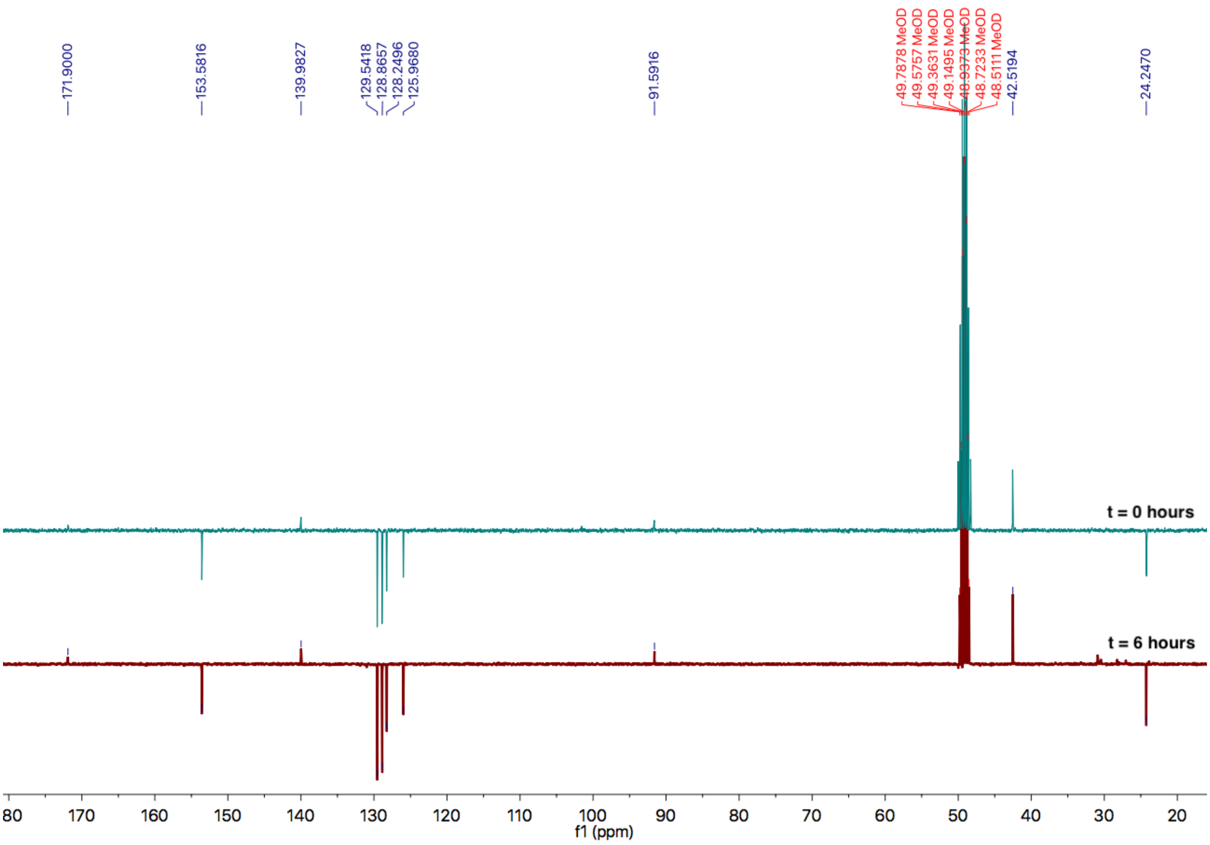

Figure S 6: Overlay ^{13}C-APT from before (green) and after (red) incubation in buffer/MeOH mixture.

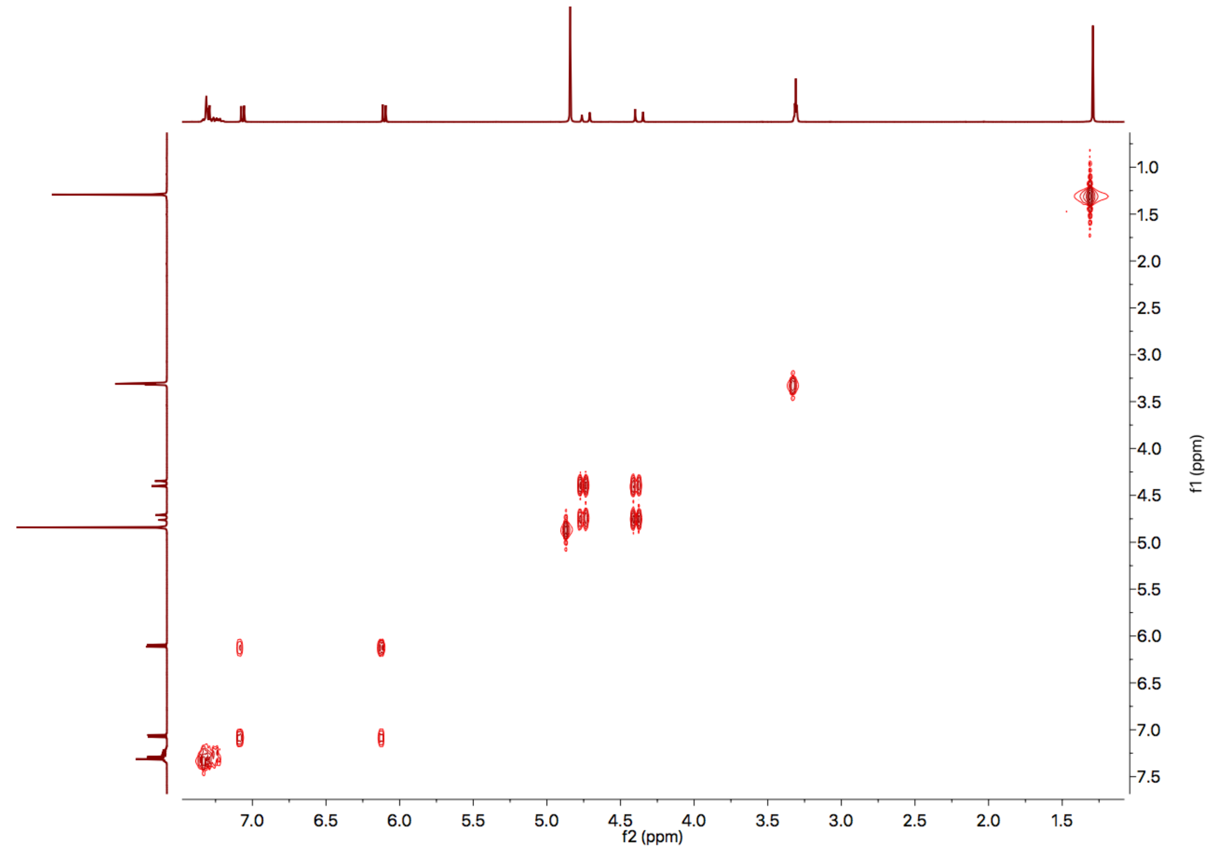

*Figure S 7: COSY-NMR of compound **2** before incubation in buffer/MeOH.*

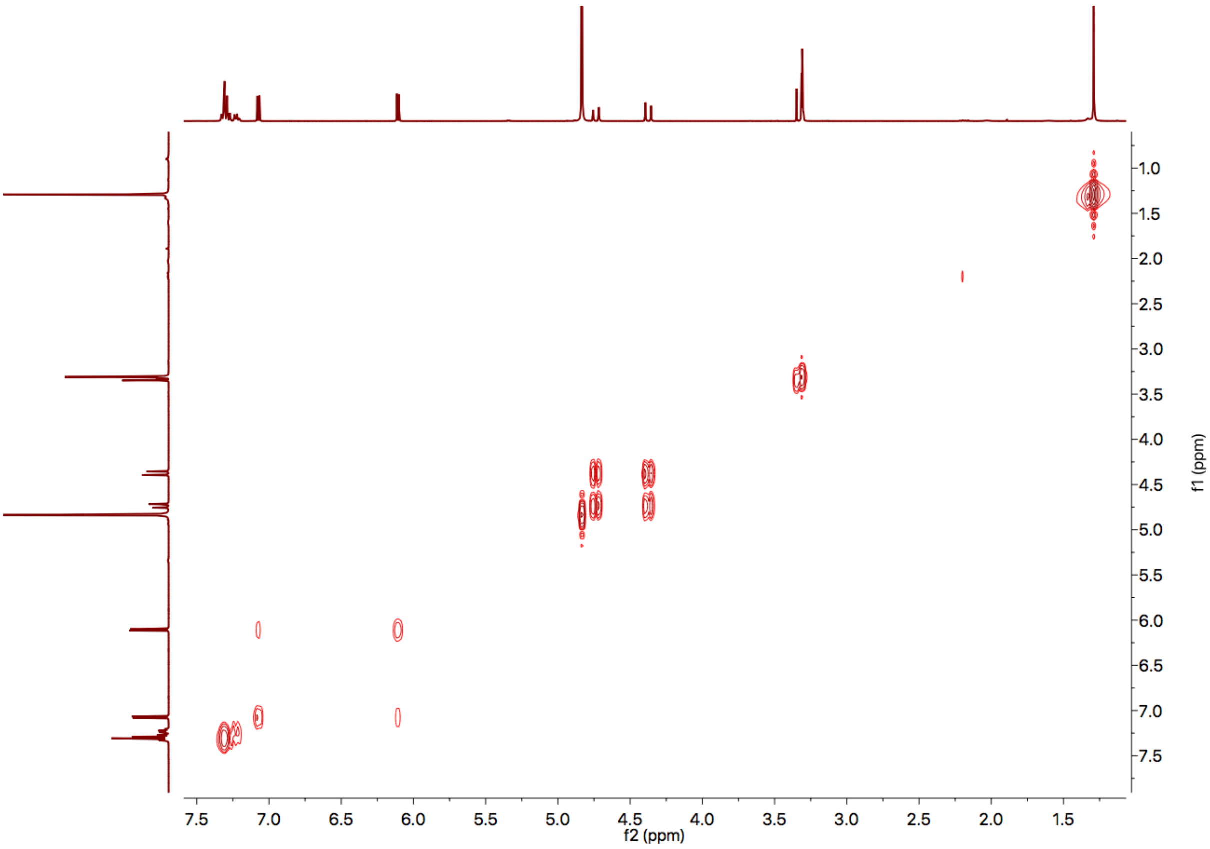

*Figure S 8: COSY-NMR of compound **2** after incubation in buffer/MeOH.*

3. Hydrolytic stability of N-Benzylmaleimide

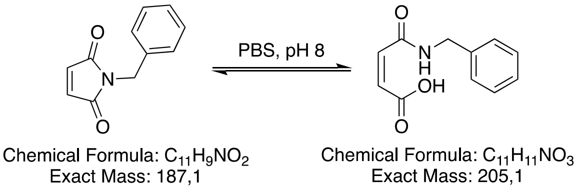

Scheme S 5: Hydrolysis of N-benzylmaleimide.

N-benzylmaleimide (20 µL out of a 10 mg/mL stock solution in MeCN) was incubated in PBS (phosphate-buffered saline) buffer (pH 8.0) at a 5 mM concentration at 25 °C. The stability was checked using RP-HPLC. Analysis was performed on a Phenomenex Luna C18 column with a flow rate of 1 mL/min. The following gradient was used: 3 min 100% H_2O followed by 0 to 100% MeCN in 20 min and 5 min 100% MeCN.

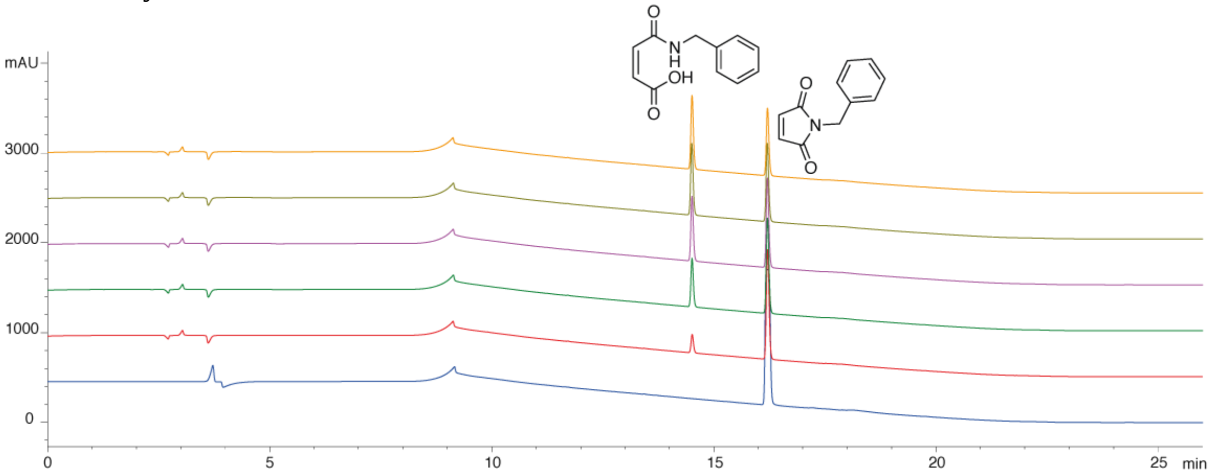

*Figure S 9: HPLC follow up on N-benzylmaleimide hydrolysis in PBS buffer (pH 8). Even though this test was performed at rt instead of 37 °C (in case of **3**), a significant amount (roughly 50%) of hydrolyzed product was observed after 2.5 hours (left peak).*

4. Reactivity of 5HP2O building blocks towards thiols

4.1. *Conjugation to thiols in MeOH*

4.1.1. General procedure for the preparation of 5-hydroxy-3-((2- hydroxyethyl)thio)-pyrrolidin-2-ones

General experimental procedure for the thiol addition to 5HP2O in MeOH (conditions A)

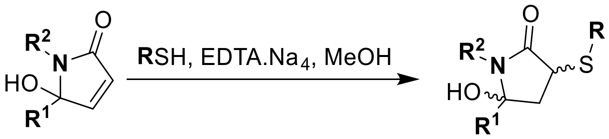

The purified lactams (0.1 mmol, 20.3 mg of **2**, 15.1 mg of **7**, 32.7 mg of **8**, 28.9 mg of **14**) were dissolved in MeOH (1 mL) and the corresponding thiol was added (9.2 μL, 0.13 mmol of β-mercaptoethanol towards conjugates **21-24** or 13 μL, 0.11 mmol of benzyl mercaptan towards conjugate **4**) followed by EDTA.Na4 (11.4 mg, 0.03 mmol, dissolved in 50 μL of water). The solution was stirred at rt until full consumption of the starting material was indicated by tlc analysis (2 h, using β-mercaptoethanol or 1 h using benzyl mercaptan). After completion of the reaction the solution was concentrated *in vacuo* and the conjugates were purified by flash column chromatography.
The diastereoisomers of conjugate **4** were separated and fully characterized by 1D and 2D-NMR in order to assign the regioselectivity of the thiol addition reaction.
Compound **2-OMe** (21.7 mg, 0.1 mmol) was also subjected to benzyl mercaptan (13 μL, 0.11 mmol) under conditions A for 24 h, however the reaction did not proceed.

In the context of the optimization of the reaction conditions, two additional methods were employed. Preliminary experiments were conducted using method A where beta-mercaptoethanol was added to a solution of the unsaturated lactams in MeOH, whereas subsequent experiments were conducted using method B, in an attempt towards optimization of the conjugation conditions. In method B, mercaptoethanol was pretreated with the reducing agent TCEP prior to its addition to the solution of the unsaturated lactams. The processes are described in detail as follows:

A: The purified lactams (0.12 mmol, 25 mg for **2**, 0.15 mmol, 23 mg for **7** and 0.17 mmol or 50 mg for **14**) were dissolved in MeOH (2 mL) and β-mercaptoethanol (0.16 mmol, 11.2 μL for **2**, 0.19 mmol, 14 μL for **7** or 0.22 mmol, 16 μL for **14**) was added. The mixtures were left for 18 h stirring and subsequently concentrated *in vacuo*.

B: The purified lactams (0.06 mmol, 18 mg for **8**) were dissolved in MeOH (2 mL) and β-mercaptoethanol (0.07 mmol, 4.8 μL for **8**) pretreated with TCEP (0.05 mmol, 14 mg for **8**) for 30 min, was added. TCEP-HCl was neutralized with 10% NaOH prior to its use. The mixture was left to react for 30 min for the preparation of **23**. After completion of the reactions, as indicated by tlc analysis, the mixtures were concentrated in vacuo and washed with EtOAc or CH_2Cl_2/Brine.

1-benzyl-3-(benzylthio)-5-hydroxy-5-methylpyrrolidin-2-one (4)

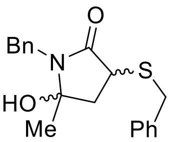

The reaction was accomplished according to the general experimental procedure described above affording **4** as a 1.8:1 mixture of diastereoisomers (separable). The conjugate 4 was purified by flash column chromatography (silica gel, petroleum ether:EtOAc, 10:1 → 5:1). Yield 70% for both isomers (22.9 mg). Yield 45% (14.7 mg), for the major isomer, 25 % for the minor isomer (8.2 mg).

Minor (less polar). ^{1}H NMR (500 MHz, $CDCl_3$): δ = 7.44 (d, *J* = 7.2 Hz, 2H), 7.35-7.25 (m, 8H), 4.69 (d, *J* = 15.4 Hz, 1H), 4.39 (d, *J* = 15.4 Hz, 1H), 4.27 (d, *J* = 13.4 Hz, 1H), 3.90 (d, *J* = 13.4 Hz, 1H), 3.40 (d, J_1 = 8.4 Hz, J_2 = 1.8 Hz, 1H), 2.76 (s, 1H), 2.45 (d, J_1 = 14.4 Hz, J_2 = 8.4 Hz, 1H), 1.95 (d, J_1 = 14.4 Hz, J_2 = 1.8 Hz, 1H), 1.34 (s, 3H) ppm; ^{13}C NMR (125 MHz, $CDCl_3$): δ = 173.2, 138.3, 137.2, 129.3 (2C), 128.6 (4C), 127.7 (2C), 127.3, 127.2, 89.4, 42.7, 41.8, 38.7, 35.9, 26.2 ppm; HRMS (Orbitrap ESI): calculated for $C_{19}H_{20}NOS$: 310.1260 [M-H_2O+H]$^+$; found 310.1265.

Major (more polar). ^{1}H NMR (500 MHz, $CDCl_3$): δ = 7.41 (d, *J* = 7.3 Hz, 2H), 7.31 (m, 6H), 7.25 (m, 2H), 4.54 (d, *J* = 15.4 Hz, 1H), 4.45 (d, *J* = 15.4 Hz, 1H), 4.23 (d, *J* = 13.3 Hz, 1H), 3.91 (d, *J* = 13.3 Hz, 1H), 3.52 (d, J_1 = 9.0 Hz, J_2 = 6.9 Hz, 1H), 2.50 (brs, 1H), 2.40 (d, J_1 = 14.0 Hz, J_2 = 9.0 Hz, 1H), 1.94 (d, J_1 = 14.0 Hz, J_2 = 6.9 Hz, 1H), 1.41 (s, 3H) ppm; ^{13}C NMR (125 MHz, $CDCl_3$): δ = 174.0, 138.0, 137.6, 129.2 (2C), 128.7 (2C), 128.5 (2C), 127.7 (2C), 127.4, 127.2, 88.8, 42.7, 42.4, 39.7, 35.7, 27.2 ppm; HRMS (Orbitrap ESI): calculated for $C_{19}H_{22}NO_2S$: 328.1366 [M+H]$^+$; found 328.1358.

Selected HMBC correlations for both isomers

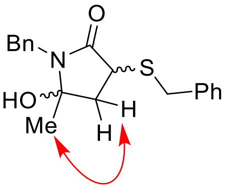

1-benzyl-5-hydroxy-3-((2-hydroxyethyl)thio)-5-methylpyrrolidin-2-one (21)

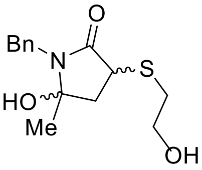

The reaction was accomplished according to the general experimental procedure described above affording **21** as a 1.5:1 mixture of diastereoisomers (separable). The conjugate **21** was purified by flash column chromatography (silica gel, petroleum ether:EtOAc, 2:1 → 1:1). Yield 78% for both isomers (21.9 mg). Yield 48% (13.5 mg), for the major isomer, 30 % for the minor isomer (8.4 mg).

Minor (less polar). ^{1}H NMR (500 MHz, CDCl3): δ = 7.35-7.23 (m, 5H), 4.63 (d, *J* = 15.3 Hz, 1H), 4.42 (d, J = 15.3 Hz, 1H), 3.91 (m, 2H), 3.74 (dd, J_1 = 8.6 Hz, J_2 = 3.2 Hz, 1H), 3.14 (ddd, J_1 = 14.6 Hz, J_2 = 6.6 Hz, J_3 = 3.7 Hz, 1H), 2.86 (m, 1H), 2.59 (dd, J_1 = 14.4 Hz, J_2 = 8.6 Hz, 1H), 2.05 (dd, J_1 = 14.4 Hz, J_2 = 3.2 Hz, 1H), 1.37 (s, 3H) ppm; ^{13}C NMR (125 MHz, CDCl3): δ = 174.1, 137.9, 128.6 (2C), 127.8 (2C), 127.4, 89.3, 62.7, 42.9, 42.8, 40.9, 36.0, 26.2 ppm; HRMS (Orbitrap ESI): calculated for $C_{14}H_{20}NO_3S$: 282.1158 [M+H]$^+$; found 282.1150.

Major (more polar). ^{1}H NMR (500 MHz, CDCl3): δ = 7.33-7.24 (m, 5H), 6.08 (brs, 1H), 4.69 (d, *J* = 15.5 Hz, 1H), 4.52 (brs, 1H), 4.36 (d, *J* = 15.5 Hz, 1H), 3.85 (m, 2H), 3.74 (t, *J* = 9.0 Hz, 1H), 2.96 (dt, J_1 = 15.2 Hz, J_2 = 3.7 Hz, 1H), 2.79 (m, 1H), 2.67 (dd, J_1 = 14.1 Hz, J_2 = 9.0 Hz, 1H), 1.96 (dd, J_1 = 14.1 Hz, J_2 = 9.0 Hz, 1H), 1.36 (s, 3H) ppm; ^{13}C NMR (125 MHz, CDCl3): δ = 176.7, 137.6, 128.7 (2C), 127.6 (2C), 127.4, 88.2, 63.2, 43.8, 43.4, 42.8, 38.1, 27.1 ppm; HRMS (TOF ESI): calculated for $C_{14}H_{20}NO_3S$: 282.1158 [M+H]$^+$; found: 282.1172.

5-hydroxy-3-((2-hydroxyethyl)thio)-5-methyl-1-(prop-2-yn-1-yl)pyrrolidin-2-one (22)

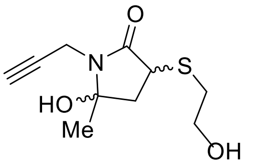

The reaction was accomplished according to the general experimental procedure described above affording **22** as a 1.5:1 mixture of diastereoisomers (inseparable). The conjugate **22** was purified by flash column chromatography (silica gel, petroleum ether:EtOAc, 2:1 → 1:2). Yield 80% for both isomers (18.3 mg).

^{1}H NMR (500 MHz, $CDCl_3$): δ = 4.15 (dd, J_1 = 17.7 Hz, J_2 = 2.3 Hz, 1H for minor), 4.11 (m, 2H for major), 4.02 (dd, J_1 = 17.7 Hz, J_2 = 2.3 Hz, 1H for minor), 3.82 (m, 2H for major plus 2H for minor), 3.73 (t, J = 8.9, 1H for major), 3.60 (dd, J_1 = 9.0 Hz, J_2 = 4.9 Hz, 1H for minor), 3.04 (dt, J_1 = 14.6 Hz, J_2 = 5.2 Hz, 1H for minor), 2.93 (dt, J_1 = 15.0 Hz, J_2 = 4.0 Hz, 1H for major), 2.82 (m, 1H for major plus 1H for minor), 2.65 (m, 1H for major plus 1H for minor), 2.24 (t, J = 2.3, 1H for major), 2.20 (t, J = 2.3, 1H for minor), 2.05 (dd, J_1 = 14.2 Hz, J_2 = 4.9 Hz, 1H for minor), 1.95 (dd, J_1 = 14.0 Hz, J_2 = 8.9 Hz, 1H for major), 1.64 (s, 3H for major), 1.59 (s, 3H for minor) ppm; ^{13}C NMR (125 MHz, $CDCl_3$): δ = 175.4 (major), 173.2 (minor), 88.6 (minor), 87.9 (major), 79.0 (minor), 78.9 (major), 71.6 (major), 71.3 (minor), 62.7 (major), 62.2 (minor), 43.4 (major), 43.1 (major), 42.9 (minor), 41.5 (minor), 37.5 (major), 35.9 (minor), 28.0 (major), 27.9 (minor), 26.5 (major), 25.8 (minor); HRMS (TOF ESI): calculated for $C_{10}H_{15}NO_3S$: 230.0845 $[M+H]^+$; found: 230.0840.

5-hydroxy-3-((2-hydroxyethyl)thio)-5-methyl-1-(pyren-1-ylmethyl)pyrrolidin-2-one (23)

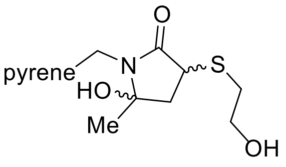

The reaction was accomplished according to the general experimental procedure described above affording **23** as a 1.5:1 mixture of diastereoisomers (separable). The conjugate **23** was purified by flash column chromatography (silica gel, petroleum ether:EtOAc, 2:1 → 1:2). Yield 67% for both isomers (27.1 mg). Yield 40% (16.2 mg), for the major isomer.

Major (less polar): ^{1}H NMR (500 MHz, $CDCl_3$): δ = 8.40 (d, J = 9.3 Hz, 1H), 8.20 (d, J = 7.6 Hz, 1H), 8.20 (d, J = 7.4 Hz, 1H), 8.16 (d, J = 9.3 Hz, 1H), 8.13 (d, J = 7.8 Hz, 1H), 8.03 (m, 3H), 7.96 (d, J = 7.8 Hz, 1H), 5.35 (d, J = 15.7 Hz, 1H), 5.30 (d, J = 15.7 Hz, 1H), 4.02-3.88 (m, 2H), 3.84 (dd, J_1 = 8.6 Hz, J_2 = 3.2 Hz, 1H), 3.23 (ddd, J_1 = 14.7 Hz, J_2 = 6.5 Hz, J_3= 3.7 Hz, 1H), 2.90 (m, 2H), 2.62 (dd, J_1 = 14.4 Hz, J_2 = 8.6 Hz, 1H), 2.08 (dd, J_1 = 14.4 Hz, J_2 = 3.2 Hz, 1H), 1.28 (s, 3H) ppm; ^{13}C NMR (125 MHz, $CDCl_3$): δ = 174.1, 131.3, 130.9, 130.7, 130.6, 128.5, 128.1, 127.4 (2C), 126.3, 126.1, 125.4, 125.3, 124.9, 124.8, 124.7, 122.7, 89.7, 62.8, 42.9, 41.1, 40.9, 36.1, 26.2 ppm.

Ethyl 3-(1-benzyl-2-hydroxy-4-((2-hydroxyethyl)thio)-5-oxopyrrolidin-2-yl)propanoate (24)

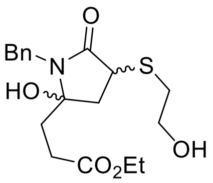

The reaction was accomplished according to the general experimental procedure described above affording **24** as a 1.5:1 mixture of diastereoisomers (inseparable). The conjugate **24** was purified by flash column chromatography (silica gel, petroleum ether:EtOAc, 2:1 → 1:1). After the chromatographic purification the dr value was increased to 5.5:1. Yield 65% (23.8 mg).

^{1}H NMR (500 MHz, $CDCl_3$): δ = 7.35-7.22 (m, 5H), 5.58 (brs, 1H), 4.76 (brs, 1H), 4.68 (d, J = 15.5 Hz, 1H), 4.35 (d, J = 15.5 Hz, 1H), 4.09 (q, J = 7.2 Hz, 2H), 3.85 (m, 2H), 3.74 (t, J = 8.8 Hz, 1H), 2.97 (dt, J_1 = 14.9 Hz, J_2 = 4.1 Hz, 1H), 2.82 (dt, J_1 = 14.9 Hz, J_2 = 5.7 Hz, 1H),

2.78-2.70 (m, 1H), 2.56 (dd, J_1 = 14.0 Hz, J_2 = 8.8 Hz, 1H), 2.38 (td, J_1 = 16.8 Hz, J_2 = 7.8 Hz, 1H), 2.27-2.14 (m, 2H), 1.92 (dd, J_1 = 14.0 Hz, J_2 = 8.8 Hz, 1H), 1.72 (m, 1H), 1.22 (t, J = 7.2, 3H); ^{13}C NMR (125 MHz, $CDCl_3$): δ = 176.7, 173.7, 137.5, 128.7 (2C), 127.8 (2C), 127.5, 89.9, 62.9, 61.0, 42.9, 42.8, 41.0, 37.8, 34.2, 28.8, 14.1 ppm.

4.2. Conjugation to thiols in H_2O/MeCN

General experimental procedure for the thiol addition to 5HP2O in H_2O/MeCN 3/2 v/v (conditions B)

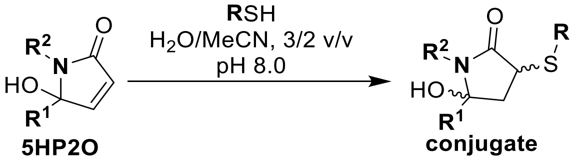

The purified lactams (0.2 mmol, 40.6 mg of **2**, 31.4 mg of **3**, 39 mg of **12**, 47.4 mg of **13**) were dissolved in H_2O/MeCN (3/2 v/v, 5 mL for **2** and **3**, 2 mL for **12** and **13**) and the pH was adjusted to 8.0 with 0.1 M NaOH solution. The corresponding thiol was added (0.22 mmol, 23.7 μL, of 1-butanethiol towards the conjugates **5** and **6** or 0.26 mmol, 18.3 μL of β-mercaptoethanol towards conjugates **25** and **26**) and the solution was stirred for 1 h (towards **5** and **6**) or 3 h (towards **25** and **26**) at rt, until full consumption of the starting material was indicated by tlc analysis (the reaction of **2** and **3** with 1-butanethiol was also monitored by LC-MS analysis of the crude reaction mixture). After completion of the reaction the solution was concentrated *in vacuo* and the conjugates were purified by flash column chromatography.

1-benzyl-3-(butylthio)-5-hydroxy-5-methylpyrrolidin-2-one (5)

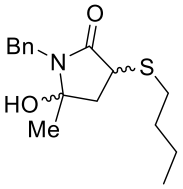

The reaction was accomplished according to the general experimental procedure described above affording **5** as a 1.5:1 mixture of diastereoisomers (separable). The conjugate **5** was purified by flash column chromatography (silica gel, hexane:EtOAc, 4:1 → 2:1). Yield 40% for both isomers (23 mg). Yield 23% (14 mg), for the major isomer (more polar).

^{1}H NMR (400 MHz, CD_3CN): δ = 7.33-7.22 (m, 5H), 4.58 (d, J = 15.6 Hz, 1H), 4.25 (d, J = 15.6 Hz, 1H), 3.99 (brs, 1H), 3.71 (dd, J_1 = 8.8 Hz, J_2 = 7.5 Hz, 1H), 2.76 (m, 2H), 2.52 (dd, J_1 = 13.8 Hz, J_2 = 8.8 Hz, 1H), 2.02 (dd, J_1 = 13.8 Hz, J_2 = 7.5 Hz, 1H), 1.59 (m, 2H), 1.41 (m, 2H), 1.33 (s, 3H), 0.92 (t, J = 7.4 Hz, 3H) ppm; ^{13}C NMR (101 MHz, CD_3CN): δ = 175.04, 140.37, 129.70 (2C), 128.63 (2C), 128.21, 89.36, 44.23, 43.38, 42.62, 32.58, 31.78, 28.04, 23.09, 14.36 ppm.

HRMS (TOF ESI): calculated for $C_{16}H_{23}NO_2S$: 294.1449 $[M+H]^+$, observed masses: 294.1531 $[M+H]^+$.

Selected HMBC correlations

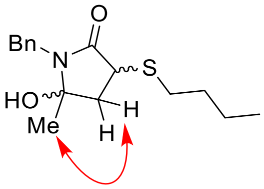

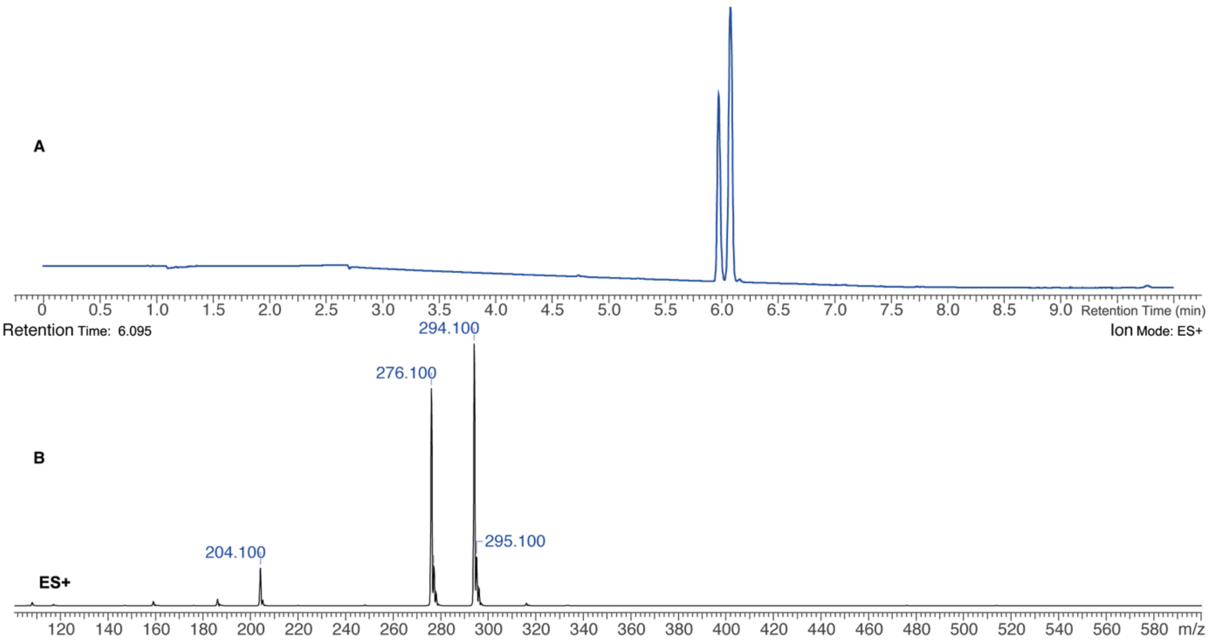

*Figure S 10: LC-MS analysis of the crude mixture of 1-butanethiol conjugation experiment. (A) Liquid chromatogram at 214 nm. (B) Mass spectrum of product conjugate **5**. Two peaks are observed due to diastereomeric mixture.*

3-(butylthio)-5-hydroxy-1-(2-hydroxyethyl)-5-methylpyrrolidin-2-one (6)

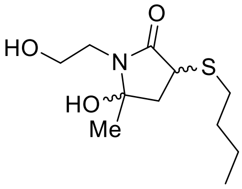

The reaction was accomplished according to the general experimental procedure described above affording **6** as a 1.5:1 mixture of diastereoisomers. The conjugate **6** was purified by preparative RP-HPLC. Yield 42% for both isomers (21 mg). Yield 25% (13 mg), for the major isomer (more polar).

^{1}H NMR (400 MHz, DMSO-d$_6$): δ =5.91 (s, 1H), 4.78 (t, J = 6.5 Hz, 1H), 3.55 (dd, J_1 = 9.1 Hz, J_2 = 8.0 Hz, 1H), 3.45 (m, 2H), 3.26 (dt, J_1 = 13.8 Hz, J_2 = 6.9 Hz, 1H), 3.09 (dt, J_1 = 13.8 Hz, J_2 = 6.9 Hz, 1H), 2.66, (m, 2H), 2.53 (m, 1H), 1.82 (dd, J_1 = 13.1 Hz, J_2 = 8.1 Hz, 1H), 1.51 (m, 2H), 1.37 (m, 2H), 1.33 (s, 3H), 0.87 (t, J = 7.3 Hz, 3H) ppm; ^{13}C NMR (101 MHz, DMSO-d$_6$): δ 171.3, 86.6, 58.9, 42.9, 41.5, 41.1, 31.0, 30.0, 26.2, 21.4, 13.5 ppm.

HRMS (TOF ESI): calculated for $C_{11}H_{21}NO_3S$: 248.1242 $[M+H]^+$, observed masses: 248.1310 $[M+H]^+$.

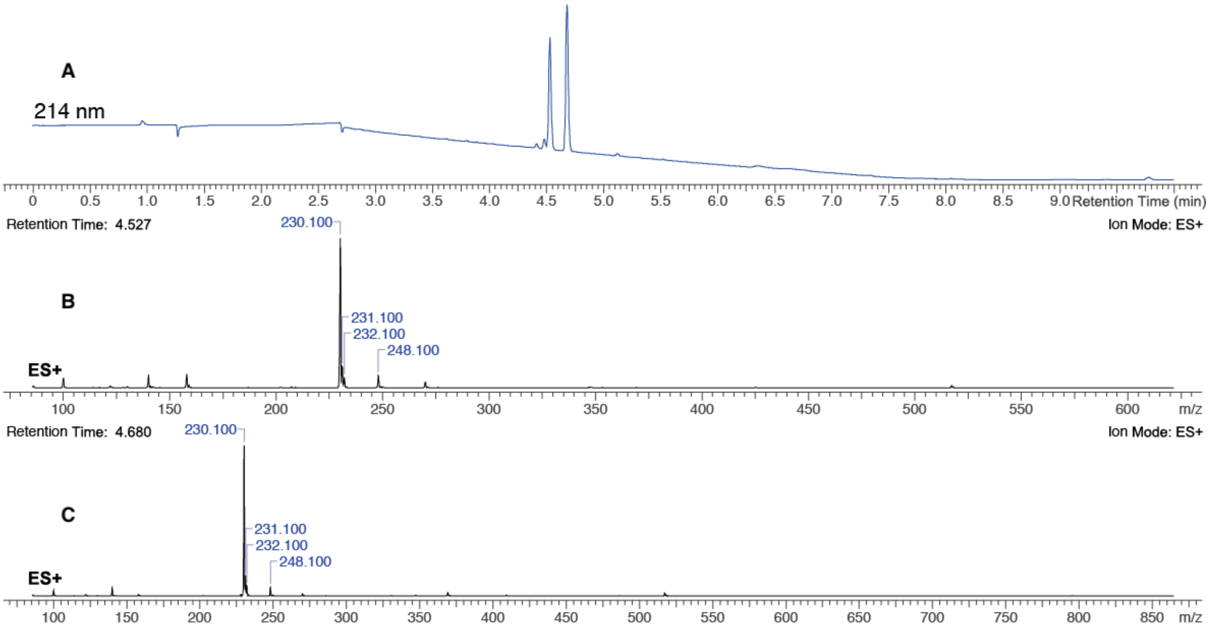

*Figure S 11: LC-MS analysis of the crude mixture of 1-butanethiol conjugation experiment with compound **3**. (A) Liquid chromatogram at 214 nm. (B) Mass spectrum of peak at 4.52 min. (C) Mass spectrum peak at 4.68 min. Two peaks are observed due to diastereomeric mixture.*

5-hydroxy-3-((2-hydroxyethyl)thio)-5-(3-hydroxypropyl)-1-(prop-2-yn-1-yl)pyrrolidin-2-one (25)

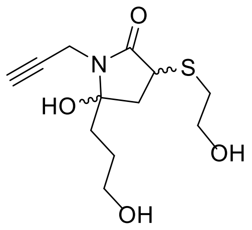

The reaction was accomplished according to the general experimental procedure described above affording **25** as a 1:1 mixture of diastereoisomers (inseperable). The conjugate **25** was purified by flash column chromatography (silica gel, EtOAc). Yield 69% (37.7 mg). ^{1}H NMR (500 MHz, CD_3OD): δ = 4.13 (dd, J_1 = 17.7 Hz, J_2 = 2.5 Hz, 1H for one isomer), 4.11 (dd, J_1 = 17.7 Hz, J_2 = 2.5 Hz, 1H for one isomer), 3.93 (dd, J_1 = 17.7 Hz, J_2 = 2.5 Hz, 1H for one isomer), 3.91 (dd, J_1 = 17.7 Hz, J_2 = 2.5 Hz, 1H for one isomer), 3.76-3.50 (m, 5H for each isomer), 2.95 (m, 1H for each isomer), 2.81 (m, 1H for each isomer plus 1H for one isomer), 2.56 (m, 1H for each isomer), 2.47 (dd, J_1 = 14.1 Hz, J_2 = 9.4 Hz, 1H for one isomer), 2.11 (dd, J_1 = 14.1 Hz, J_2 = 7.5 Hz, 1H for one isomer), 2.01 (m, 1H for one isomer), 1.96-1.75 (m, 2H for each isomer), 1.70-1.52 (m, 2H for each isomer) ppm; ^{13}C NMR (125 MHz, CD_3OD): δ 174.4 (one isomer), 173.1 (one isomer), 90.1 (one isomer), 89.7 (one isomer), 78.7 (one isomer), 78.5 (one isomer), 70.7 (one isomer), 61.2 (one isomer), 61.1 (1C for each isomer), 61.0 (one isomer), 42.2 (one isomer), 41.3 (one isomer), 40.4 (one isomer), 39.7 (one isomer), 35.1 (one isomer), 34.8 (one isomer), 33.9 (one isomer), 33.8 (one isomer), 27.2 (one isomer), 26.9 (one isomer), 26.7 (one isomer), 26.6 (one isomer), ppm; HRMS (Orbitrap ESI): calculated for $C_{12}H_{20}NO_4S$: 274.1108 $[M+H]^+$; found 274.1110.

Ethyl 3-(2-hydroxy-5-oxo-1-(prop-2-yn-1-yl)-2,5-dihydro-1H-pyrrol-2-yl)propanoate (26)

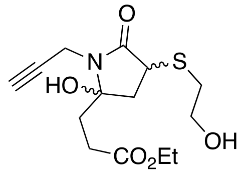

The reaction was accomplished according to the general experimental procedure described above affording **26** as a 1:1 mixture of diastereoisomers. The conjugate **26** was purified by flash column chromatography (silica gel, EtOAc). Yield 65% for both isomers (41 mg). Yield 30% (18.9 mg) for one isomer (more polar).

^{1}H NMR (300 MHz, $CDCl_3$): δ = 4.16 (q, *J* = 7.1 Hz, 2H), 4.14 (m, 2H), 3.95 (dd, J_1 = 17.6 Hz, J_2 = 2.5 Hz, 1H), 3.67 (dd, J_1 = 14.6 Hz, J_2 = 9.3 Hz, 1H), 3.44 (d, *J* = 8.4 Hz, 1H), 2.95 (m, 2H), 2.74, (dd, J_1 = 15.5 Hz, J_2 = 7.0 Hz, 1H), 2.57-2.36 (m, 5H), 2.19 (t, *J* = 2.5 Hz, 1H), 2.06 (m, 1H), 1.27 (t, *J* = 7.1 Hz, 3H) ppm; ^{13}C NMR (75 MHz, $CDCl_3$): δ 175.1, 172.6, 94.0, 78.1, 71.5, 67.4, 60.7, 40.2, 36.3, 33.7, 31.3, 28.9, 27.9, 14.2 ppm; HRMS (Orbitrap ESI): calculated for $C_{14}H_{22}NO_5S$: 316.1213 $[M+H]^+$; found 316.1207.

5. Stability of 5-hydroxy-3-((2- hydroxyethyl)thio)-pyrrolidin-2-one (21)

5.1. Hydrolytic stability studies

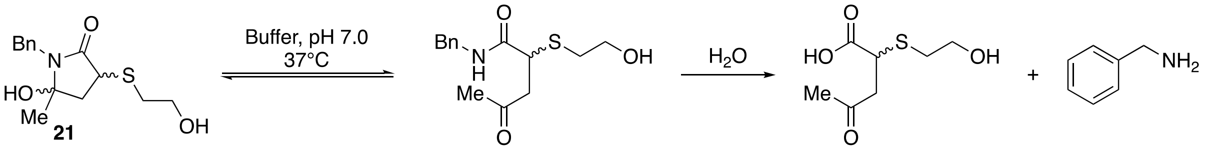

Scheme S 6: Reaction scheme of ring-opening followed by possible hydrolysis.

Compound **21** was dissolved at a 2 mM concentration using the following buffers: A) MES (pH = 6.0; 0.1 M), B) sodium phosphate buffer (pH = 7.0; 0.1 M) and C) sodium borate buffer (pH = 8.0; 0.1 M). The solutions were incubated at 37 °C and shaken for 24 h in the dark. Analysis was performed on Phenomenex Luna C18 column with a flow rate of 1 mL/min. The following gradient was employed: 3 min 100% H_2O followed by 0 to 100% MeCN in 20 min and 5 min at 100% MeCN.

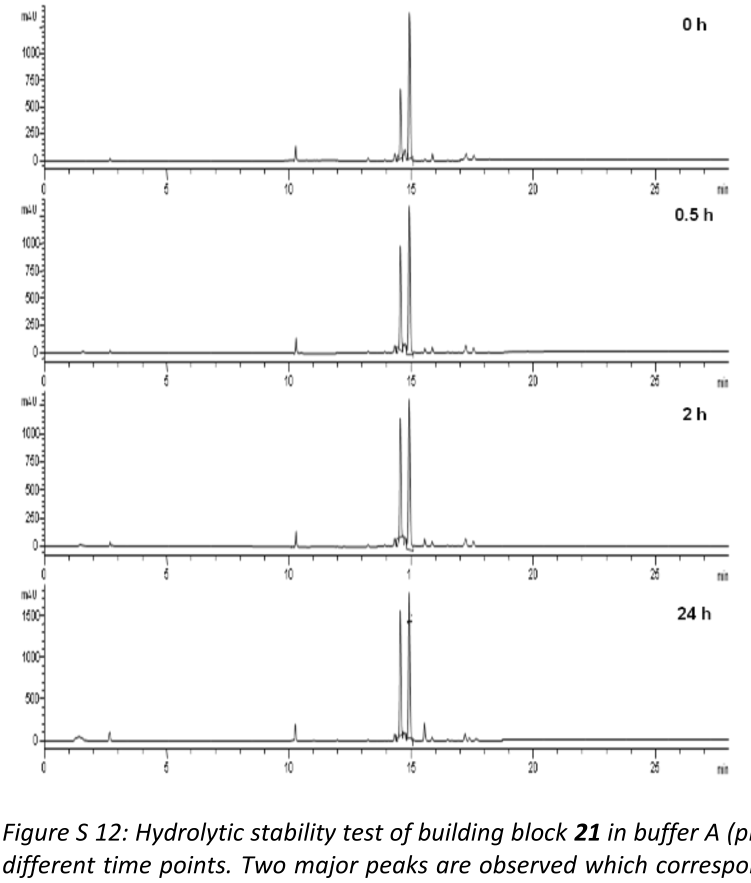

*Figure S 12: Hydrolytic stability test of building block **21** in buffer A (pH 6.0). HPLC (214 nm) traces are shown at different time points. Two major peaks are observed which correspond to the two diastereomers of the intact building block **21**.*

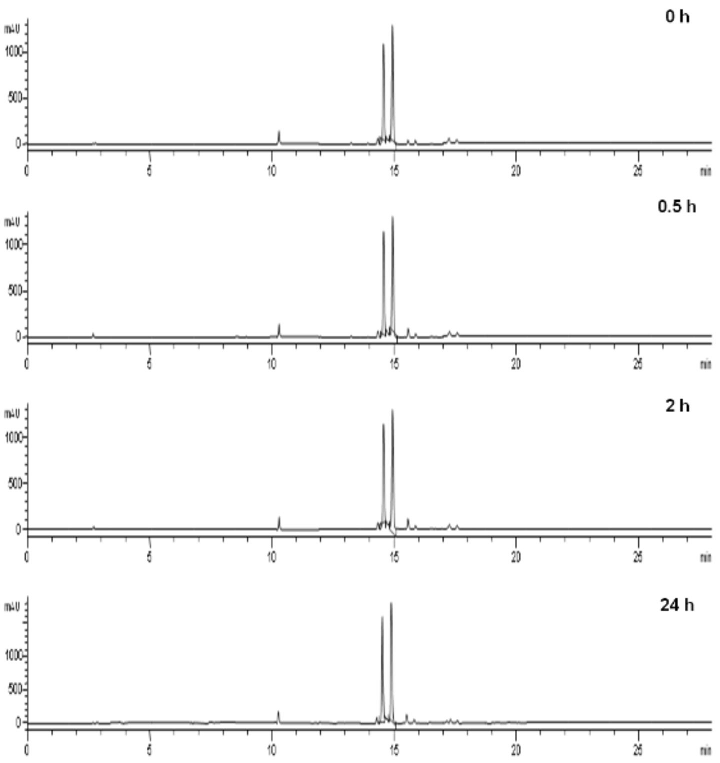

*Figure S 13: Hydrolytic stability of 5HP2O building block **21** in buffer B (pH 7.0). HPLC (214 nm) traces are shown at different time points. Two major peaks are observed which correspond to the two diastereomers of the intact building block **21**.*

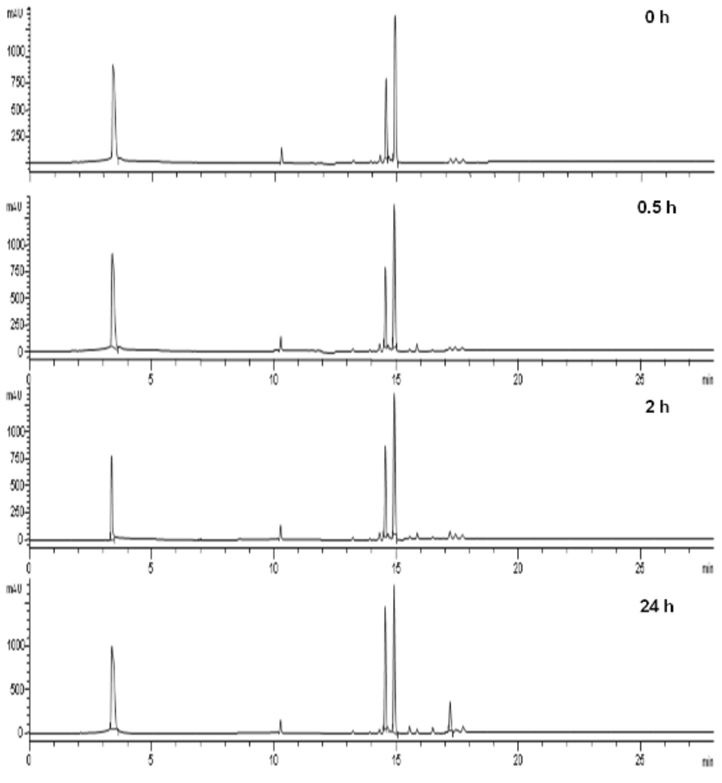

*Figure S 14: Hydrolytic stability of 5HP2O building block **21** in buffer C (pH 8.0). HPLC (214 nm) traces are shown at different time points. Two major peaks are observed which correspond to the two diastereomers of the intact building block **21**.*

5.2. Glutathione competition experiment

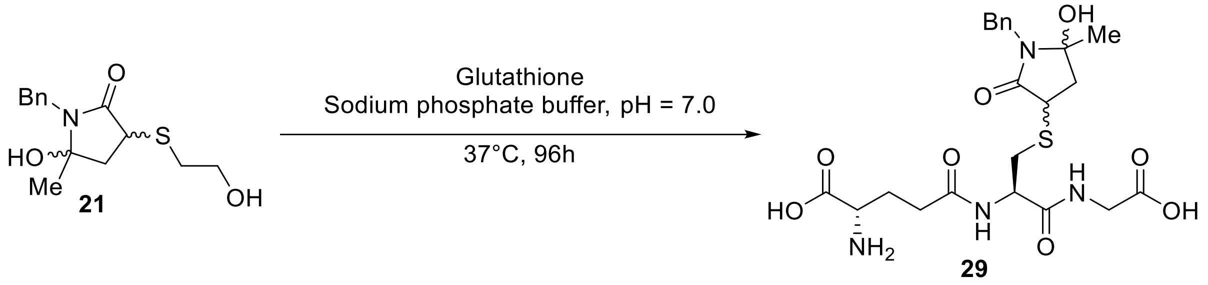

*Scheme S 7: Thiol exchange experiment between compound **18** and glutathione.*

Compound **21** was dissolved in a 0.1 M sodium phosphate buffer at pH 7 containing 10 equiv of glutathione. The mixture was incubated at 37 °C and shaken for 96 hours. The stability was checked using RP-HPLC. Analysis was performed on Phenomenex Luna C18 column with a flow rate of 1 mL/min. The following gradient was employed: 3 min 100% H_2O followed by 0 to 100% MeCN in 20 min and 5 min at 100% MeCN.

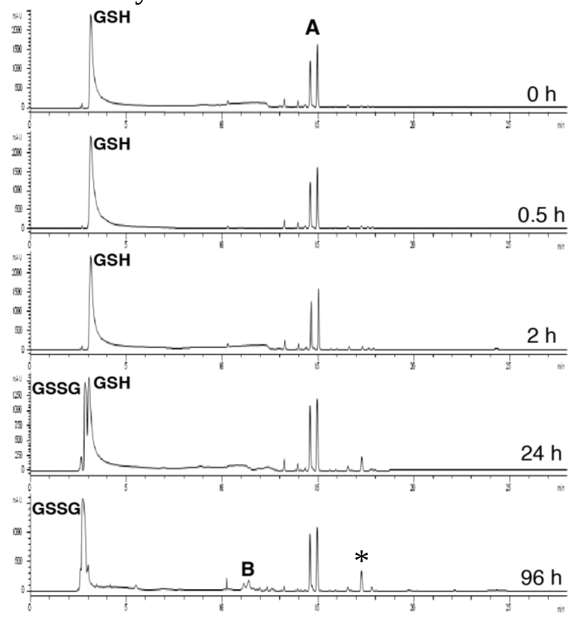

*Figure S 15: HPLC follow up of the thiol exchange experiment between compound **21** and glutathione at different time points. Two peaks (A) are observed for the starting compound **21** due to the presence of two diastereoisomers. Trace amounts of exchanged product **29** are observed (peak B) after 4 days together with a considerable amount of glutathione dimer (GSSG).*

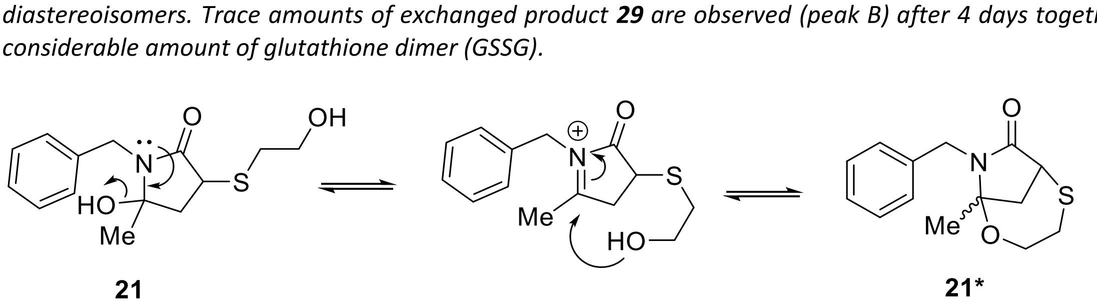

*Scheme S 8: Mechanistic proposal for the formation of side product **21***.*

Side product **21*** is formed during GSH exchange experiment (peak at t = 17.5 min, indicated with *) and can be formed following the suggested mechanism above.

6. Reactivity of 5HP2O building blocks towards peptide-like compounds

6.1. N-Acetyl-L-cysteine (NAC) conjugation

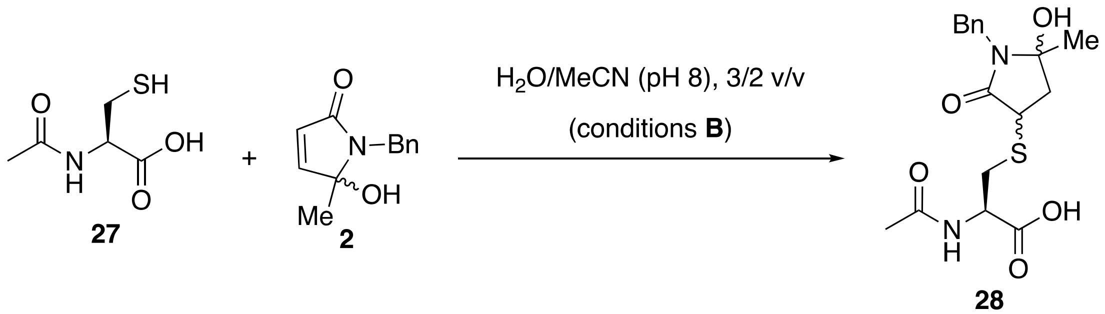

*Scheme S 9: Conjugation of N-acetyl-L-Cysteine to 5HP2O building block **2**.*

The purified lactam (0.1 mmol, 20 mg, **2**) was dissolved in H_2O/MeCN (3/2 v/v, 15 mL) mixture and *N*-acetyl-*L*-Cysteine (0.11 mmol, 18 mg, **27**) was added. The mixture was adjusted to pH 8.0 (with 0.1 M NaOH) and left to react for 3 hours at room temperature. The progress of the reaction was monitored by RP-HPLC. The formation of the desired conjugate was confirmed by LC-MS analysis which is shown below. Analysis was performed on Phenomenex Luna C18 column with a flow rate of 1 mL/min. The following gradient was employed: 3 min 100% H_2O followed by 0 to 100% MeCN in 20 min and 5 minutes at 100% MeCN.

Yield 64% (23.6 mg).The mixture of four isomers was analysed by NMR: ^{1}H NMR (400 MHz, DMSO-d_6): δ = 7.73 (m, 1H), 7.27 (m, 5H), 4.48 (m, 1H) 4.20 (m, 1H), 4.14 (m, 1H), 3.77 (m, 1H), 3.03 (m, 2H), 2.44 (m, 1H), 1.91 (m, 1H), 1.84 (s, 3H), 1.22 plus 1.19 (s, 3H) ppm; ^{13}C NMR (101 MHz, DMSO-d6): δ = 173.2, 172.3, 168.6, 138.8, 128.2 (2C), 127 (2C), 126.6, 87.2, 53.5, 42.9, 41.8, 41.6, 33.8, 27.3, 22.7 ppm.

HRMS (TOF ESI): calculated for $C_{17}H_{22}N_2O_5S$: 367.1249 $[M+H]^+$, mass found: 367.1312 $[M+H]^+$.

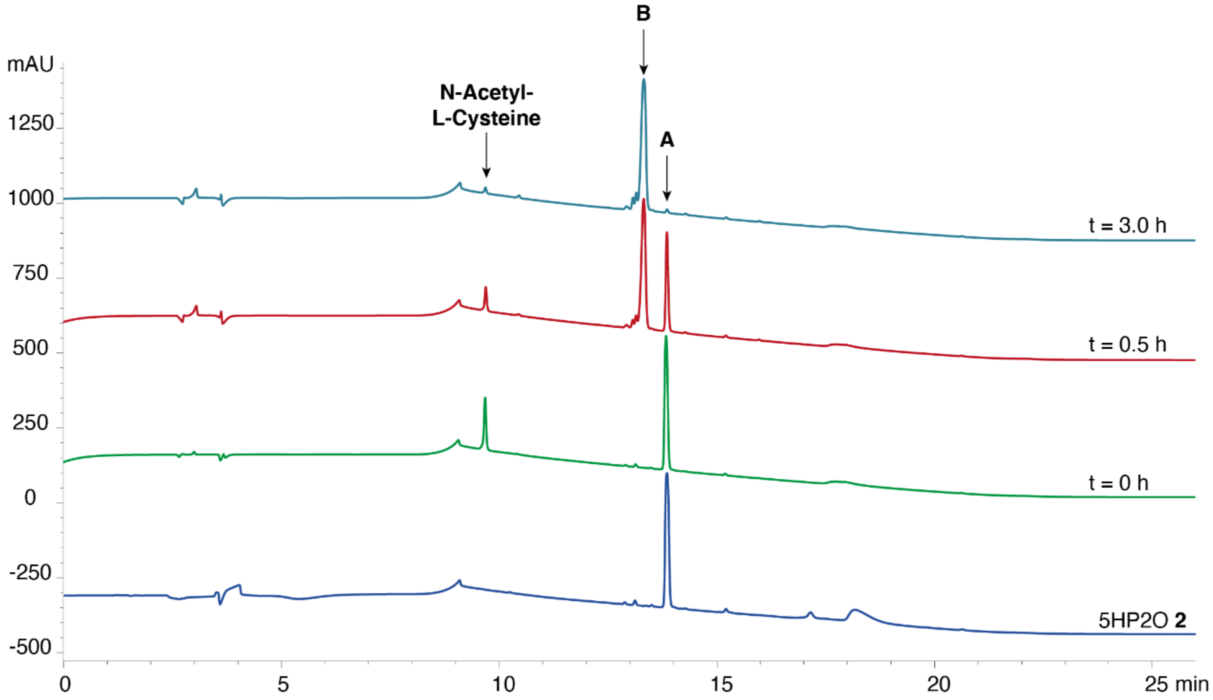

Figure S 16: Follow up of N-acetyl-L-cysteine conjugation to building block ***2*** *using RP-HPLC. LC traces (214 nm) are shown at different time points. (****A****) Corresponds to starting building block* ***2.*** *(****B****) Corresponds to formed conjugate* ***28****.*

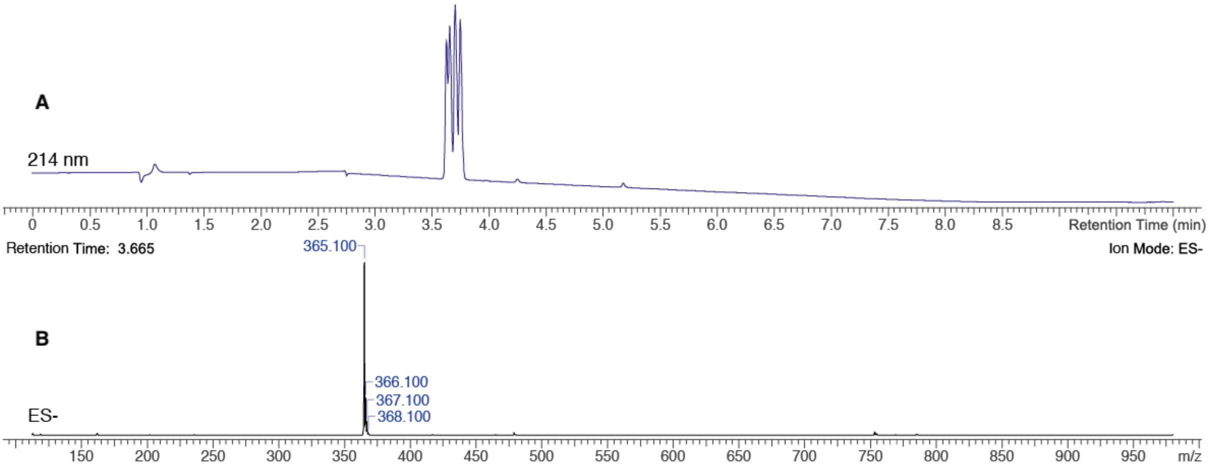

Figure S 17: LC-MS analysis of crude reaction mixture of N-Acetyl-L-Cysteine conjugation to building block ***2****. (A) Liquid chromatogram (214 nm) (B) Mass spectrum (ESI negative mode) corresponding to product* ***28****.*

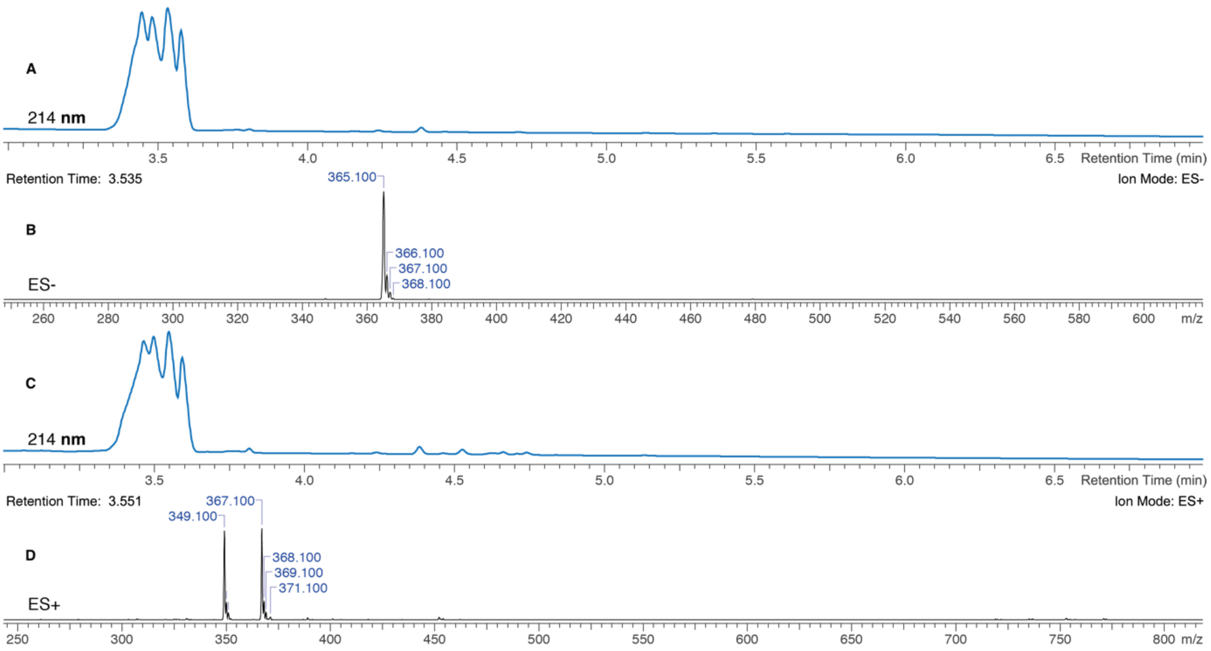

Figure S 18: Comparison between LC-MS measured in negative mode (A-B) and positive mode (C-D) of the same sample. (A) Liquid chromatogram in negative mode (B) Mass spectrum of product peak measured in negative mode (C) Liquid chromatogram in positive mode (D) Mass spectrum of product peak measured in positive mode: the -18 product is significantly present whereas this is not the case in negative mode (B).

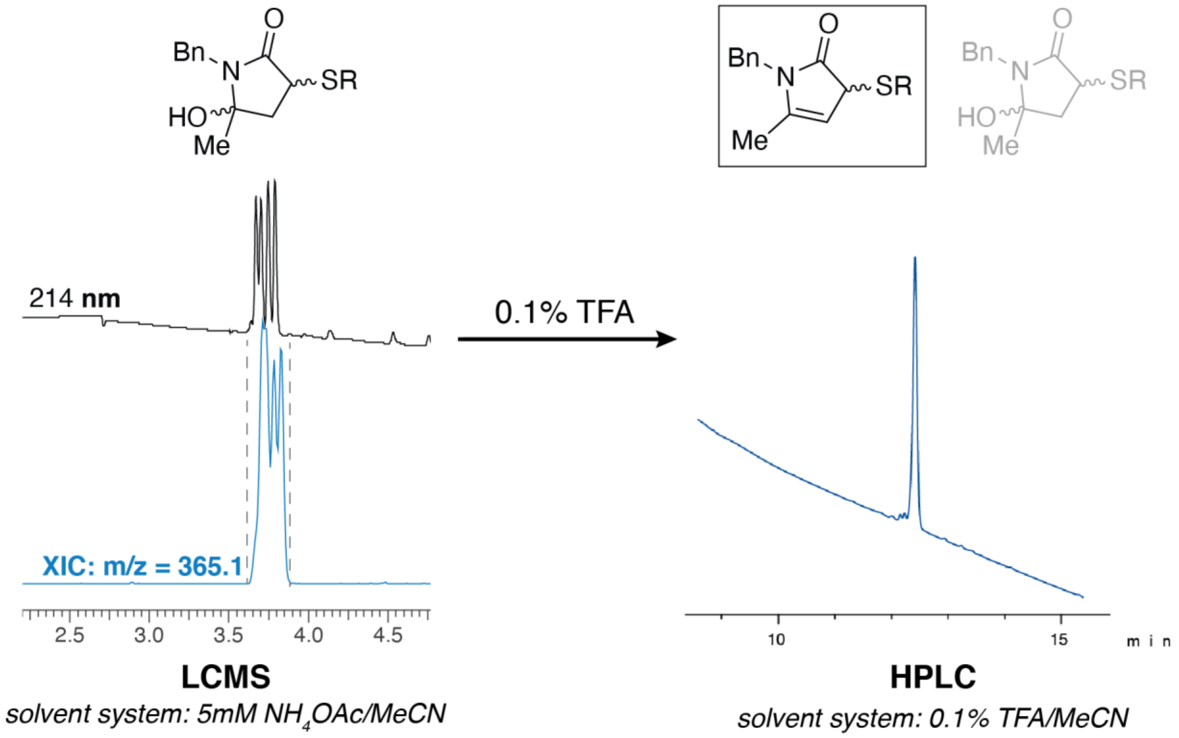

Figure S 19: Explanation of peak differences observed in LCMS and HPLC.

As can be seen in the figure above 4 peaks are observed in LCMS-analysis corresponding to the diastereomers of product **28** (R = NAC). Due to a different solvent system of our HPLC system, one of the stereocenters is eliminated (95%) resulting from the limited amount of product being injected in the system. Trace amounts of intact product **28** can be seen as shoulder peaks on the left of major peak at t = 12.5 min.

6.1.1. Kinetic analysis

6.1.1.1. 1-benzyl-5-hydroxy-5-methyl-1,5-dihydro-2H-pyrrol-2-one (2)

N-acetyl-*L*-Cysteine (0.006 mmol, 1 mg, **27**) was dissolved and incubated with respectively 5 (0.03 mmol, 6.1 mg) and 20 (0.12 mmol, 25 mg) equiv of purified lactam (**2**) in a Borate/Citrate/Phosphate ternary buffered pH 8 system[2] with 35% MeCN (20 mL). The progress of the reaction was monitored by RP-HPLC.

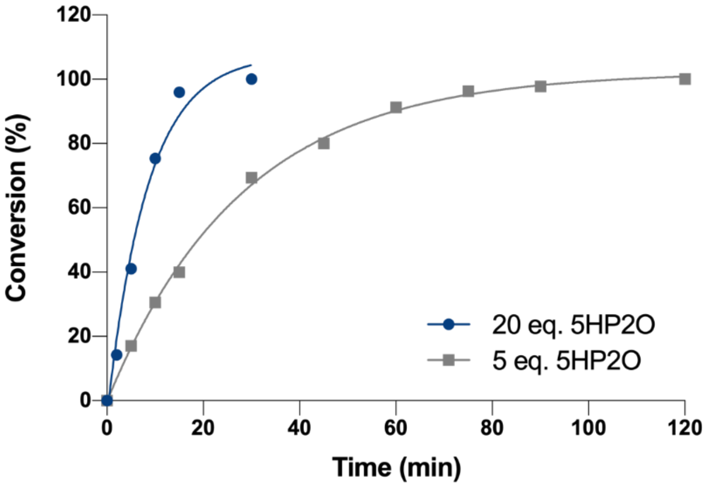

*Figure S 20: Conversion of NAC-5HP2O conjugate **28** is shown in function of the time for respectively 5 (grey) and 20 (blue) equiv of 5HP2O building block **2**.*

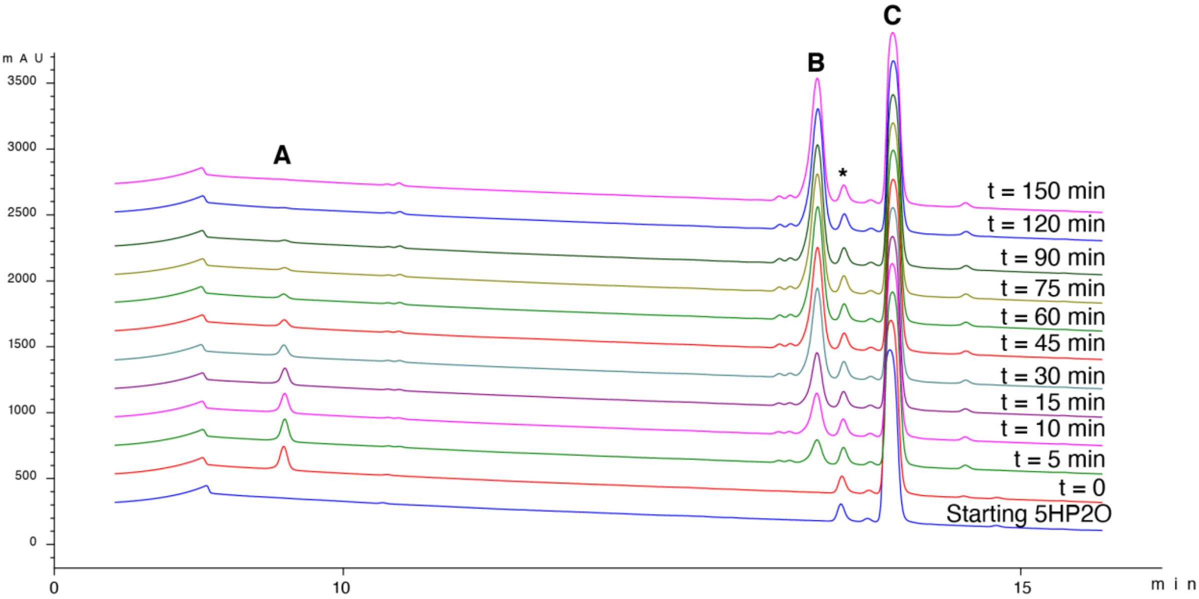

*Figure S 21: Follow up of N-acetyl-L-cysteine conjugation to 5 equiv of building block **2** using RP-HPLC. Zoomed regions of LC traces (214 nm) are shown at different time points. (**A**) Corresponds to starting building block **27.** (**B**) Corresponds to formed conjugate **28** (**C**) Corresponds to starting building block **2**. Small impurity in starting 5HP2O **2** is indicated with *.*

---

[2]Carmody, W. R. (1961), *Journal of Chemical Education*, 38, **11**, 559.

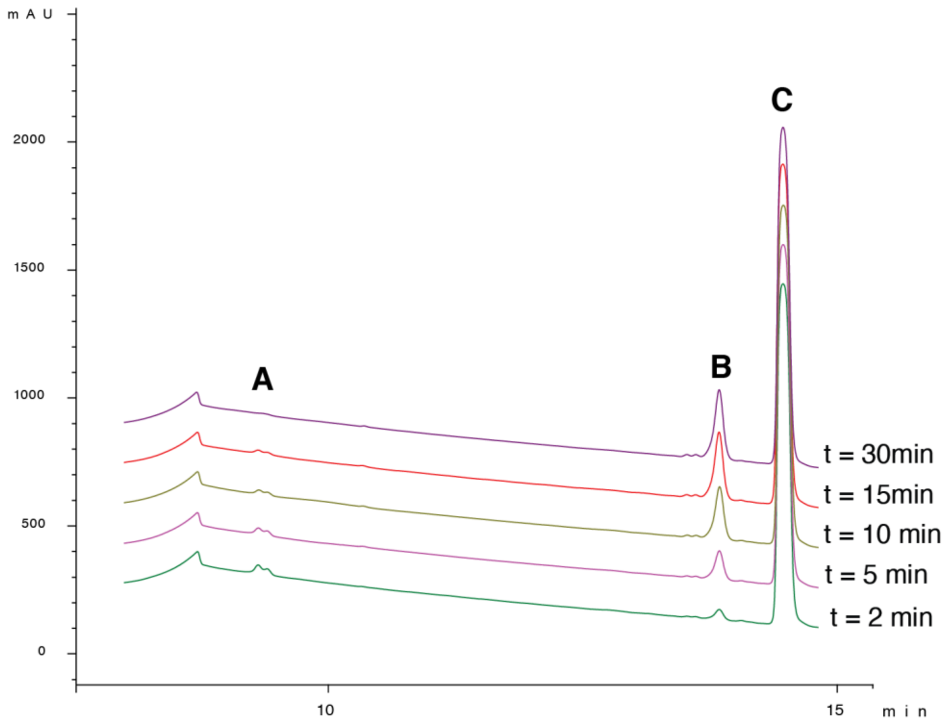

*Figure S 22: : Follow up of N-acetyl-L-cysteine conjugation to 5 equiv of building block **2** using RP-HPLC. Zoomed regions of LC traces (214 nm) are shown at different time points. (**A**) Corresponds to starting building block **27.** (**B**) Corresponds to formed conjugate **28** (**C**) Corresponds to starting building block **2**.*

6.1.1.2. Benzyl-maleimide

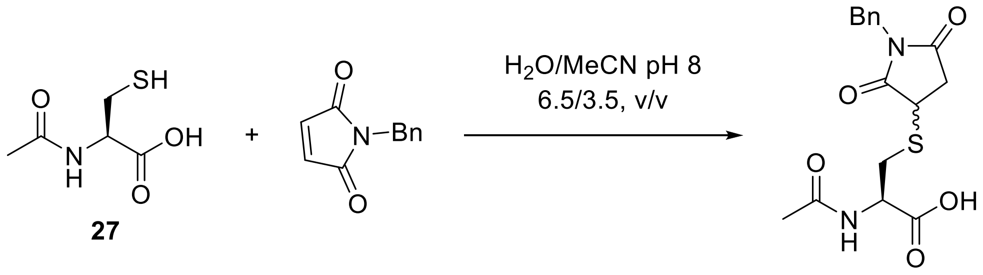

27

NAC-MAL

Scheme S 10: Conjugation of N-acetyl-L-Cysteine to Benzyl-maleimide.

N-acetyl-*L*-Cysteine (0.006 mmol, 1 mg, **27**) was dissolved and incubated with 5 equiv (0.03 mmol, 5.7 mg) of benzyl-maleimide in a Borate/Citrate/Phosphate ternary buffered pH 8 system[3] with 35% MeCN (20 mL). The progress of the reaction was monitored by RP-HPLC. Fast kinetics that are characteristic for maleimides were confirmed by immediate formation of the conjugate (**NAC-MAL**) upon addition of the maleimide.

[3]Carmody, W. R. (1961), *Journal of Chemical Education*, 38, **11**, 559.

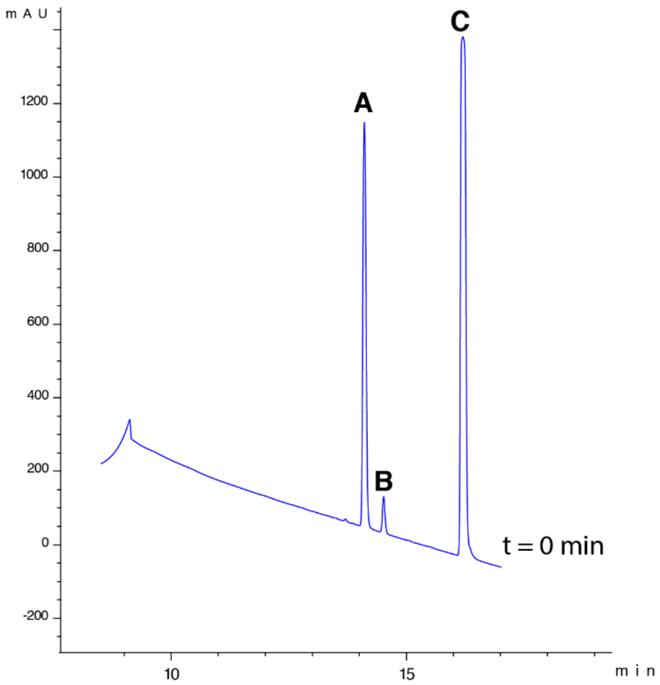

*Figure S 23: Zoomed region of LC trace (214 nm) is shown at the moment of addition of maleimide (t_0, t = 0 min). Full conversion is seen in less than a minute. (**A**) Corresponds to NAC-Maleimide conjugate. (**B**) Corresponds to ring-opened hydrolyzed benzyl-maleimide. (**C**) Corresponds to starting benzyl-maleimide.*

6.2. Stability of the NAC conjugates

6.2.1. Hydrolytic stability

6.2.1.1.5HP2O-conjugate

NAC-5HP2O conjugate **28** was incubated in a Borate/Citrate/Phosphate ternary buffered system[4] at pH 7, 8 and 9 at a concentration of 0.4 mM. The stability of the NAC-conjugate was checked by RP-HPLC after 24 hours of incubation.

[4]Carmody, W. R. (1961), *Journal of Chemical Education*, 38, **11**, 559.

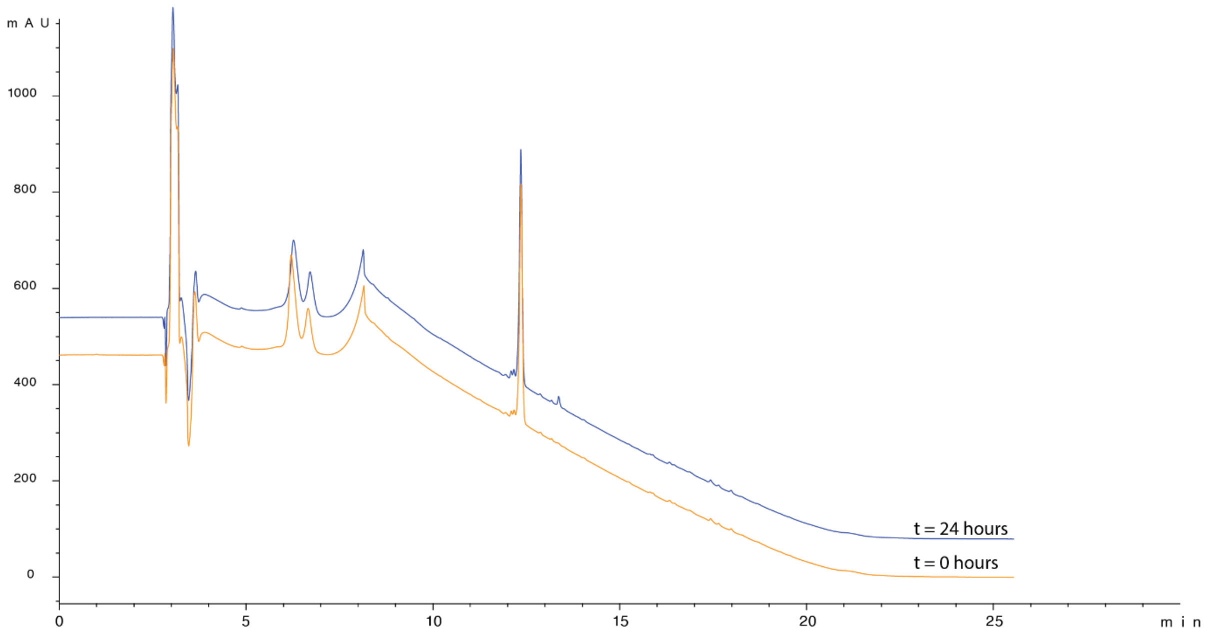

*Figure S 24: RP-HPLC analysis of NAC-5HP2O conjugate **28** before (orange) and after 24 hours (blue) of incubation in pH 7 buffered media.*

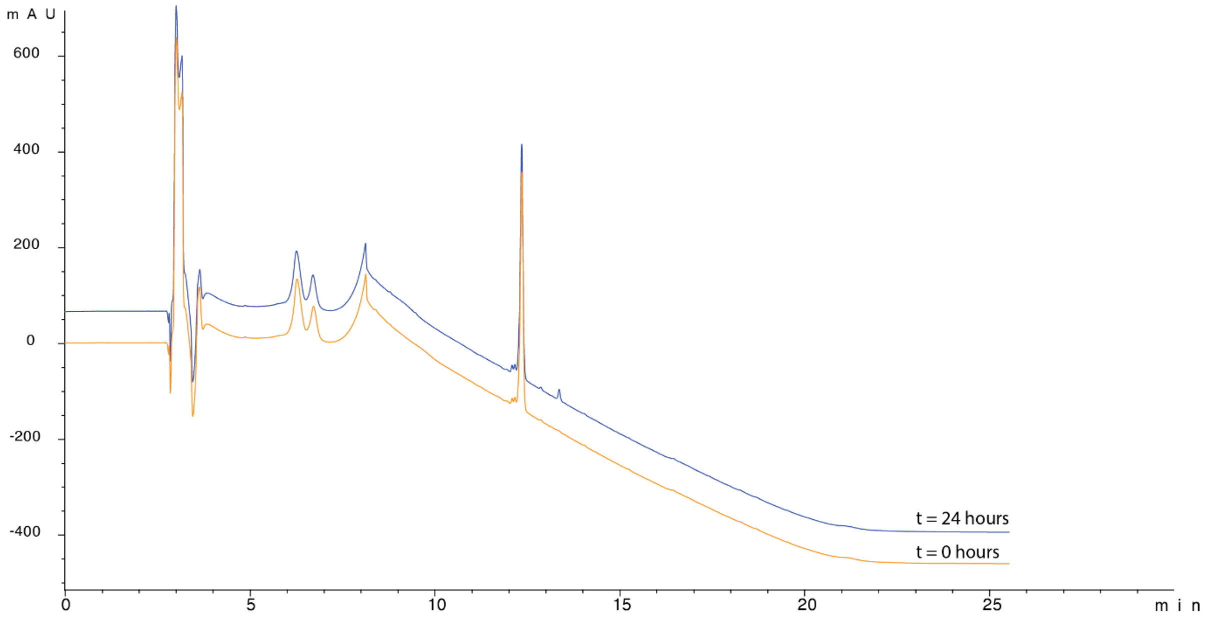

*Figure S 25: RP-HPLC analysis of NAC-5HP2O conjugate **28** before (orange) and after 24 hours (blue) of incubation in pH 8 buffered media.*

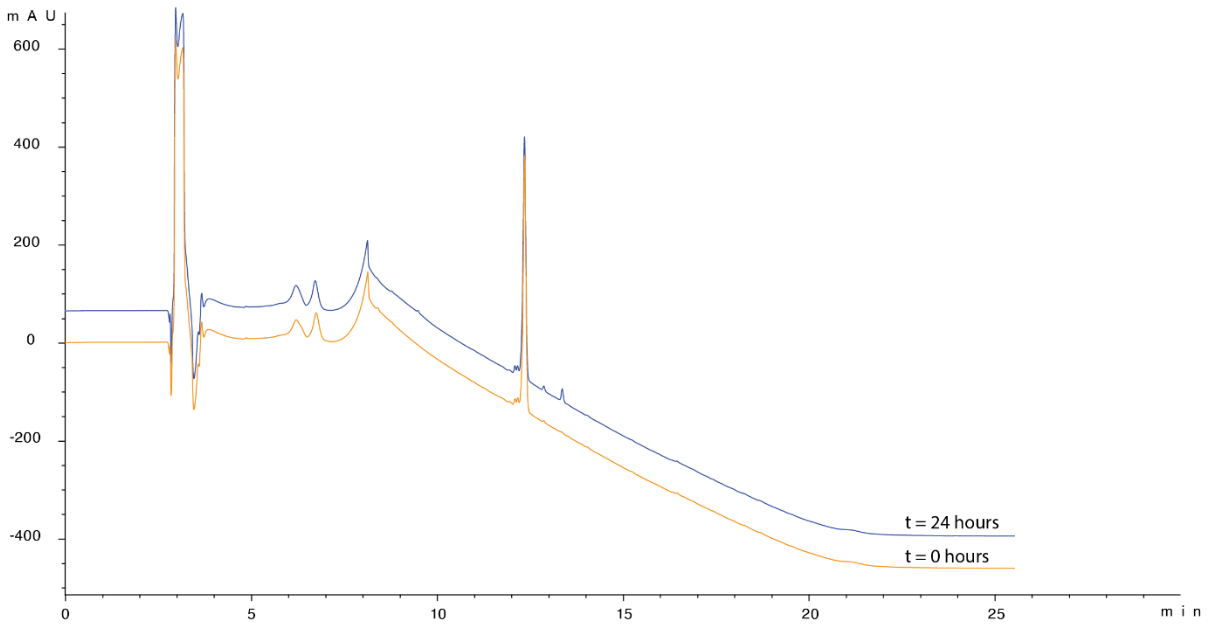

*Figure S 26: RP-HPLC analysis of NAC-5HP2O conjugate **28** before (orange) and after 24 hours (blue) of incubation in pH 9 buffered media*

6.2.1.2. Maleimide-conjugate

Benzyl-maleimide-NAC conjugate (**NAC-MAL**) was incubated in a Borate/Citrate/Phosphate ternary buffered system[5] at pH 7, 8 and 9 at a concentration of 0.4 mM. The stability of the NAC-conjugate was checked by RP-HPLC after 24 hours of incubation.

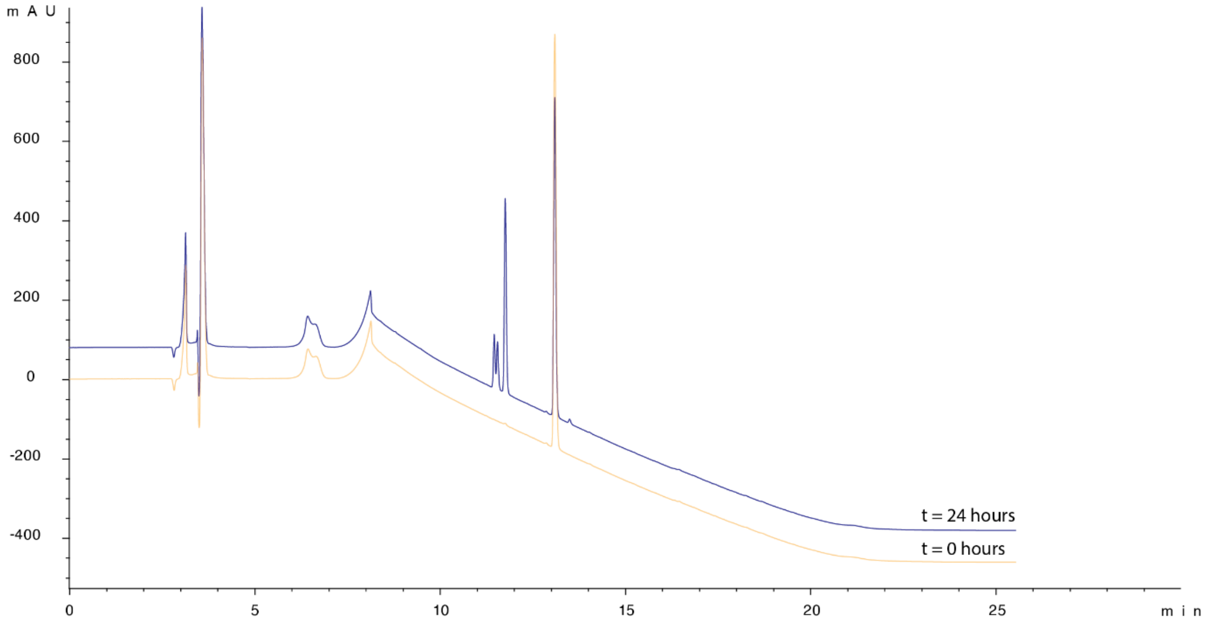

Figure S 27: RP-HPLC analysis of NAC-MAL conjugate before (light-orange) and after 24 hours (blue) of incubation in pH 7 buffered media.

[5] Carmody, W. R. (1961), *Journal of Chemical Education*, 38, **11**, 559.

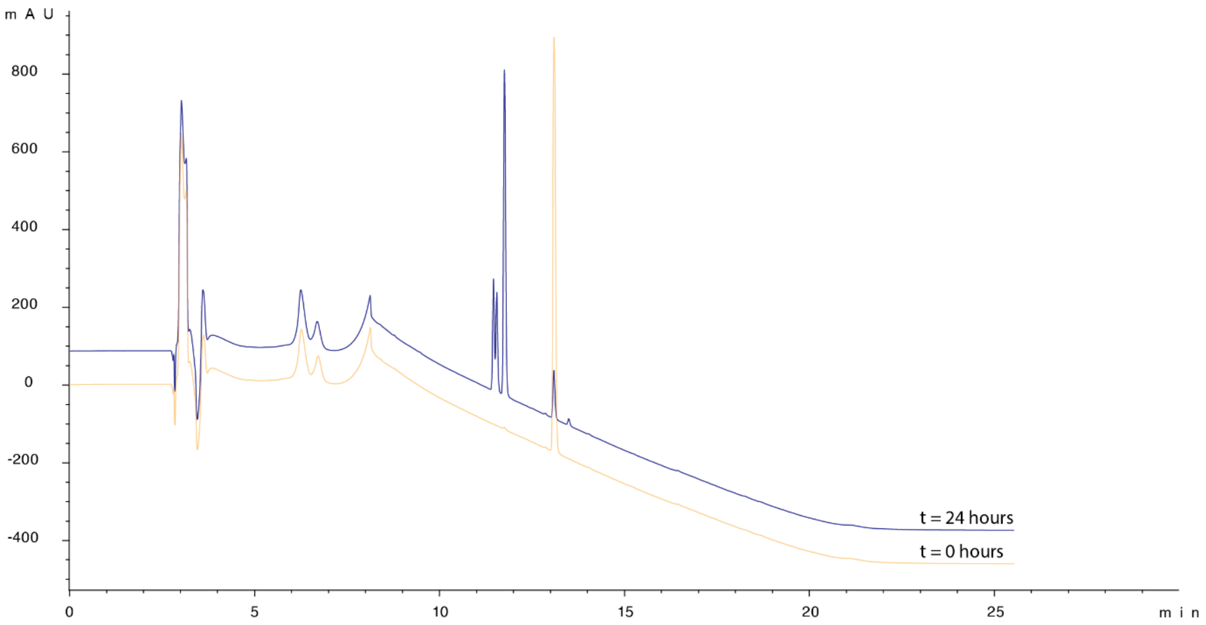

Figure S 28: RP-HPLC analysis of NAC-MAL conjugate before (light-orange) and after 24 hours (blue) of incubation in pH 8 buffered media.

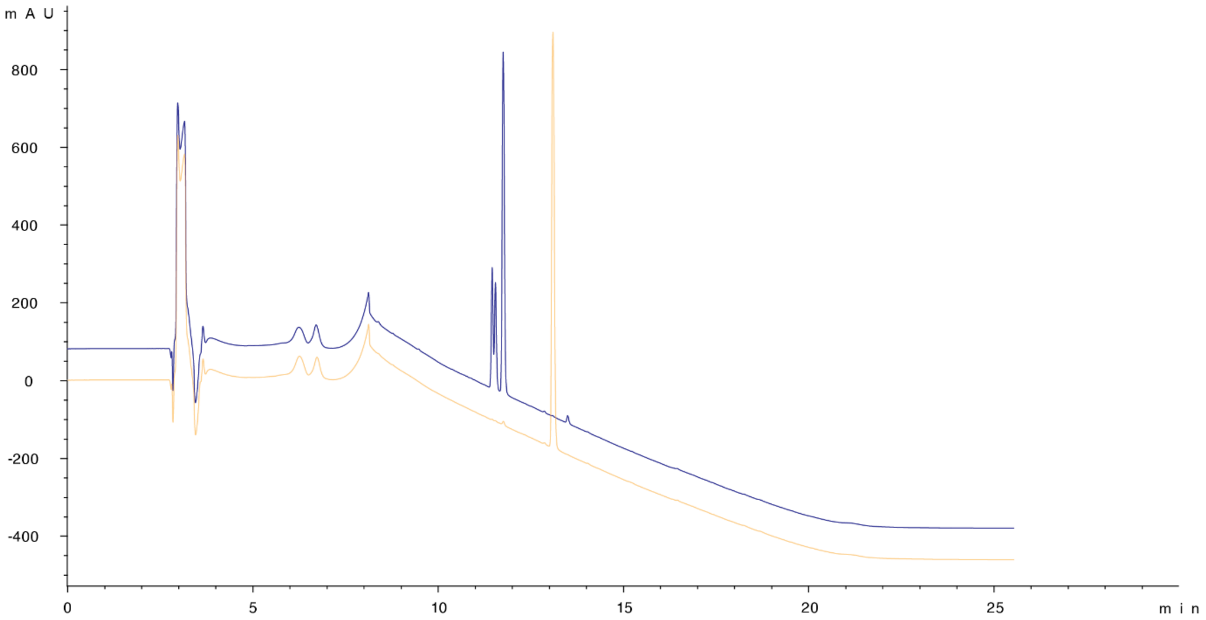

Figure S 29: RP-HPLC analysis of NAC-MAL conjugate before (light-orange) and after 24 hours (blue) of incubation in pH 9 buffered media.

6.2.2. Glutathione competition experiment

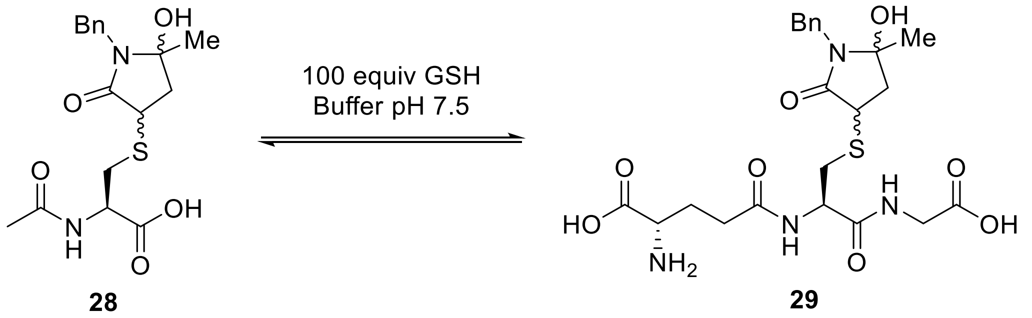

Scheme S 11: Thiol exchange experiment between compound ***28*** *and glutathione.*

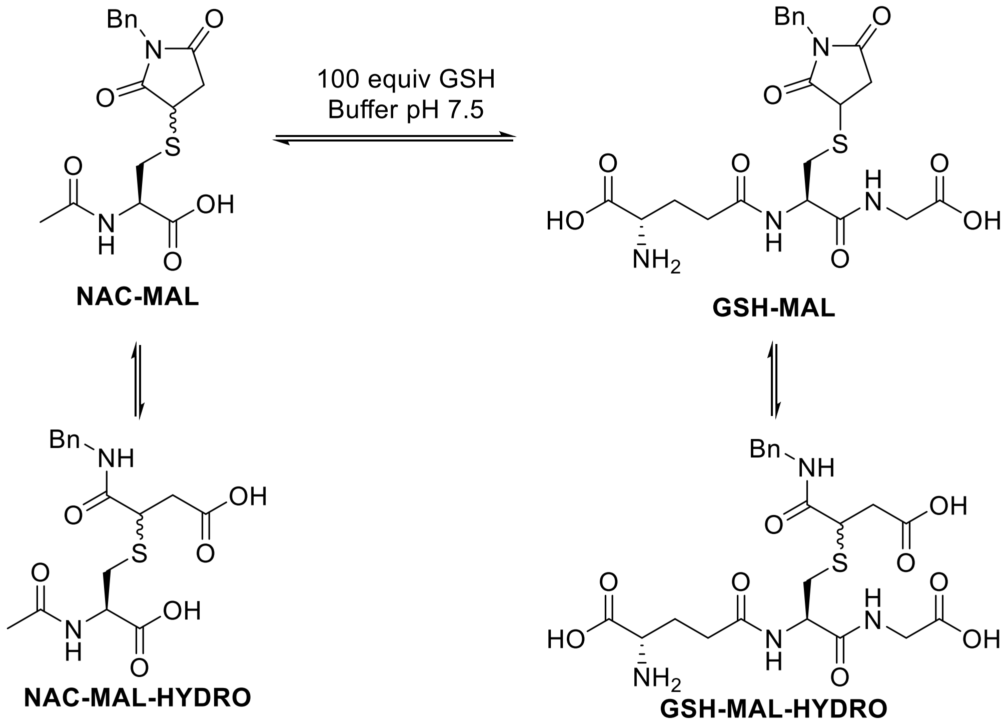

Scheme S 12: Thiol exchange experiment between compound ***NAC-MAL*** *and glutathione and their ring-opened hydrolyzed products.*

NAC-conjugates (**28** and **NAC-MAL**) were incubated in aqueous buffered media at a pH of 7.5 with 10 mM GSH at a concentration of 0.1 mM. Degradation of the NAC-conjugates was followed via HPLC and plotted as % of intact NAC-conjugate.

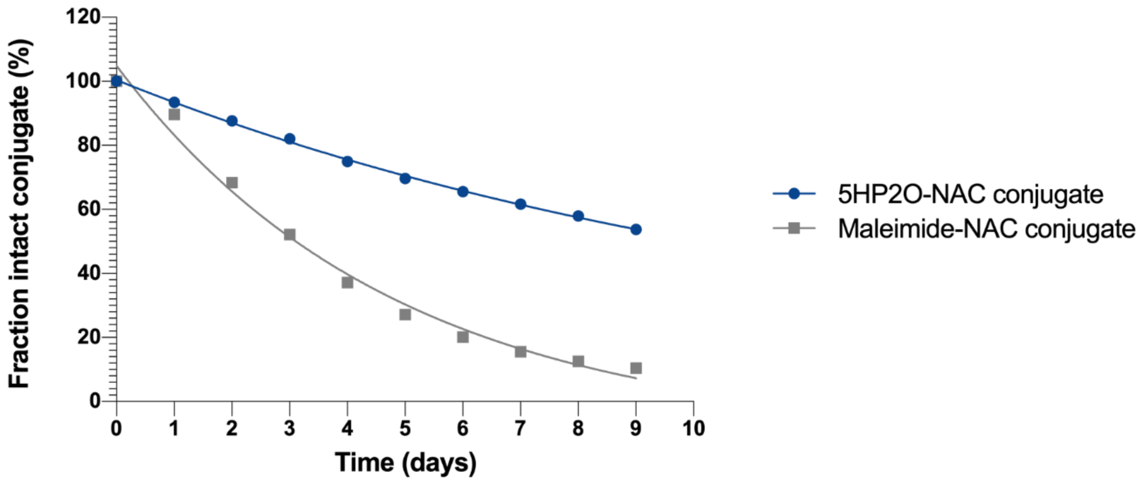

Figure S 30: Graph showing the percentage of intact NAC-conjugate in function of time. Intact 5HP2O conjugate is shown in blue and intact Maleimide conjugate in grey.

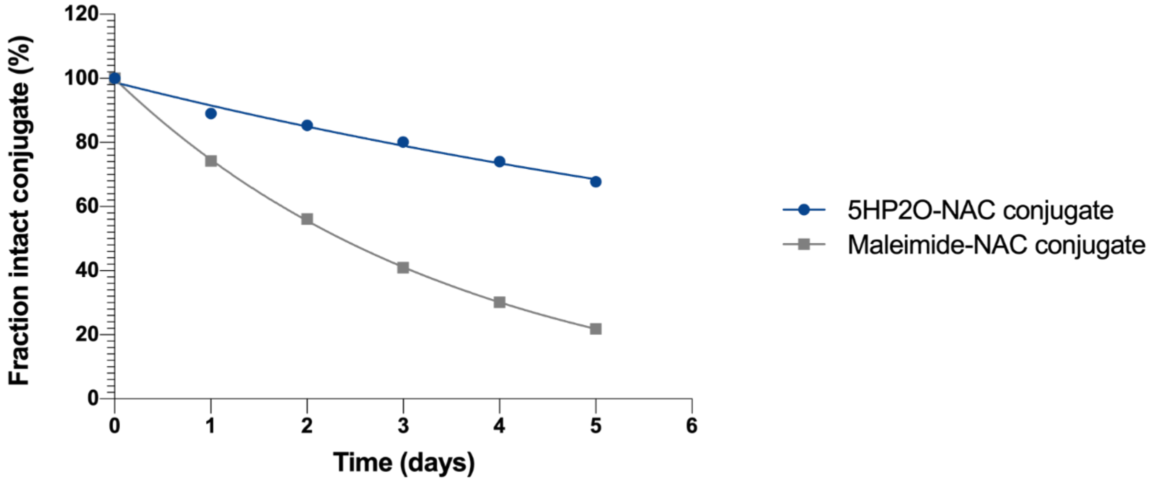

Figure S 31: Graph showing the percentage of intact NAC-conjugate in function of time. Intact 5HP2O conjugate is shown in blue and intact Maleimide conjugate in grey.

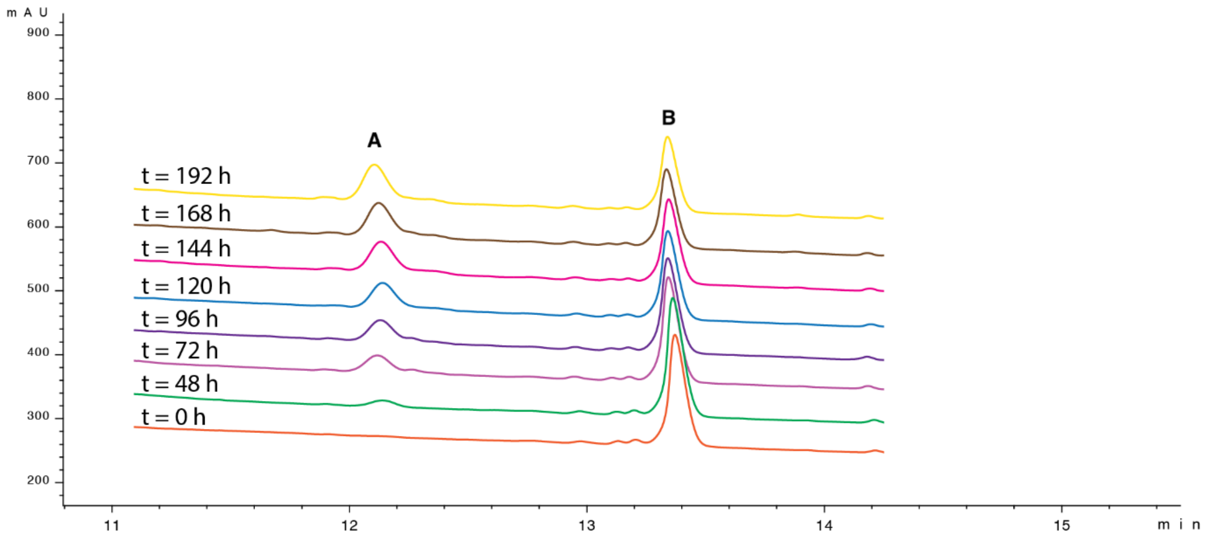

*Figure S 32: Follow up of degradation of 5HP2O-NAC conjugate **28** in function of time. Zoomed regions of LC traces (214 nm) are shown at different time points. (**A**) Corresponds to exchanged GSH product. (**B**) Corresponds to intact 5HP2O-NAC conjugate **28**.*

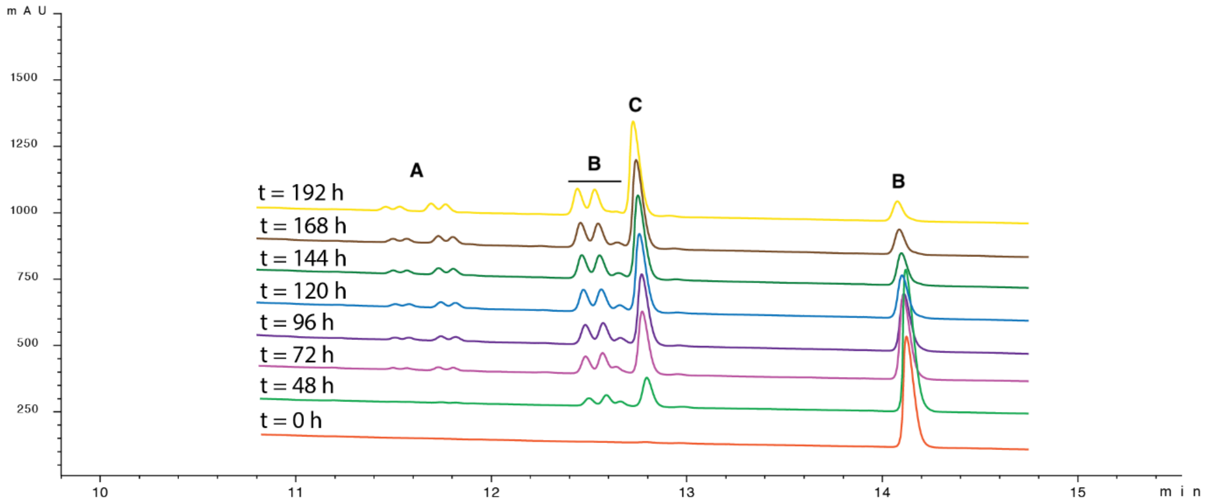

*Figure S 33: Follow up of degradation of Maleimide-NAC conjugate **NAC-MAL** in function of time. Zoomed regions of LC traces (214 nm) are shown at different time points. (**A**) Corresponds to hydrolyzed exchanged GSH product. (**B**) Corresponds to hydrolyzed NAC-MAL and GSH exchanged product. (**C**) Corresponds to hydrolyzed **NAC-MAL.** (**D**) Corresponds to intact **NAC-MAL**.*

6.3. General procedure for the conjugation of α,β-unsaturated γ-lactam with GSH

Unsaturated lactams (**2**, **3**, **7, 15, 16**) were dissolved in aqueous solution (1.0 mL) followed by addition of GSH (1.3 equiv.). Reactions were incubated at room temperature and their progress was monitored by RP-HPLC. RP-HPLC analyses were performed as described previously using a Phenomenex Luna C18 column (250 x 4.6 mm, 5 µm particle size at 35 °C). A flow rate of 1 ml/min was used with the following solvent system: 0.1% TFA in H_2O (A) and MeCN (B). The column was flushed for 3 min with 100% A, then a gradient from 0 to 100% B over 15 min, followed by 5 min of flushing with 100% B.

6.3.1. Conjugation of lactam 2 with GSH

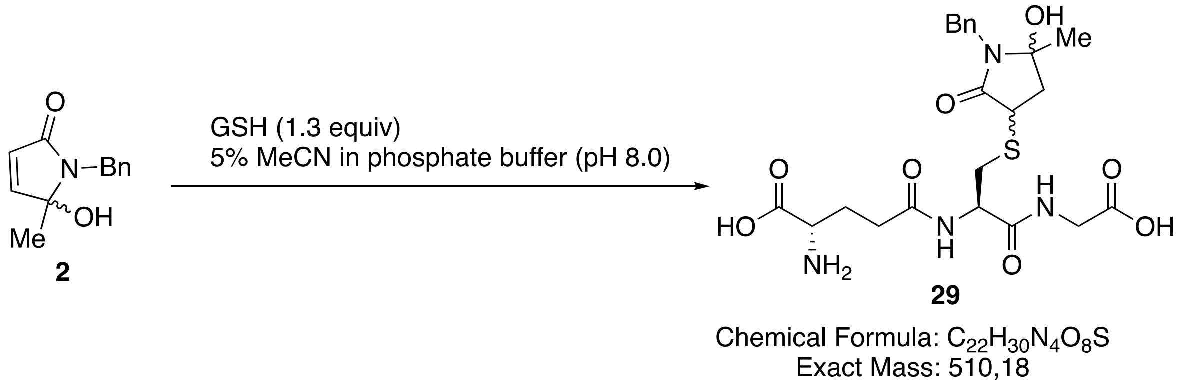

*Scheme S 13: Glutathione conjugation to 5HP2O building block **2**.*

The reaction was accomplished according to the general experimental procedure described above, utilizing the lactam **2** (0.010 µmol, 2 mg) and GSH (0.013 µmol, 4 mg) in 5% (vol/vol) CH_3CN/1 M phosphate buffer pH 8.0 (1.0 mL). Reaction progress was monitored by HPLC and formation of the desired conjugate was confirmed by LC-MS analysis which is shown below.

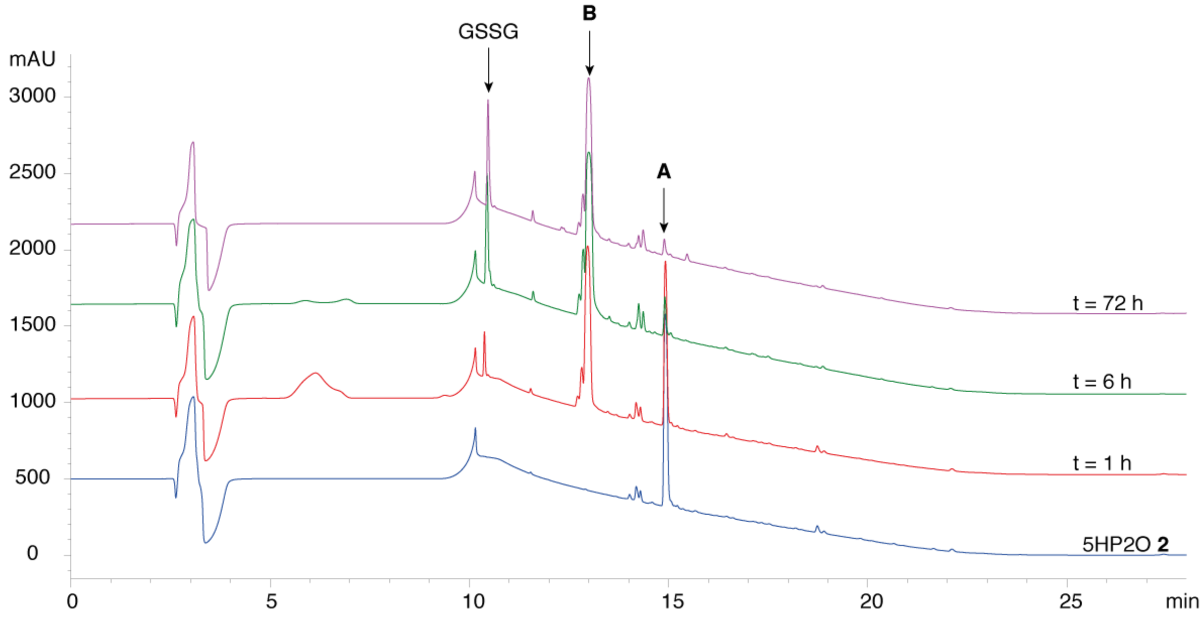

*Figure S 34: Follow up of glutathione addition to 5HP2O building block **2** using RP-HPLC. LC (214 nm) traces are shown at different time points. Peak **A** corresponds to starting material (**2**), peak **B** to product **29**. Glutathione dimer (GSSG) is formed over time.*

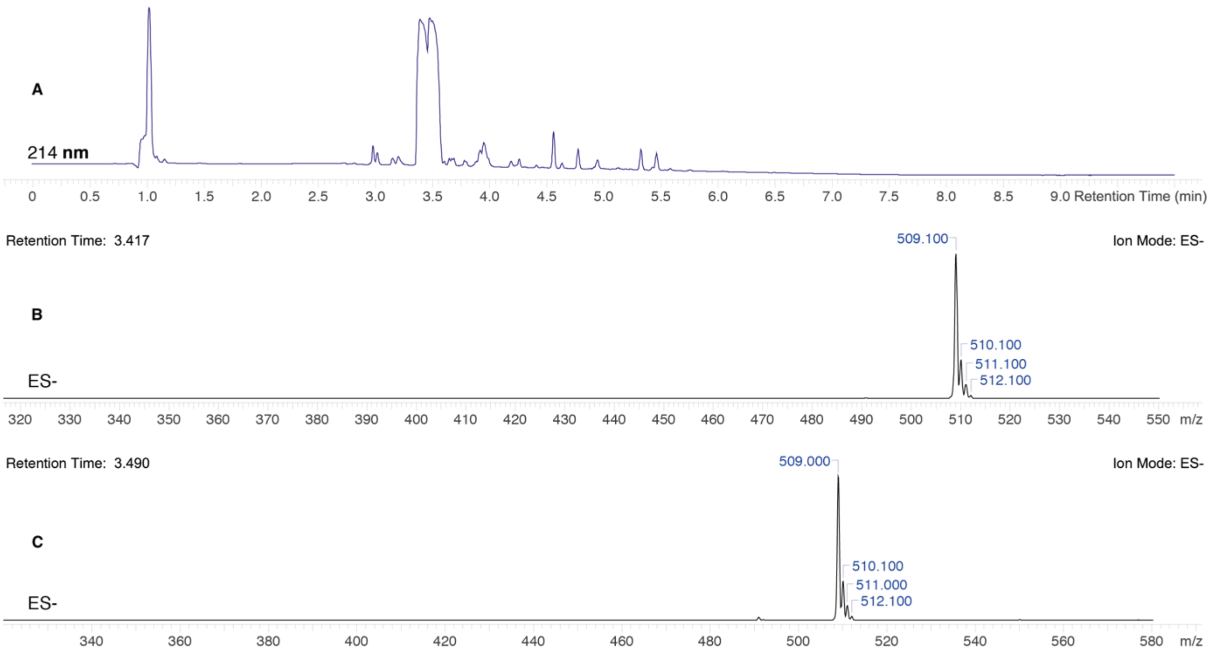

Figure S 35: LC-MS analysis of crude conjugation mixture. (A) Liquid chromatogram (214 nm), (B) Mass spectrum of product peak (C) mass spectrum of product peak. Two product peaks observed due to stereoisomers.

6.3.2. Conjugation of lactam 3 with GSH

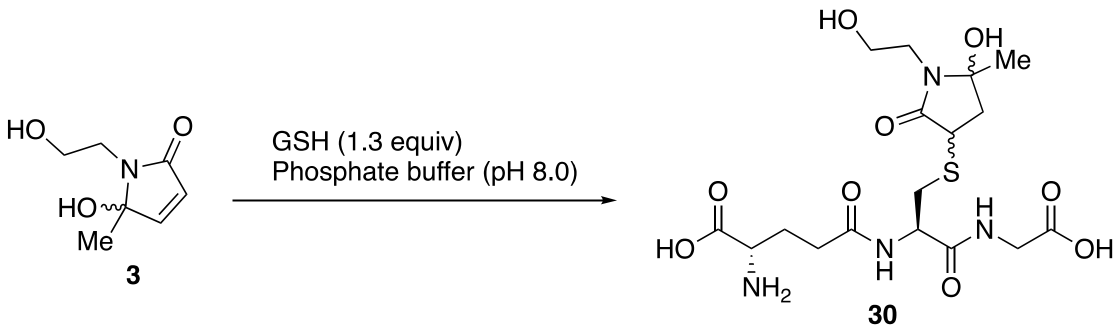

Chemical Formula: $C_{17}H_{28}N_4O_9S$
Exact Mass: 464,16

*Scheme S 14: Glutathione conjugation to 5HP2O building block **3**.*

The reaction was accomplished according to the general experimental procedure described above, utilizing the lactam **3** (0.013 µmol, 2 mg) and GSH (0.017 µmol, 5 mg) in 1 M phosphate buffer of pH 8.0 (1.0 mL). The formation of the desired conjugate was confirmed by LC-MS analysis which is shown below.

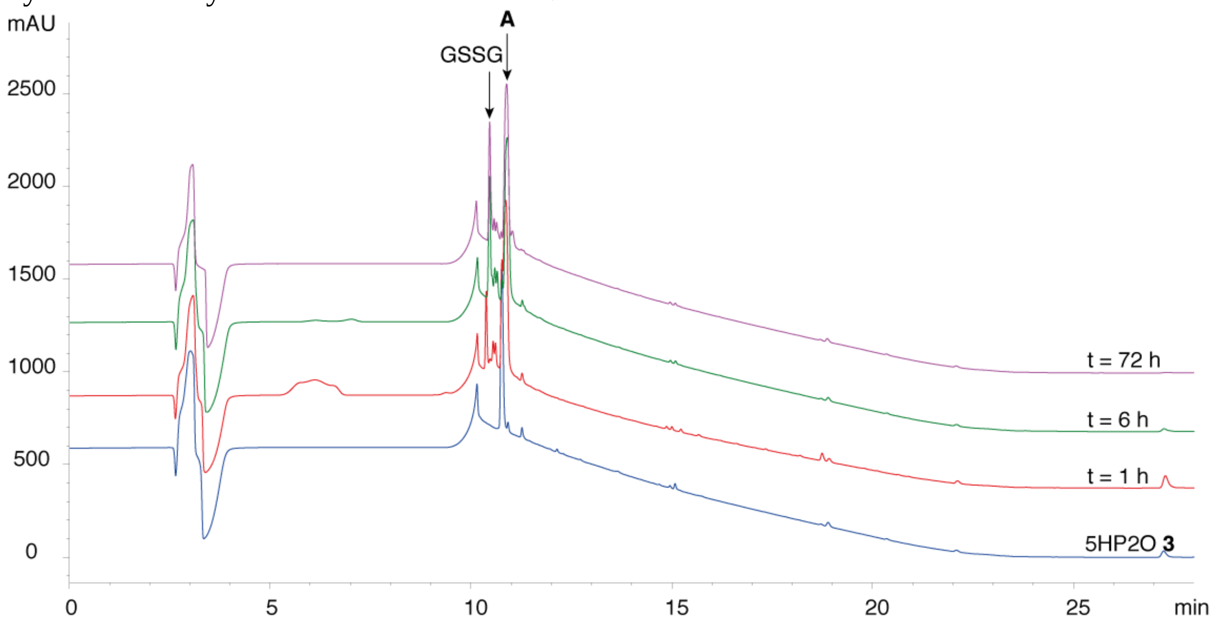

*Figure S 36: Follow up of glutathione addition to 5HP2O building block using RP-HPLC. LC (214 nm) traces are shown at different time points. Peak **A** corresponds to starting material (**3**) as well as to final conjugate product **30**. Glutathione dimer (GSSG) is formed over time.*

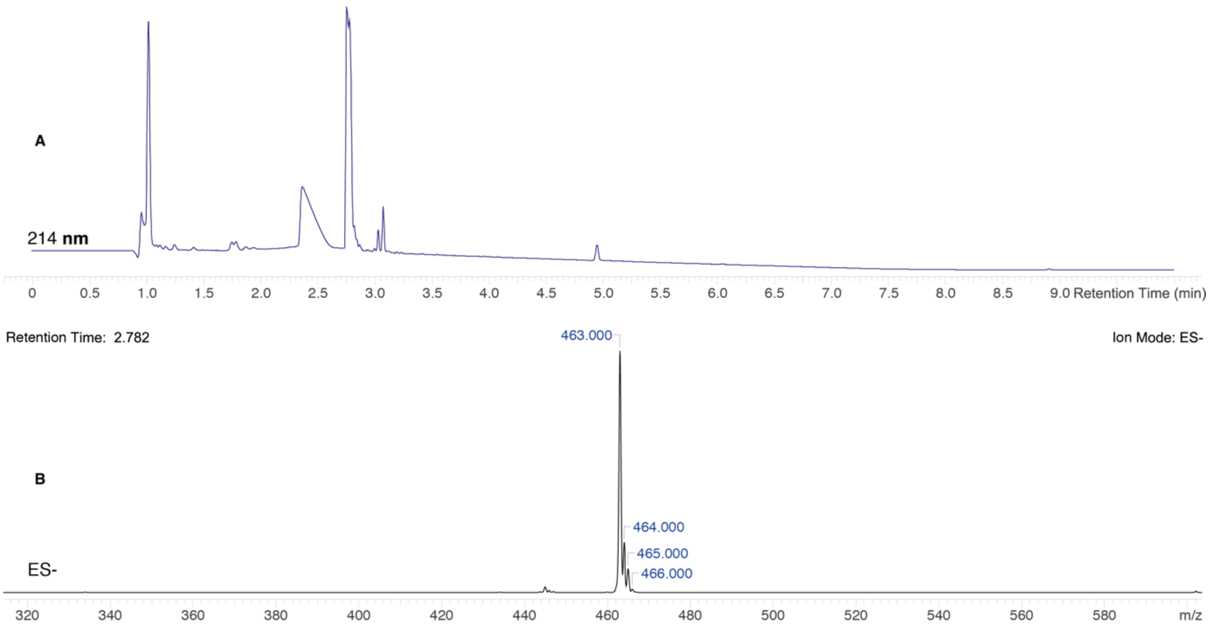

Figure S 37: LC-MS analysis of crude conjugation mixture. (A) Liquid chromatogram (214 nm), (B) Mass spectrum of product peak.

6.3.3. Conjugation of lactam 7 with GSH

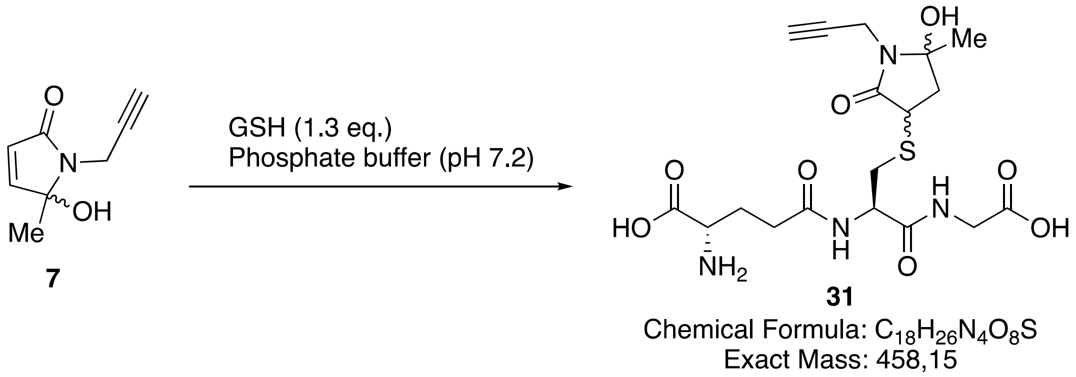

Scheme S 15: Glutathione conjugation to 5HP2O building block ***7****.*

The reaction was accomplished according to the general experimental procedure described above, utilizing the lactam **7** (0.013 µmol, 2 mg) and GSH (0.017 µmol, 5 mg) in 1 M phosphate buffer of pH 7.2 (1.0 mL). Reaction progress was monitored by HPLC and formation of the desired conjugate was confirmed by LC-MS analysis which is shown below.

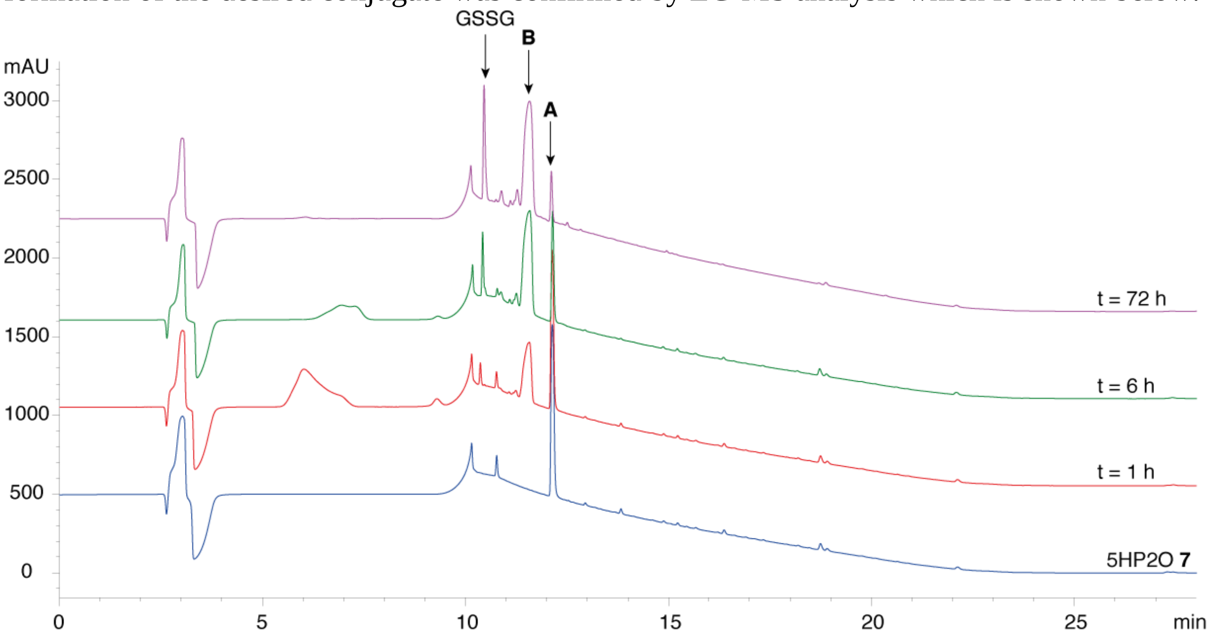

Figure S 38: Follow up of glutathione addition to 5HP2O building block using RP-HPLC. LC (214 nm) traces are shown at different time points. Peak ***A*** *corresponds to starting material (****7****), peak* ***B*** *to product* ***31****. Glutathione dimer (GSSG) is formed over time.*

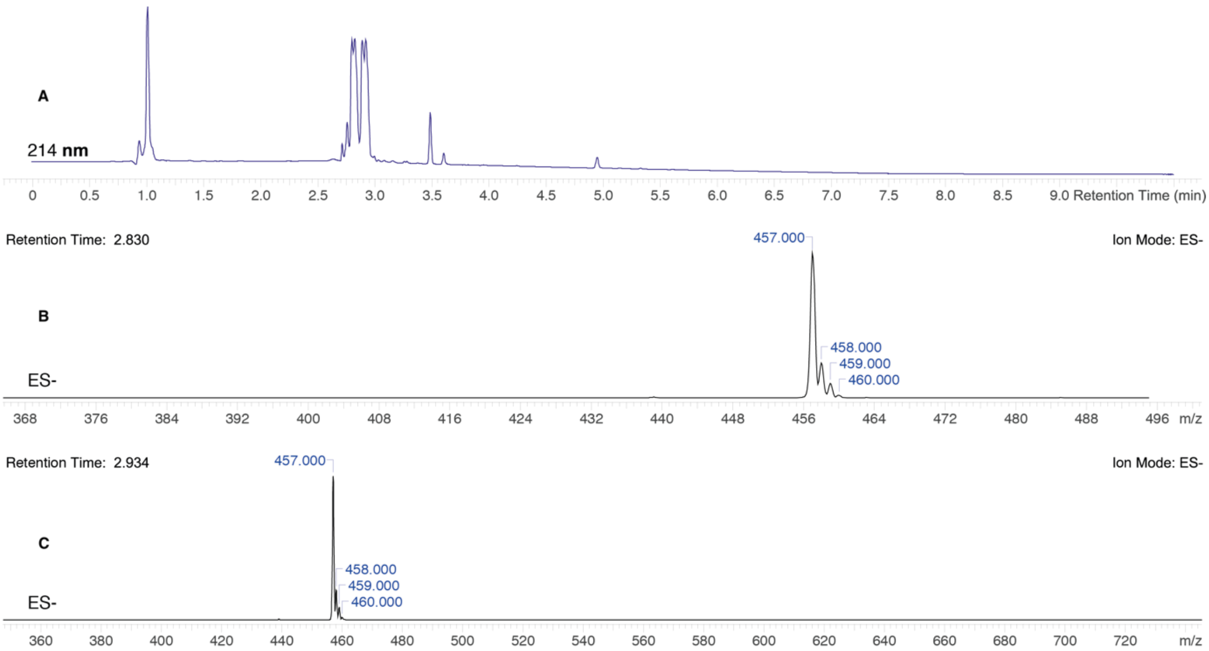

Figure S 39: LC-MS analysis of crude conjugation mixture. (A) Liquid chromatogram (214 nm), (B) Mass spectrum of product peak (C) mass spectrum of product peak. Four product peaks observed due to the formation of four different diastereoisomers.

6.3.4. Conjugation of lactam 15 with GSH

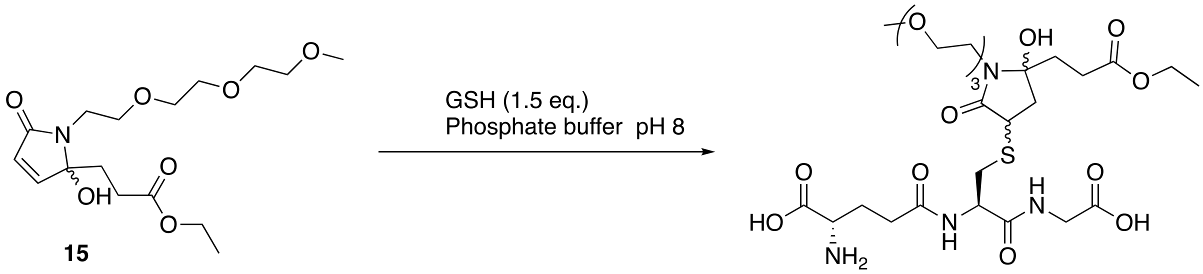

15

32

Chemical Formula: $C_{26}H_{44}N_4O_{13}S$
Exact Mass: 652,26

Scheme S 16: Glutathione conjugation to 5HP2O building block ***15****.*

The reaction was accomplished according to the general experimental procedure described above, utilizing the lactam **15** (0.007 mmol, 2 mg) and GSH (0.0105 mmol, 3.2 mg) in 1 M phosphate buffer of pH 8.0 (1.0 mL). The formation of the desired conjugate was confirmed by LC-MS analysis. Calculated mass for $C_{26}H_{43}N_4O_{13}S$: 651.3 $[M\text{-}H]^-$; mass found: 633.3 $[M\text{-}H\text{-}H_2O]^-$ and hydrolysed conjugates 623.1 $[M\text{-}H\text{-}EtOH\text{+}H_2O]^-$ and 605.1 $[M\text{-}H\text{-}EtOH\text{+}H_2O\text{-}H_2O]^-$.

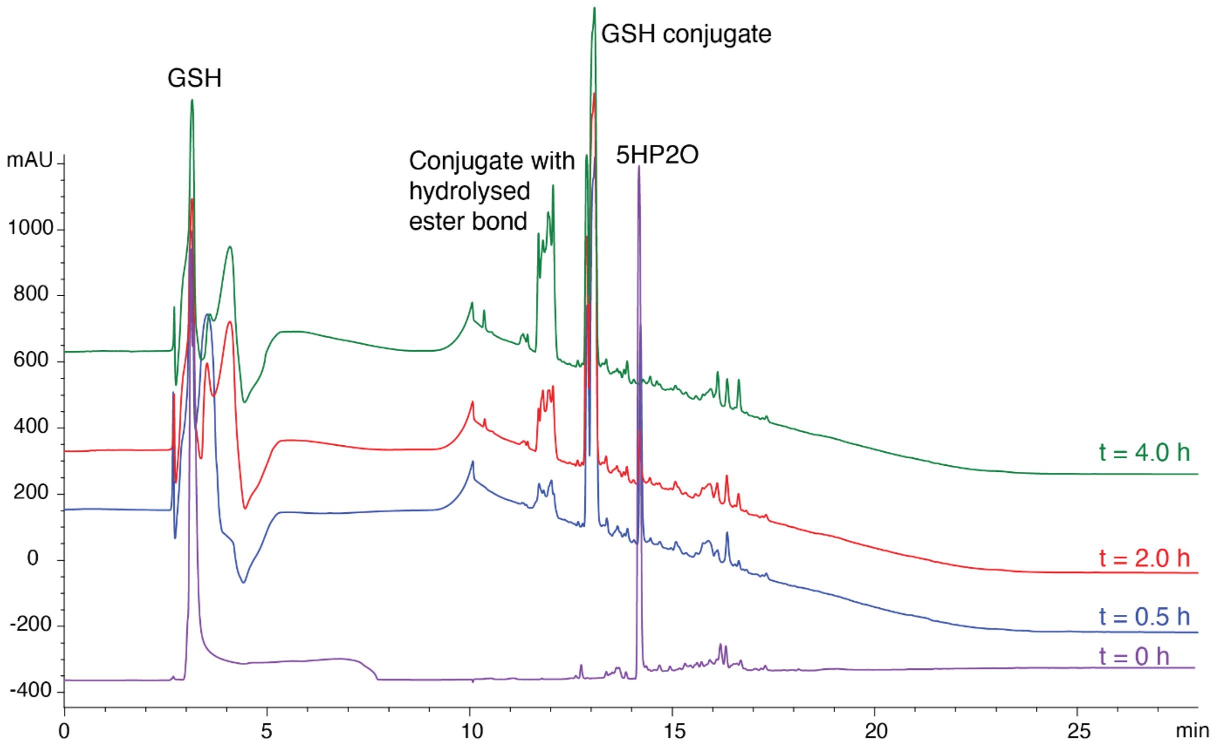

Figure S 40: Follow up of glutathione addition to 5HP2O building block using RP-HPLC. LC (214 nm) traces are shown at different time points. Due to basic conditions, hydrolysis of the ester bond was observed (Hydrolysed conjugate).

6.3.5. Conjugation of lactam 16 with GSH

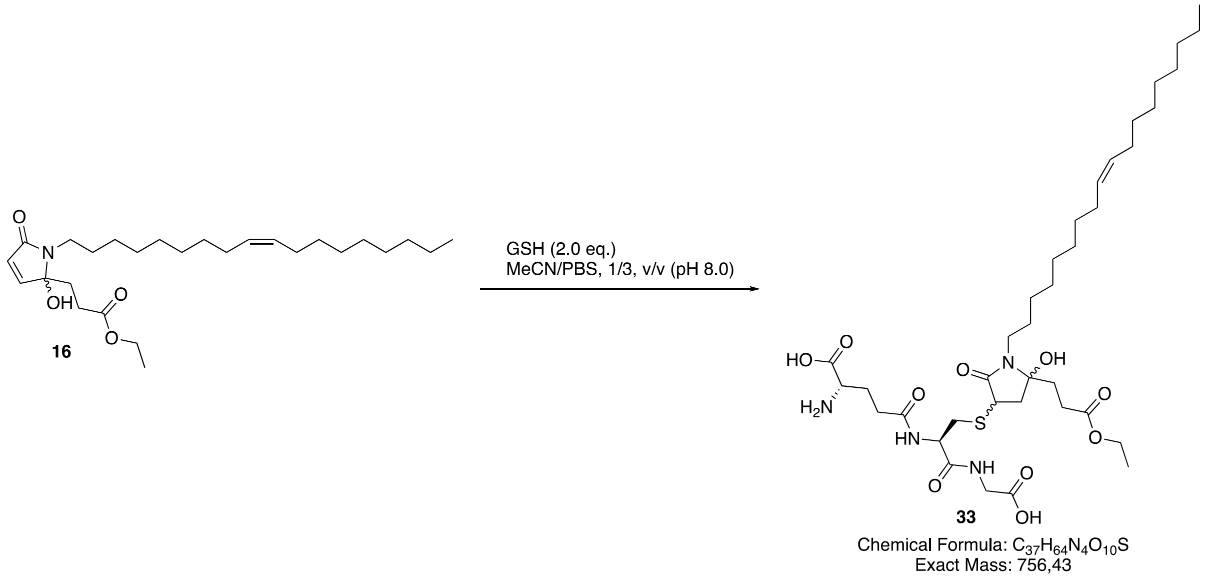

Scheme S 17: Glutathione conjugation to 5HP2O building block 16.

The reaction was accomplished according to the general experimental procedure described above, utilizing the lactam **16** (0.005 mmol, 2.25 mg) and GSH (0.0105 mmol, 3.2 mg) in 1.2 mL MeCN/PBS (1M, pH 8.0) (1/3 v/v). The formation of the desired conjugate was confirmed by LC-MS analysis. Due to solubility incompatibilities, no full conversion was observed. LC-MS; Calculated mass for $C_{37}H_{63}N_4O_{10}S$: 755.4 $[M-H]^-$; mass found: 755.3 $[M-H]^-$.

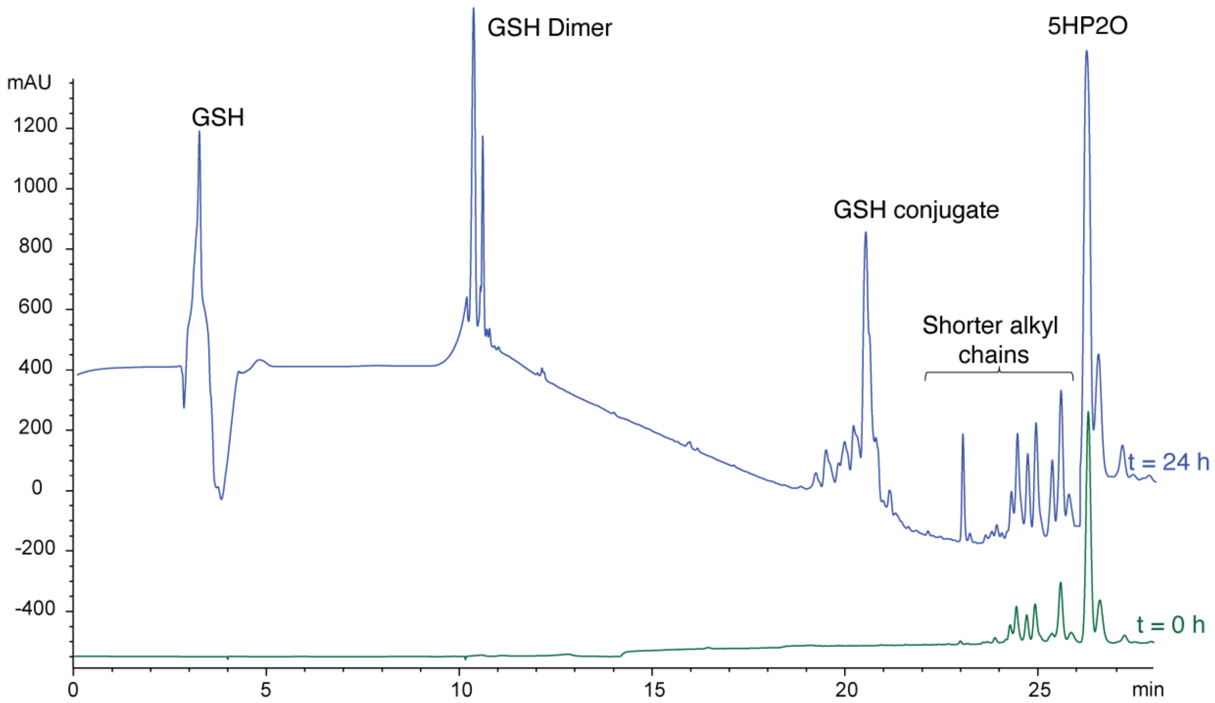

*Figure S 41: Follow up of glutathione addition to 5HP2O building block using RP-HPLC. LC (214 nm) traces are shown at different time points. * The oleyl amine was obtained from sigma as 98% purity containing shorter alkyl chains.*

7. Reactivity of 5HP2O building blocks towards proteins

7.1. MB23 Alphabody Protein

MB23 was expressed and purified as described elsewhere.[6]
PDB entries of related structures: 5MJ3 and 5MJ4.

7.1.1. MB23 pre-reduction

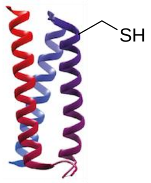

m/z: 11468.6

TCEP-HCl (0.023 mg, 0.08 µmol, 2 eq.) was added to 100 µL of MB23 (c = 4.6 mg/mL in 50 mM MES pH 6.0, 0.5 M NaCl) and shaken at r.t. for 60 minutes. After this time, the protein was separated from TCEP-HCl and buffer exchanged into 10 mM Tris, pH 7.4 by means of a Micro BioSpin 6 column (Bio-Rad). Reduced protein was analysed by LC-MS, the associated ESI-MS is shown below.

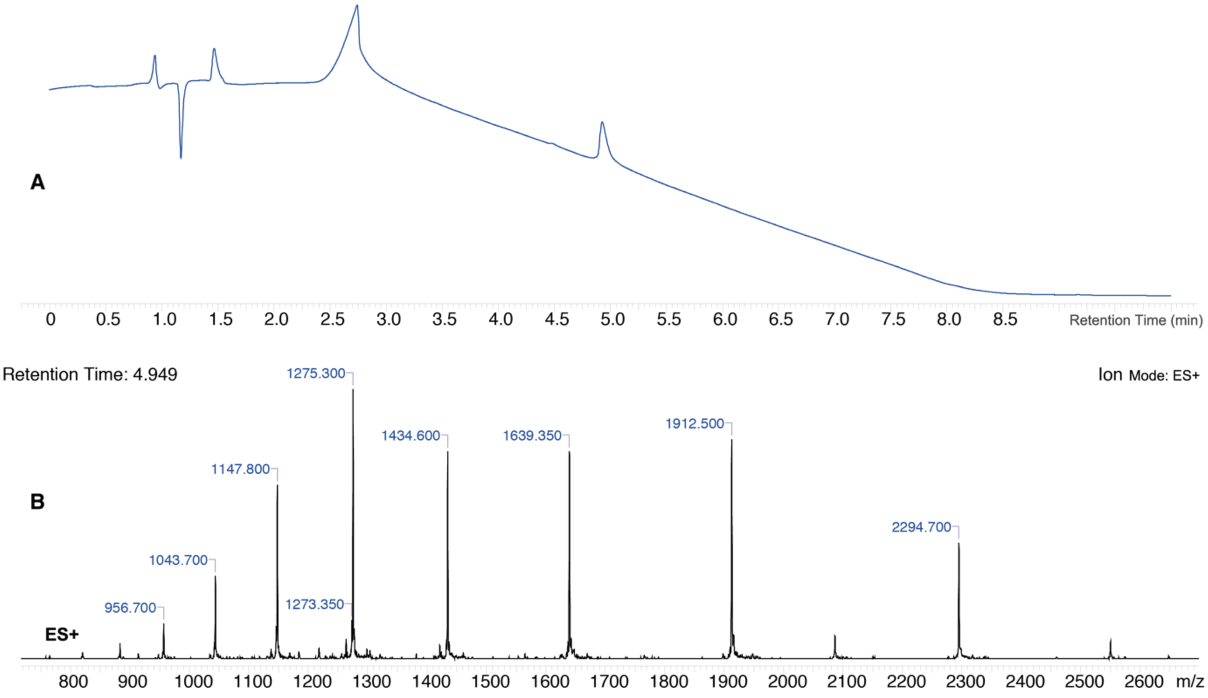

Figure S 42: LC-MS analysis of Alphabody. (A) Liquid Chromatogram (214 nm) and (B) Corresponding MS of Alphabody peak at 4.94 min.

[6] J. Desmet, K. Verstraete, Y. Bloch, E. Lorent, Y. Wen, B. Devreese, K. Vandenbroucke, S. Loverix, T. Hettmann, S. Deroo, K. Somers, P. Henderikx, I. Lasters, S. N. Savvides, *Nat. Commun.*, 2014, ***5***, 5237.

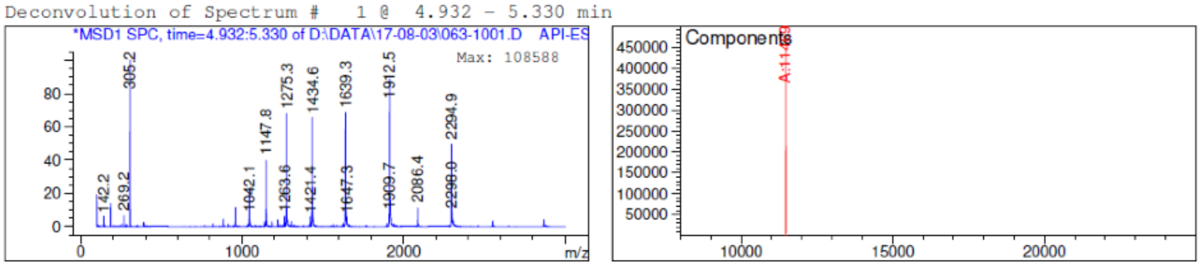

Figure S 43: MS analysis of reduced MB23 Alphabody. Deconvoluted mass is shown on the right. Calculated mass: 11468.6 Da, observed mass: 11469 Da.

7.1.2. Verification of free thiol functionality by reaction of MB23 with Ellman's reagent

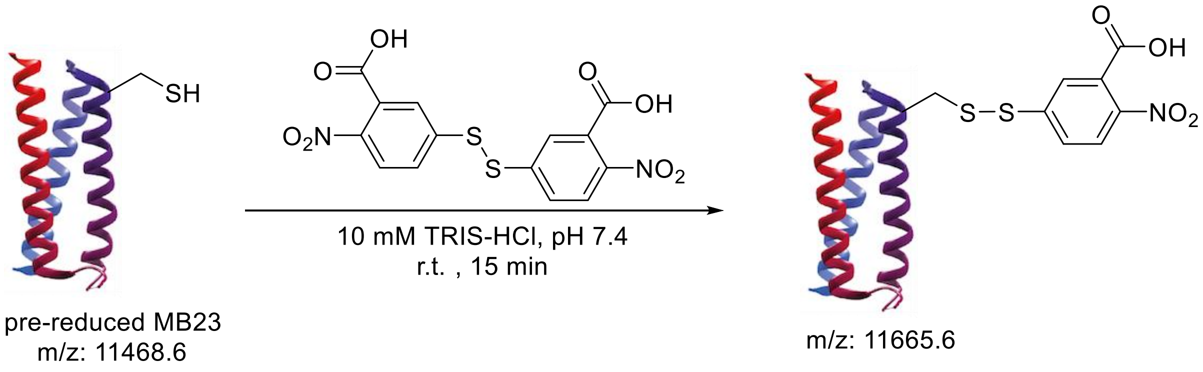

Scheme S 18: Reaction scheme for verification of free thiols on reduced Alphabody using Ellman's reagent.

A solution of Ellman's reagent was prepared (0.6 mg in 108 µL PBS, pH 7.4). 10 µL of this solution was added to 75 µL of reduced MB23 (c = 0.2 mg/mL in 10 mM Tris-HCl, pH 7.4) and shaken at r.t. for 15 minutes. After this time, the protein was separated from excess Ellman's reagent by means of a Micro BioSpin 6 column (Bio-Rad). Protein was analysed by LC-MS, the associated MS is shown below. By conjugation of a small building block to a protein, the difference in mass with the native alphabody mass is too small to cause a shift in protein peak. Therefore we look to the deconvoluted masses of the protein peak.

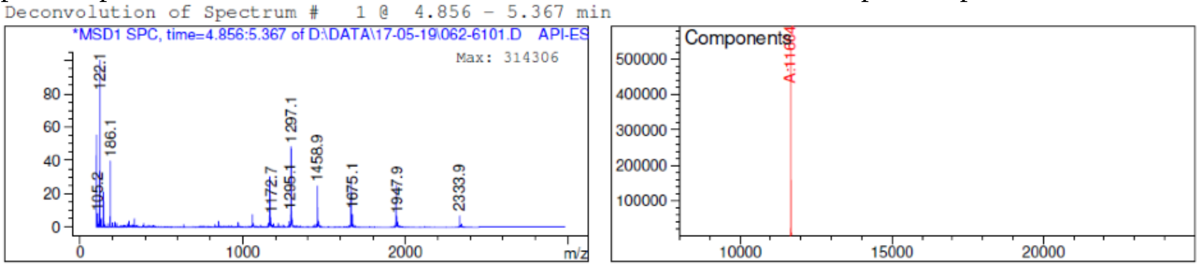

Figure S 44: ESI-MS analysis of MB23 Alphabody conjugate. Deconvoluted mass is shown on the right. Calculated mass: 11665 Da, observed mass: 11664 Da.

7.1.3. Alphabody conjugation to 5HP2O building blocks

Conjugation to 5HP2O building block 2

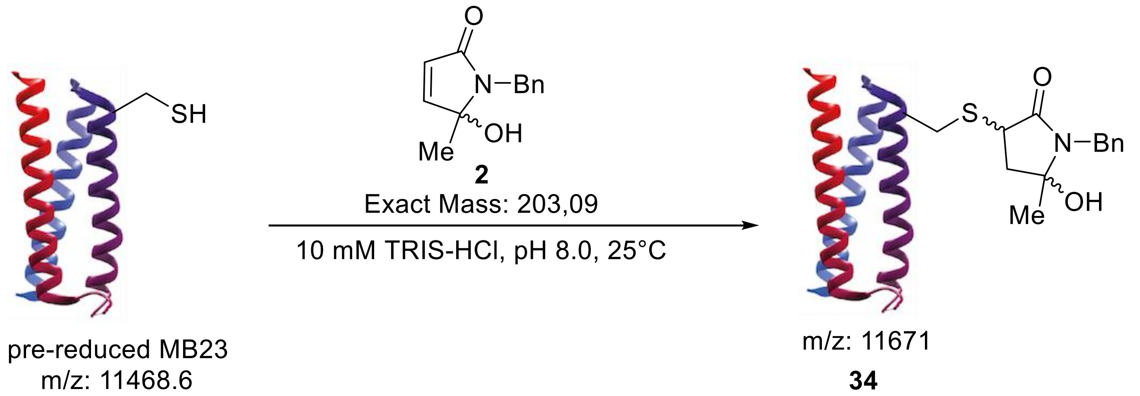

*Scheme S 19: Conjugation of MB23 Alphabody to 5HP2O building block **2**.*

MB23 was first reduced with TCEP to remove any dimeric species formed during storage following the procedure described above. To 150.25 µL of reduced MB23 (0.25 mg, 21.8 nmol, c = 1.21 mg/mL in 10 mM Tris-HCl, pH 8.0) was added 5HP2O building block **2** (1.125 µL from 40 mg/mL solution in DMSO, 0.218 µmol, 10 eq.), and the reaction was allowed to shake at 25 °C overnight. Solvent was removed by speed vac and conjugated **34** was resuspended in 250 µL H_2O (c = 1 mg/mL) for LCMS analysis. The associated LC-MS is shown below.

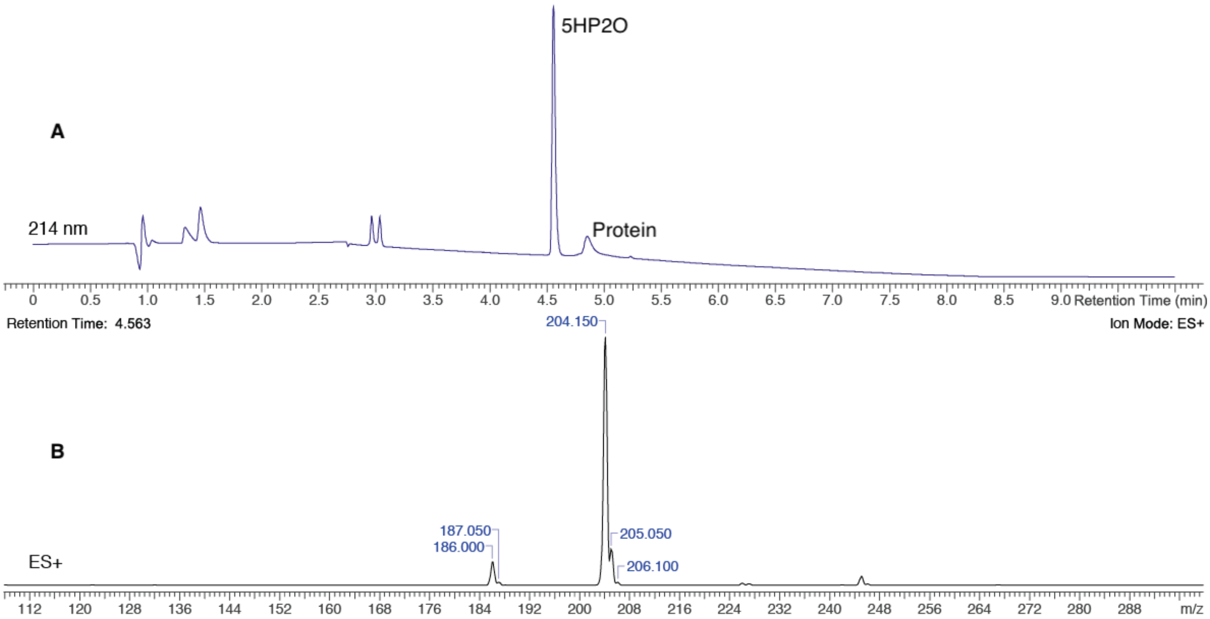

Figure S 45: LC-MS analysis of crude conjugation reaction mixture. (A) Liquid chromatogram (214 nm) (B) Mass spectrum of excess 5HP2O building block at 4.56 min.

Deconvolution of Spectrum # 1 @ 4.868 min

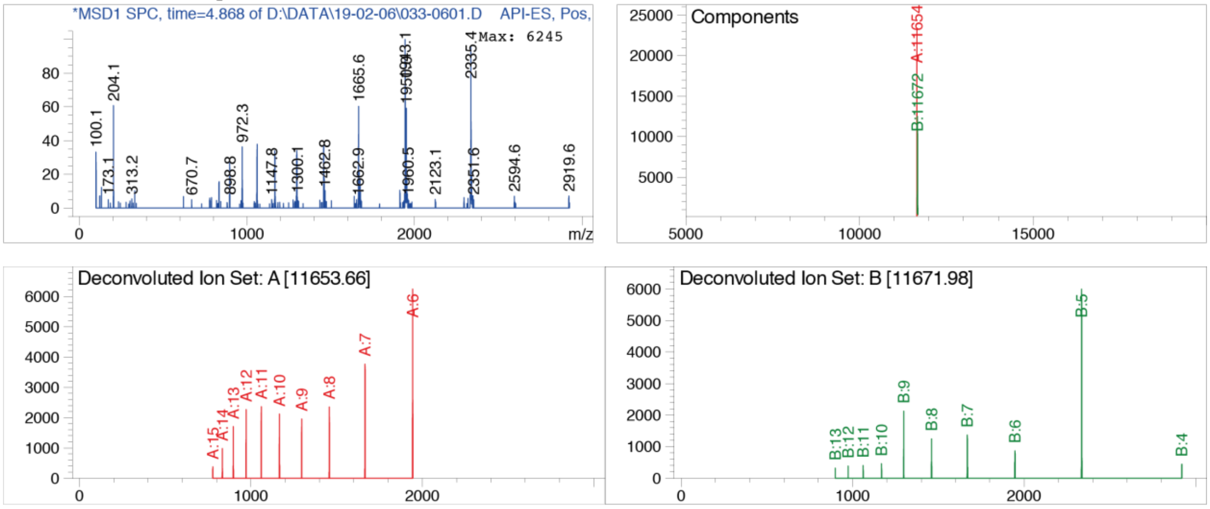

Component	Molecular Weight	Absolute Abundance	Relative Abundance
A	11653.66	23462	100.00
B	11671.98	12525	53.38

*** End of Report ***

Figure S 46: Deconvoluted mass of peak at 4.87 min which corresponds to the Alphabody-conjugate. Calculated mass: 11671 Da, observed masses: (B) 11672 $[M+H]^+$ and (A) 11654 $[M+H-H_2O]^+$.

Screening of buffers – pH influence

MB23 was first reduced with TCEP to remove any dimeric species formed during storage following the procedure described above followed by incubation in appropriate 10 mM TRIS buffers with pH adjustment to 6, 7, 8 and 9 after which the 5HP2O is added as described above for **2**. MALDI-TOF analysis was performed for each pH after 2 hours and overnight.

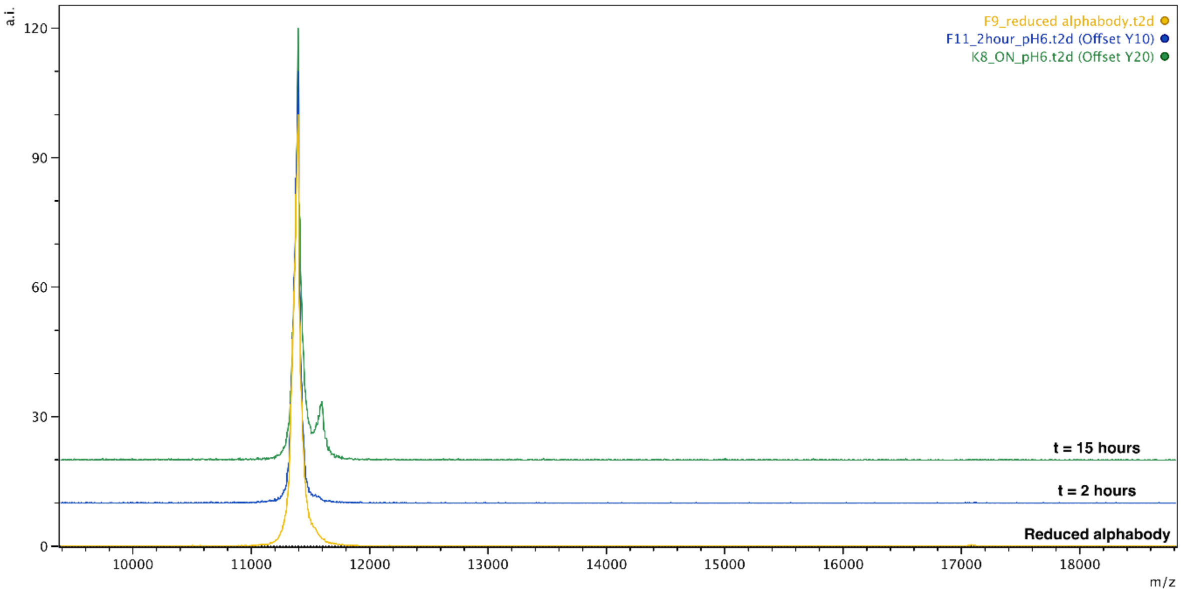

Figure S 47: MALDI-TOF analysis of alphabody conjugate after 2 hours and after overnight at a pH of 6.

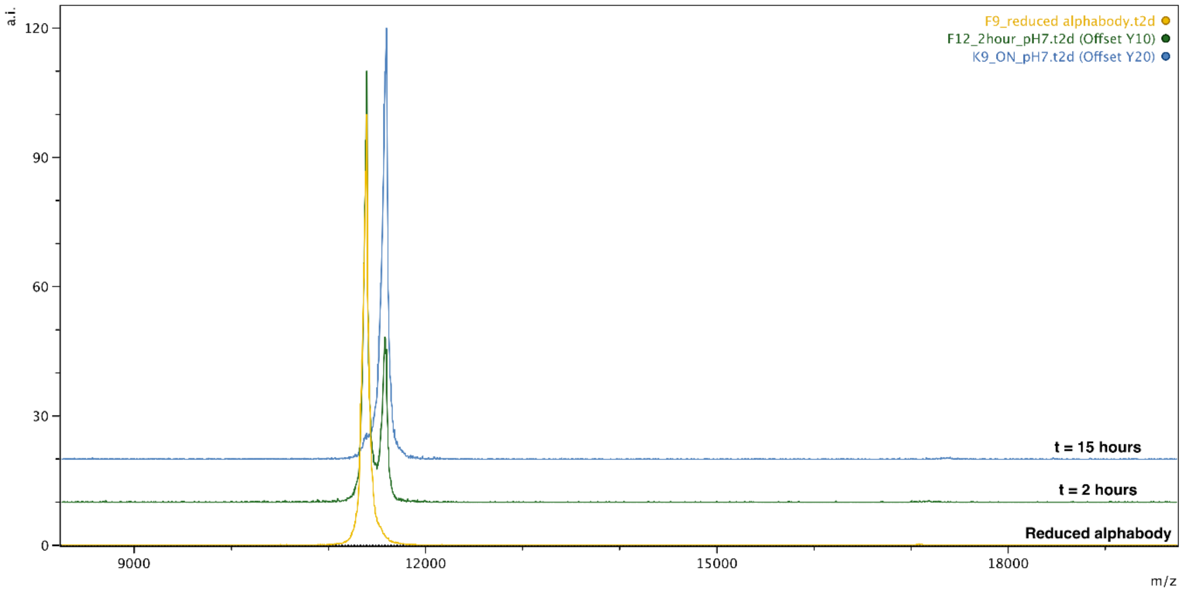

Figure S 48: MALDI-TOF analysis of alphabody conjugate after 2 hours and after overnight at a pH of 7.

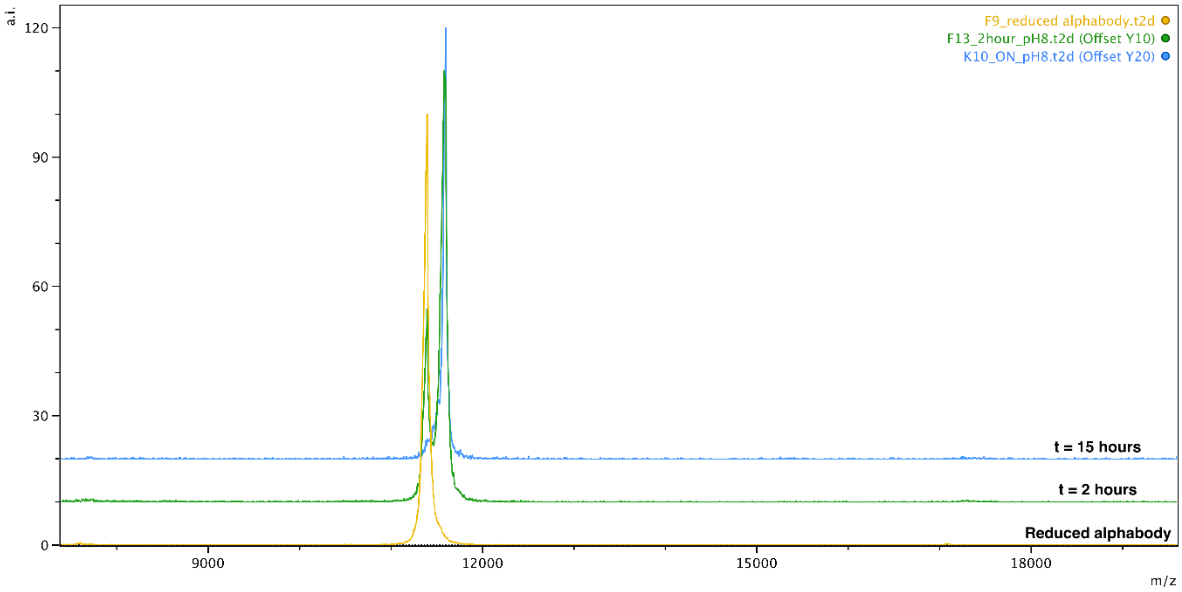

Figure S 49: MALDI-TOF analysis of alphabody conjugate after 2 hours and after overnight at a pH of 8.

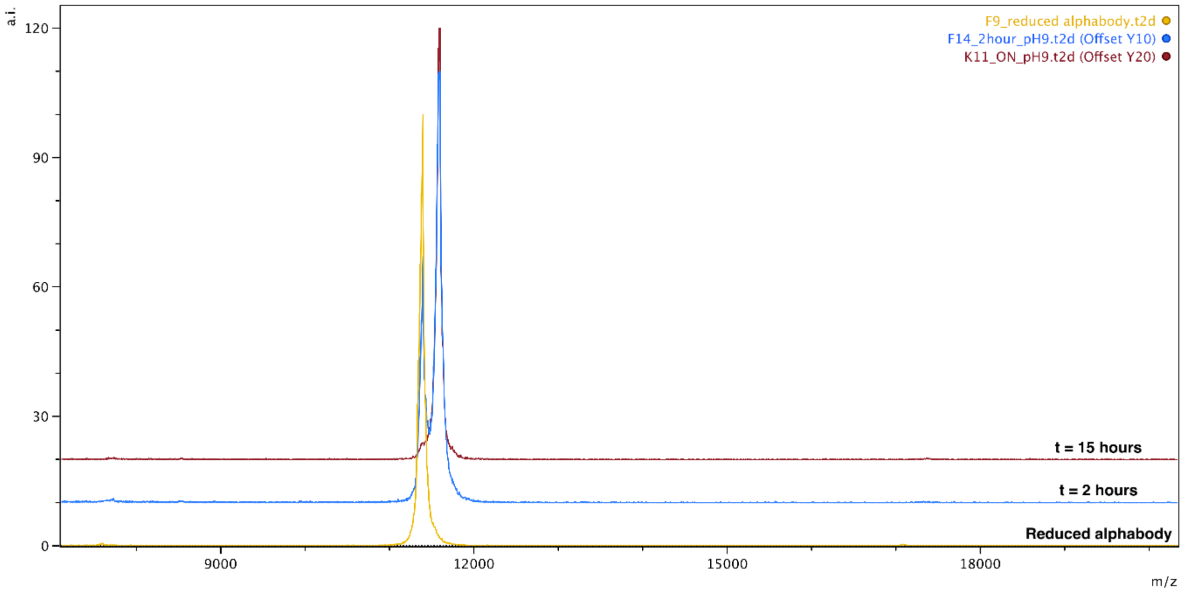

Figure S 50: MALDI-TOF analysis of alphabody conjugate after 2 hours and after overnight at a pH of 9.

Conjugation to 5HP2O building block 3

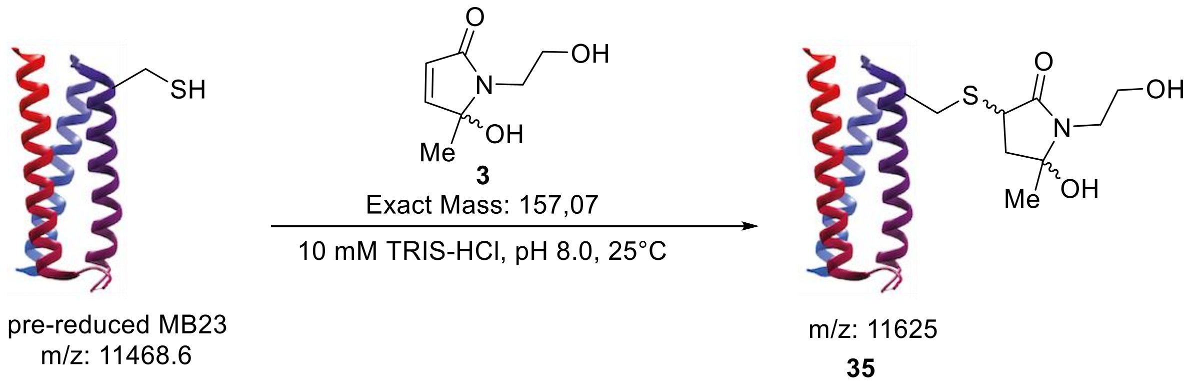

Scheme S 20: Conjugation of MB23 Alphabody to 5HP2O building block ***3****.*

MB23 was first reduced with TCEP to remove any dimeric species formed during storage following the procedure described above. To 150.25 µL of reduced MB23 (0.25 mg, 21.8 nmol, c = 1.21 mg/mL in 10 mM Tris-HCl, pH 8.0) was added 5HP2O building block **3** (4.28 µL from 8 mg/mL solution in 10% DMSO/H_2O, 0.218 µmol, 10 eq.), and the reaction was allowed to shake at 25 °C overnight. After this time, the protein was separated from excess 5HP2O reagent by a MicroSpin 6 column from BioRad. Protein conjugate **35** was analysed by LC-MS.

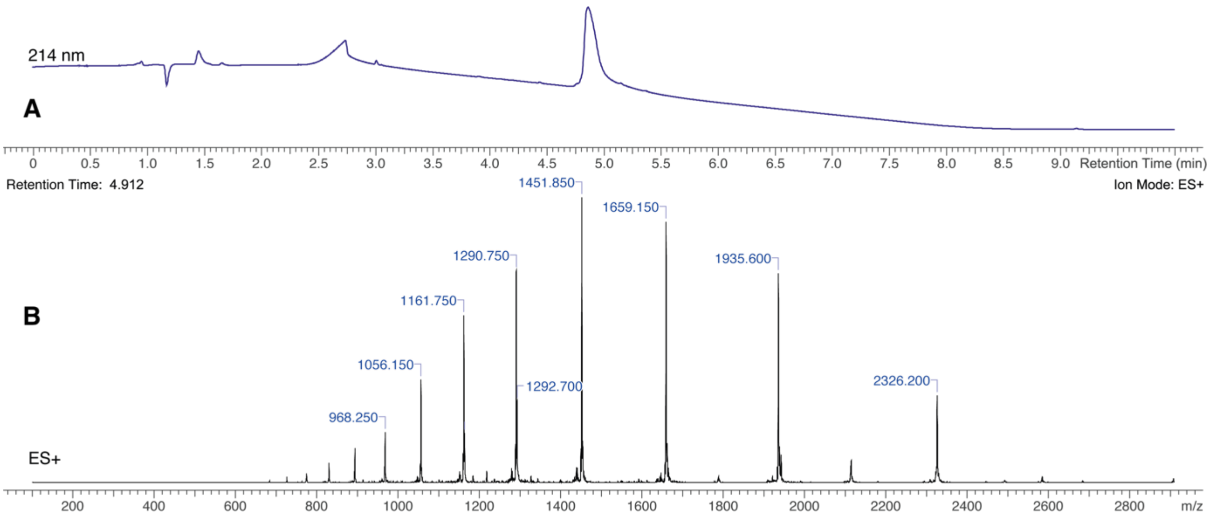

Figure S 51: LC-MS analysis of conjugation reaction mixture. (A) Liquid chromatogram (214 nm) (B) Mass spectrum of alphabody protein conjugate.

Deconvolution of Spectrum # 1 @ 4.912 min

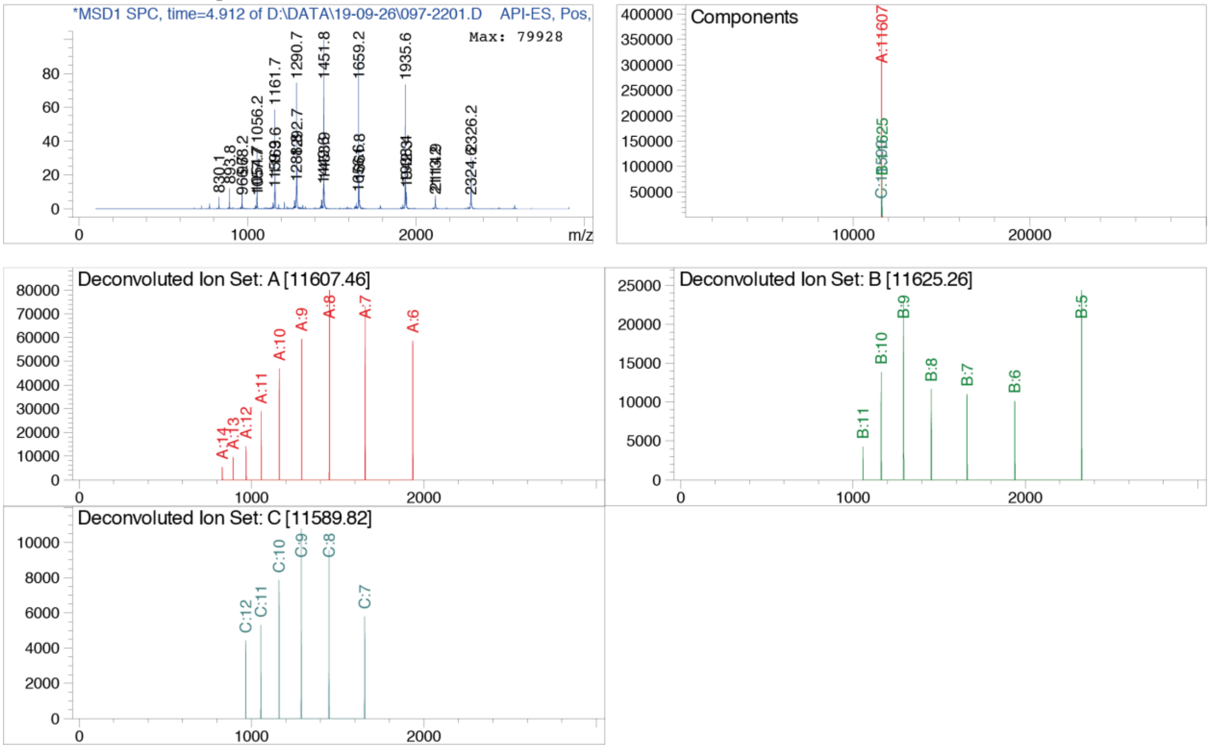

Component	Molecular Weight	Absolute Abundance	Relative Abundance
A	11607.46	373679	100.00
B	11625.26	96573	25.84
C	11589.82	43329	11.60

*** End of Report ***

Figure S 52: Deconvoluted mass of the Alphabody-conjugate. Calculated mass: 11626 Da, observed masses: (A) 11607 Da $[M+H-H_2O]^+$, (B) 11625 Da $[M+H]^+$ and (C) 11589 Da $[M+H-2H_2O]^+$.

Conjugation to 5HP2O building block 7

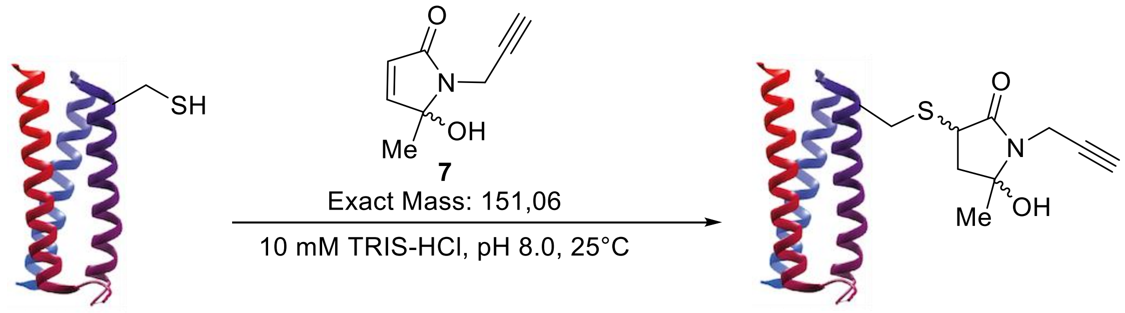

pre-reduced MB23
m/z: 11468.6

m/z: 11620
36

*Scheme S 21: Conjugation of MB23 Alphabody to 5HP2O building block **7**.*

MB23 was first reduced with TCEP to remove any dimeric species formed during storage following the procedure described above. To 150.25 µL of reduced MB23 (0.25 mg, 21.8 nmol, c = 1.21 mg/mL in 10 mM Tris-HCl, pH 8.0) was added 5HP2O building block **7** (3.29 µL from 10 mg/mL solution in DMSO, 0.218 µmol, 10 eq.), and the reaction was allowed to shake at 25 °C overnight. Solvent was removed by speed vac and conjugated **36** was resuspended in 250 µL H_2O (c = 1 mg/mL) for LCMS analysis.

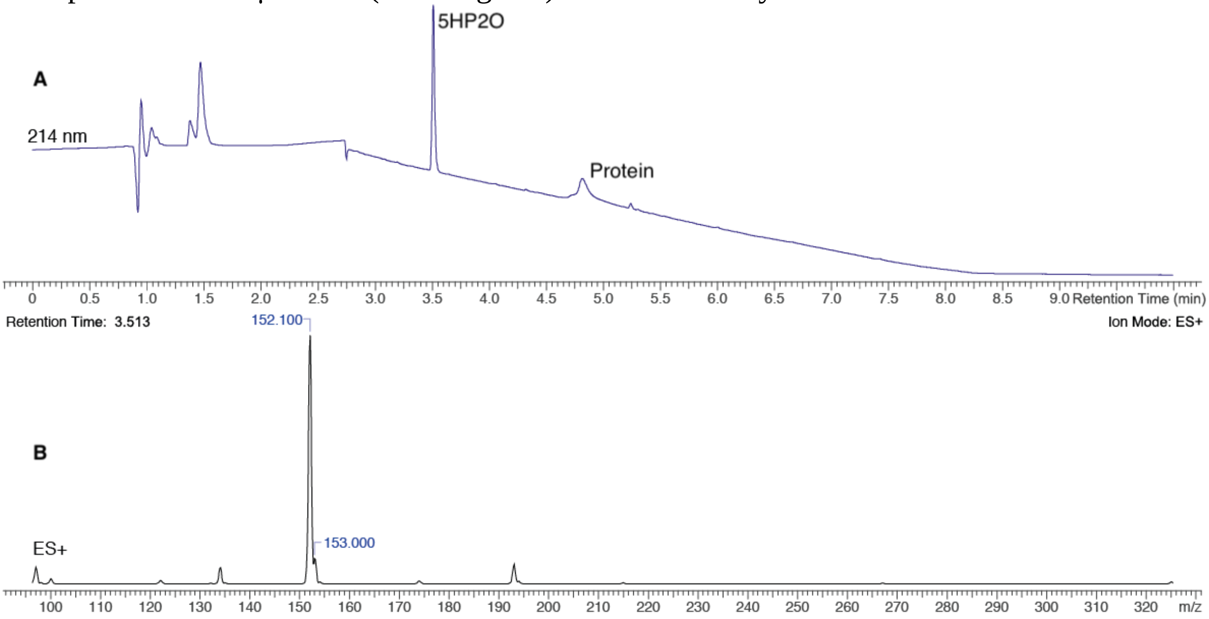

Figure S 53: LC-MS analysis of crude conjugation reaction mixture. (A) Liquid chromatogram (214 nm) (B) Mass spectrum of excess 5HP2O building block at 3.51 min.

Deconvolution of Spectrum # 1 @ 4.849 min

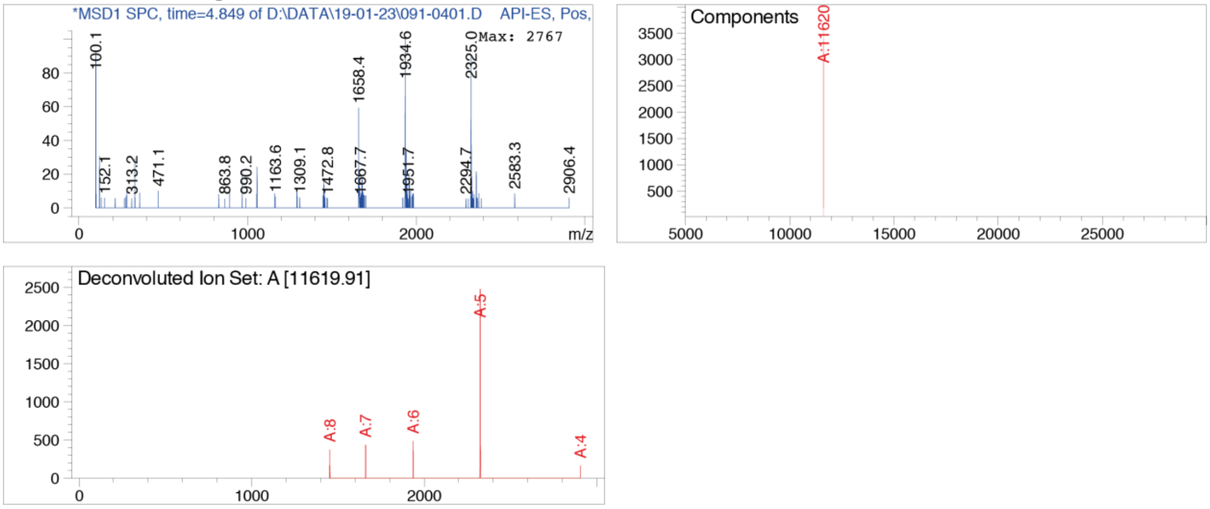

Component	Molecular Weight	Absolute Abundance	Relative Abundance
A	11619.91	3612	100.00

*** End of Report ***

Figure S 54: Deconvoluted mass of peak at 4.85 min which corresponds to the Alphabody-conjugate. Calculated mass: 11620 Da, observed mass: 11619.9 [M+H]+.

Kinetic follow up

Deconvoluted masses of LC-MS analysis after 1 hour:

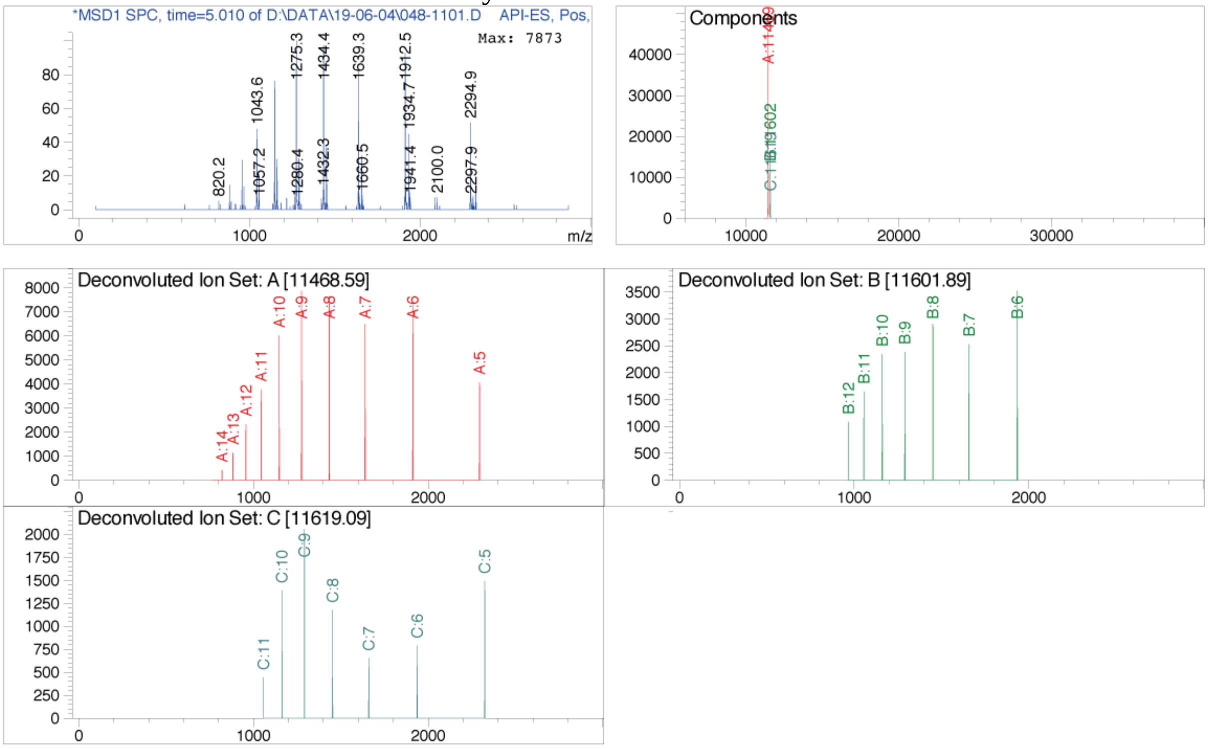

Component	Molecular Weight	Absolute Abundance	Relative Abundance
A	11468.59	46268	100.00
B	11601.89	16303	35.24
C	11619.09	7753	16.76

Figure S 55: Deconvoluted masses after 1 hour. Calculated mass: 11620 Da, observed masses: (A) 11468 [M+H]+ unmodified Alphabody, (B) 11602 [M+H-H_2O]+ and (C) 11619 [M+H]+Corresponding to the product conjugate.

Deconvoluted masses of LC-MS analysis after 6 hour:

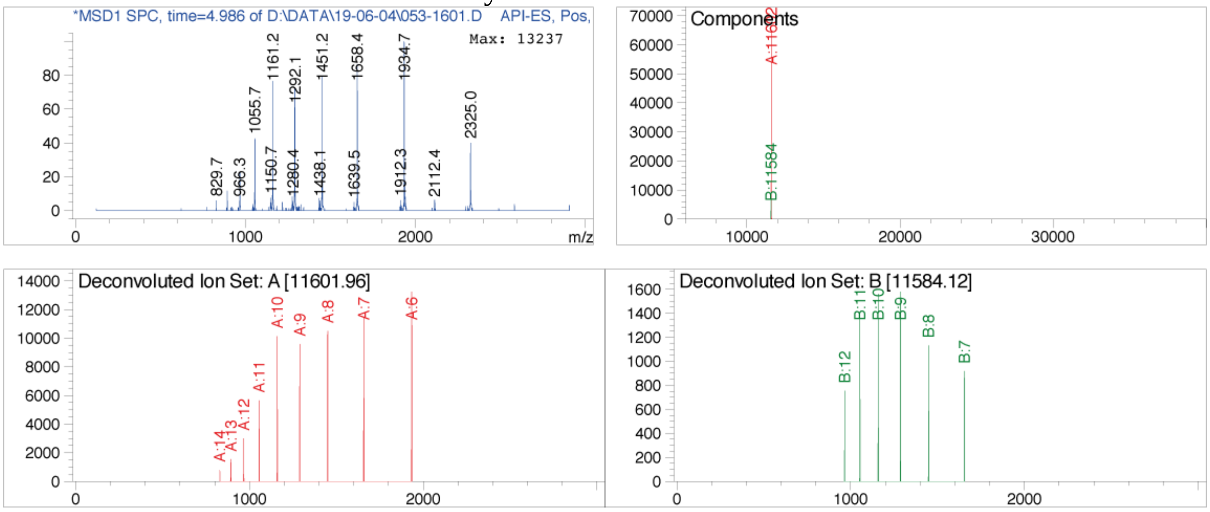

Component	Molecular Weight	Absolute Abundance	Relative Abundance
A	11601.96	65299	100.00
B	11584.12	7260	11.12

Figure S 56: Deconvoluted masses after 6 hours. Calculated mass: 11620 Da, observed masses: (A) 11602 $[M+H-H_2O]^+$ and (B) 11584 $[M+H-2H_2O]^+$.

Conjugation to 5HP2O building block 9

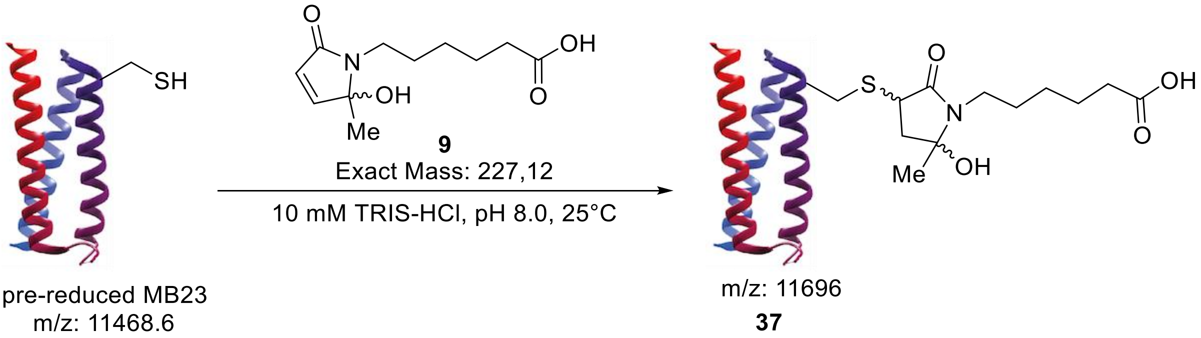

*Scheme S 22: Conjugation of MB23 Alphabody to 5HP2O building block **9**.*

MB23 was first reduced with TCEP to remove any dimeric species formed during storage following the procedure described above. To 207.25 µL of reduced MB23 (0.25 mg, 21.8 nmol, c = 1.21 mg/mL in 10 mM Tris-HCl, pH 8.0) was added 5HP2O building block **9** (1.666 µL from 30 mg/mL solution in DMSO, 0.218 µmol, 10 eq.), and the reaction was allowed to shake at 25 °C overnight. Solvent was removed by speed vac and conjugated **37** was resuspended in 250 µL H_2O (c = 1 mg/mL) and analysed by LC-MS. The associated LC-MS is shown below.

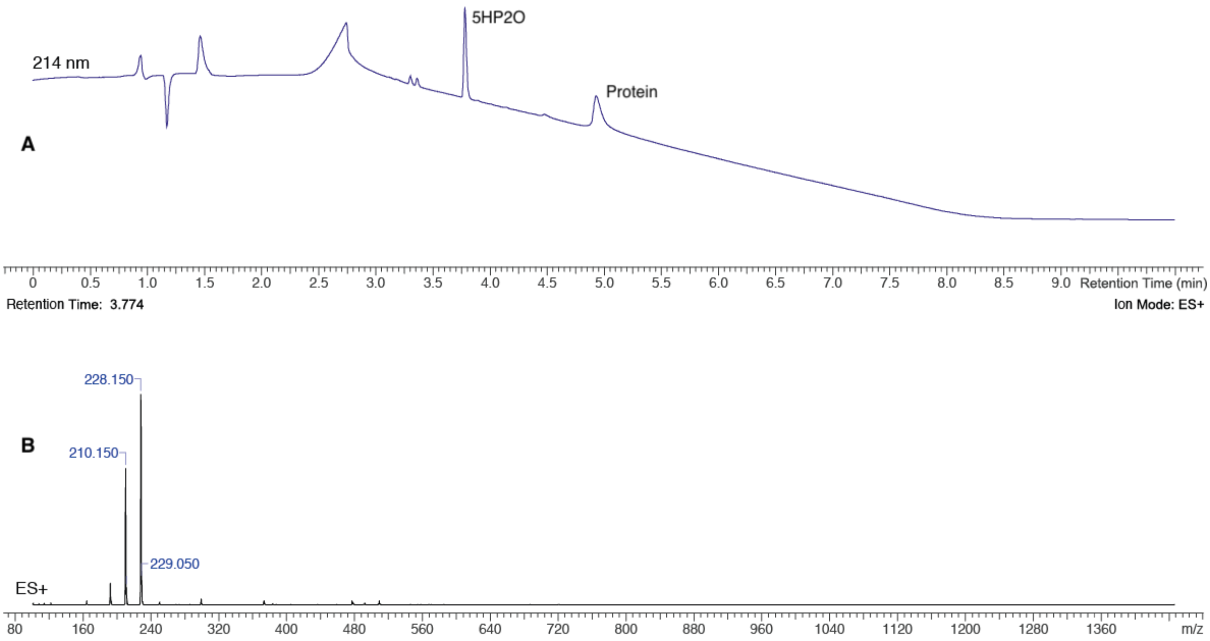

Figure S 57: LC-MS analysis of crude conjugation reaction mixture. (A) Liquid chromatogram (214 nm) (B) Mass spectrum of excess 5HP2O building block at 3.77 min.

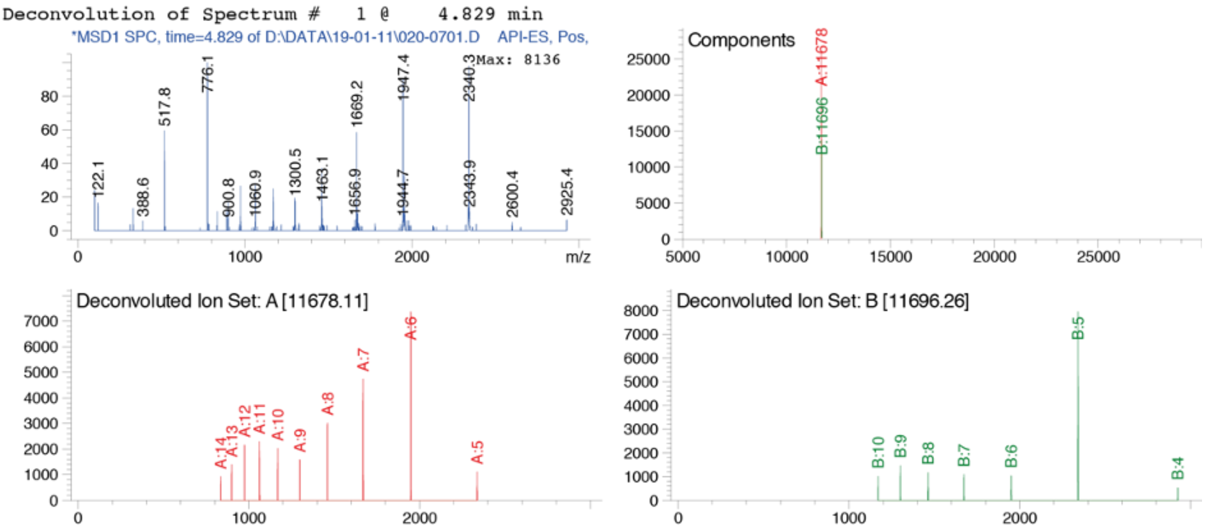

Component	Molecular Weight	Absolute Abundance	Relative Abundance
A	11678.11	26194	100.00
B	11696.26	13730	52.42

*** End of Report ***

Figure S 58: Deconvoluted mass of peak at 4.83 min which corresponds to the Alphabody-conjugate. Calculated mass: 11696 Da, observed masses: (A) 11678 $[M+H-H_2O]^+$ and (B) 11696 $[M+H]^+$.

Conjugation to 5HP2O building block 11

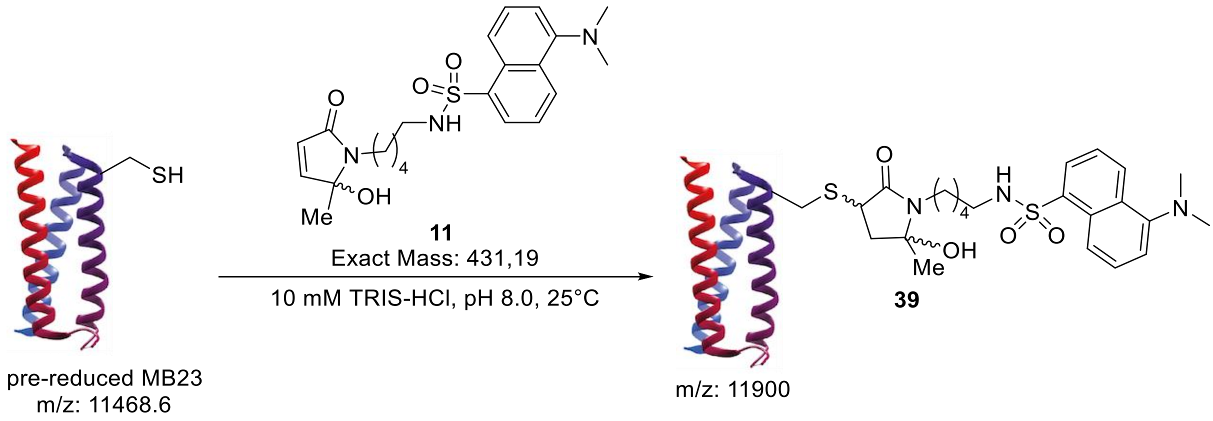

*Scheme S 23: Conjugation of MB23 Alphabody to 5HP2O building block **11**.*

MB23 was first reduced with TCEP to remove any dimeric species formed during storage following the procedure described above. To 207.25 µL of reduced MB23 (0.25 mg, 21.8 nmol, c = 1.21 mg/mL in 10 mM Tris-HCl, pH 8.0) was added 5HP2O building block **11** (4.70 µL from 20 mg/mL solution in DMSO, 0.218 µmol, 10 eq.), and the reaction was allowed to shake at 25 °C overnight. After this time, the protein was separated from excess 5HP2O reagent by a MicroSpin 6 column from BioRad and analysed by LC-MS. The associated MS is shown below.

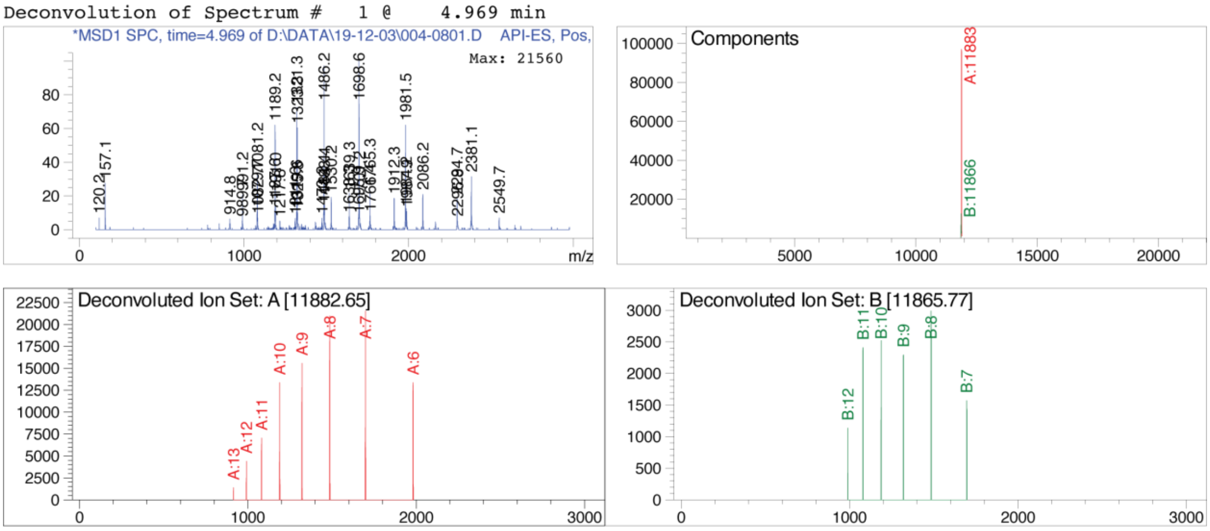

Component	Molecular Weight	Absolute Abundance	Relative Abundance
A	11882.65	97092	100.00
B	11865.77	12520	12.89

Figure S 59: Deconvoluted mass of peak at 4.969 min which corresponds to the Alphabody-conjugate. Calculated mass (m/z): 11899.8 Da, observed masses: (A) 11882.65 $[M+H-H_2O]^+$ and (B) 11865.77 $[M+H-2*H_2O]^+$.

Conjugation to 5HP2O building block 20

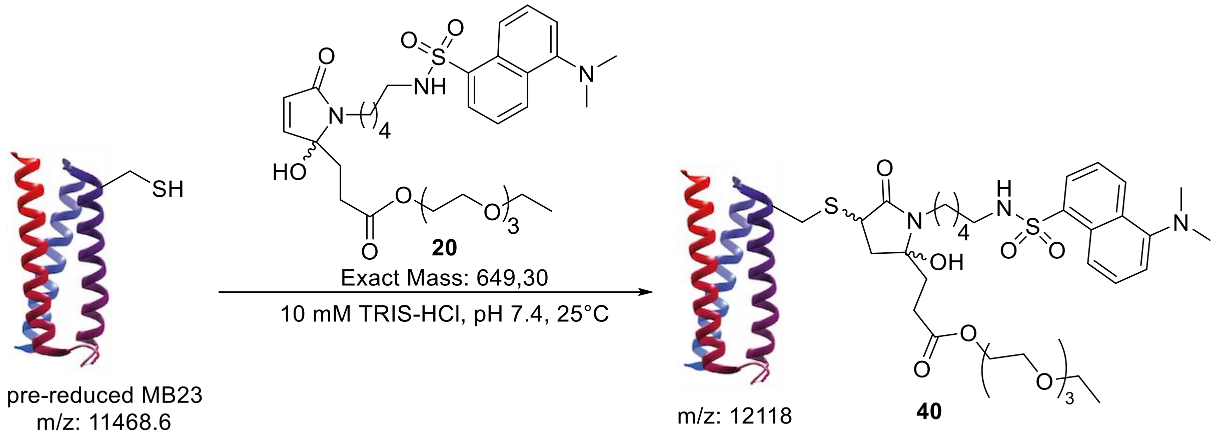

*Scheme S 24: Bifunctionalization of MB23 Alphabody to 5HP2O building block **20**.*

MB23 was first reduced with TCEP to remove any dimeric species formed during storage following the procedure described above. To 78 µL of reduced MB23 (0.0621 mg, 5.42 nmol, c = 0.8 mg/mL in 10 mM Tris-HCl pH 7.4/DMSO (68/10)) was added 5HP2O building block (1.2 µL from 28.5 mg/mL solution in DMSO, 0.054 µmol, 10 eq.), and the reaction was allowed to shake at 25°C overnight. Reaction was followed by MALDI/LCMS and after 6 hours, the protein was separated from excess 5HP2O reagent by a MicroSpin 6 column from BioRad and analysed by LC-MS. Associated MALDI and MS spectra are shown below.

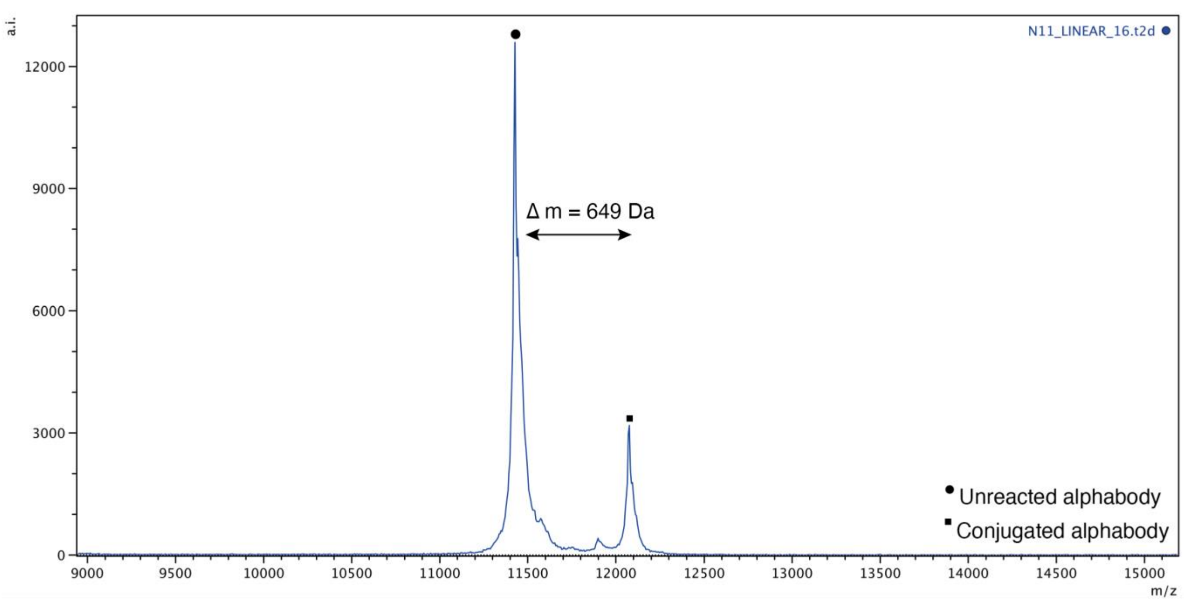

*Figure S 60: MALDI-TOF MS analysis after 2 hours. Mass difference can be seen of 649 Da which corresponds with the mass of 5HP2O building block **20**.*

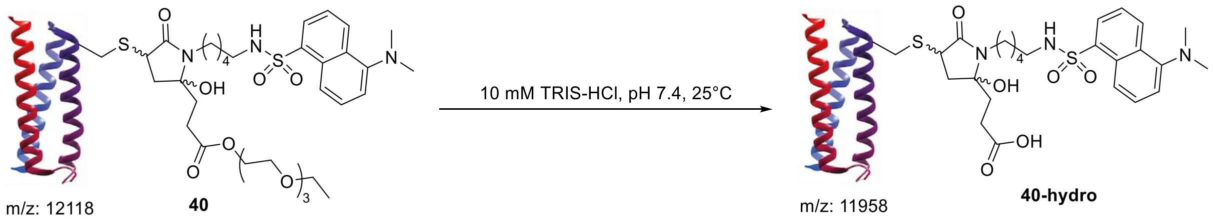

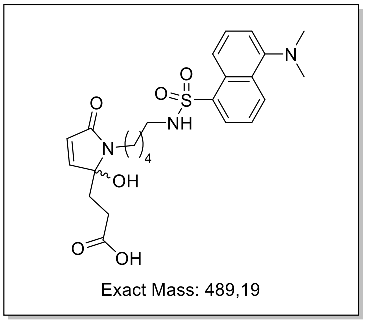

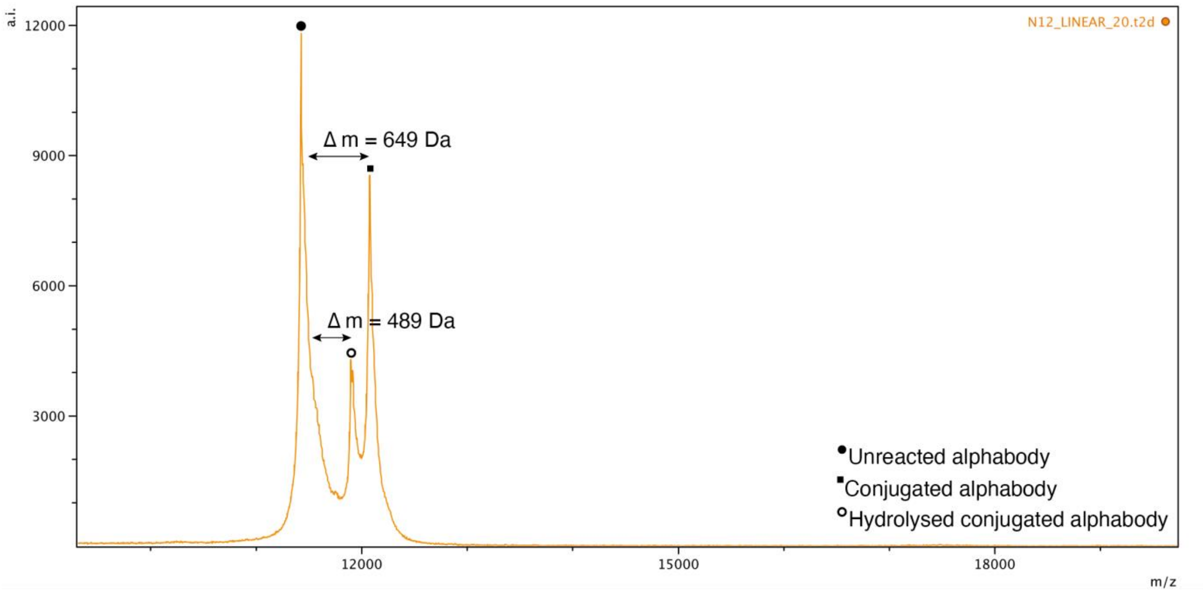

Figure S 61: MALDI-TOF MS analysis after 4 hours. Almost 50% conversion is observed after 4 hours. Additional conjugate is observed due to hydrolysis of the ester bond.

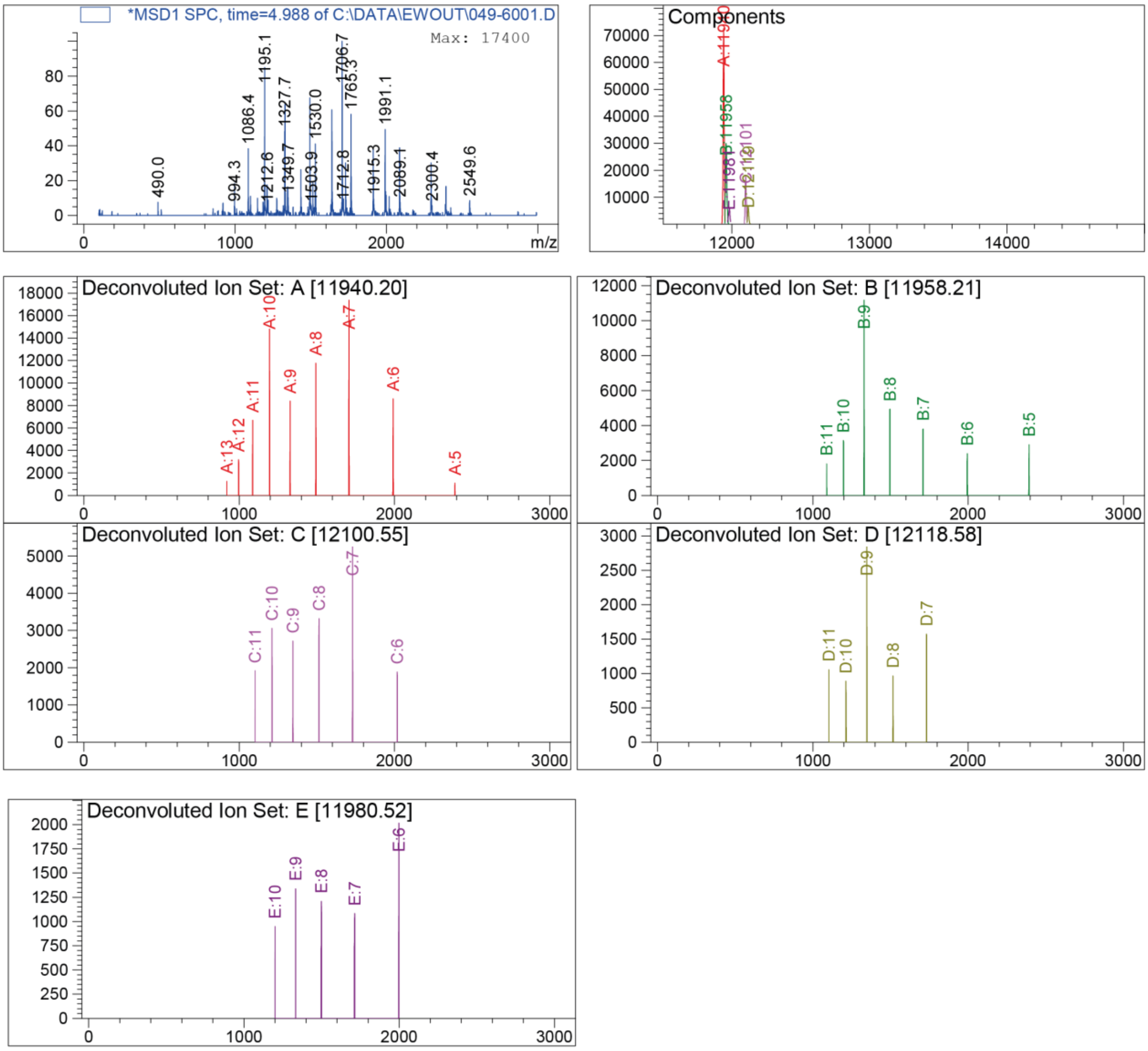

Component	Molecular Weight	Absolute Abundance	Relative Abundance
A	11940.20	72577	100.00
B	11958.21	29957	41.28
C	12100.55	17754	24.46
D	12118.58	6950	9.58
E	11980.52	6188	8.53

*Figure S 62: Deconvoluted mass of peak corresponding to the Alphabody-conjugate after 6 hours. Calculated mass (m/z): 12118 Da, observed masses: (A) 11940 [(**40-hydro**) – H_2O]$^+$, (B) 11958 [(**40-hydro**)]$^+$, (C) 12100 [M+H-H_2O]$^+$,(D) 12118 [M+H]$^+$and (E)11980 [M+H-2H_2O]$^+$.*

7.1.4. Alphabody conjugation to 6-maleimidohexanoic acid

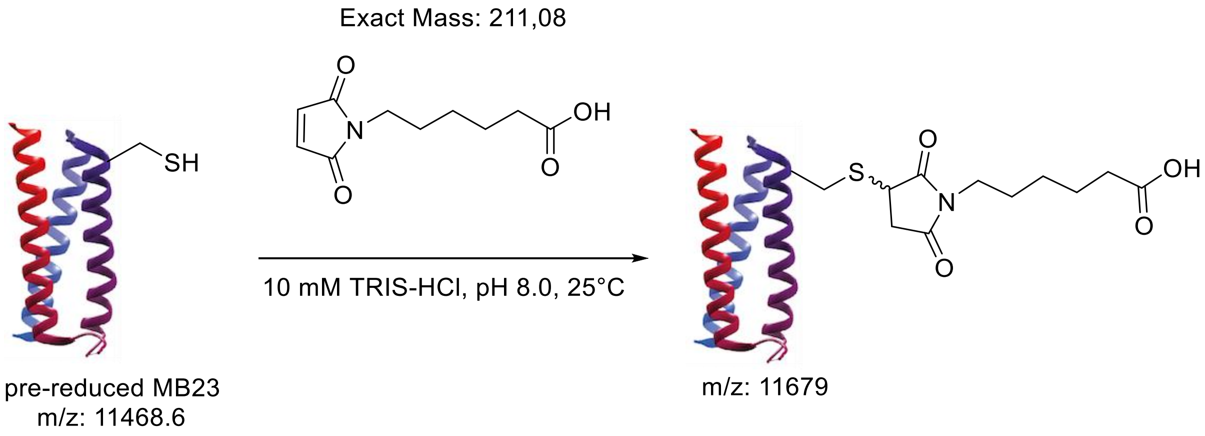

Scheme S 25: Conjugation scheme of Alphabody towards Maleimide.

MB23 was first reduced with TCEP to remove any dimeric species formed during storage following the procedure described above. To 150.25 µL of reduced MB23 (0.25 mg, 21.8 nmol, c = 1.21 mg/mL in 10 mM Tris-HCl, pH 8.0) was added 6-maleimidohexanoic acid (2.3 µL from 10 mg/mL solution in DMSO, 0.109 µmol, 5 eq.), and the reaction was allowed to shake at 25 °C overnight. Solvent was removed by speed vac and alphabody conjugate was resuspended in 250 µL H_2O (c = 1 mg/mL) for LCMS analysis. The associated MS is shown below.

Deconvolution of Spectrum # 1 @ 4.965 min

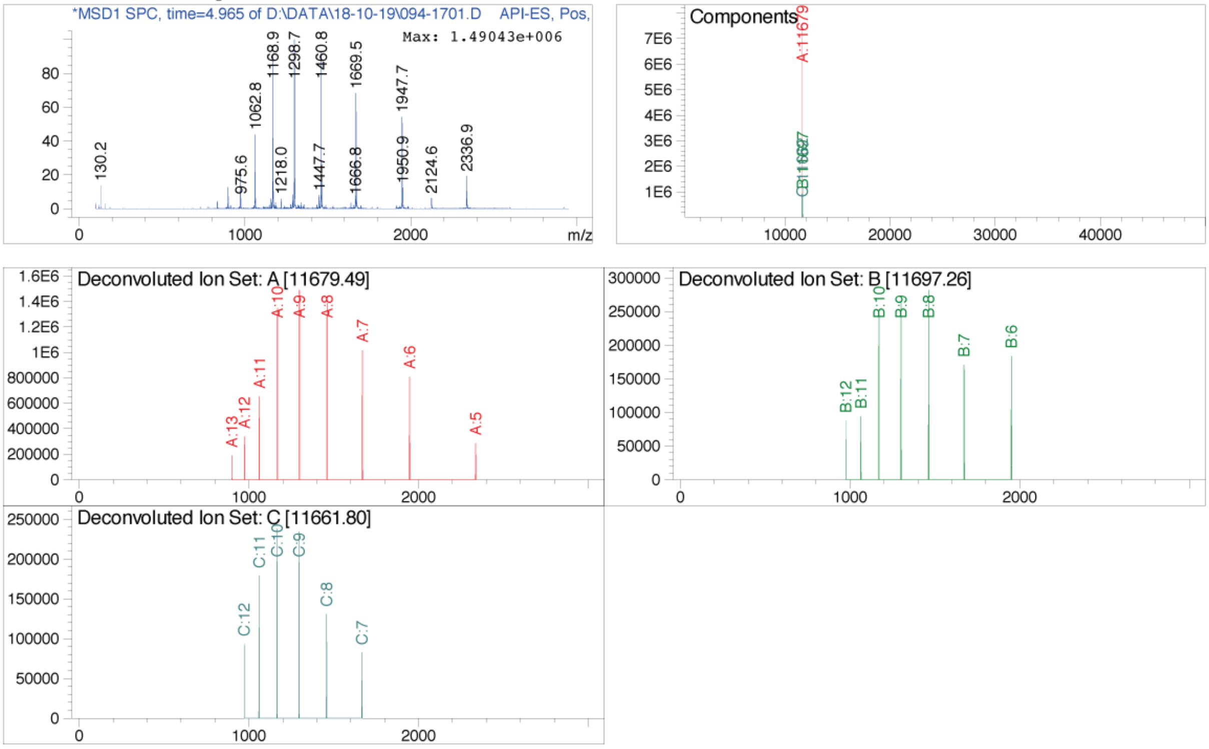

Component	Molecular Weight	Absolute Abundance	Relative Abundance
A	11679.49	7428980	100.00
B	11697.26	1291553	17.39
C	11661.80	939149	12.64

Figure S 63: Deconvoluted masses of maleimide conjugation to Alphabody. Calculated mass: 11679 Da $[M+H]^+$, Observed masses: (A) 11679 $[M+H]^+$, (B) 11697 $[M+H+H_2O]^+$ corresponding to hydrolyzed conjugate and (C) 11661 $[M+H-H_2O]^+$.

7.1.5. Hydrolytic stability of the alphabody conjugate

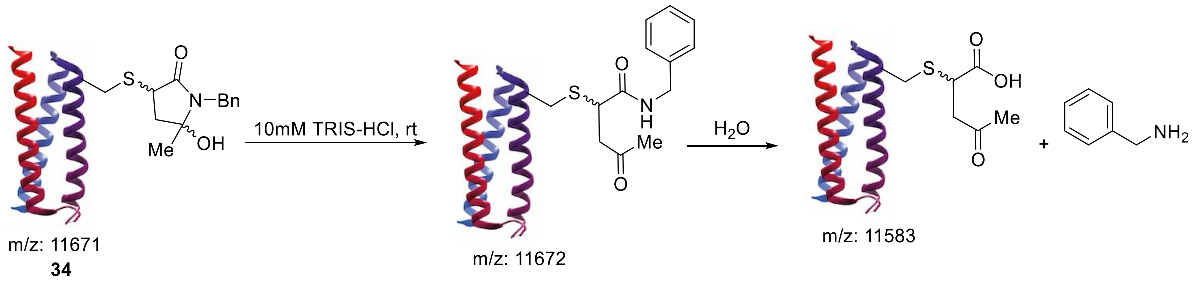

Scheme S 26: Reaction scheme of possible ring-opening followed by hydrolysis.

Alphabody conjugate (**34**) was dissolved in a 10 mM TRIS buffer at pH 7, 8 and 9, and shaken at room temperature for 6 days. The stability was checked using LC-MS.

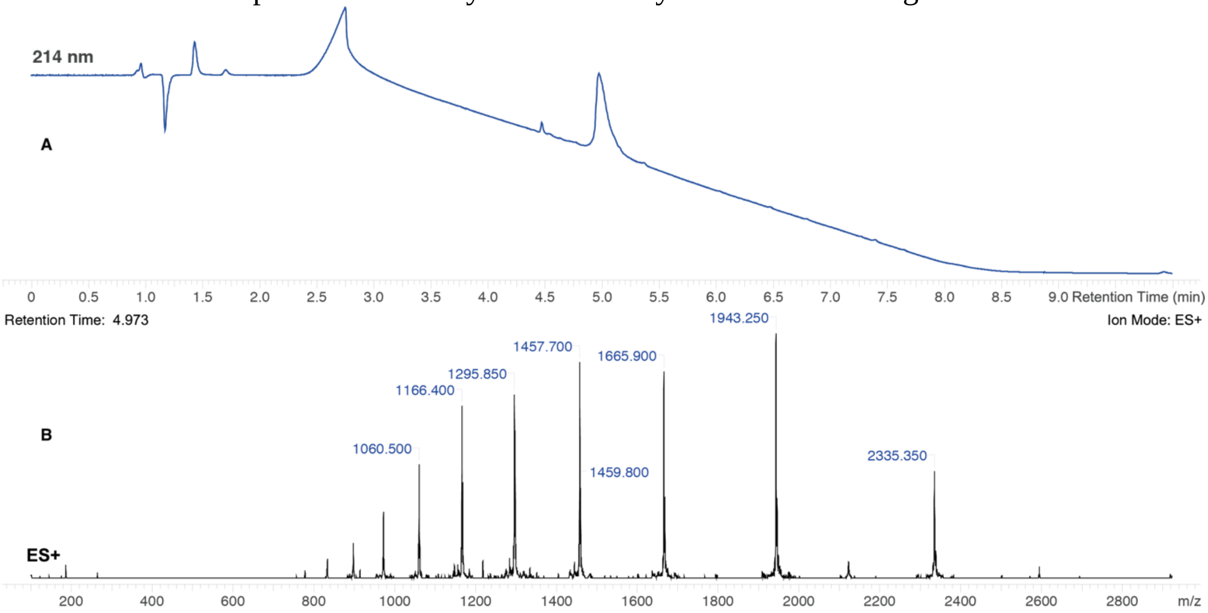

Figure S 64: LC-MS analysis of Alphabody conjugate after 6 days at pH 7. (A) Liquid chromatogram (214 nm) and (B) Mass spectrum of the Alphabody conjugate protein peak.

Deconvolution of Spectrum # 1 @ 4.991 min

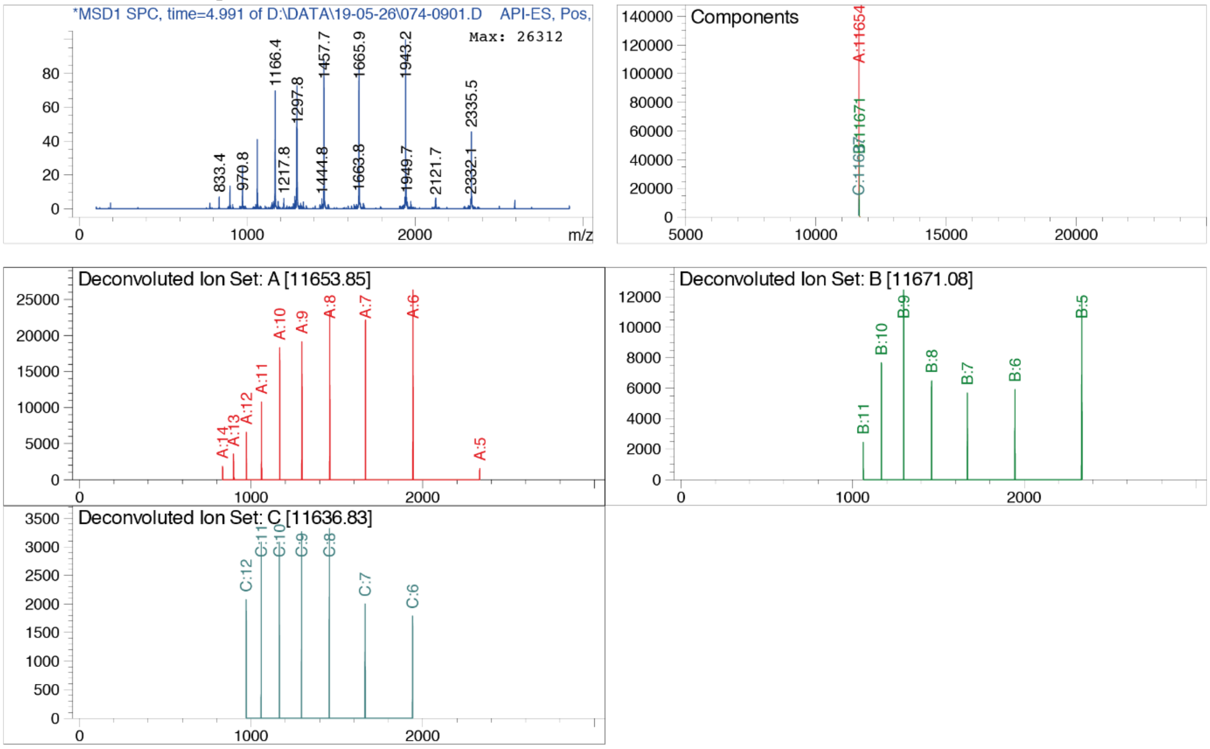

Component	Molecular Weight	Absolute Abundance	Relative Abundance
A	11653.85	132181	100.00
B	11671.08	51680	39.10
C	11636.83	17954	13.58

*** End of Report ***

Figure S 65: Deconvoluted masses of LC-MS analysis of the stability test after 6 days at pH 7. Deconvoluted masses show (A) the Alphabody conjugate $[M+H]^+$, (B) conjugate with the loss of 1 water molecule $[M+H-H_2O]^+$ and (C) conjugate with the loss of 2 water molecules $[M+H-2*H_2O]$.

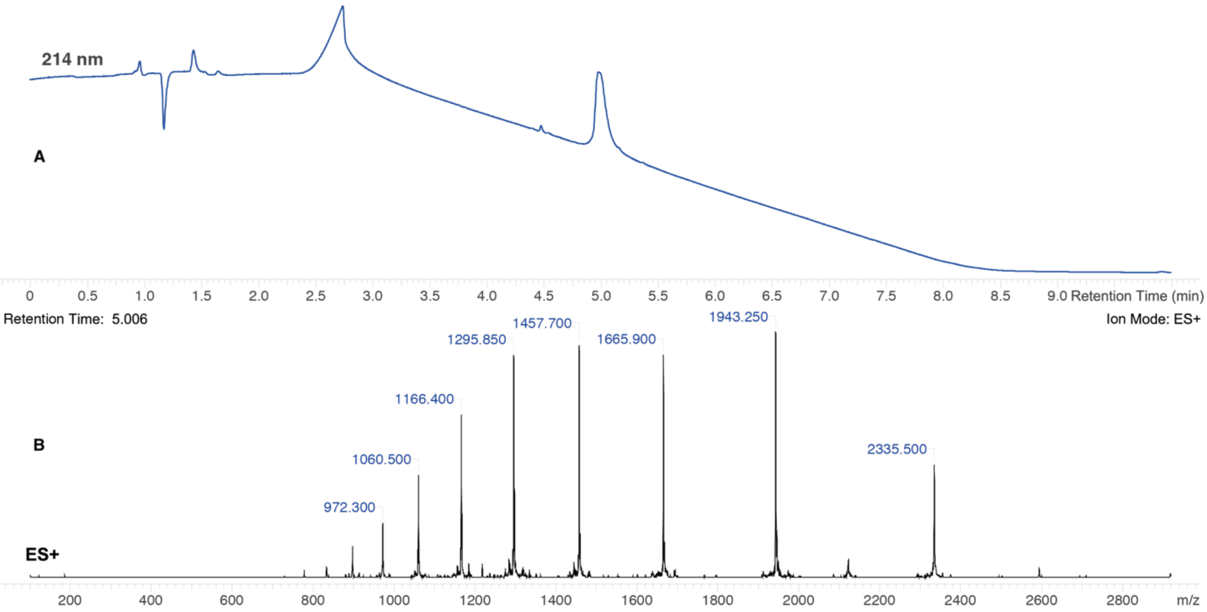

Figure S 66: LC-MS analysis of Alphabody conjugate after 6 days at pH 8. (A) Liquid chromatogram (214 nm) and (B) Mass spectrum of the Alphabody conjugate protein peak.

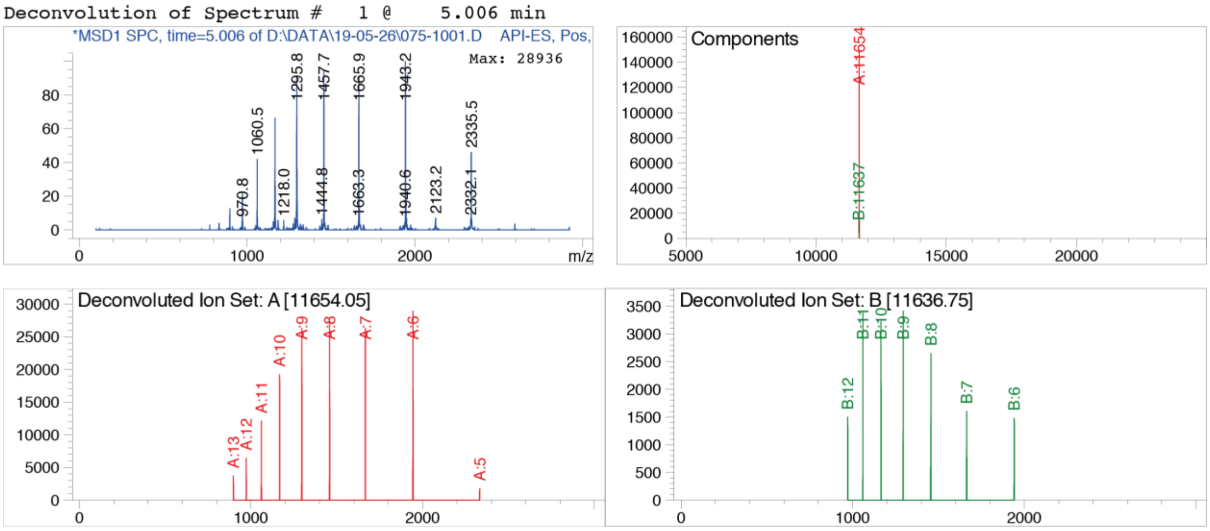

Component	Molecular Weight	Absolute Abundance	Relative Abundance
A	11654.05	150394	100.00
B	11636.75	16906	11.24

*** End of Report ***

Figure S 67: Deconvoluted masses of LC-MS analysis of the stability test after 6 days at pH 8. Deconvoluted masses show (A) the Alphabody conjugate with the loss of 1 water molecule $[M+H-H_2O]^+$ and (B) the conjugate with the loss of 2 water molecules $[M+H-2H_2O]^+$.

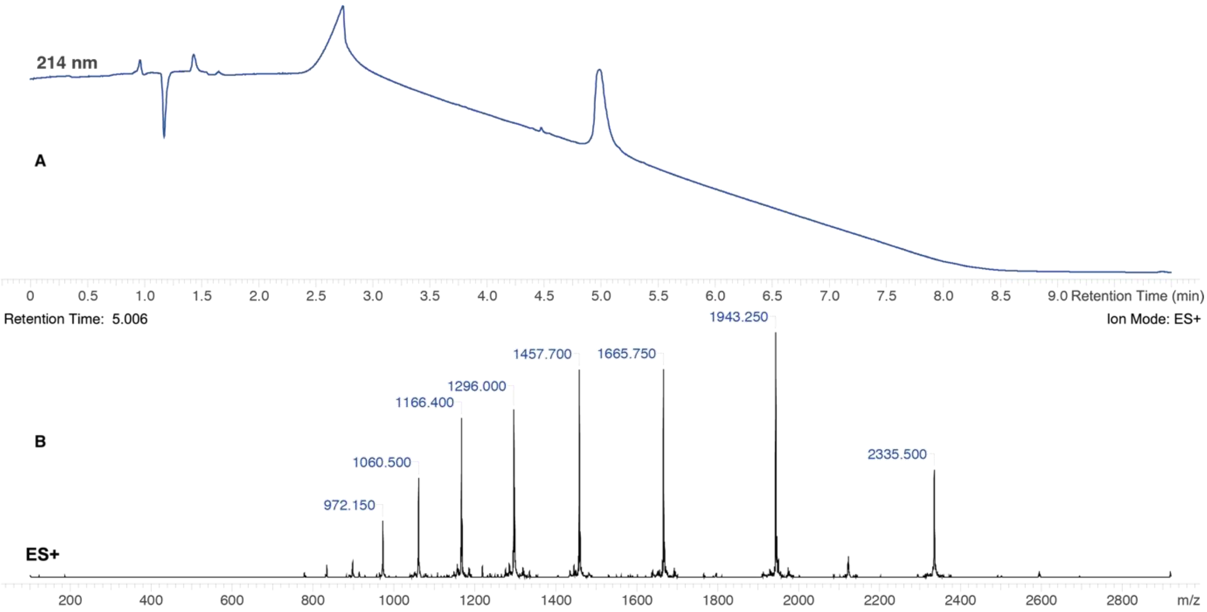

Figure S 68: LC-MS analysis of Alphabody conjugate after 6 days at pH 9. (A) Liquid chromatogram (214 nm) and (B) Mass spectrum of the Alphabody conjugate protein peak.

Deconvolution of Spectrum # 1 @ 4.987 min

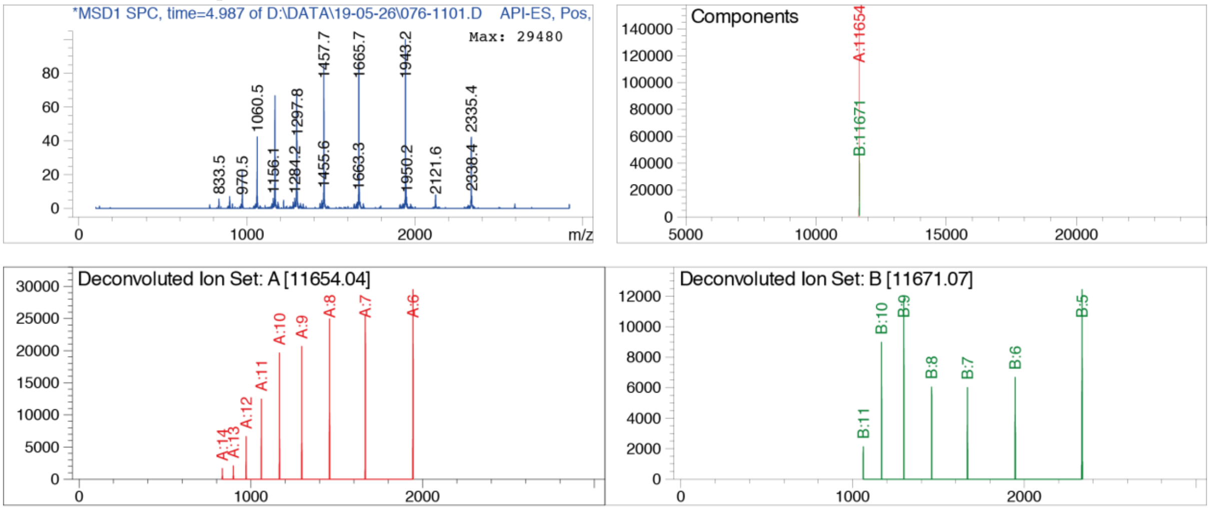

Component	Molecular Weight	Absolute Abundance	Relative Abundance
A	11654.04	141101	100.00
B	11671.07	52655	37.32

*** End of Report ***

Figure S 69: Deconvoluted masses of LC-MS analysis of the stability test after 6 days at pH 9. Deconvoluted masses show (B) the Alphabody conjugate $[M+H]^+$ and (A) the conjugate with the loss of 1 water molecule $[M+H-H_2O]^+$.

7.1.6. CuAAC reaction on Alphabody conjugate

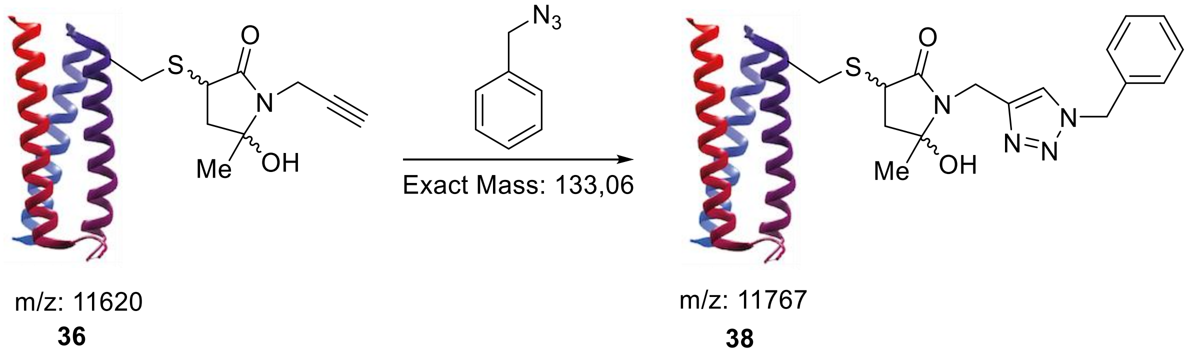

Scheme S 27: CuAAC reaction on Alphabody conjugate.

Procedure was followed according to the previously reported methodology[7].

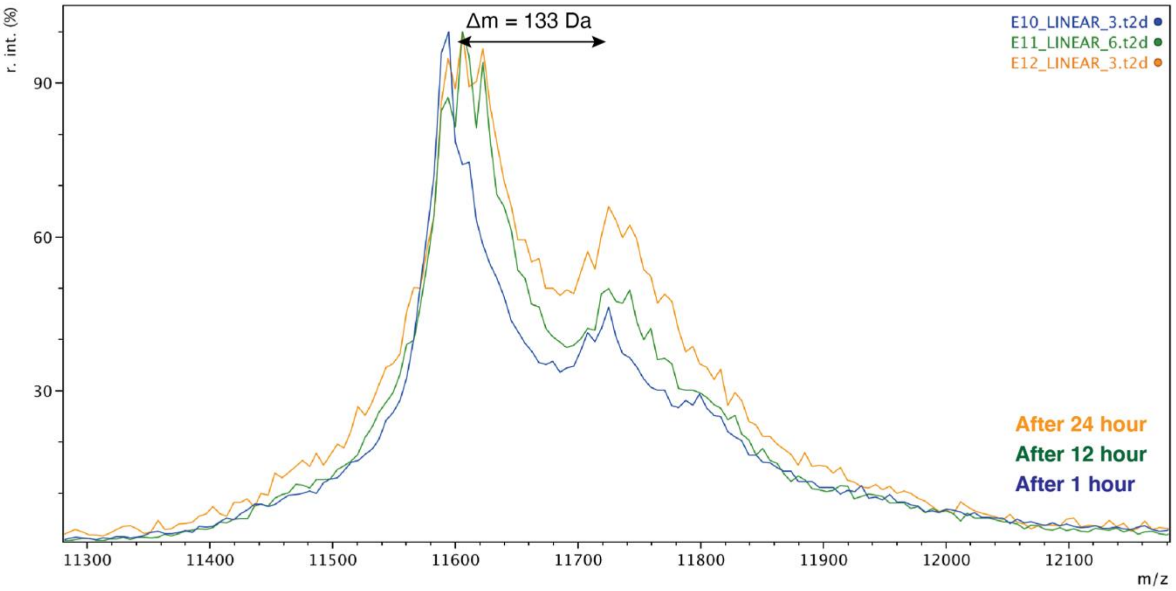

Figure S 70: Conversion to the product can be followed via MALDI-TOF analysis.

[7] V. Hong, S. I. Presolski, C. Ma, M. G. Finn, *Angew. Chem. Int. Ed.*, 2009, ***48***, 9879.

7.1.7. Glutathione competition experiment

A)

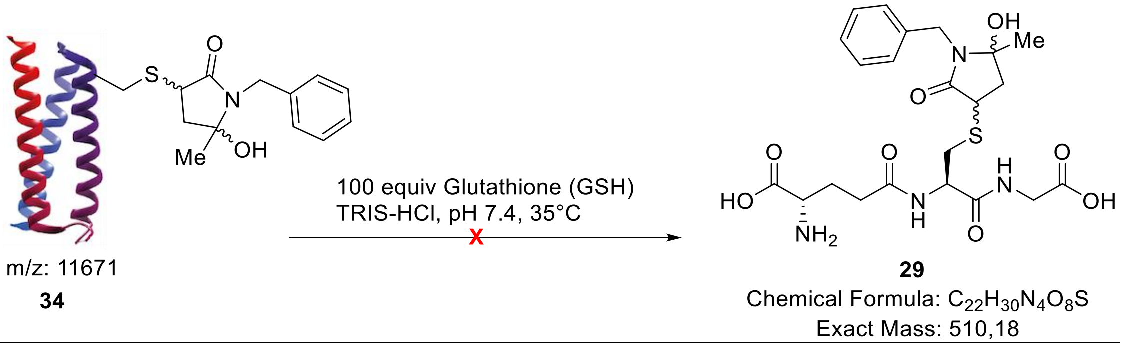

B)

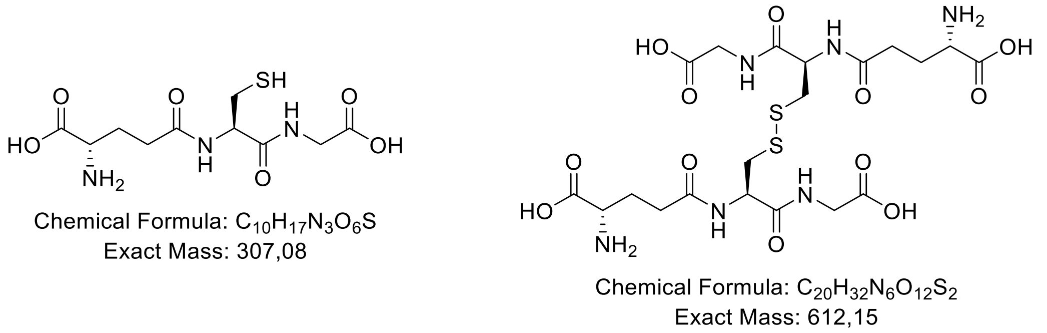

Scheme S 28: (A) Glutathione competition experiment on Alphabody conjugate and (B) Glutathione and glutathione dimer structures with corresponding mass.

Alphabody conjugate (**34**) was incubated in a 10 mM TRIS buffer at pH 7.4 with 100 equiv of glutathione, incubated at 35 °C and shaken for 5 days. The stability was checked using RP-HPLC for 55 hours. Analysis was performed on Phenomenex Luna C18 column with a flow rate of 1 mL/min. The following gradient was employed: 3 min 100% H_2O followed by 0 to 100% MeCN in 20 min and 5 min at 100% MeCN. HPLC analysis was performed up to 55 hours and is shown below.

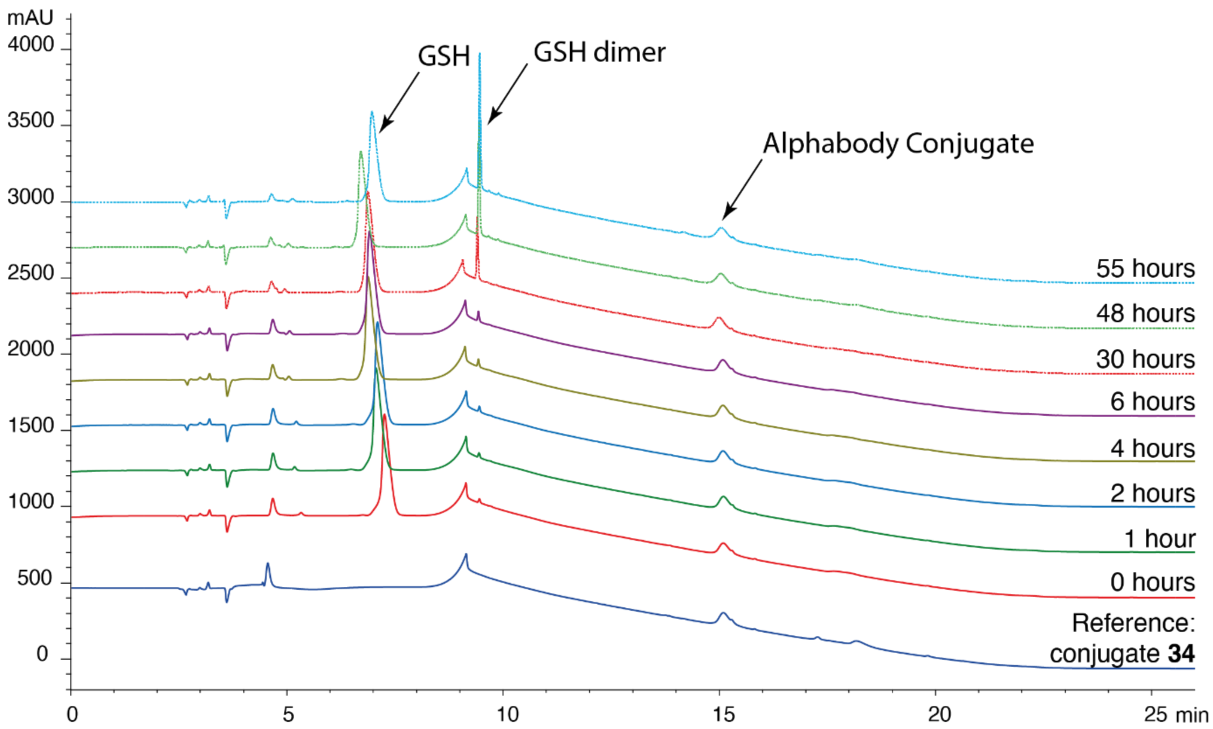

Figure S 71: Follow up of Glutathione competition experiment by RP-HPLC. LC (214 nm) traces are shown for the different time points.

Stability was further checked by LC-MS analysis after 68 hours and once more after 5 days. Corresponding chromatograms and spectra are shown below.

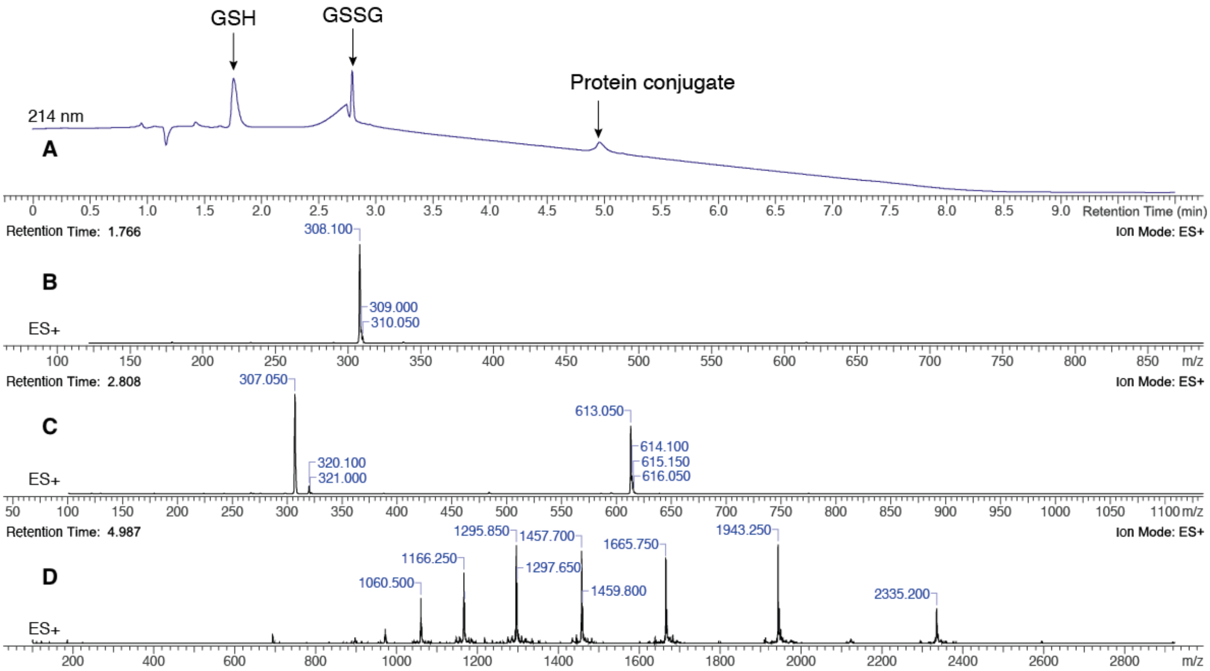

Figure S 72: LC-MS analysis of glutathione competition reaction mixture after 68 hours. (A) Liquid chromatogram (214 nm), (B) Mass spectrum of glutathione peak, (C) Mass spectrum of glutathione dimer peak (D) Mass spectrum of Alphabody protein conjugate peak.

Deconvolution of Spectrum #   1 @   4.987 min

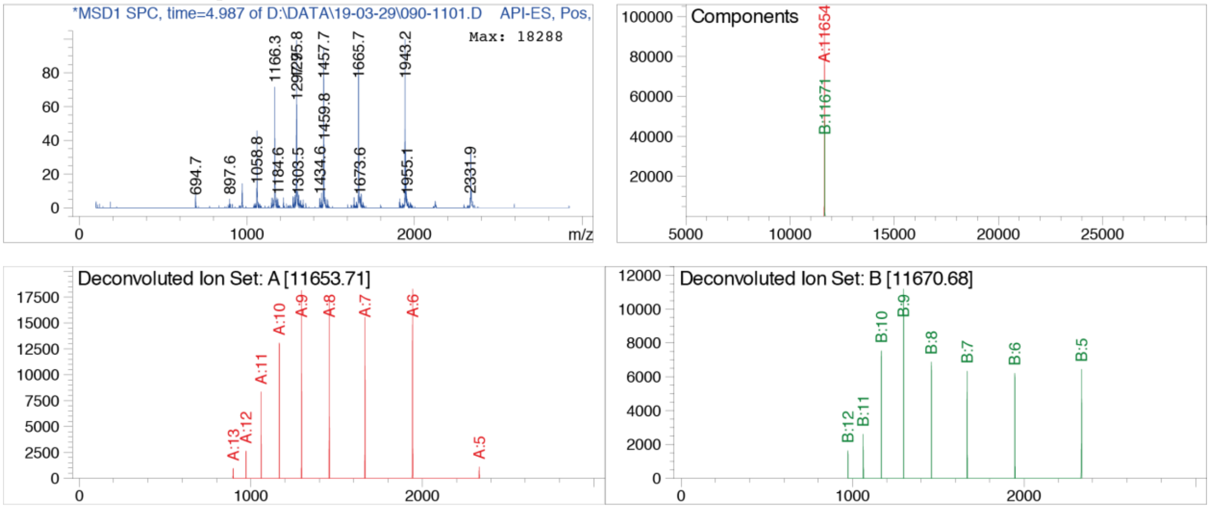

Component	Molecular Weight	Absolute Abundance	Relative Abundance
A	11653.71	94678	100.00
B	11670.68	47941	50.64

*** End of Report ***

Figure S 73: Deconvoluted masses of Alphabody conjugate peak in glutathione competition experiment after 68 hours. Deconvoluted masses show (B) the intact Alphabody conjugate $[M+H]^+$ and (A) the conjugate with the loss of 1 water molecule $[M+H-H_2O]^+$.

Deconvolution of Spectrum #   1 @   4.888 min

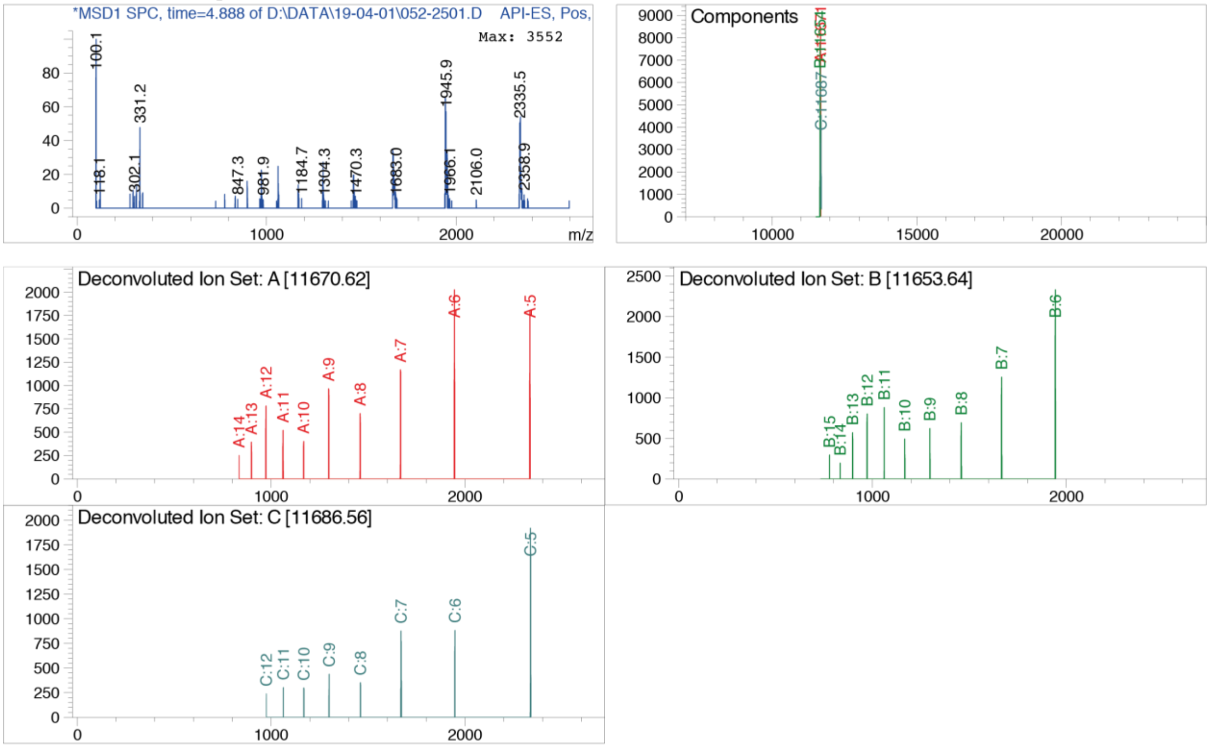

Component	Molecular Weight	Absolute Abundance	Relative Abundance
A	11670.62	8475	100.00
B	11653.64	7801	92.05
C	11686.56	4545	53.63

*** End of Report ***

Figure S 74: Deconvoluted masses of Alphabody conjugate peak in glutathione competition experiment after 5 days. Deconvoluted masses show (A) the intact Alphabody conjugate $[M+H]^+$, (B) conjugate with the loss of 1 water molecule $[M+H-H_2O]^+$ and (C) oxidation product $[M+H+O]^+$, which is often seen on methionine.

7.2. Bovine Serum Albumin (BSA) conjugation

BSA was purchased from Sigma with a 96% grade purity and analysed using LC-MS.

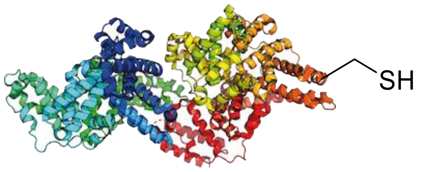

m/z = 66417

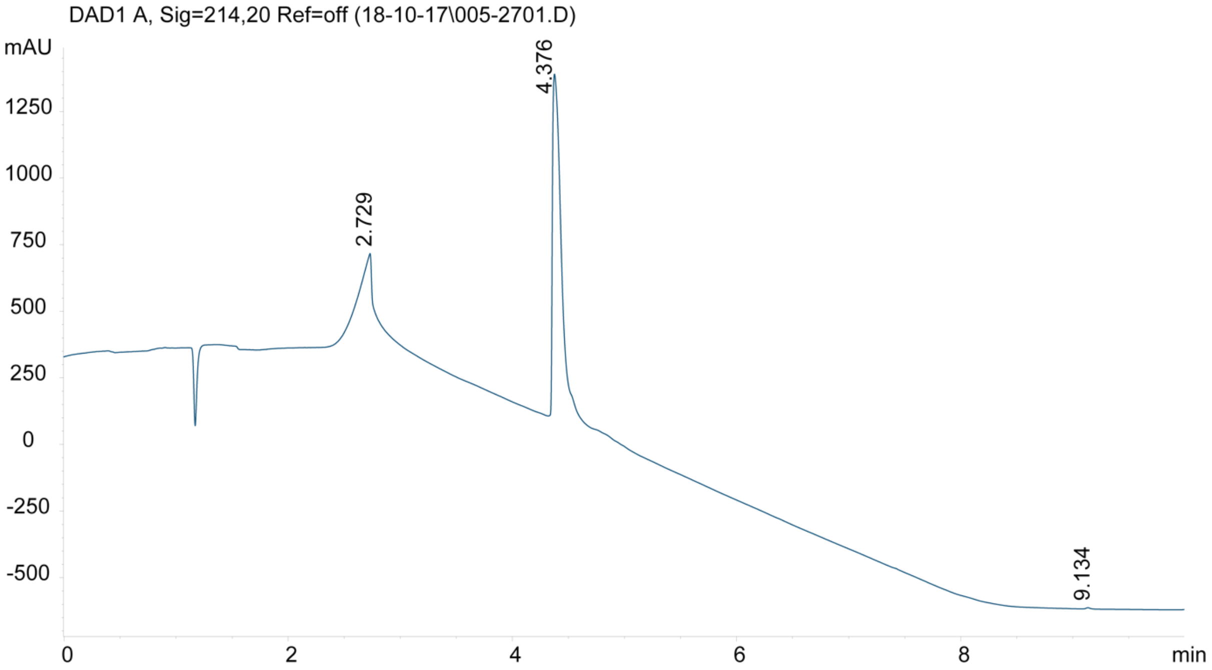

Figure S 75: LC analysis of BSA.

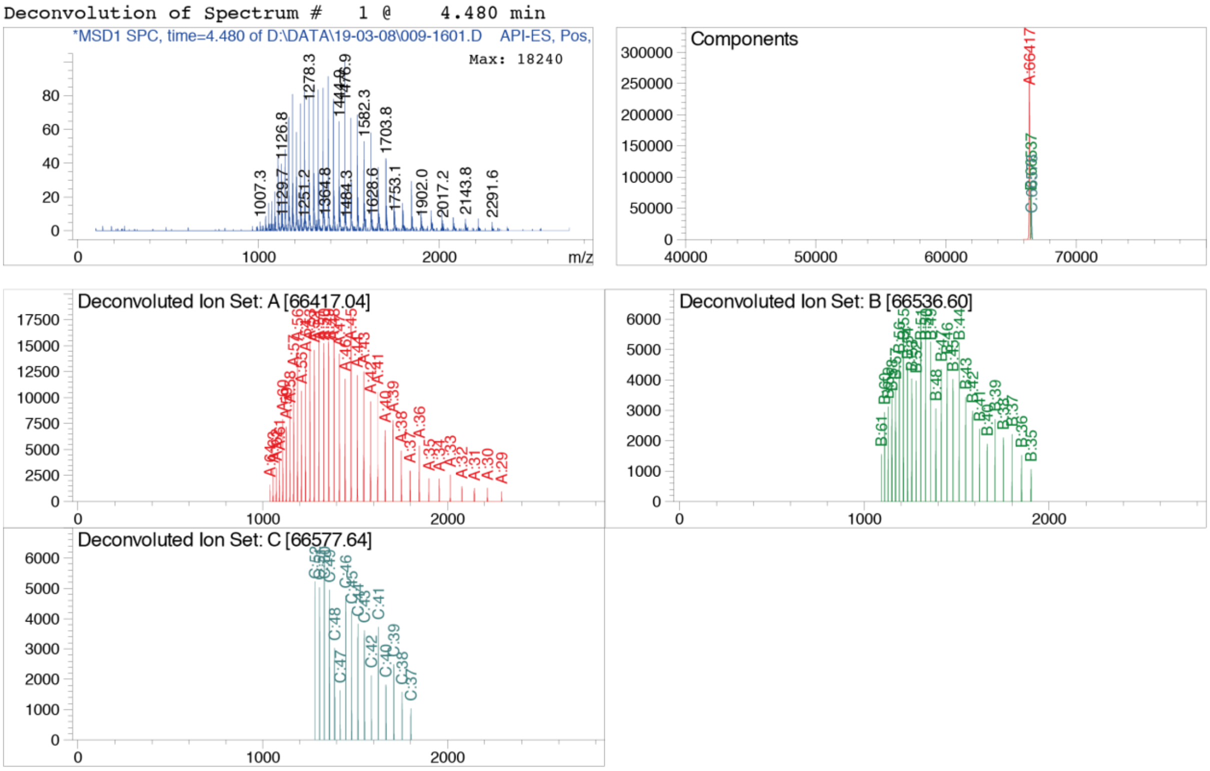

Component	Molecular Weight	Absolute Abundance	Relative Abundance
A	66417.04	303939	100.00
B	66536.60	91955	30.25
C	66577.64	49882	16.41

*** End of Report ***

Figure S 76: Deconvoluted masses of BSA protein. Main mass found: (A) 66417 Da. Other masses are the result of the purity grade.

7.2.1. Verification of free thiol functionality by reaction of BSA with Ellman's reagent

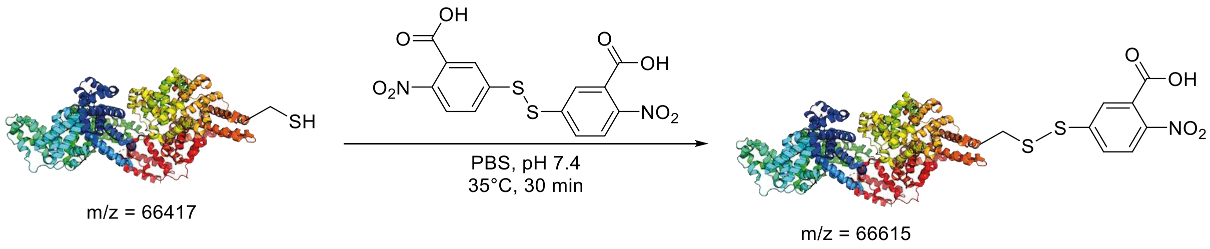

Scheme S 29: Verification of free cysteine on BSA by Ellman's reagent.

A solution of Ellman's reagent was prepared (1 mg in 400 µL PBS, pH 7.4). 11.9 µL of this solution was added to 20 µL BSA (0.1 mg, c = 5 mg/mL in PBS, pH 7.4) and shaken at 35 °C for 30 minutes. After this time, the protein was separated from excess Ellman's reagent by a MicroSpin 6 column from BioRad. Protein conjugate was analysed by LC-MS.

Deconvolution of Spectrum # 1 @ 4.461 min

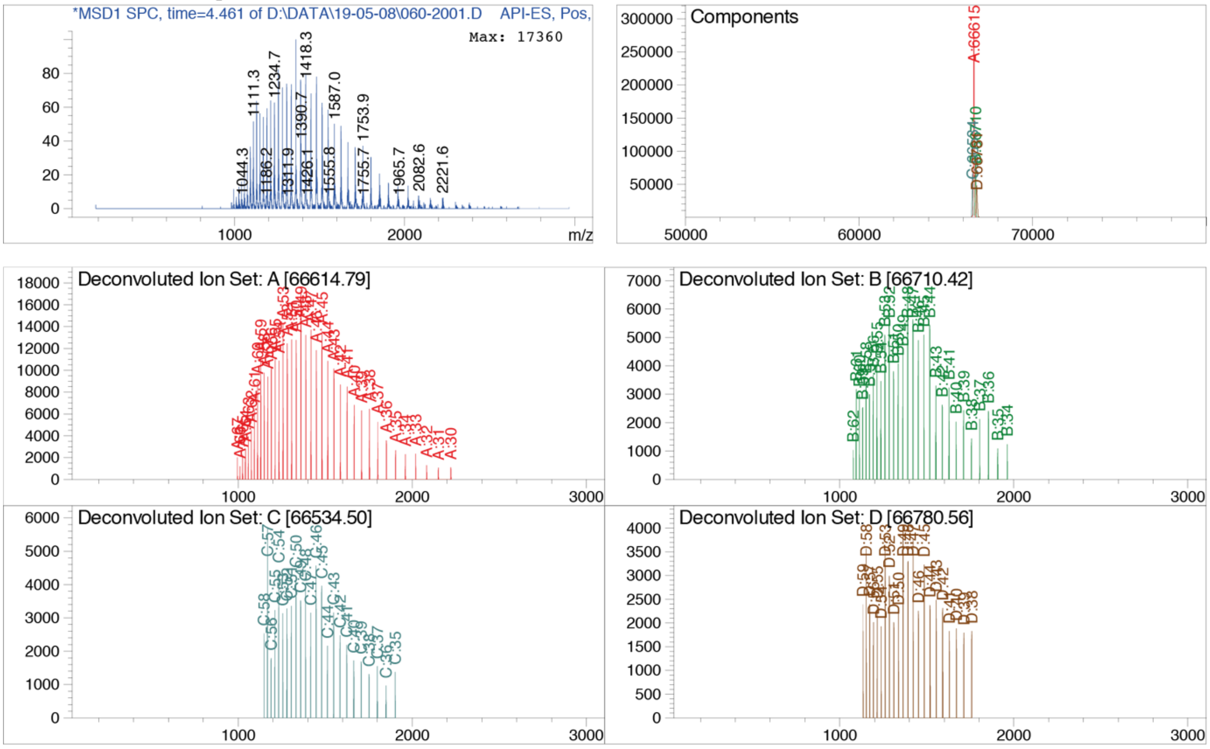

Component	Molecular Weight	Absolute Abundance	Relative Abundance
A	66614.79	286970	100.00
B	66710.42	93976	32.75
C	66534.50	66814	23.28
D	66780.56	47782	16.65

*** End of Report ***

Figure S 77: Deconvoluted masses of protein peak. Calculated mass: 66615.2 Da, main mass found: (A) 66614.8 Da.

7.2.2. BSA conjugation to 5HP2O building blocks

Conjugation to 5HP2O building block 2

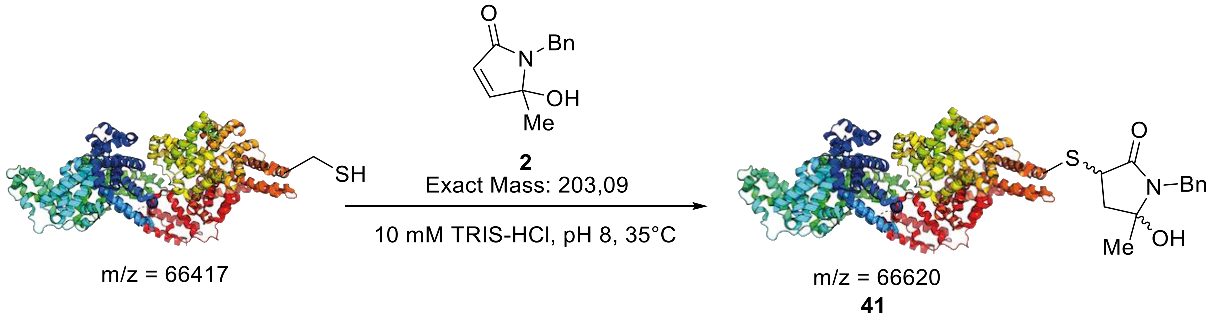

*Scheme S 30: Conjugation of BSA to 5HP2O building block **2**.*

To 207 µL of BSA (0.25 mg, 3.75 nmol, 1.21 mg/mL in 10 mM TRIS-HCl pH 8.0) was added 3.8 µL of 5HP2O building block **2** (0.152 mg, c = 40 mg/mL solution in DMSO, 0.75 µmol) and the reaction was allowed to shake overnight at 35 °C. After conjugation, the protein was separated from excess 5HP2O building block by a MicroSpin 6 column from BioRad and analysed by LC-MS.

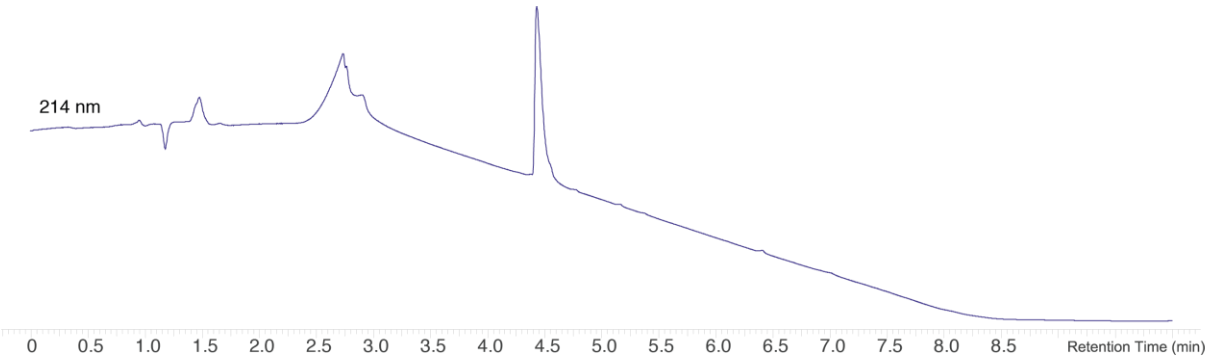

Figure S 78: LC analysis of protein peak of conjugation reaction mixture.

Deconvolution of Spectrum # 1 @ 4.380 min

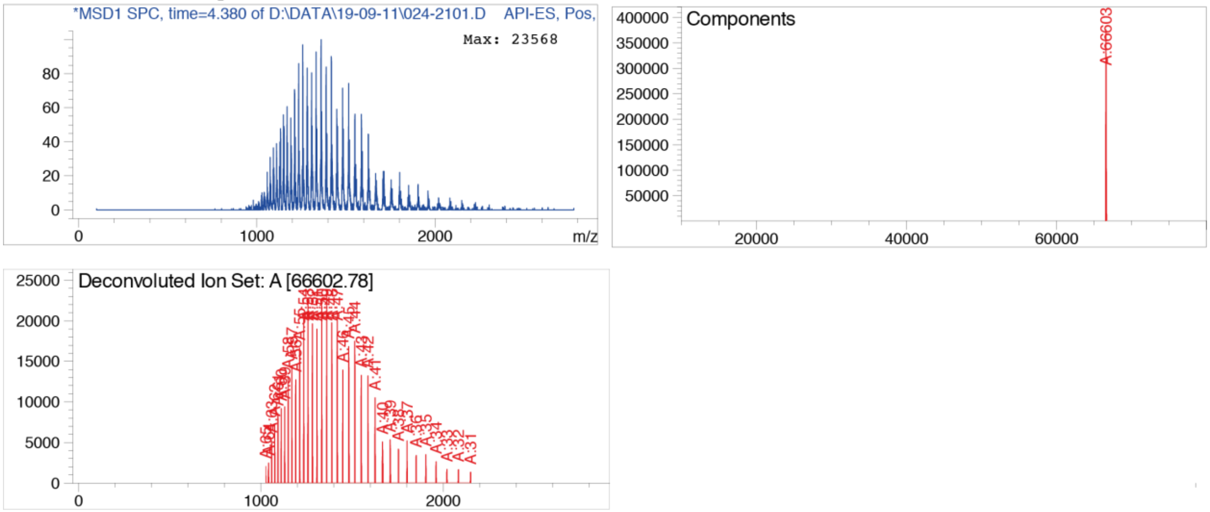

Component	Molecular Weight	Absolute Abundance	Relative Abundance
A	66602.78	375878	100.00

Figure S 79: Deconvoluted mass of protein peak of the BSA conjugation reaction mixture. Calculated mass: 66620 Da. Mass found: (A) 66602.8 Da $[M+H-H_2O]^+$.

Conjugation to 5HP2O building block 7

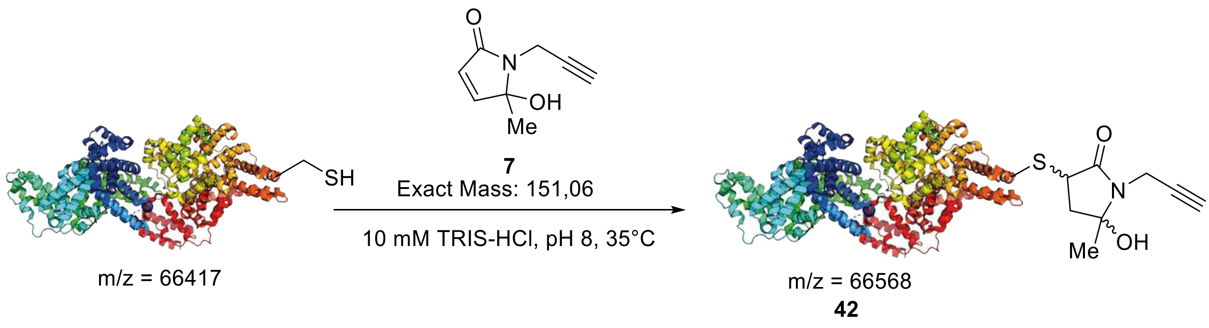

*Scheme S 31: Conjugation of BSA to 5HP2O building block **7**.*

To 125 µL of BSA (0.5 mg, 7.52 nmol, 4 mg/mL in 10 mM TRIS-HCl pH 8.0) was added 3.8 µL of 5HP2O building block **7** (0.114 mg, c = 30 mg/mL solution in DMSO, 0.75 µmol) and the reaction was allowed to shake overnight at 35 °C. After conjugation, the protein was separated from excess 5HP2O building block by a MicroSpin 6 column from BioRad and analysed by LC-MS.

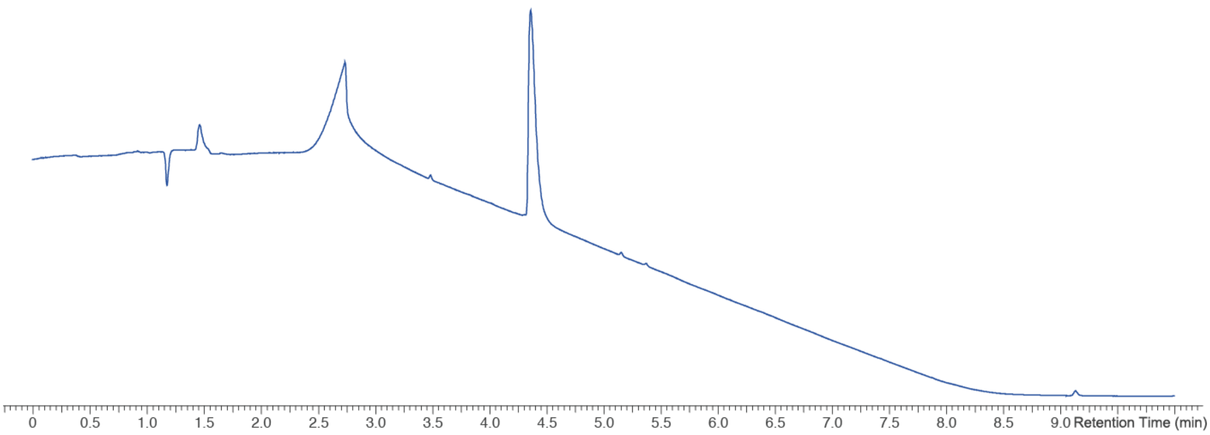

Figure S 80: LC analysis of BSA conjugation reaction mixture.

```
Deconvolution of Spectrum #   1 @    4.380 min
```

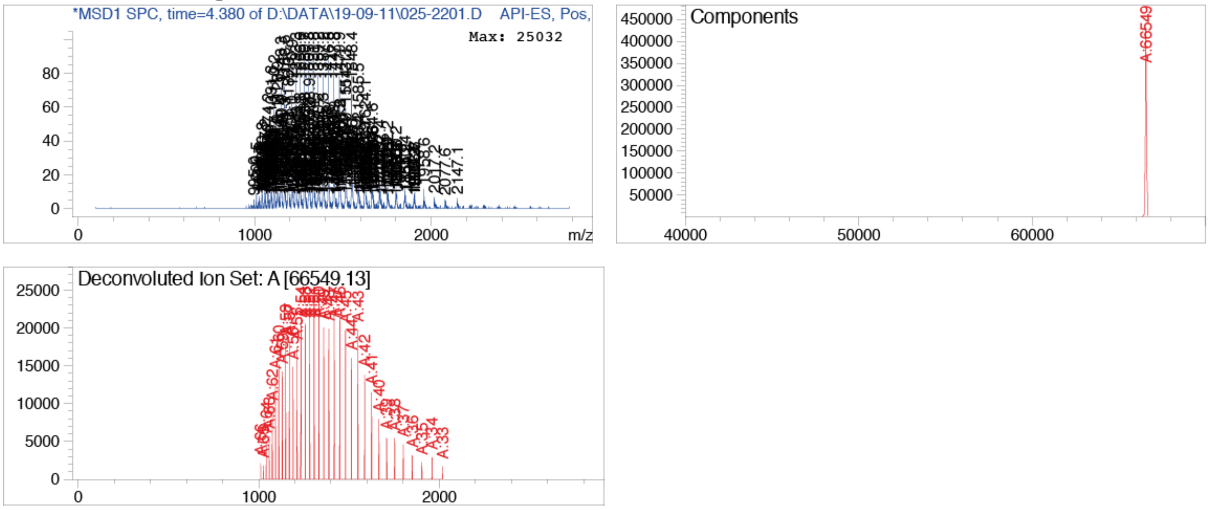

Component	Molecular Weight	Absolute Abundance	Relative Abundance
A	66549.13	431436	100.00

*** End of Report ***

Figure S 81: Deconvoluted mass of protein peak of the BSA conjugation reaction mixture. Calculated mass: 66568 Da. Mass found: (A) 66549 Da [M+H-H_2O]$^+$.

8. Reactivity of 5HP2O building blocks towards Trastuzumab antibody

Trastuzumab-Herceptin® was obtained from Genentech/Roche

8.1. Trastuzumab reduction

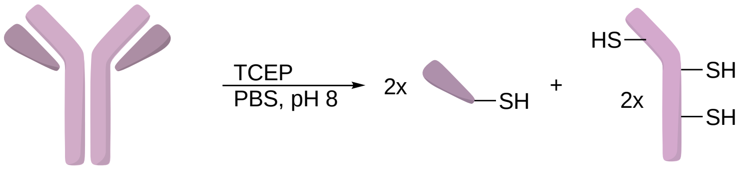

Scheme S 32: TCEP reduction of Trastuzumab antibody.

TCEP-HCl (1.6 µL out of a 1 mg/mL stock solution in H_2O, 0.0056 µmol, 20 equiv) was added to 88 µL of Trastuzumab (0,044 mg, c = 0.5 mg/mL in 11.9 mM PBS buffer at pH 8.0, 137 mM NaCl, 2.7 mM KCl) and shaken at 37 °C for 40 min. After this time, the protein was separated from TCEP-HCl and buffer exchanged into PBS pH 8 by means of a Micro BioSpin 6 column (Bio-Rad). Reduced Trastuzumab was analysed by LC-MS, the associated spectra are shown below. Due to glycosylation, dehydration and oxidation of the protein, multiple variants of the heavy-chain are observed (Figure S53 and S54). Only the most abundant masses are shown below in the conjugation reactions.

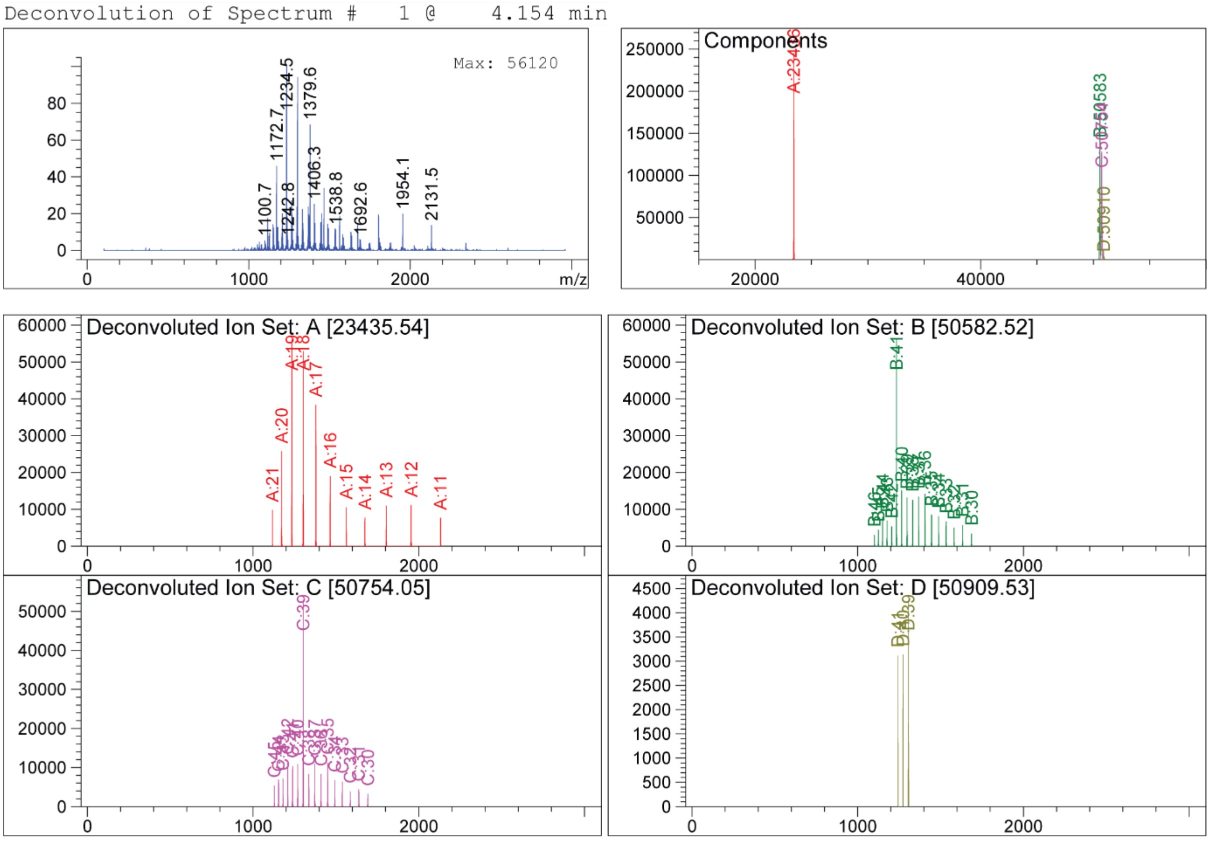

	Weight	Abundance	Abundance
A	23435.54	245558	100.00
B	50582.52	170307	69.36
C	50754.05	128139	52.18
D	50909.53	10341	4.21

Figure S 82: Deconvoluted masses of reduced Trastuzumab antibody: (A) Light chain and (B-D) heavy chain.

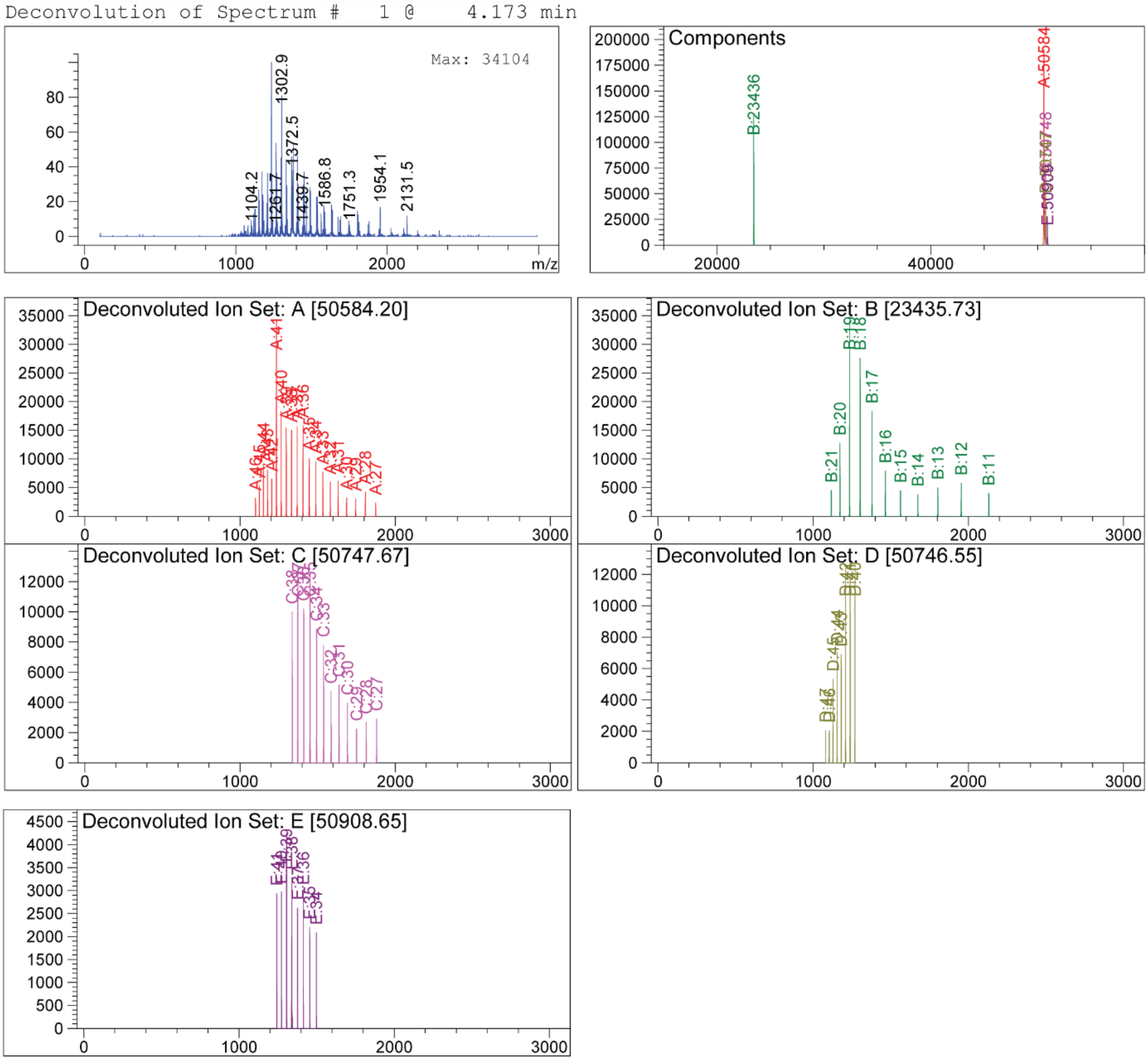

Component	Molecular Weight	Absolute Abundance	Relative Abundance
A	50584.20	190223	100.00
B	23435.73	125750	66.11
C	50747.67	81639	42.92
D	50746.55	58746	30.88
E	50908.65	22462	11.81

Figure S 83: Deconvoluted masses of reduced Trastuzumab antibody: (B) Light chain and (A,C-E) heavy chain.

8.2. Trastuzumab conjugation to 5HP2O building block 2

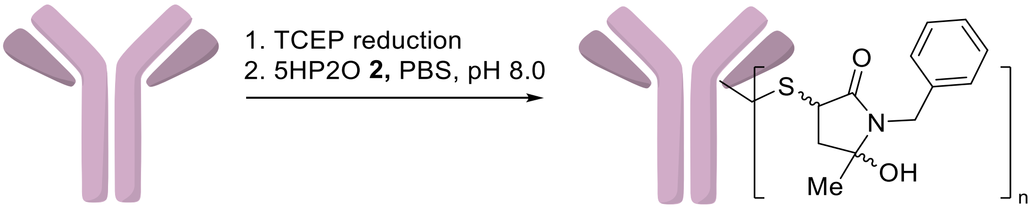

43

*Scheme S 33: Conjugation of Trastuzumab to 5HP2O building block **2**.*

Trastuzumab was first reduced with TCEP following the procedure described above. To 88 μL of reduced Trastuzumab (0.044 mg, 0.29 nmol, c = 0.5 mg/mL in PBS, pH 8) 5HP2O building block **2** (0.142 μL from 40 mg/mL solution in DMSO, 0.028 μmol, 100 equiv) was added and the reaction was allowed to shake at 25 °C overnight. After conjugation, the Trastuzumab was separated from excess 5HP2O building block by a MicroSpin 6 column from BioRad and analysed by LC-MS.

Deconvolution of Spectrum # 1 @ 4.192 min

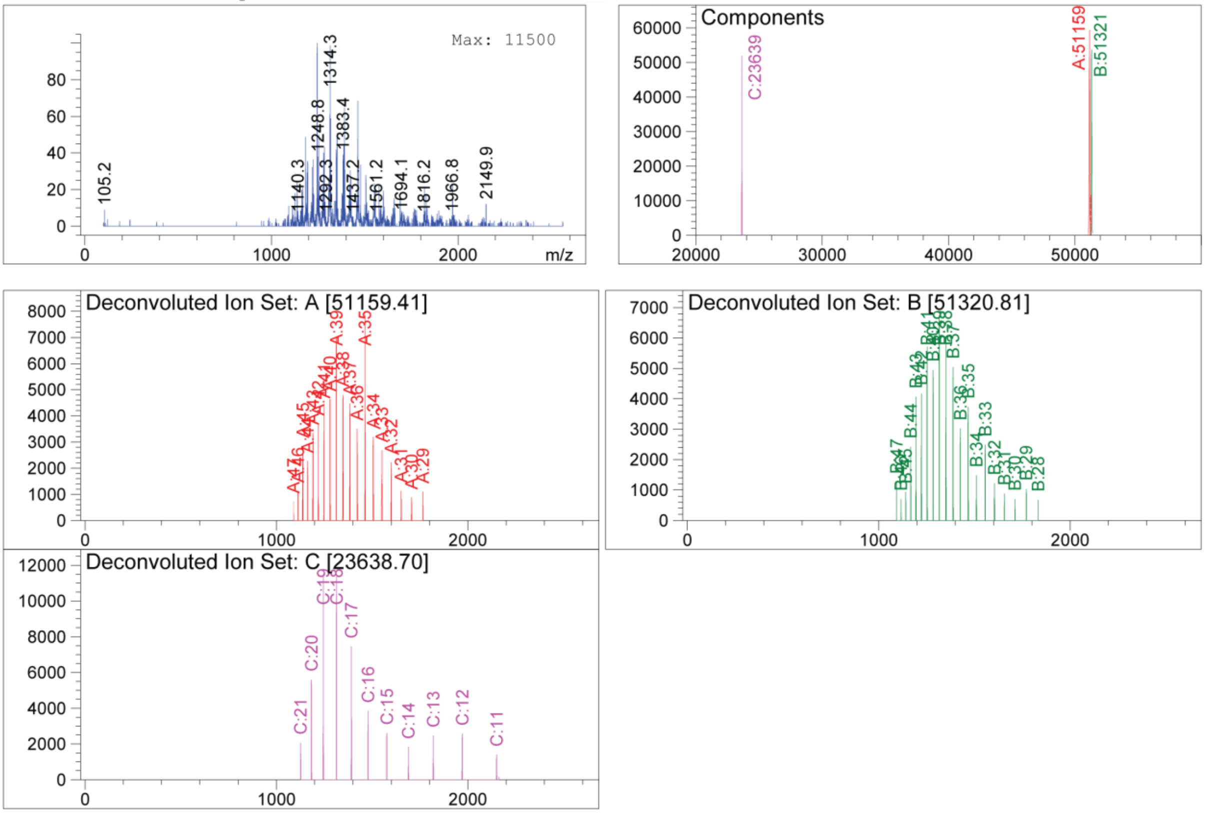

Component	Molecular Weight	Absolute Abundance	Relative Abundance
A	51159.41	59475	100.00
B	51320.81	53801	90.46
C	23638.70	51949	87.35

Figure S 84: Deconvoluted masses of conjugated Trastuzumab. Observed masses: (A) 51159 Da (Heavy-chain conjugated to two 5HP2O building blocks), (B) 51321 Da (Heavy-chain conjugated to three building blocks with the loss of two waters) (C) 23638 Da (light-chain conjugate).

8.3. Trastuzumab conjugation to 5HP2O building block 3

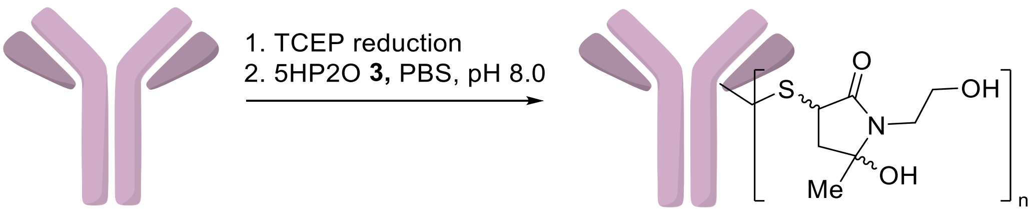

44

*Scheme S 34: Conjugation of Trastuzumab to 5HP2O building block **3**.*

Trastuzumab was first reduced with TCEP following the procedure described above. To 88 μL of reduced Trastuzumab (0.044 mg, 0.29 nmol, c = 0.5 mg/mL in PBS, pH 8) was added

5HP2O building block **3** (0.51 µL from 9 mg/mL solution in 10% DMSO/H_2O, 0.029 µmol, 100 eq.), and the reaction was allowed to shake at 25 °C overnight. After conjugation, the Trastuzumab was separated from excess 5HP2O building block by a MicroSpin 6 column from BioRad and analysed by LC-MS.

Deconvolution of Spectrum # 1 @ 4.154 min

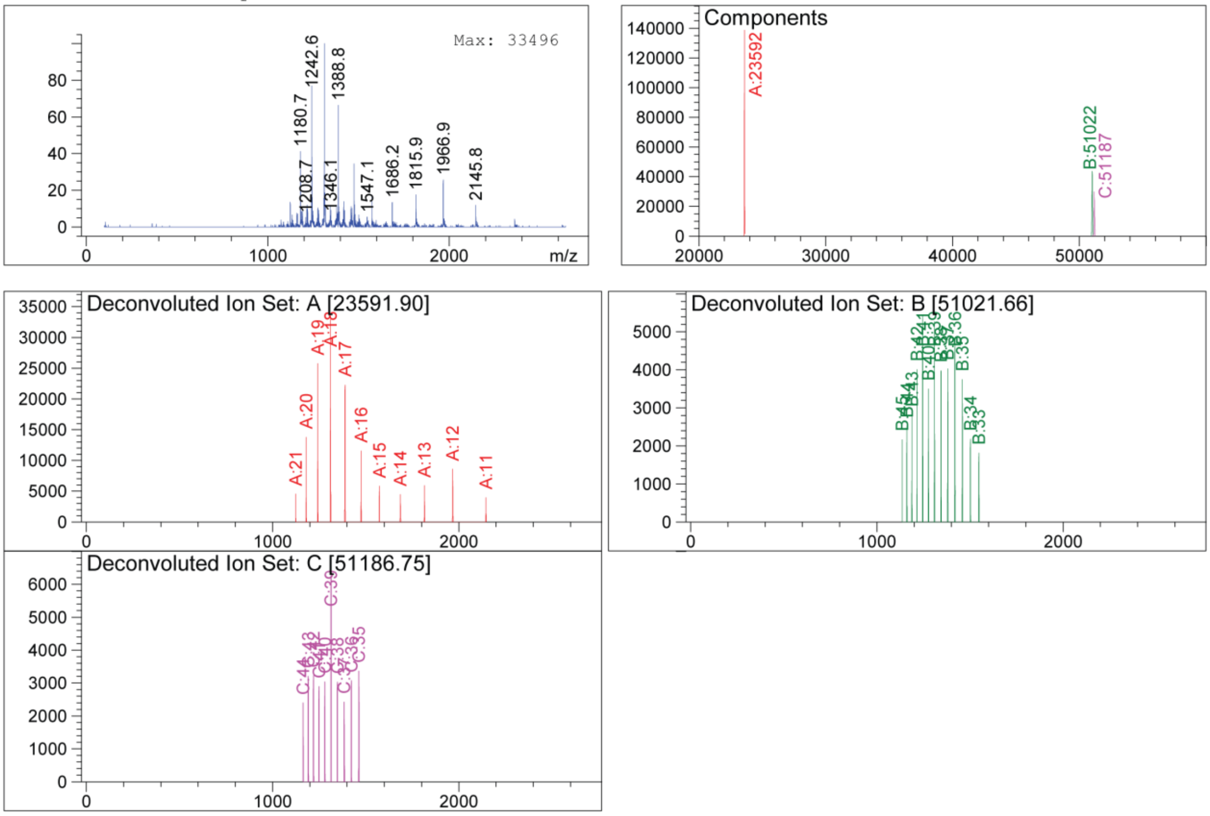

Component	Molecular Weight	Absolute Abundance	Relative Abundance
A	23591.90	138743	100.00
B	51021.66	43769	31.55
C	51186.75	29844	21.51

Figure S 85: Deconvoluted masses of Trastuzumab light- and heavy chain conjugate. Observed masses: (A) 23592 Da (light-chain conjugate), (B) 51022 Da (Heavy-chain conjugate with 2 conjugated 5HP2O building blocks and loss of two water molecules) and (C) 51187 Da (Heavy-chain conjugate with 3 conjugated 5HP2O building blocks and loss of two water molecules).

8.4. Trastuzumab conjugation to 5HP2O building block 7

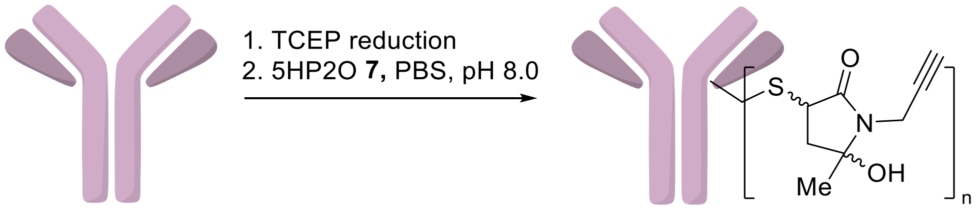

*Scheme S 35: Conjugation of Trastuzumab to 5HP2O building block **7***

Trastuzumab was first reduced with TCEP following the procedure described above. To 88 µL of reduced Trastuzumab (0.044 mg, 0.29 nmol, c = 0.5 mg/mL in PBS, pH 8) was added 5HP2O building block **7** (0.30 µL from 30 mg/mL solution in DMSO, 0.058 µmol, 200 eq.), and the reaction was allowed to shake at 25 °C overnight. After conjugation, the Trastuzumab was separated from excess 5HP2O building block by a MicroSpin 6 column from BioRad and analysed by LC-MS.

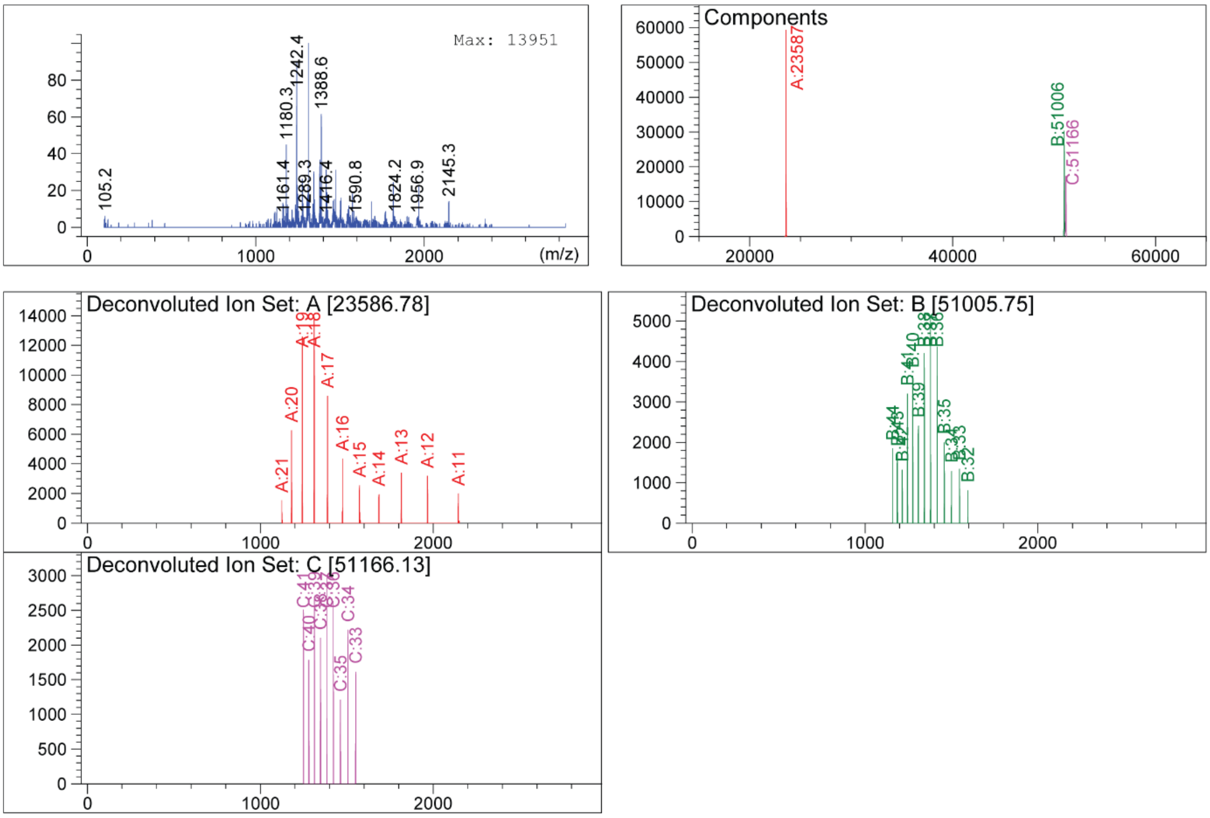

	Weight	Abundance	Abundance
A	23586.78	59362	100.00
B	51005.75	30258	50.97
C	51166.13	17310	29.16

Figure S 86: Deconvoluted masses of Trastuzumab light- and heavy chain conjugates. Observed masses: (A) 23587 Da (light-chain conjugate), (B) 51006 Da (Heavy-chain conjugate with 2 conjugated 5HP2O building blocks and loss of two water molecules) and (C) 51166 Da (Heavy-chain conjugate with 3 conjugated 5HP2O building blocks and loss of two water molecules).

8.5. Trastuzumab conjugation to 5HP2O building block 9

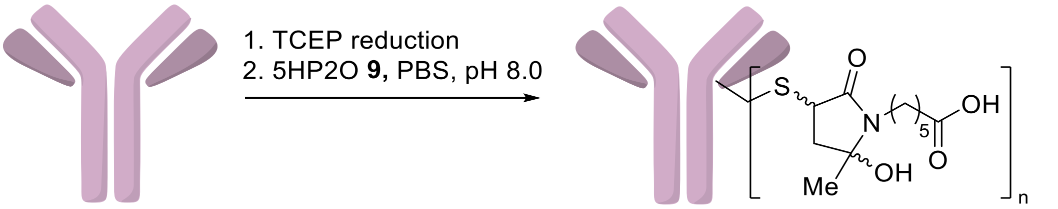

*Scheme S 36: Conjugation of Trastuzumab to 5HP2O building block **9**.*

Trastuzumab was first reduced with TCEP following the procedure described above. To 88 µL of reduced Trastuzumab (0.044 mg, 0.29 nmol, c = 0.5 mg/mL in PBS, pH 8) was added 5HP2O building block **9** (0.80 µL from 16.66 mg/mL solution in 20% DMSO/H_2O, 0.058 µmol, 200 eq.), and the reaction was allowed to shake at 25 °C overnight. After conjugation, the Trastuzumab was separated from excess 5HP2O building block by a MicroSpin 6 column from BioRad and analysed by LC-MS.

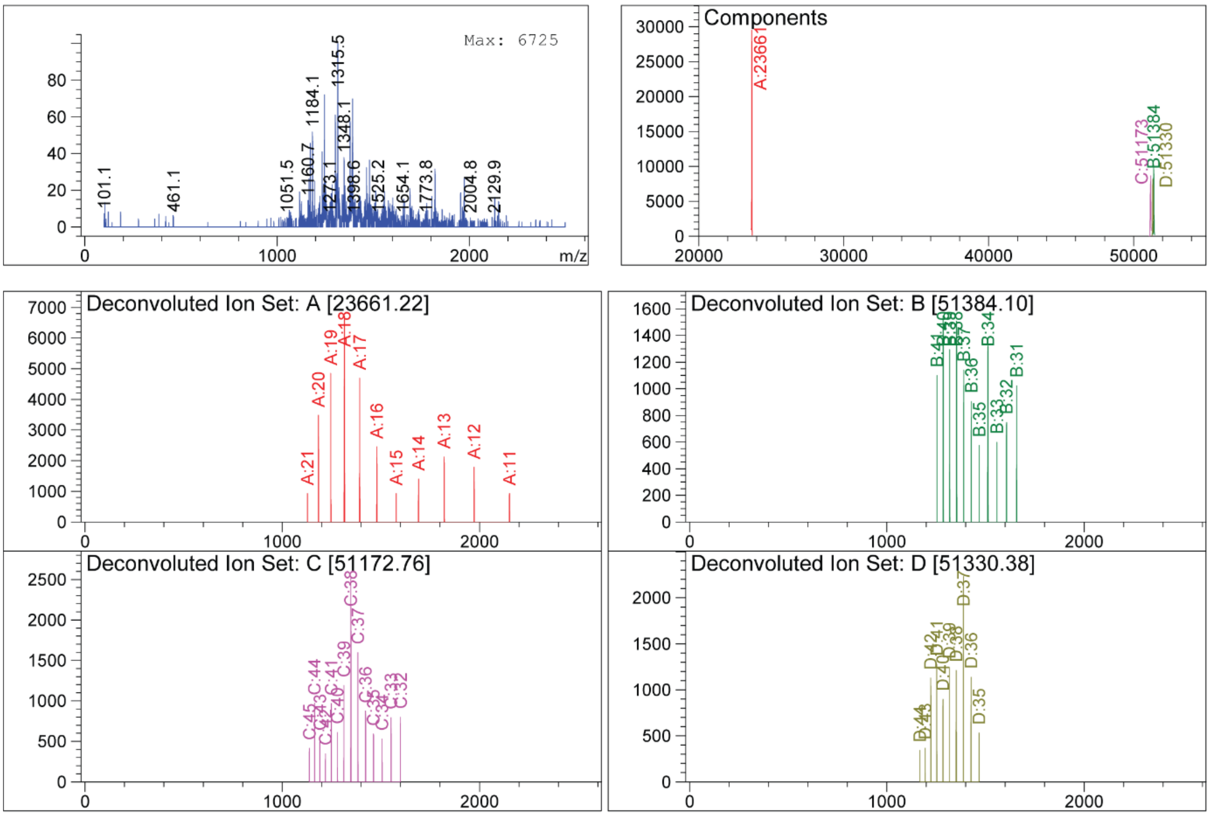

Component	Molecular Weight	Absolute Abundance	Relative Abundance
A	23661.22	29523	100.00
B	51384.10	10222	34.62
C	51172.76	8687	29.42
D	51330.38	8141	27.58

Figure S 87: Deconvoluted masses of Trastuzumab light- and heavy chain conjugates. Observed masses: (A) 235661 Da (light-chain conjugate), (B) 51384 Da (Heavy-chain conjugate with 3 conjugated 5HP2O building blocks and loss of three water molecules), (C) 51172 Da (Heavy-chain conjugate with 2 conjugated 5HP2O building blocks and loss of two water molecules) and (D) 51330 Da (Heavy-chain conjugate with 3 conjugated 5HP2O building blocks and loss of six water molecules due to the carboxylic functionality).

8.6. Trastuzumab conjugation to 5HP2O building block 11

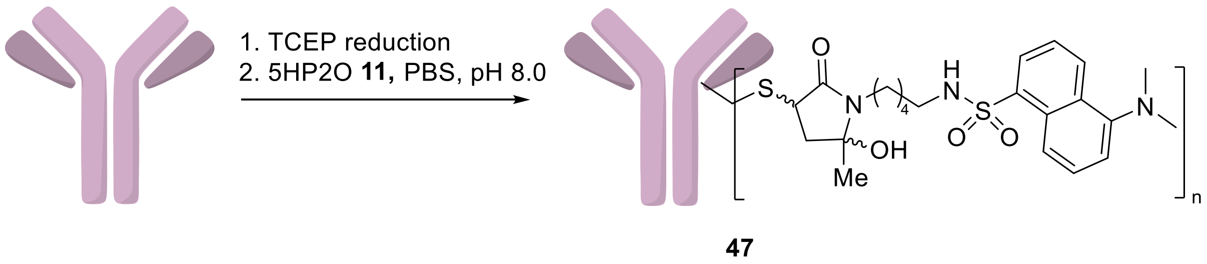

*Scheme S 37: Conjugation of Trastuzumab to 5HP2O building block **11**.*

Trastuzumab was first reduced with TCEP following the procedure described above. To 88 µL of reduced Trastuzumab (0.044 mg, 0.29 nmol, c = 0.5 mg/mL in PBS, pH 8) was added 5HP2O building block **11** (0.63 µL from 20 mg/mL solution in DMSO, 0.029 µmol, 100 eq.), and the reaction was allowed to shake at 25 °C overnight. After conjugation, the Trastuzumab was separated from excess 5HP2O building block by a MicroSpin 6 column from BioRad and analysed by LC-MS.

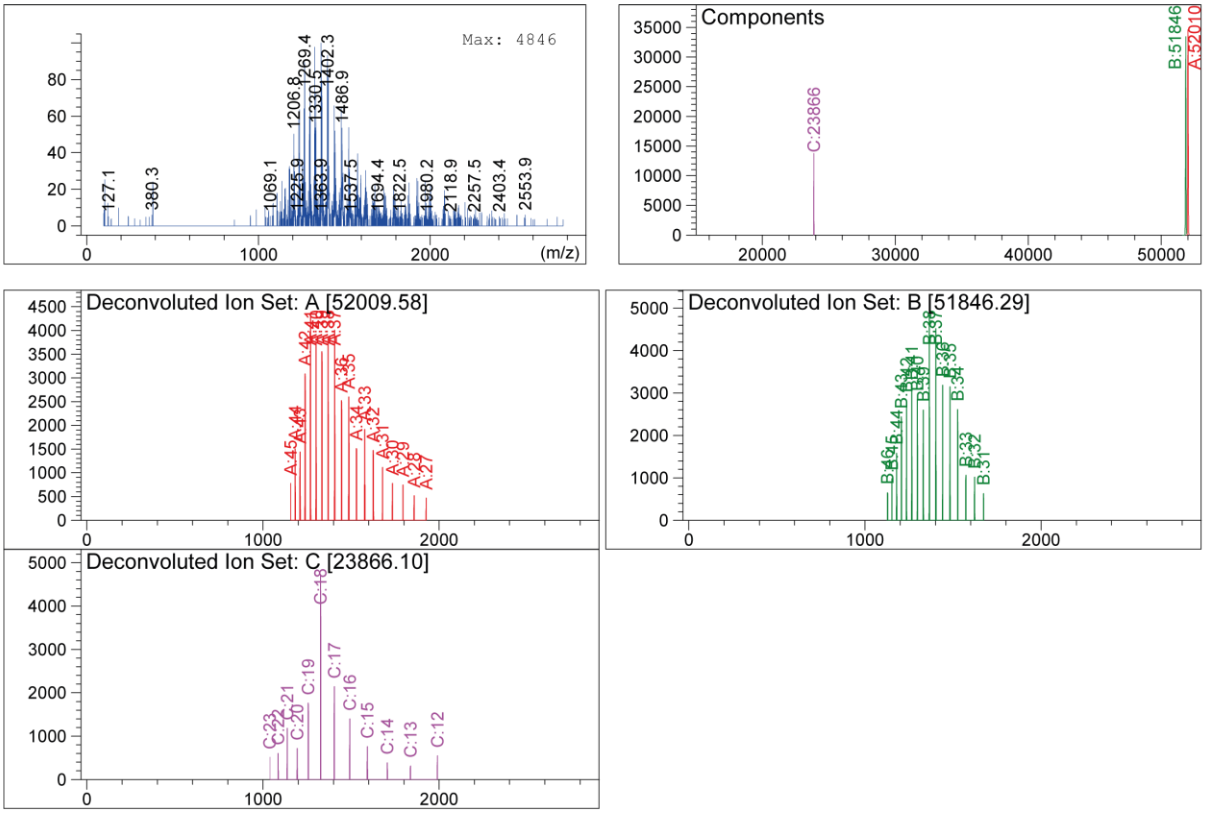

	Weight	Abundance	Abundance
A	52009.58	34658	100.00
B	51846.29	33506	96.68
C	23866.10	13780	39.76

Figure S 88: Deconvoluted masses of Trastuzumab light- and heavy chain conjugates. Observed masses: (A) 52010 Da (Heavy-chain conjugate with 3 conjugated 5HP2O building blocks and loss of two water molecules)), (B) 51846 Da (Heavy-chain conjugate with 3 conjugated 5HP2O building blocks and loss of two water molecules) and (C) 23866 Da (light-chain conjugate).

9. Copies of NMR spectra

Compound 1d

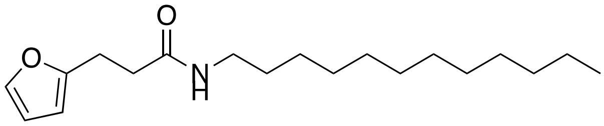

(400 MHz, $CDCl_3$)

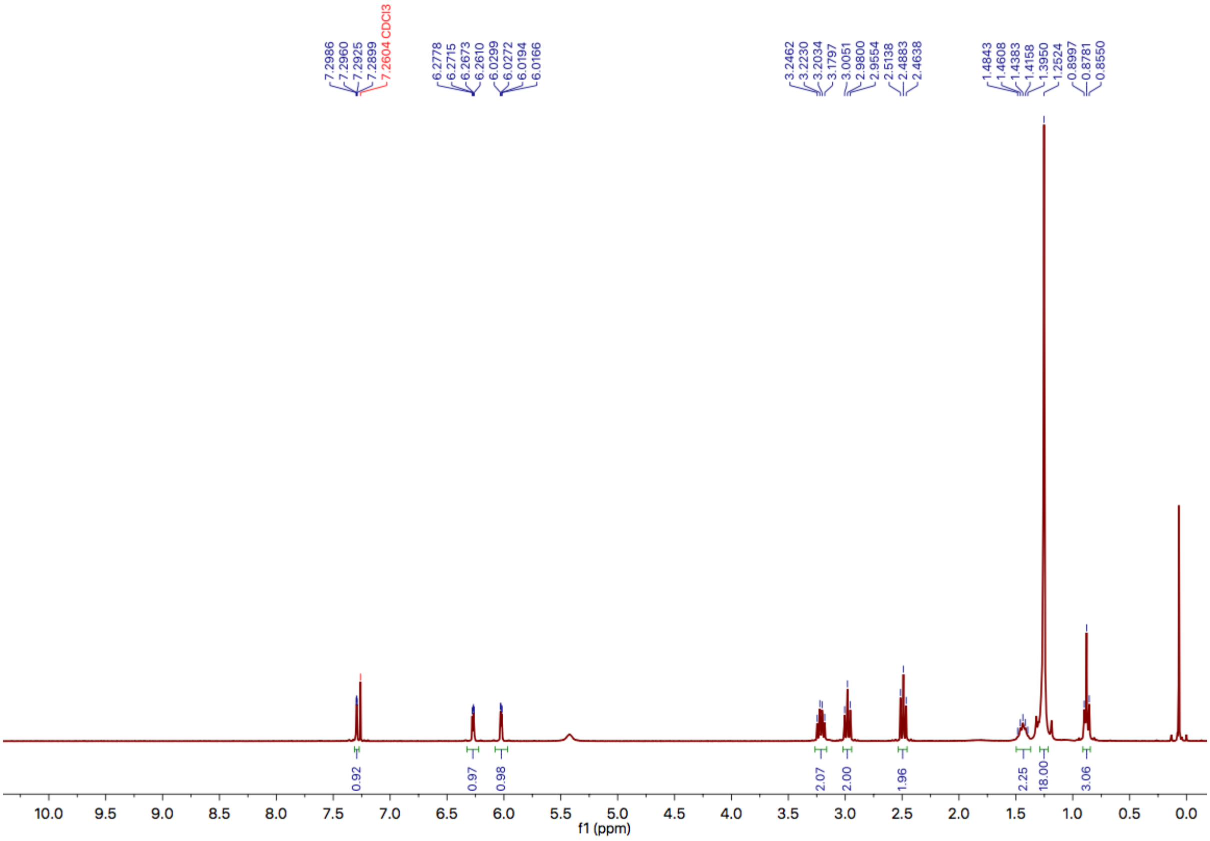

(^{13}C-APT,101 MHz, $CDCl_3$)

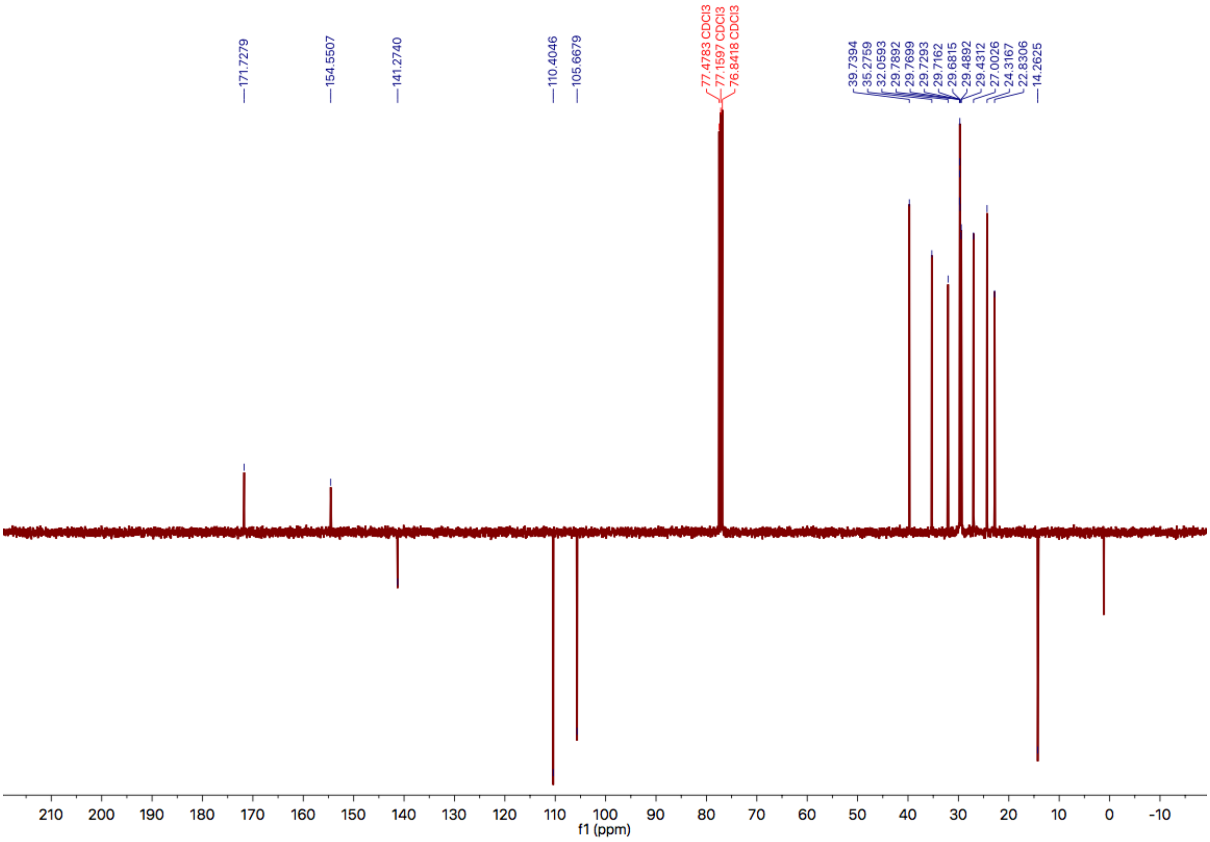

Compound 1e

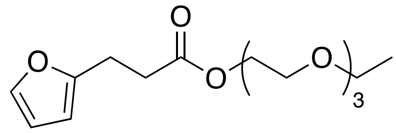

(400 MHz, CD_3OD)

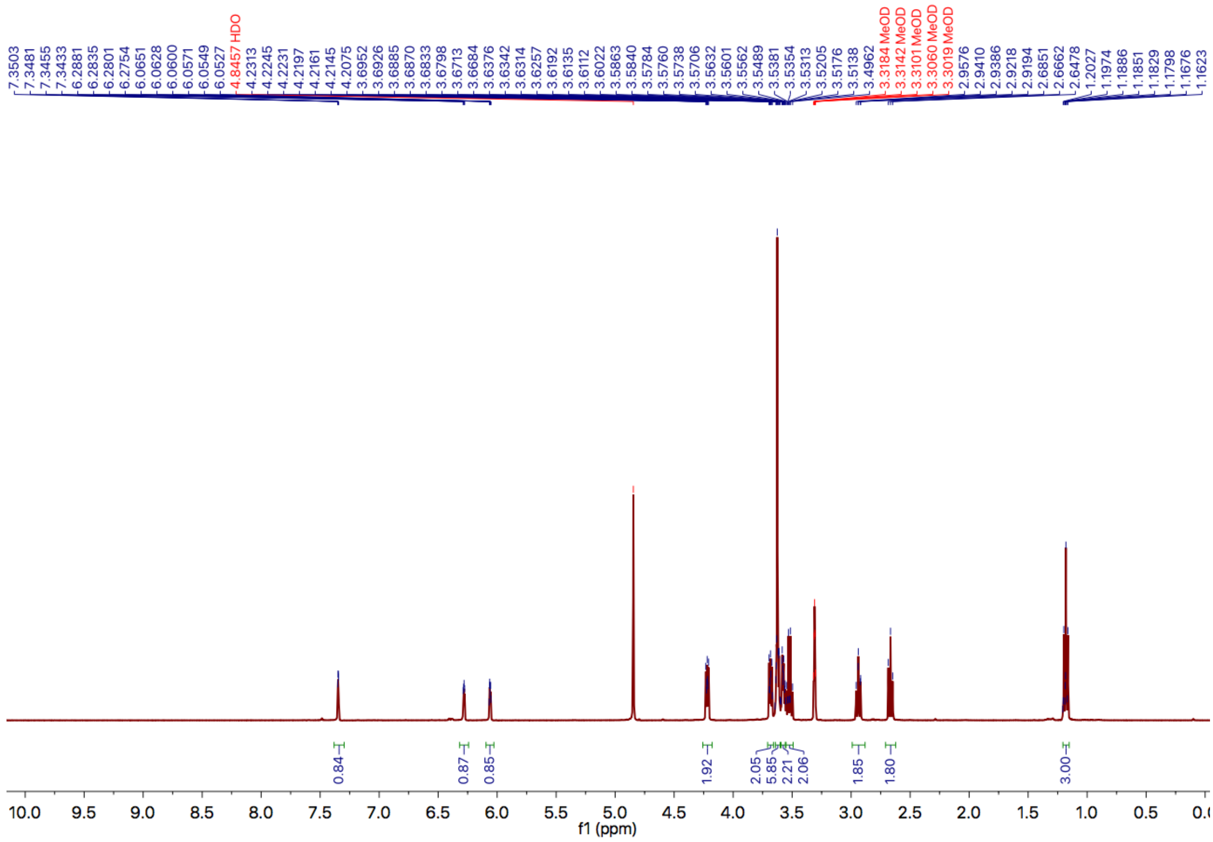

(^{13}C-APT,101 MHz, CD_3OD)

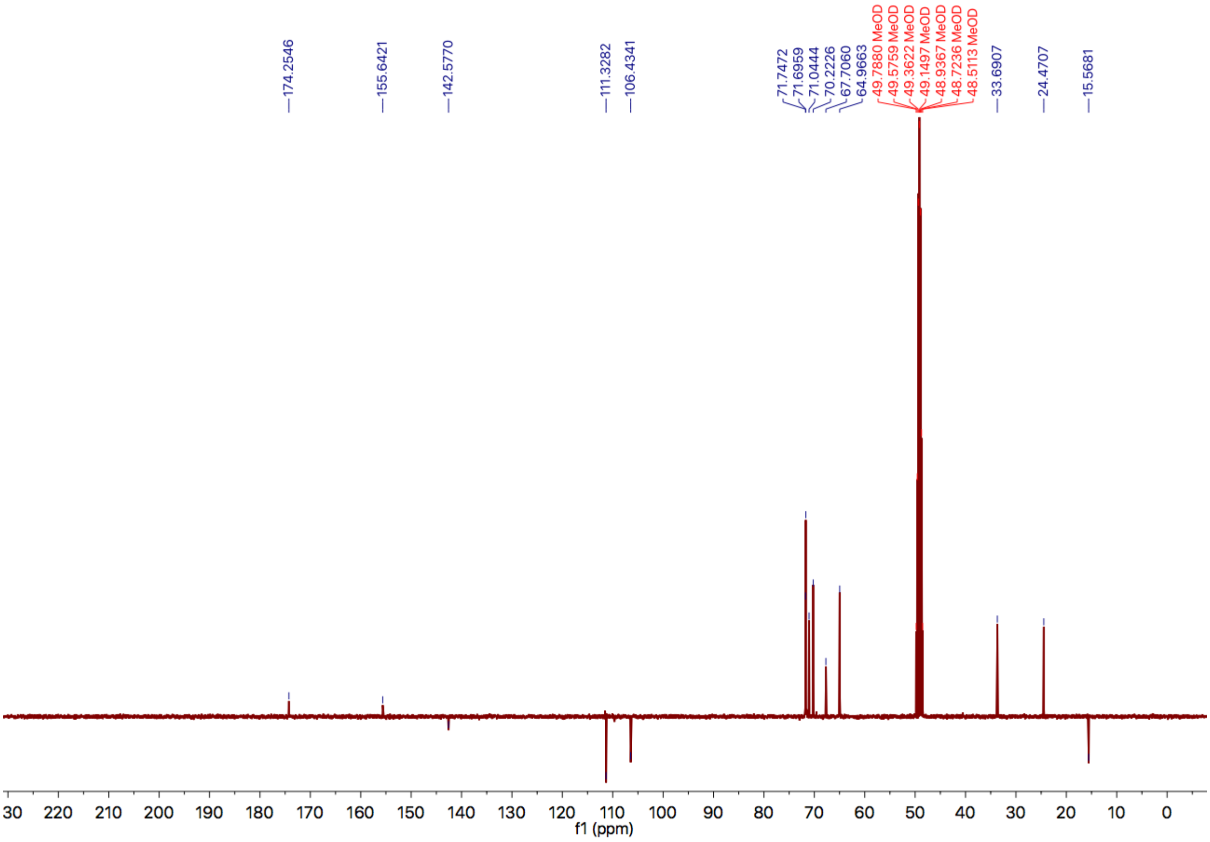

Compound 2

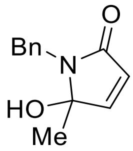

(500 MHz, $CDCl_3$)

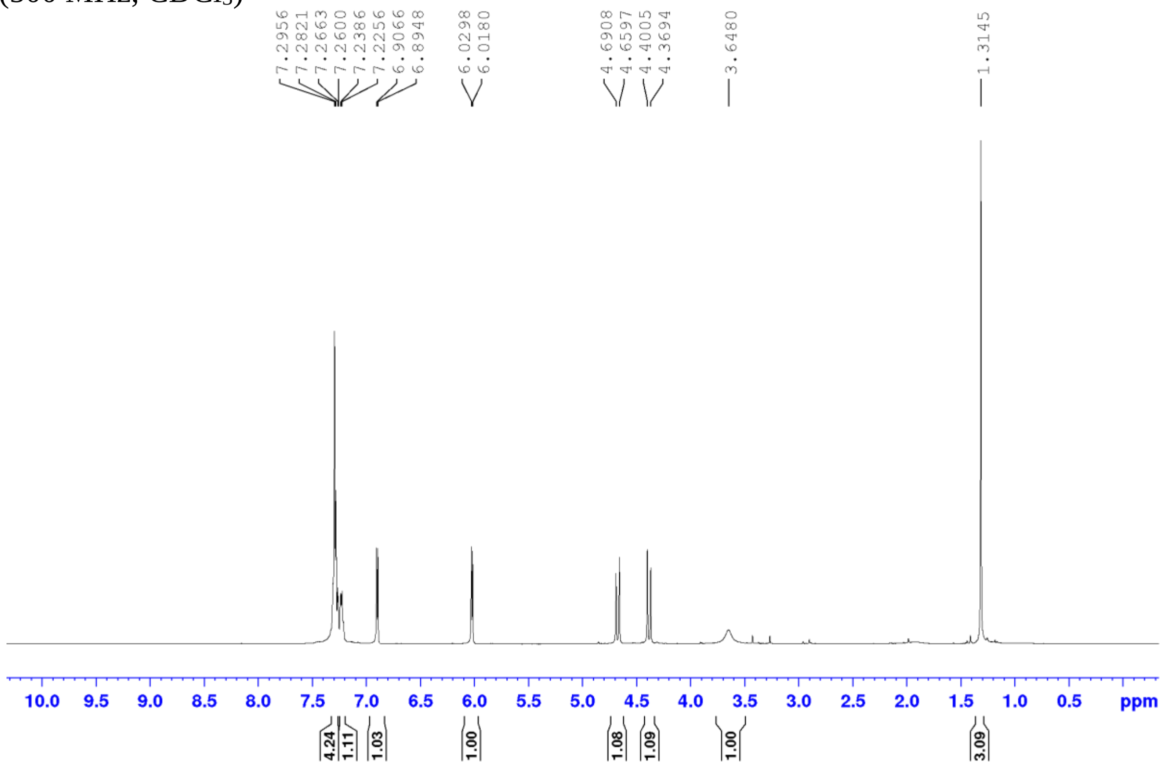

(125 MHz, $CDCl_3$)

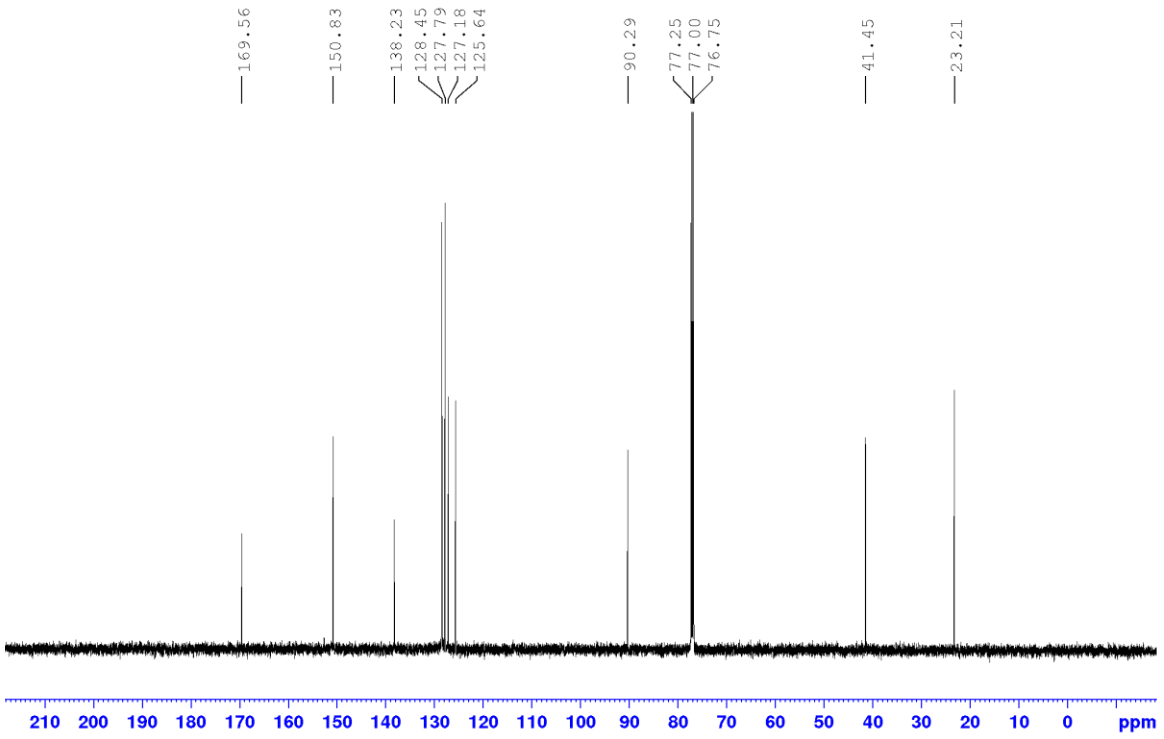

Compound 3

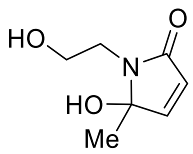

(500 MHz, $CDCl_3$)

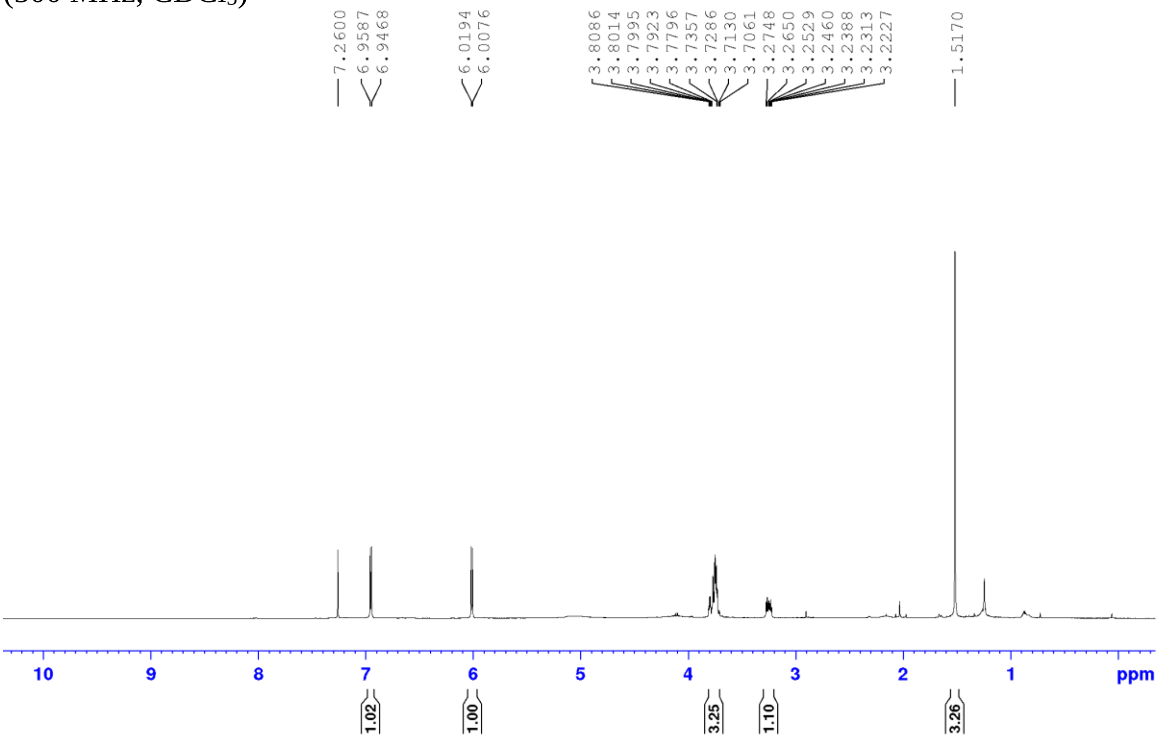

(125 MHz, $CDCl_3$)

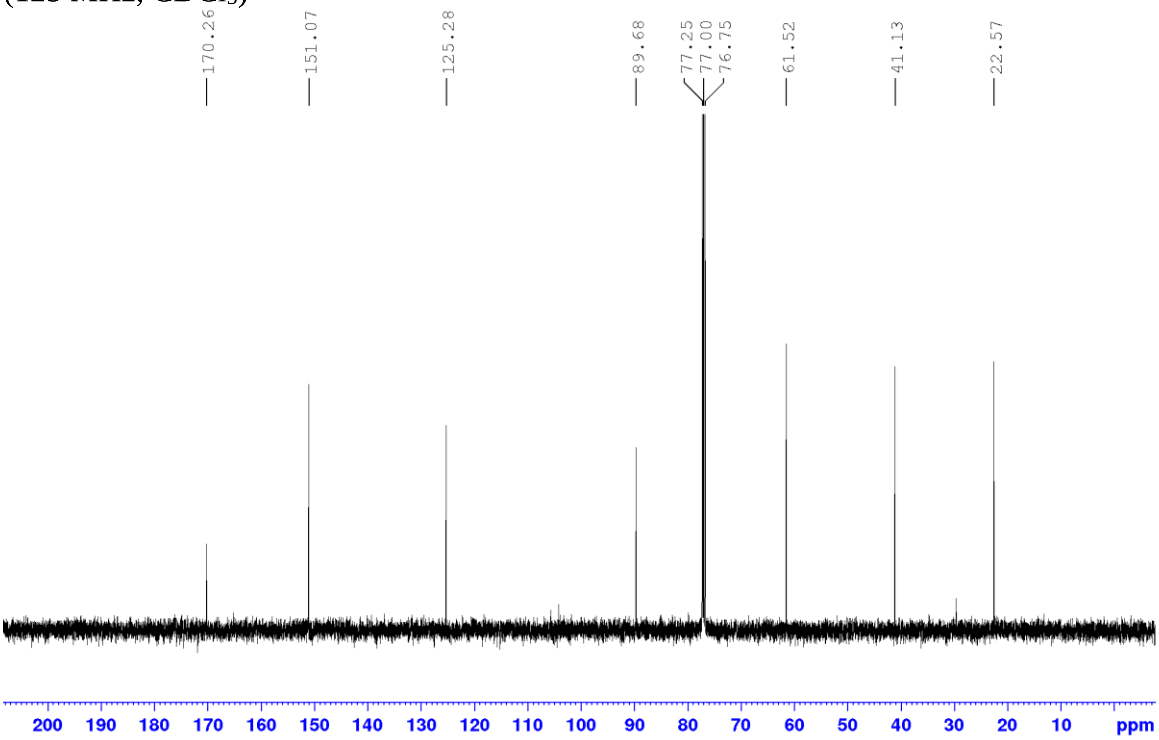

Compound 7

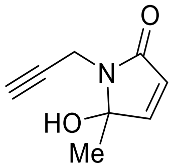

(500 MHz, $CDCl_3$)

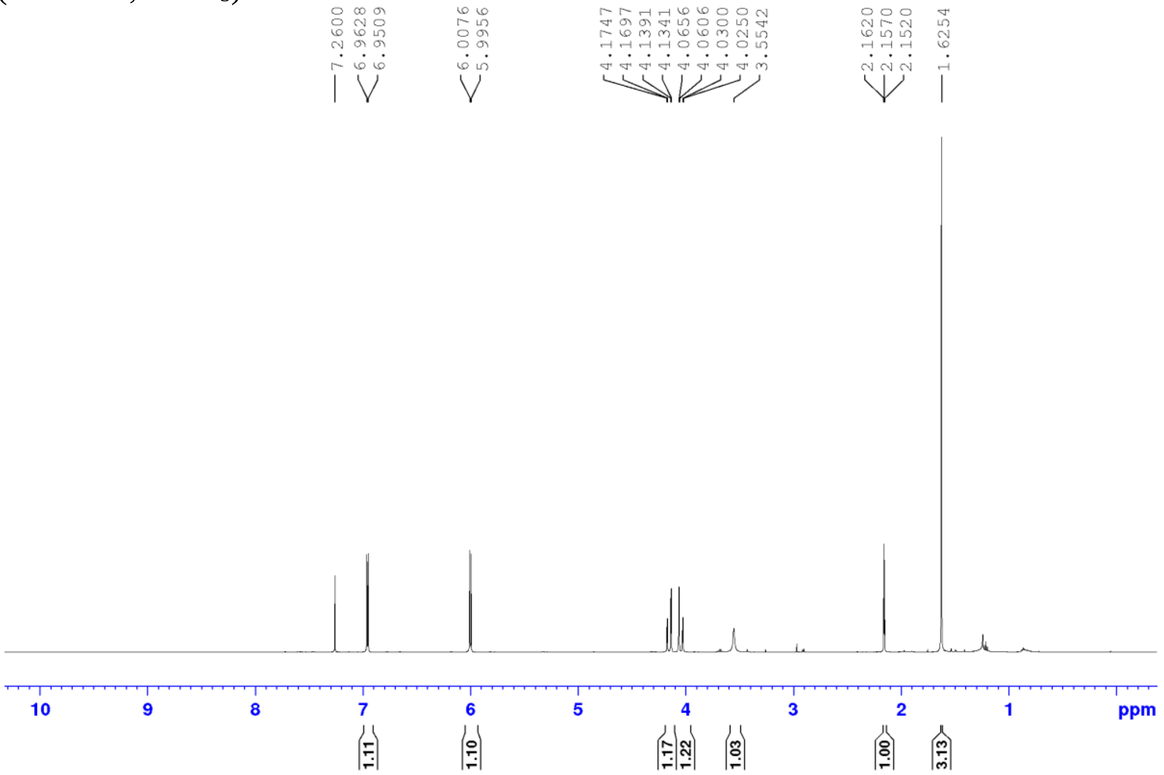

(125 MHz, $CDCl_3$)

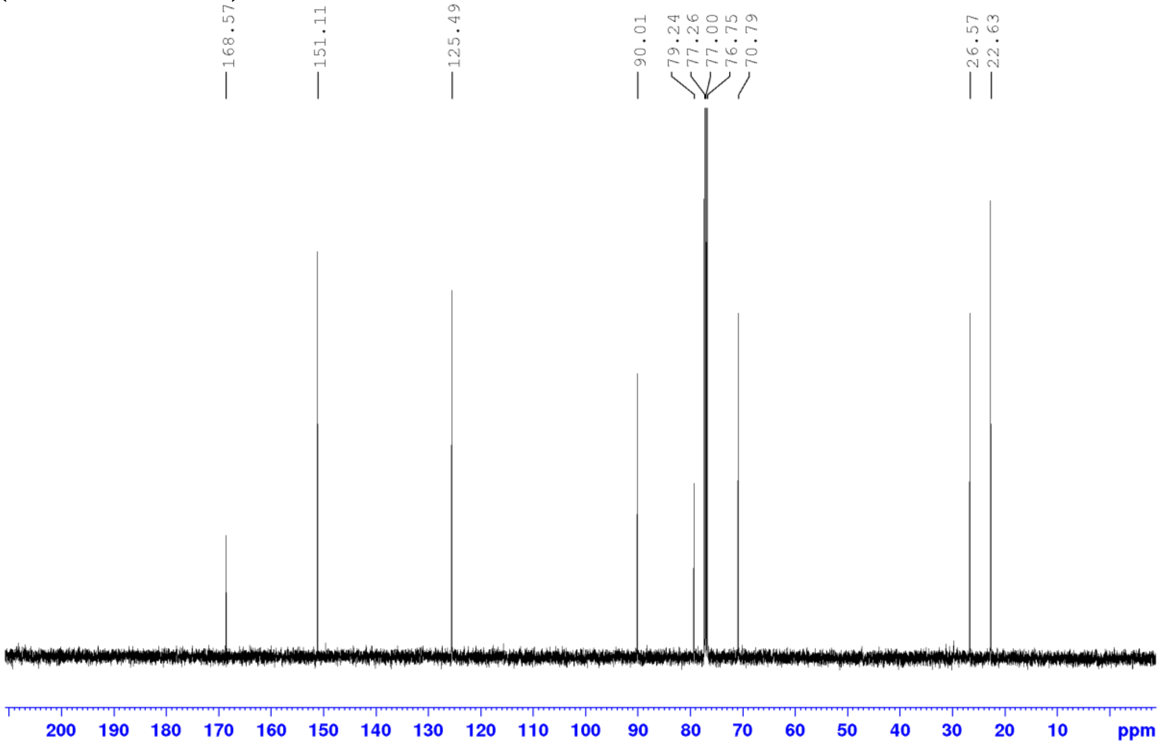

Compound 8

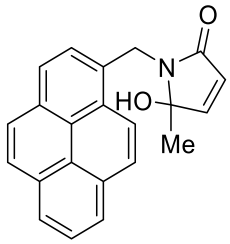

(500 MHz, $CDCl_3$)

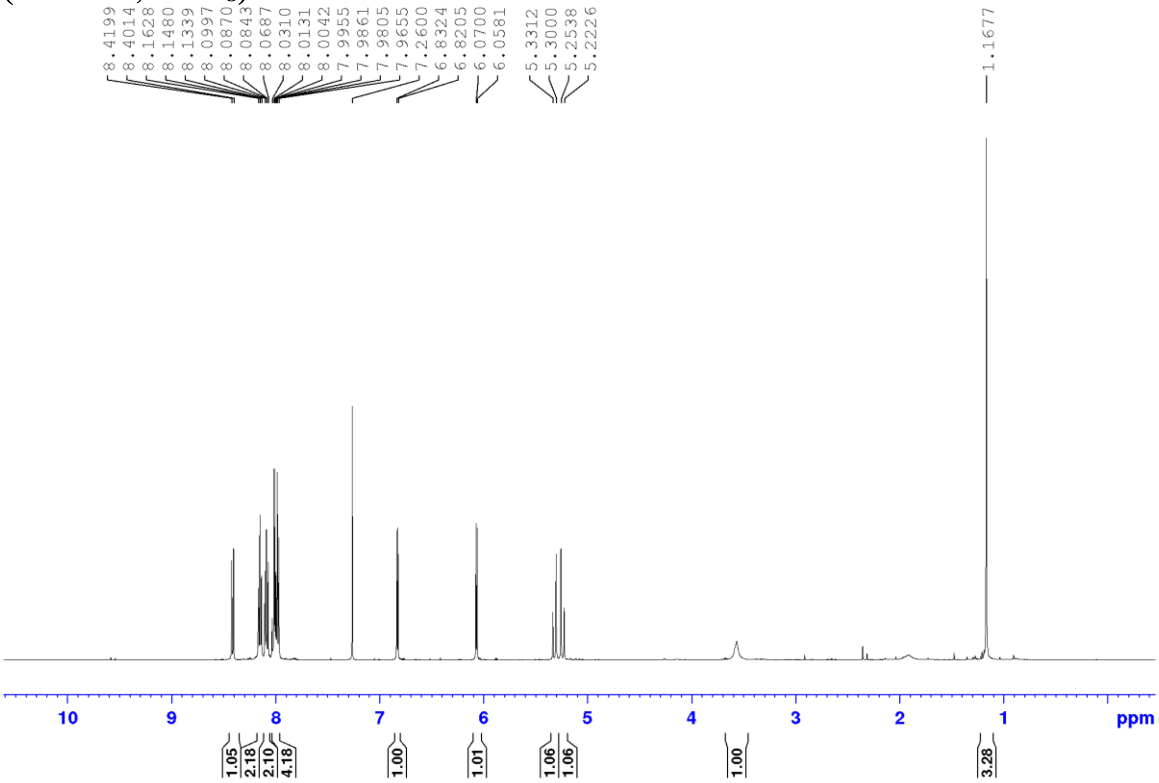

(125 MHz, $CDCl_3$)

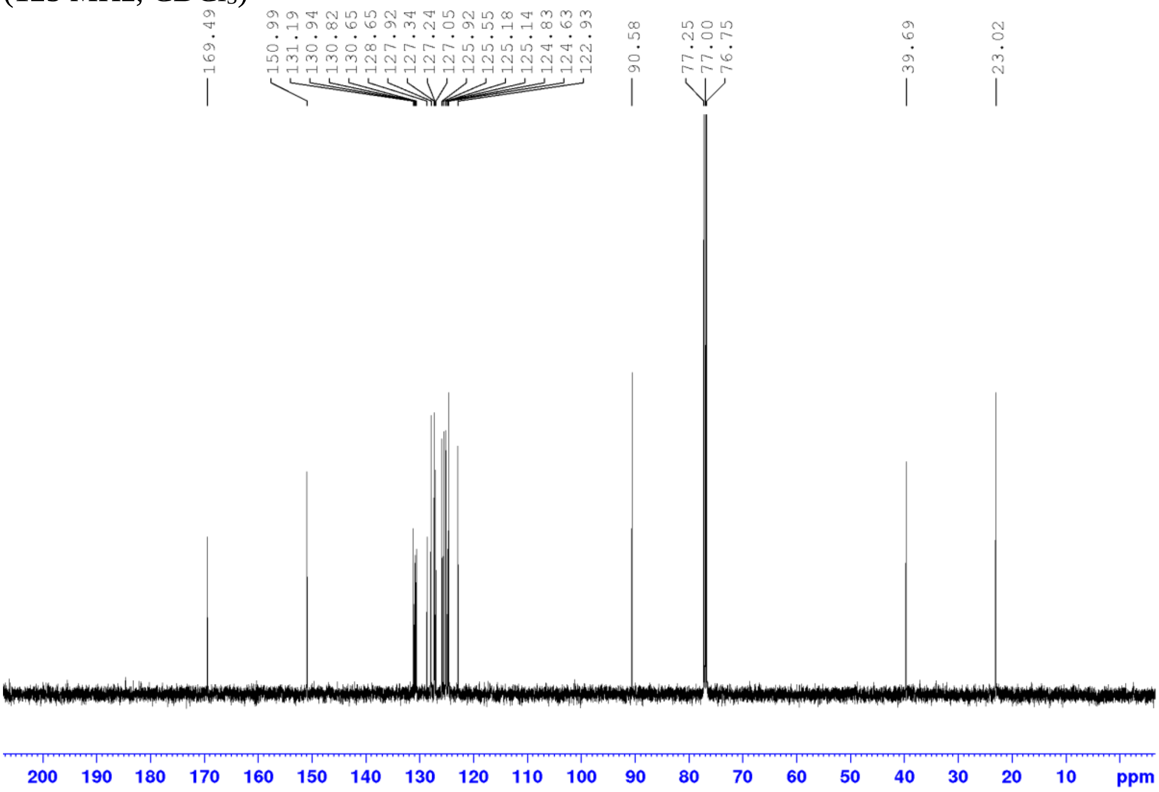

Compound 9

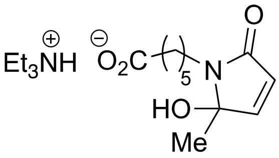

(400 MHz, DMSO-d_6)

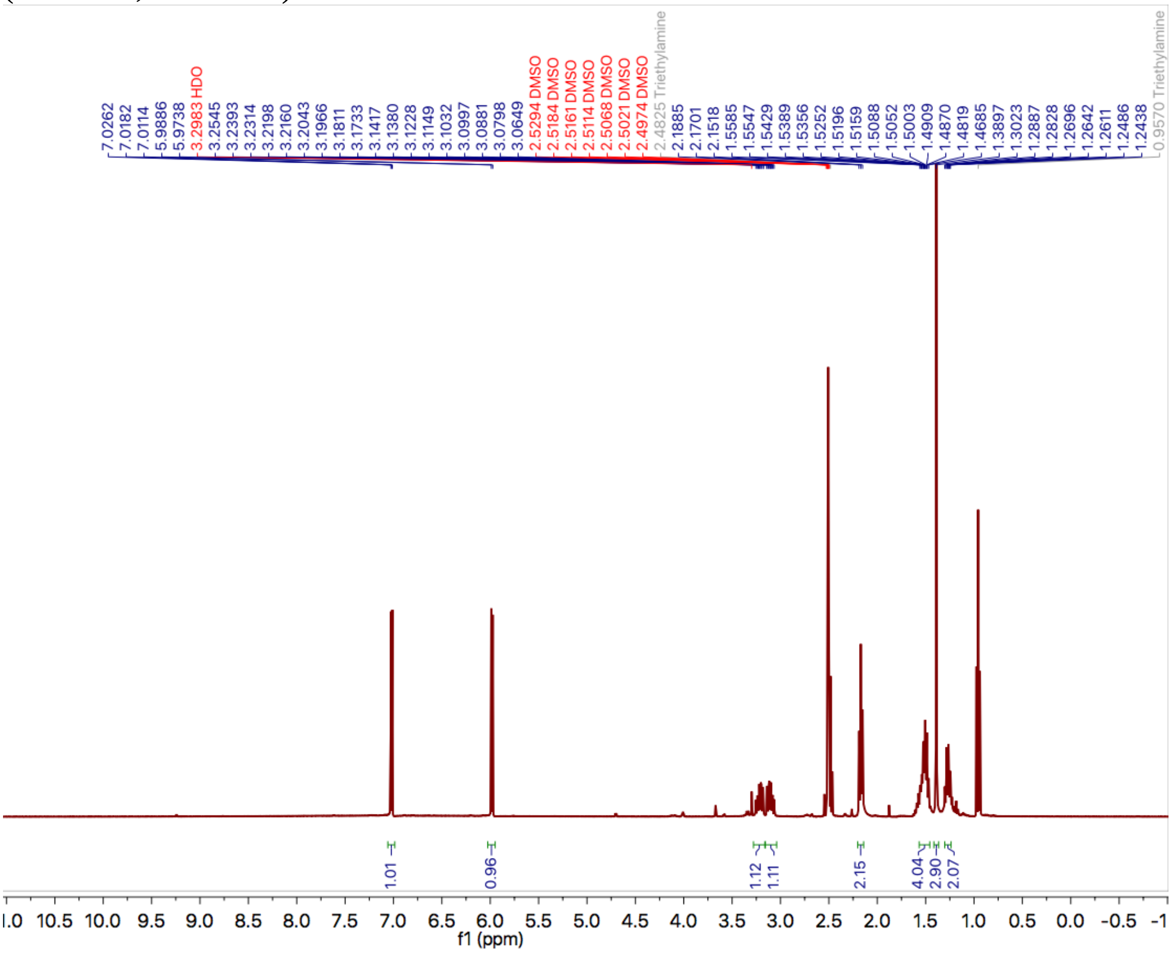

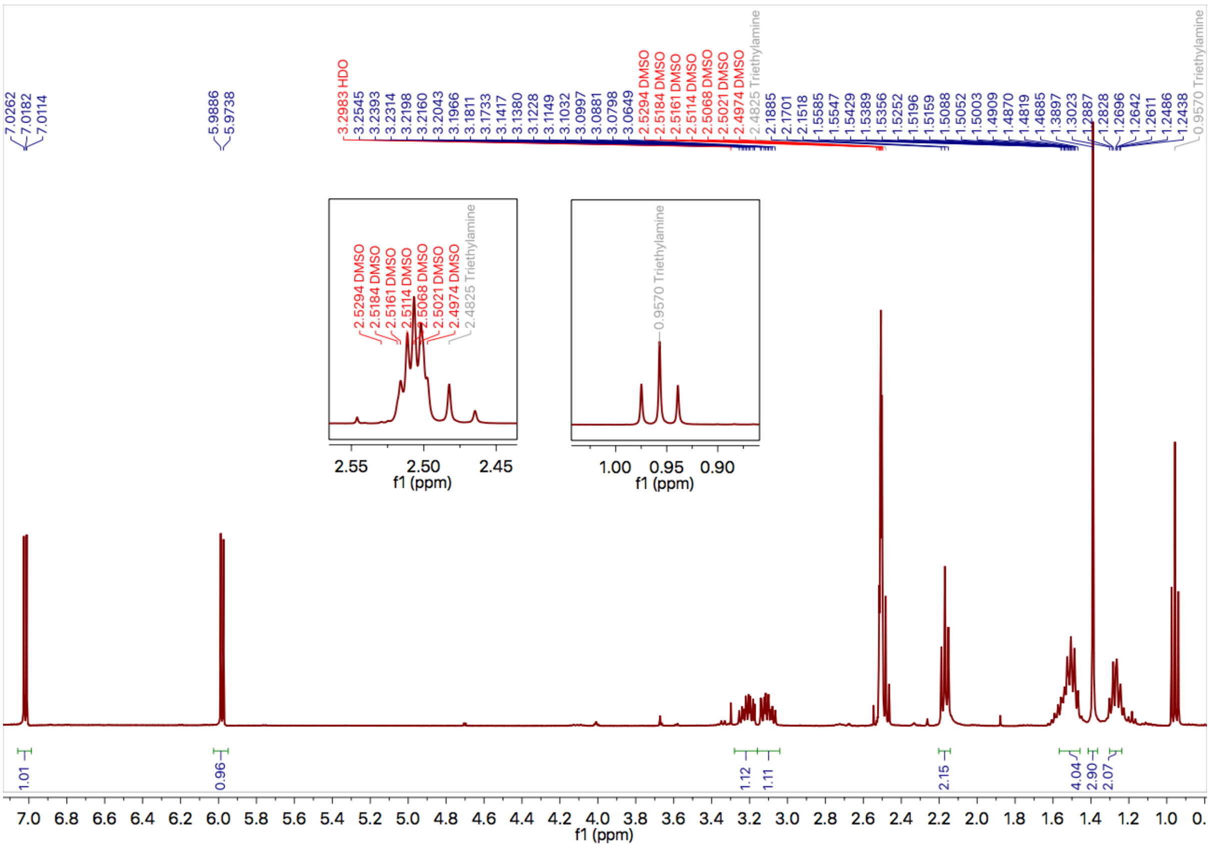

Zoomed region of proton NMR

(^{13}C-APT, 101 MHz, DMSO-d$_6$)

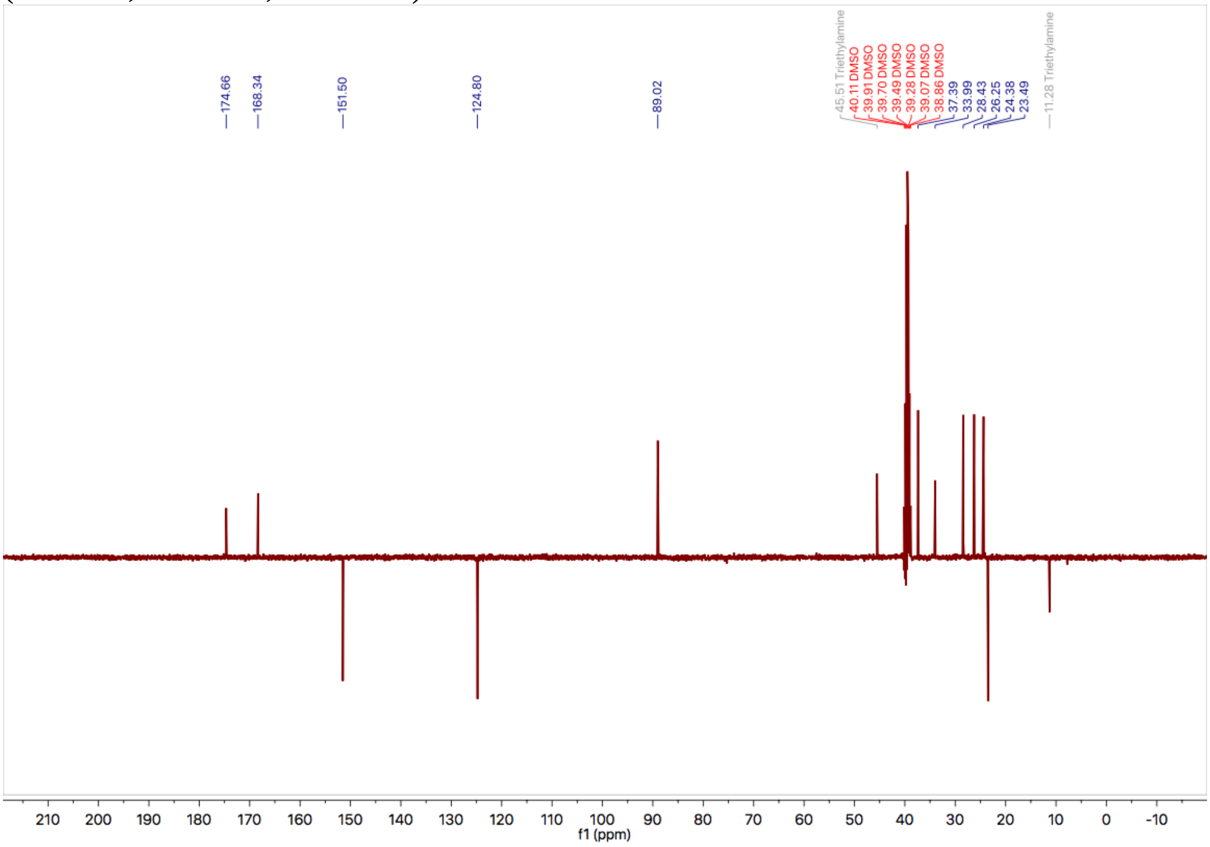

Compound 10

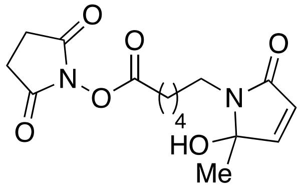

(400 MHz, CD_3CN)

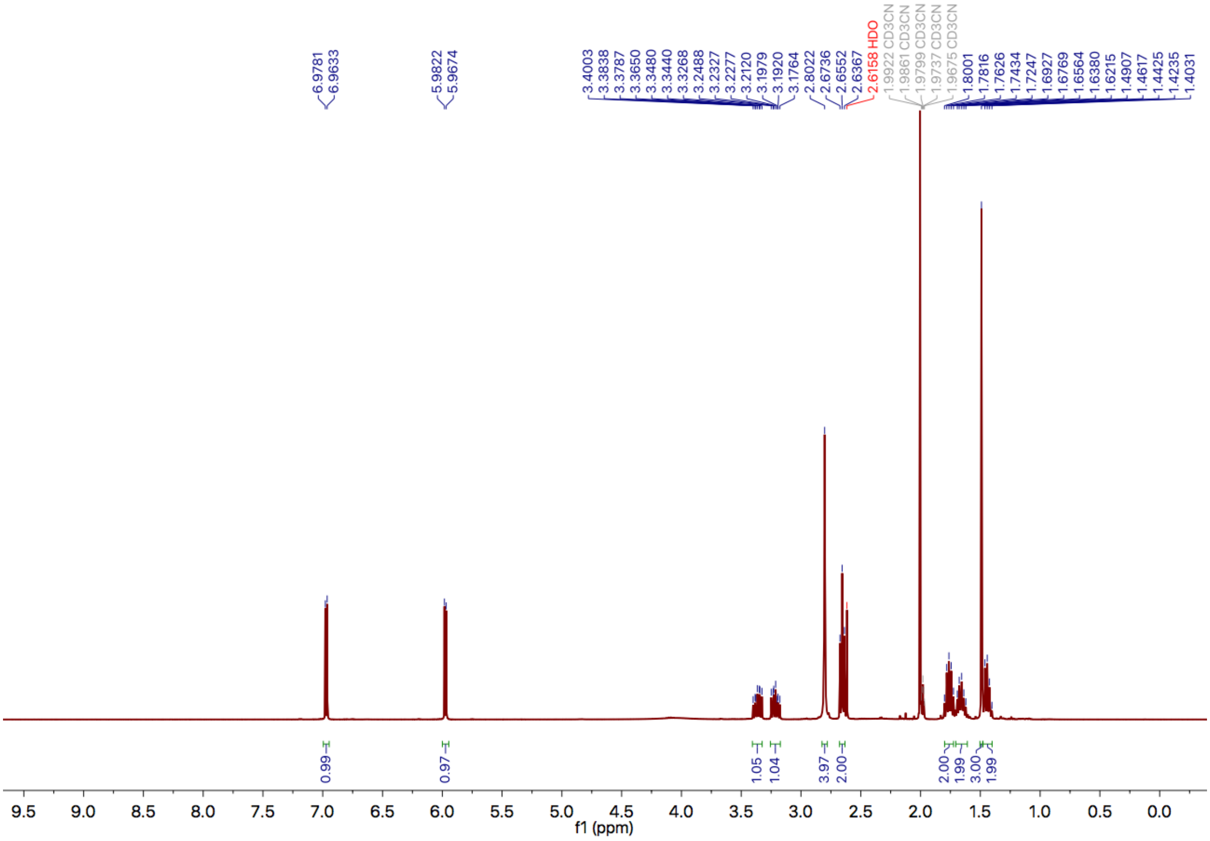

(^{13}C-APT,101 MHz, CD_3CN)

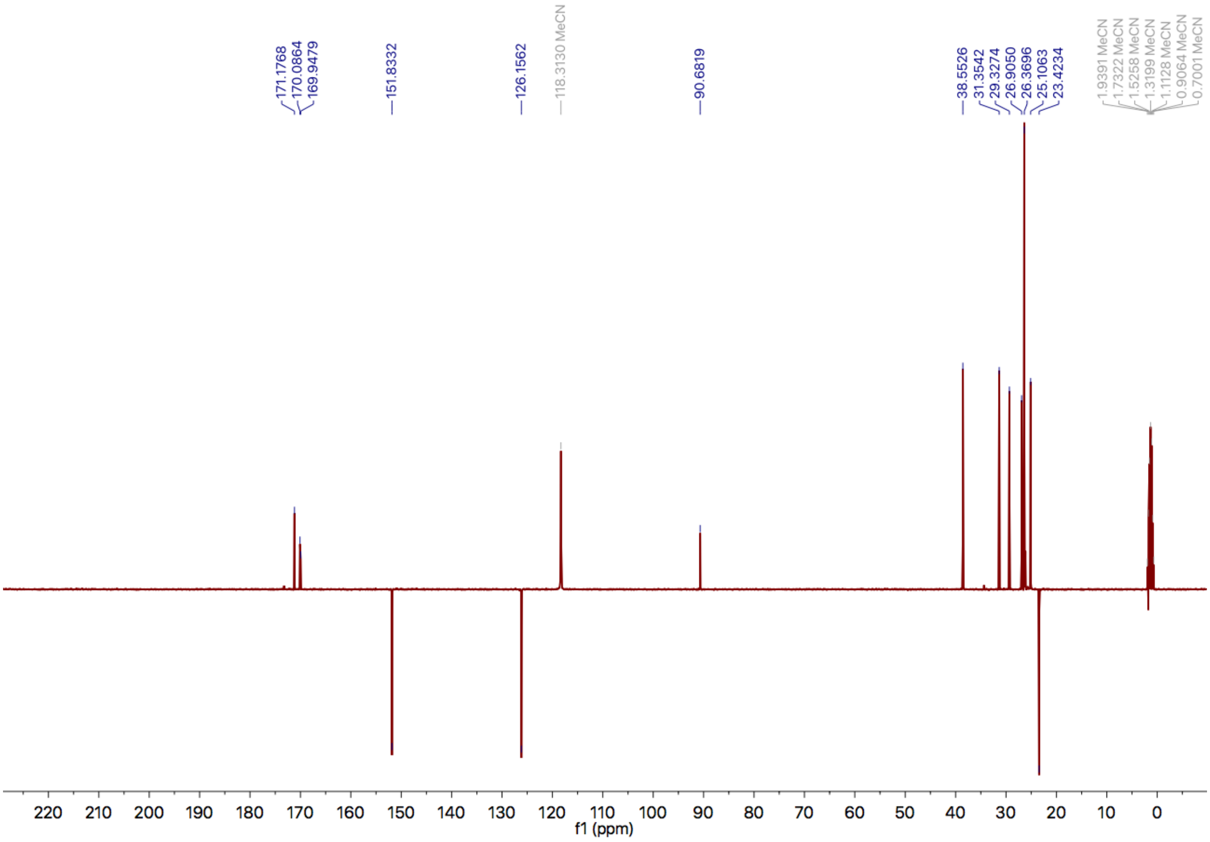

Compound 11

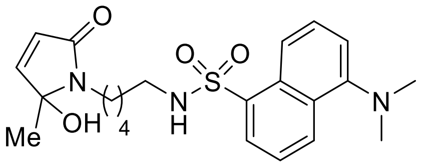

(400 MHz, CD_3OD)

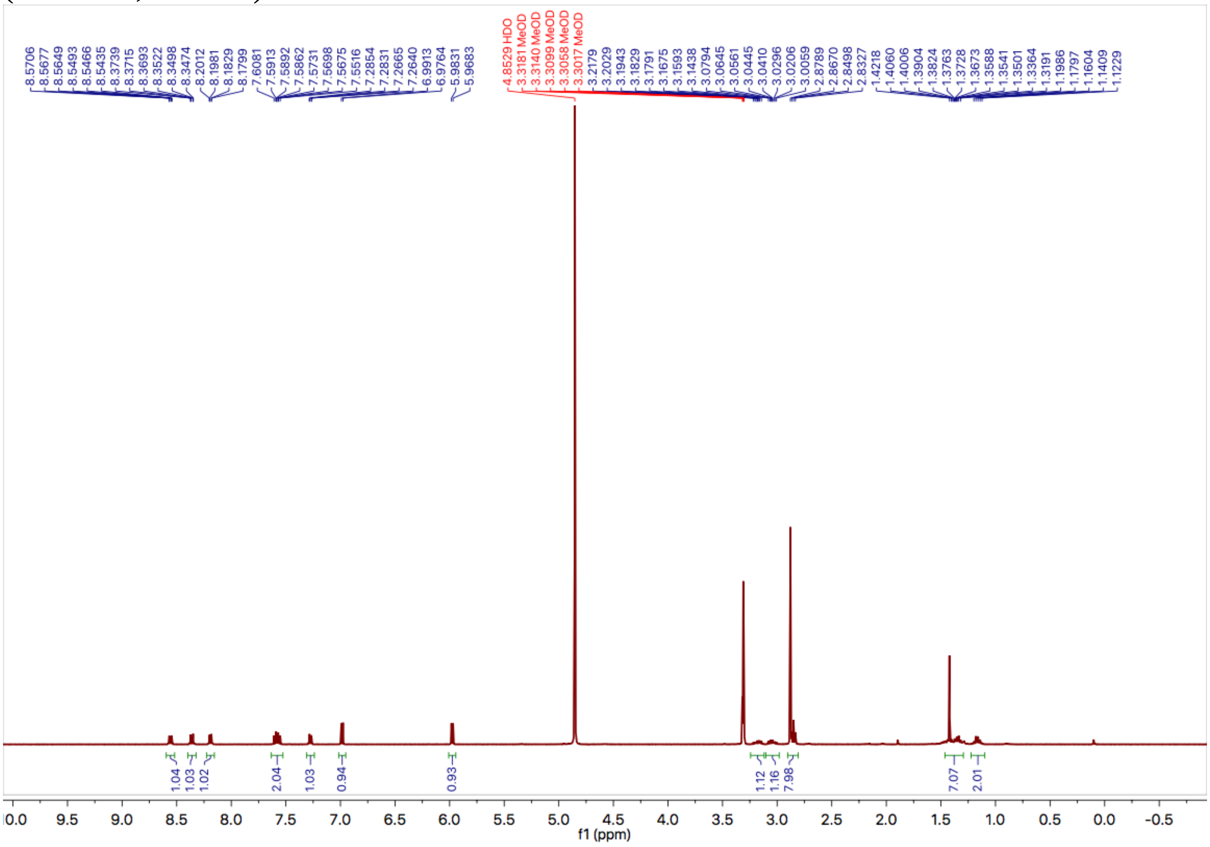

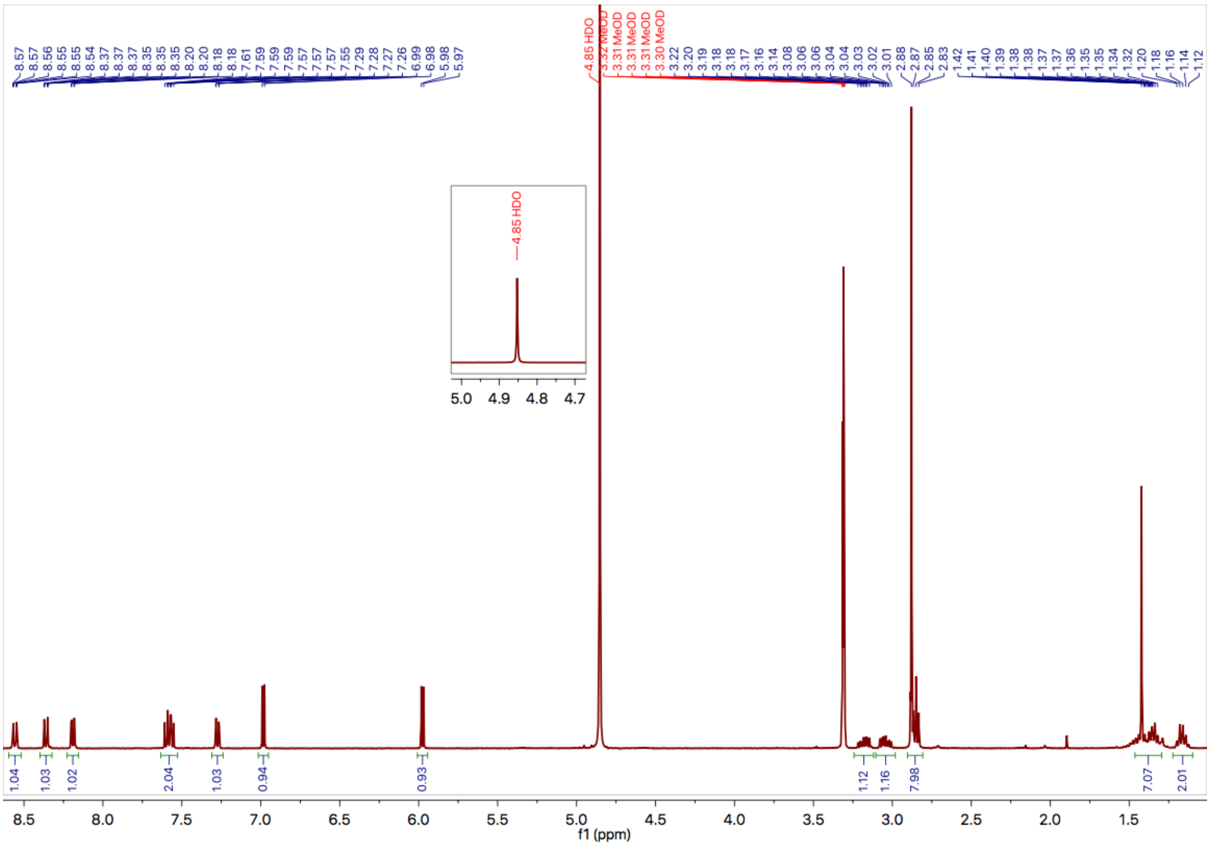

Zoomed region of proton NMR

(^{13}C-APT,101 MHz, CD_3OD)

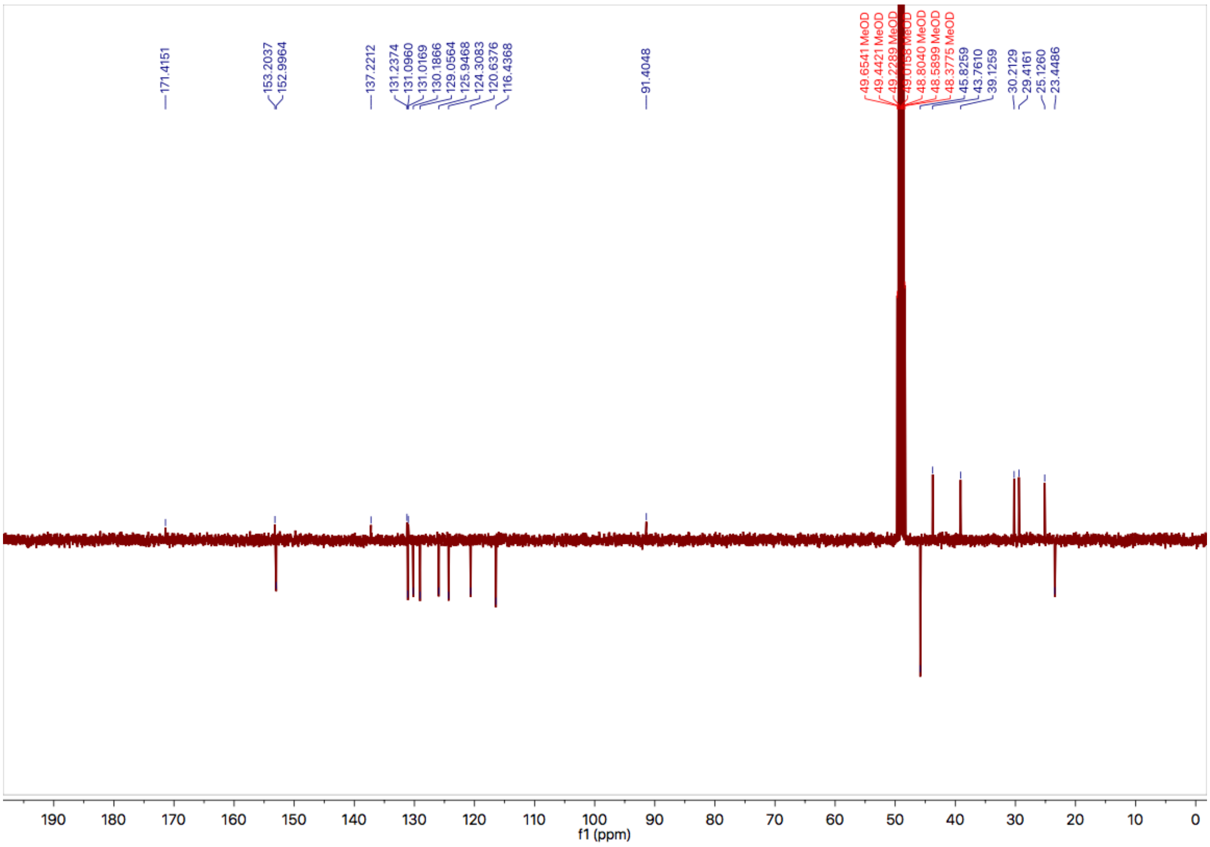

Compound 12

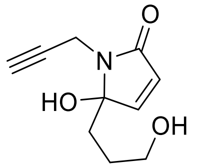

(500 MHz, CD_3OD)

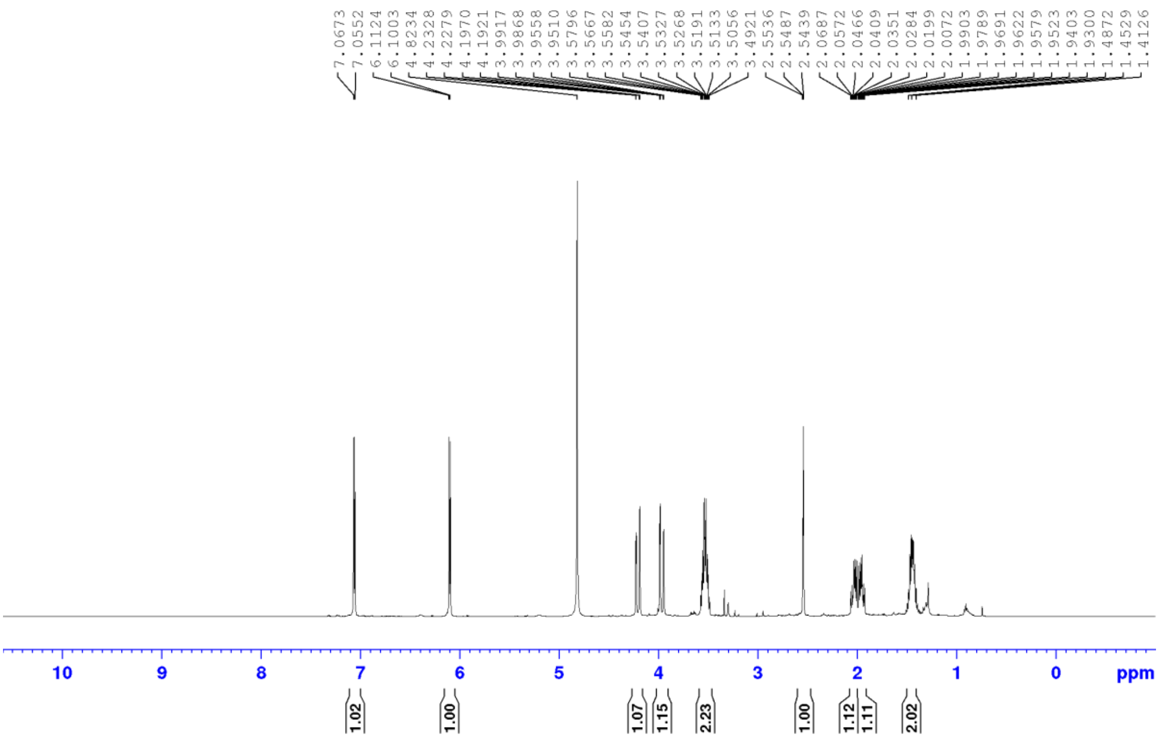

(125 MHz, CD_3OD)

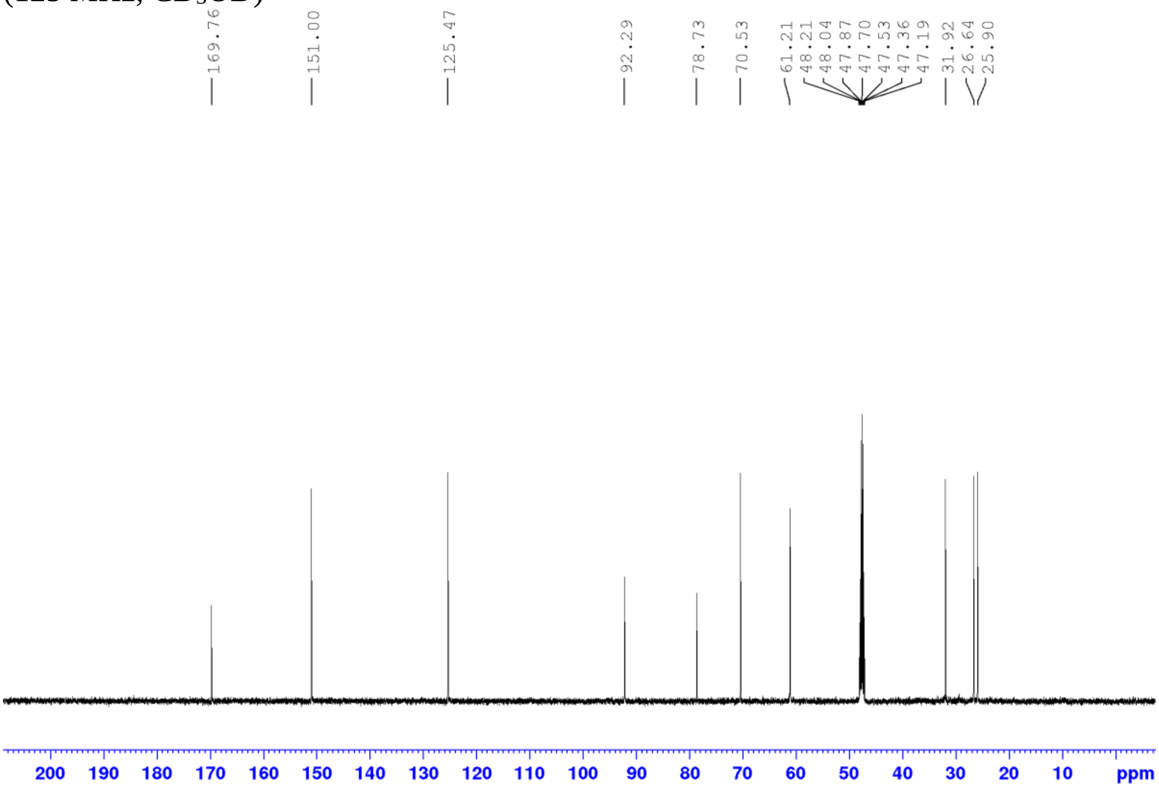

Compound 13

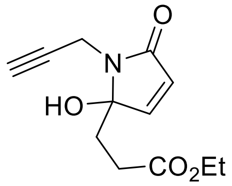

(300 MHz, $CDCl_3$)

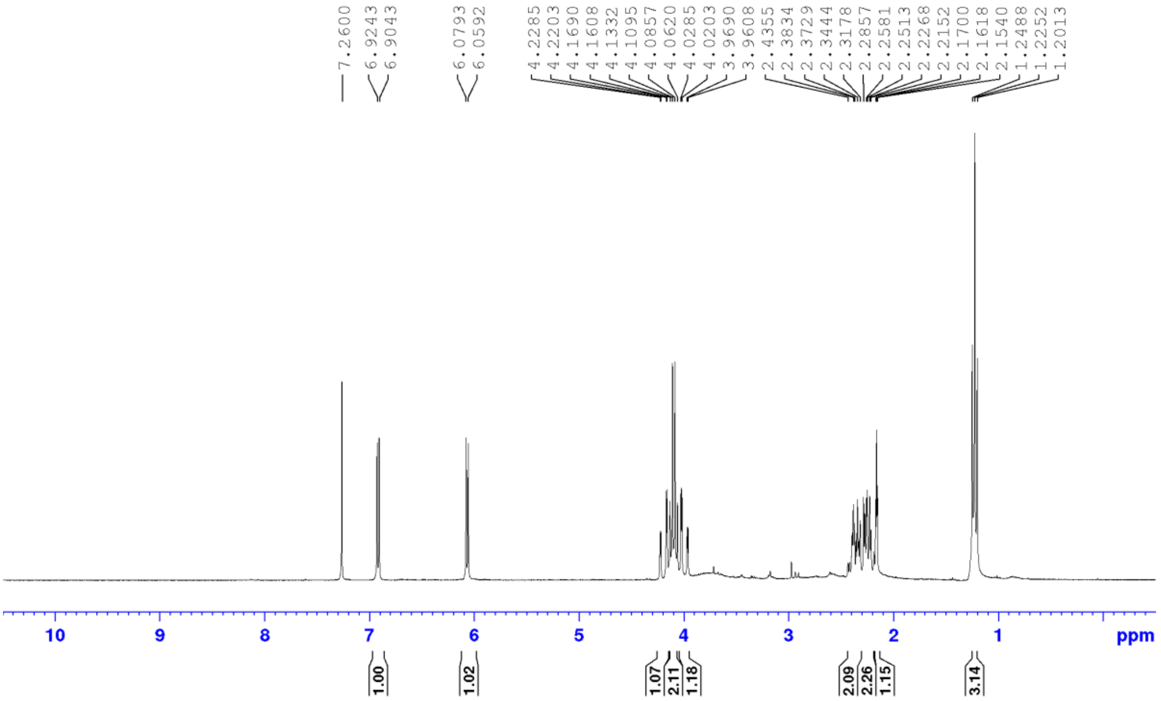

(75 MHz, $CDCl_3$)

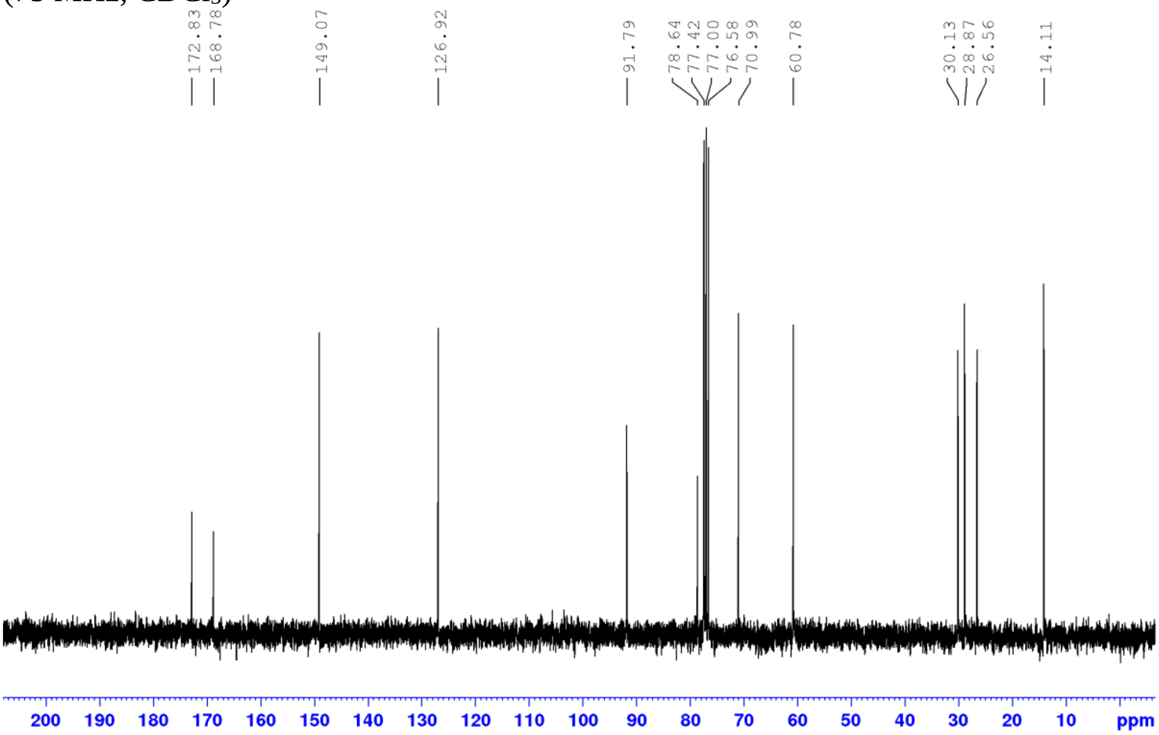
172.83
168.78
149.07
126.92
91.79
78.64
77.42
77.00
76.58
70.99
60.78
30.13
28.87
26.56
14.11
200 190 180 170 160 150 140 130 120 110 100 90 80 70 60 50 40 30 20 10 ppm

Compound 14

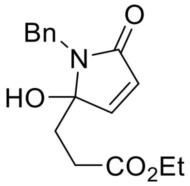

(500 MHz, $CDCl_3$)

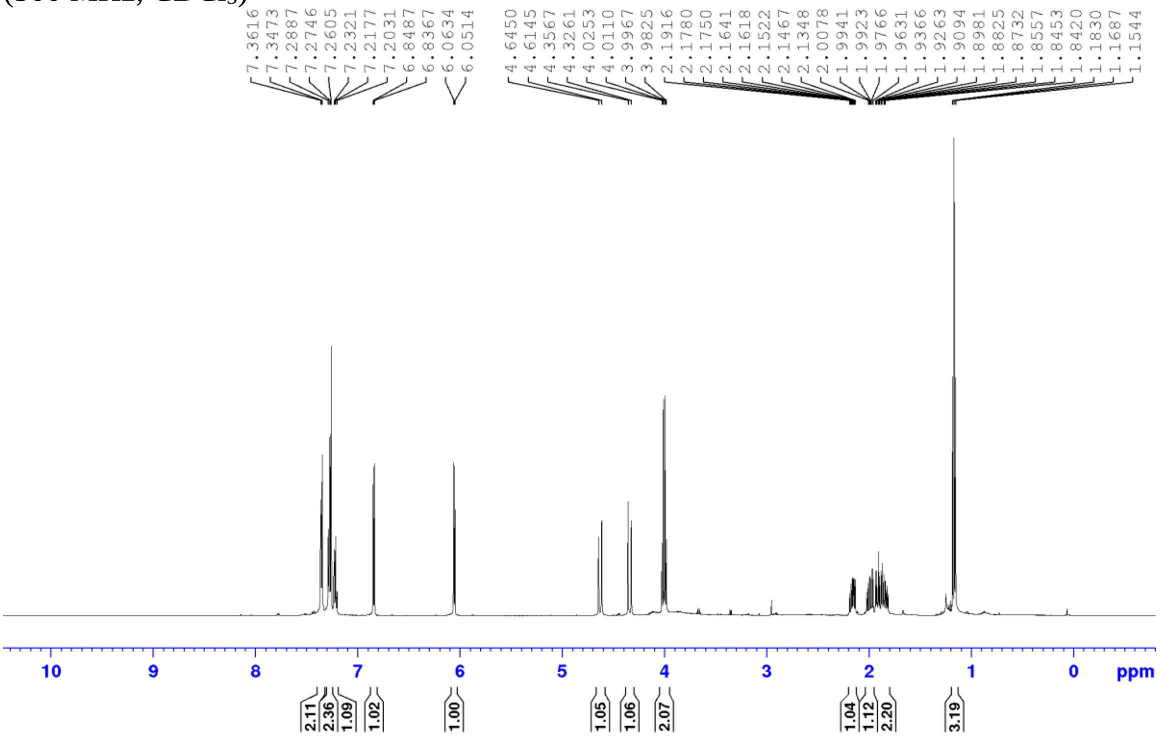

(125 MHz, $CDCl_3$)

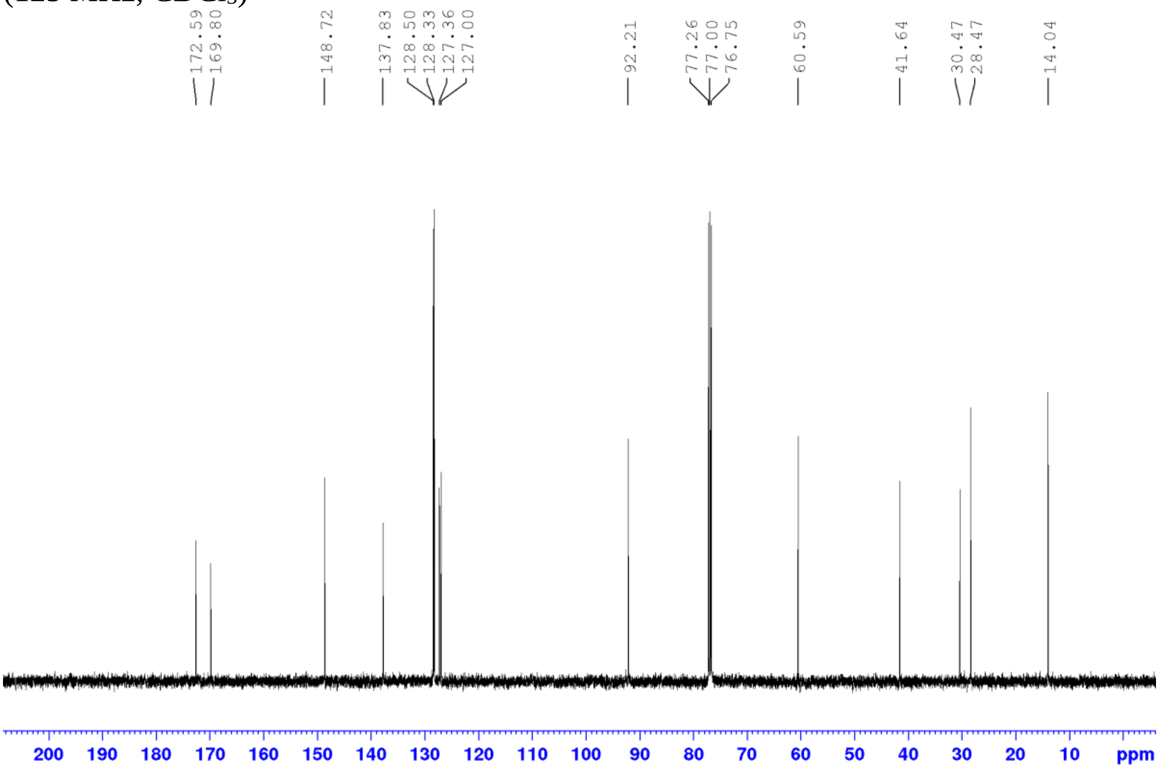

Compound 15

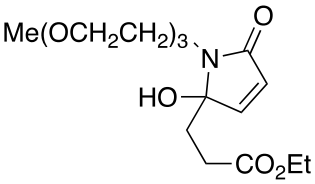

(400 MHz, CD_3OD)

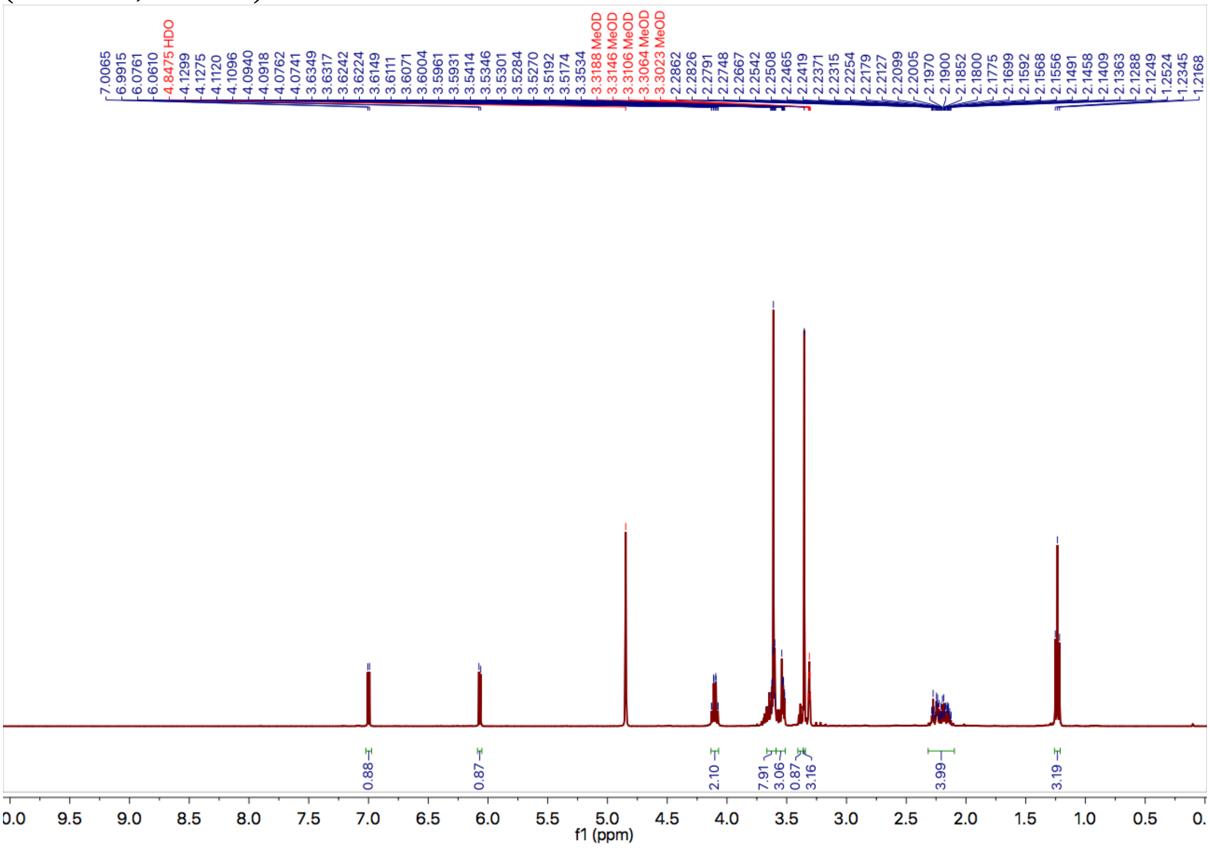

(^{13}C-APT,101 MHz, CD_3OD)

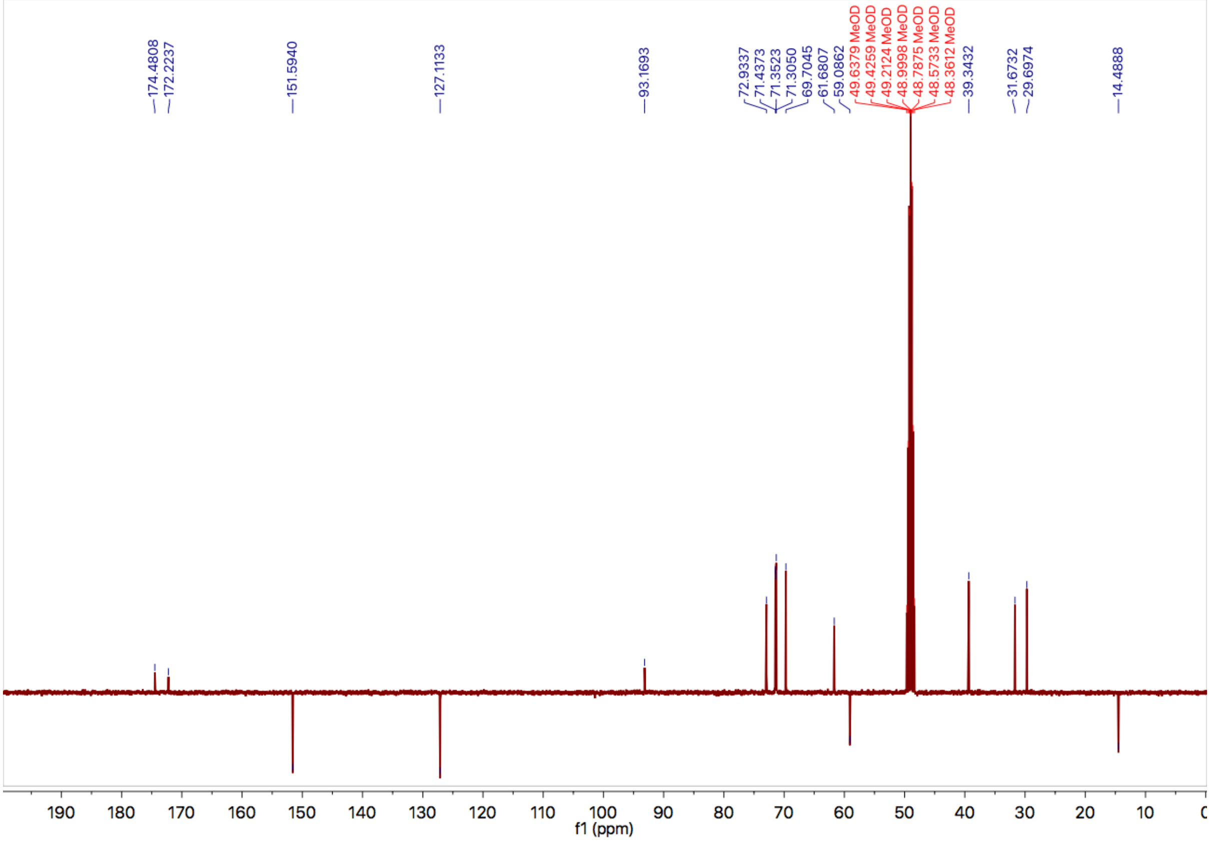

Compound 16

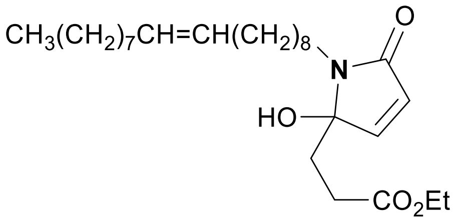

(400 MHz, DMSO-d6)

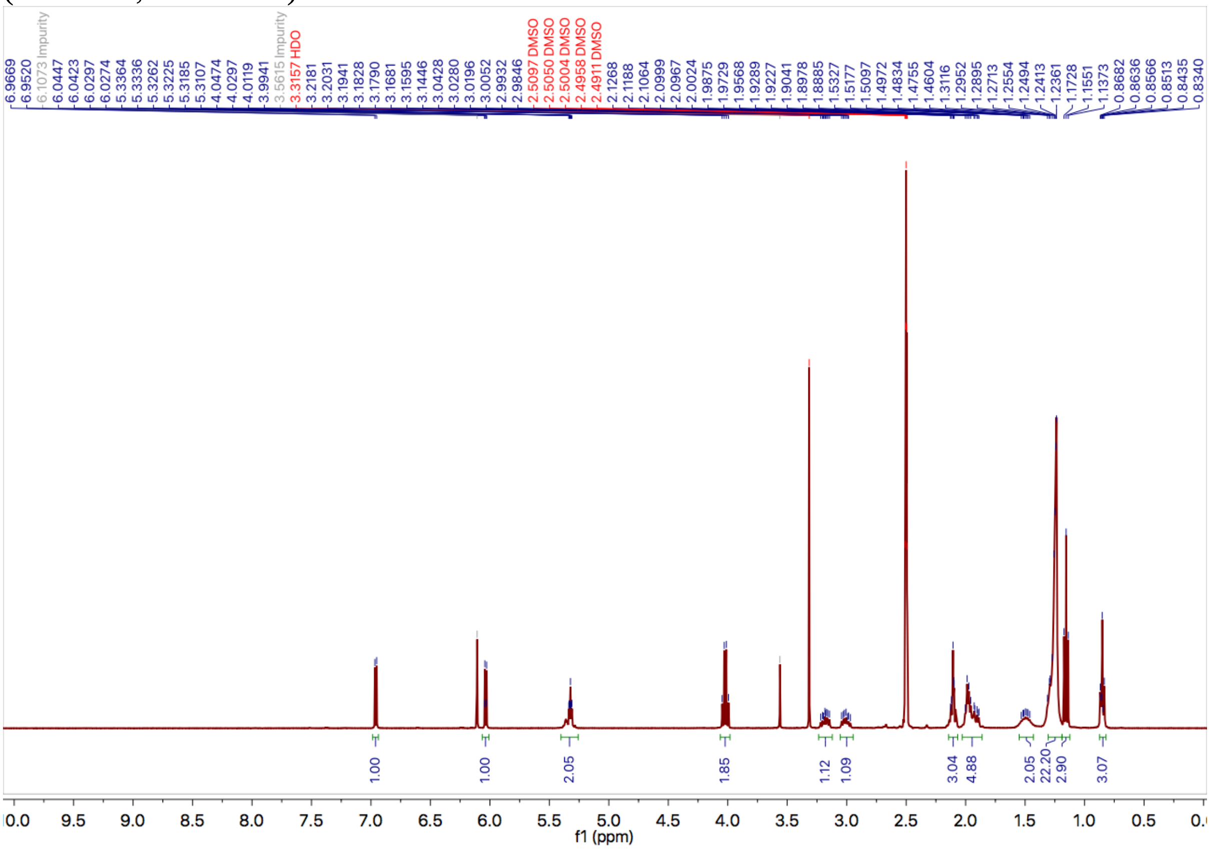

(^{13}C-APT,101 MHz, DMSO-d6)

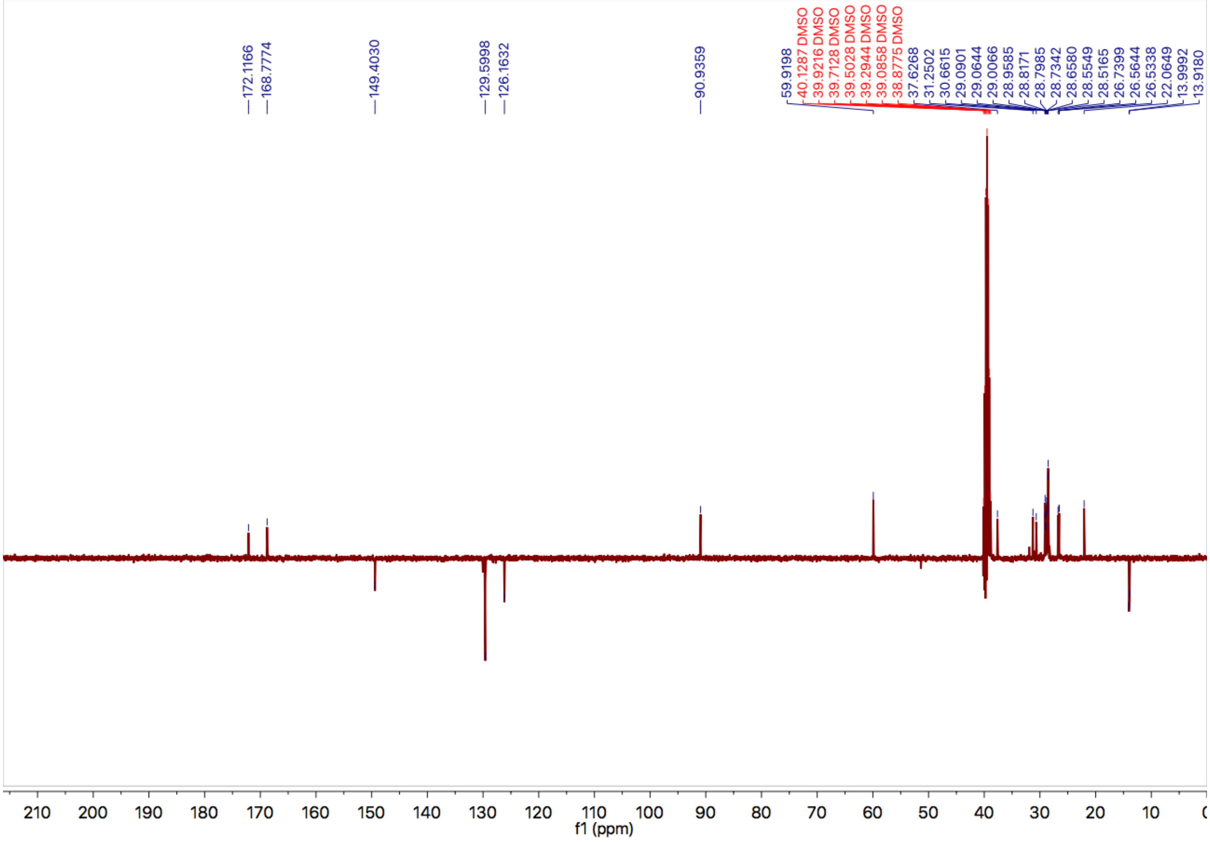

Compound 17

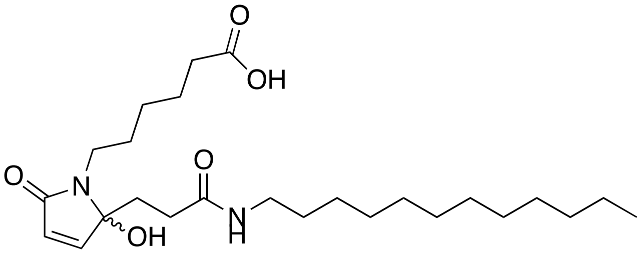

(400 MHz, DMSO-d6)

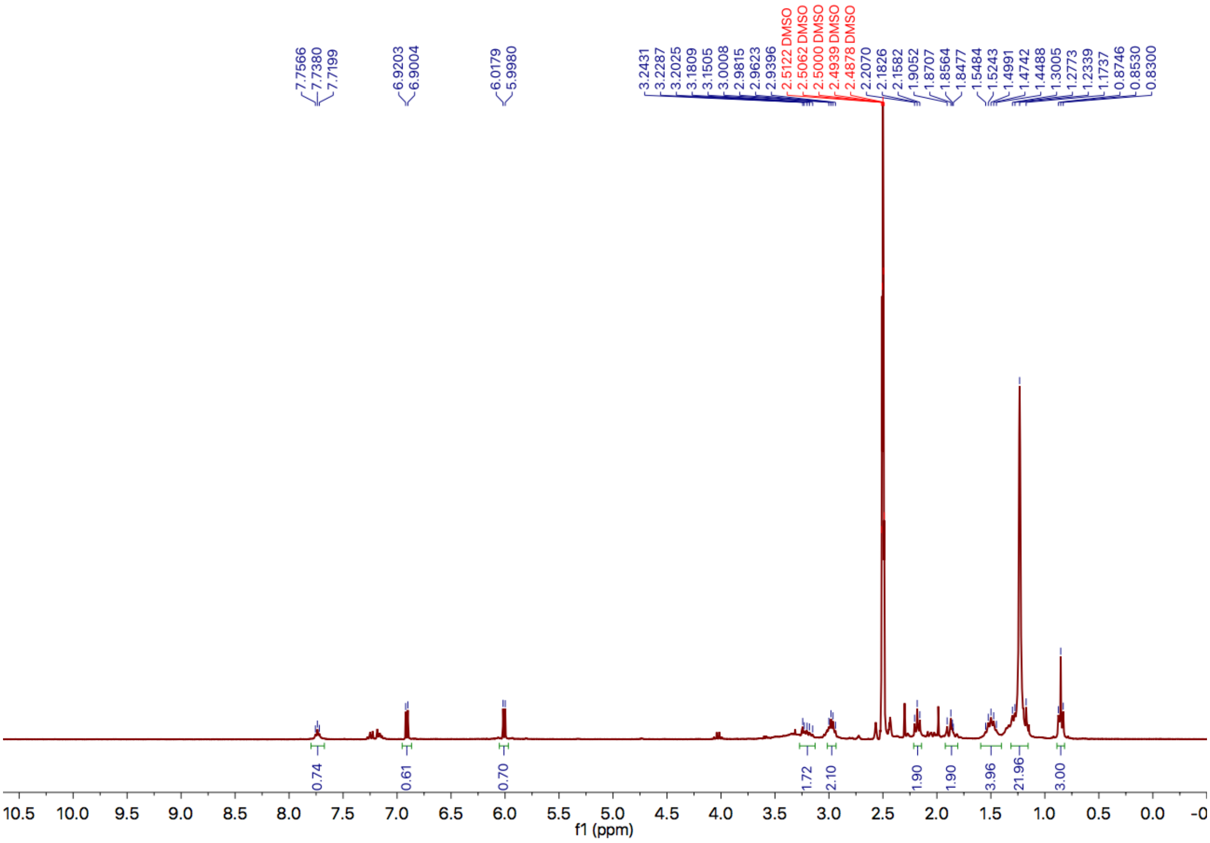

Compound 18

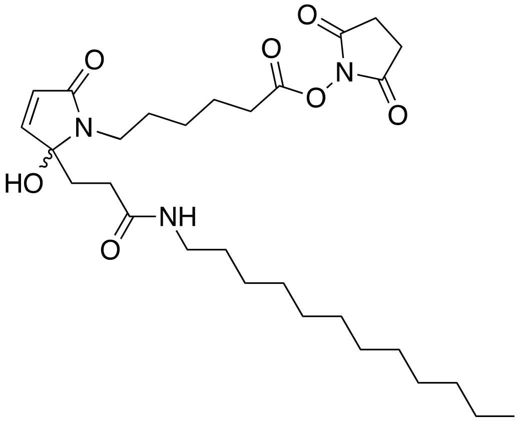

(400 MHz, CD_3OD)

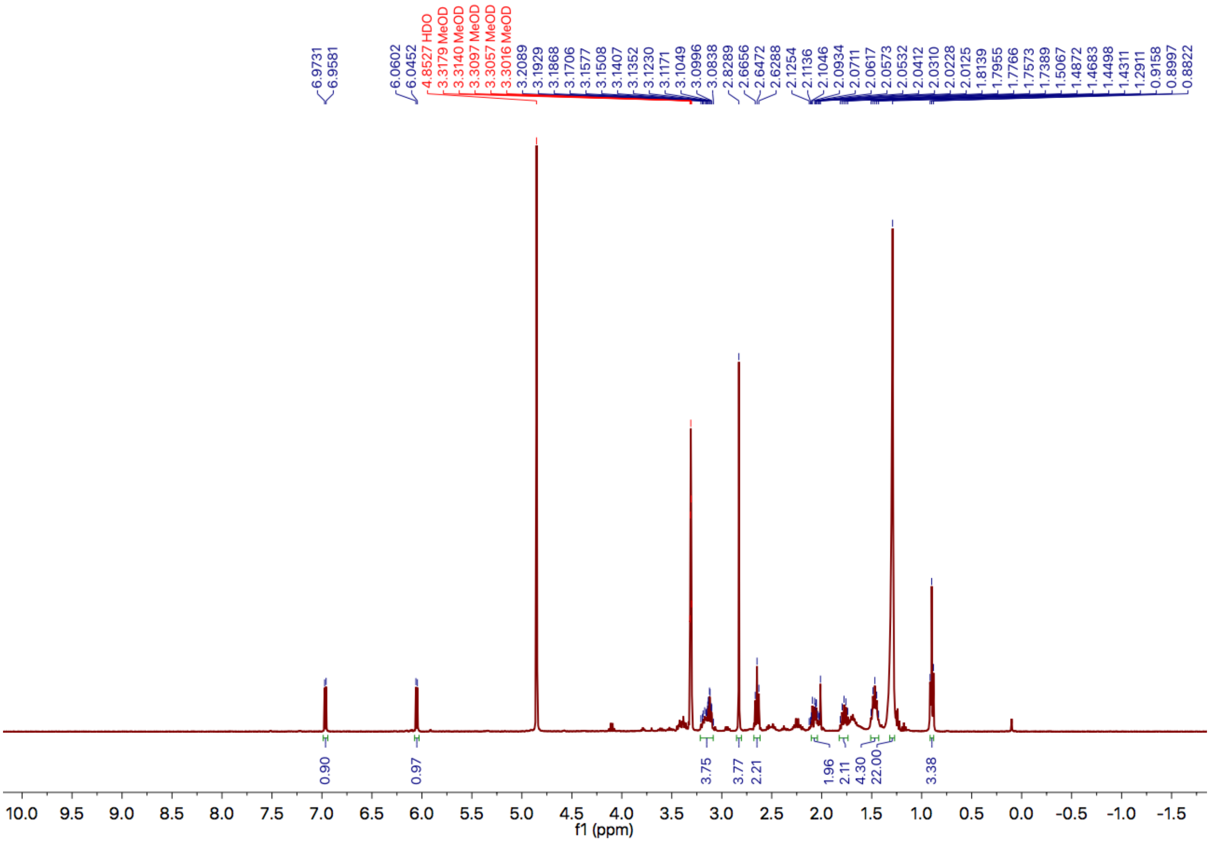

(^{13}C-APT,101 MHz, CD_3OD)

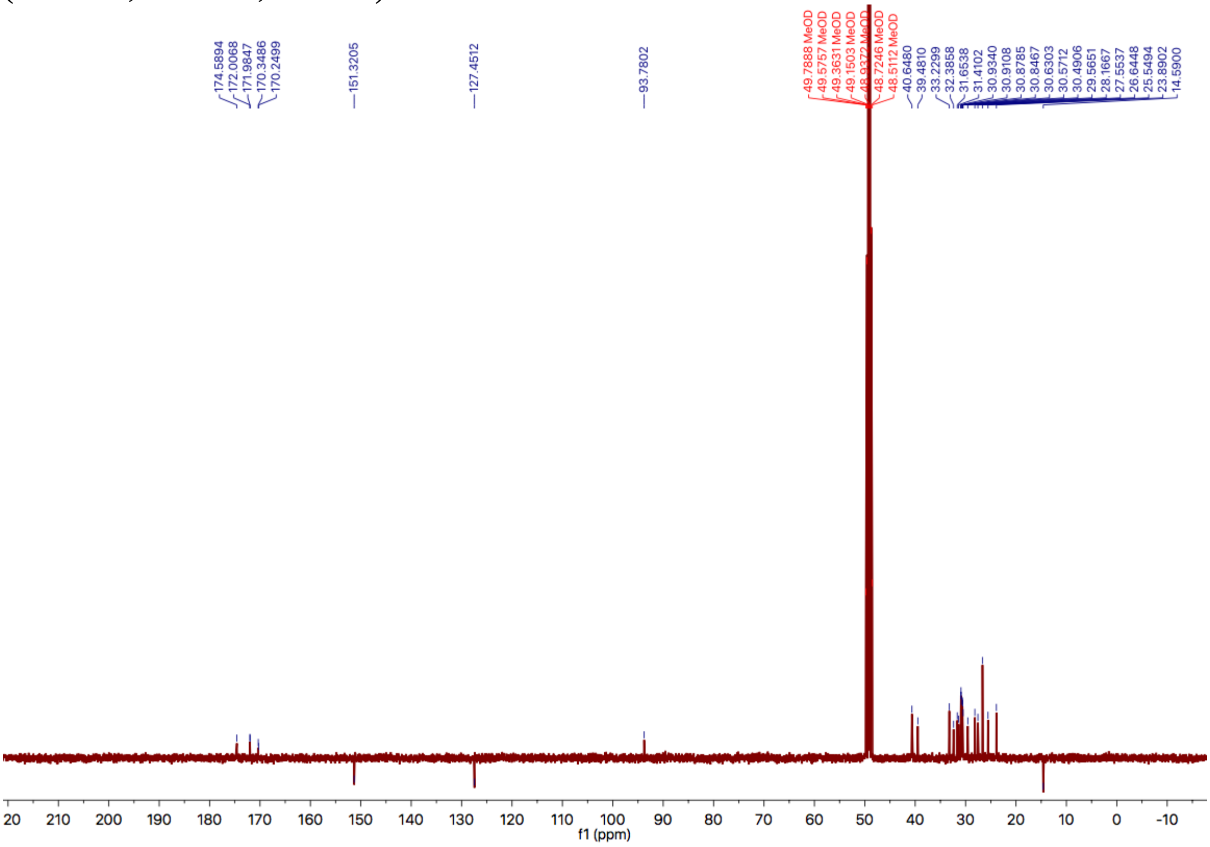

Compound 20

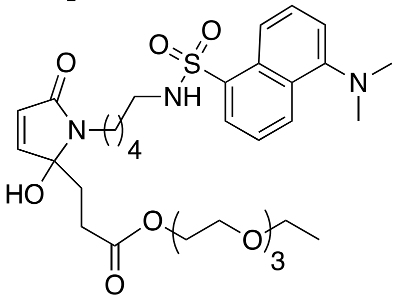

(400 MHz, CD_3CN)

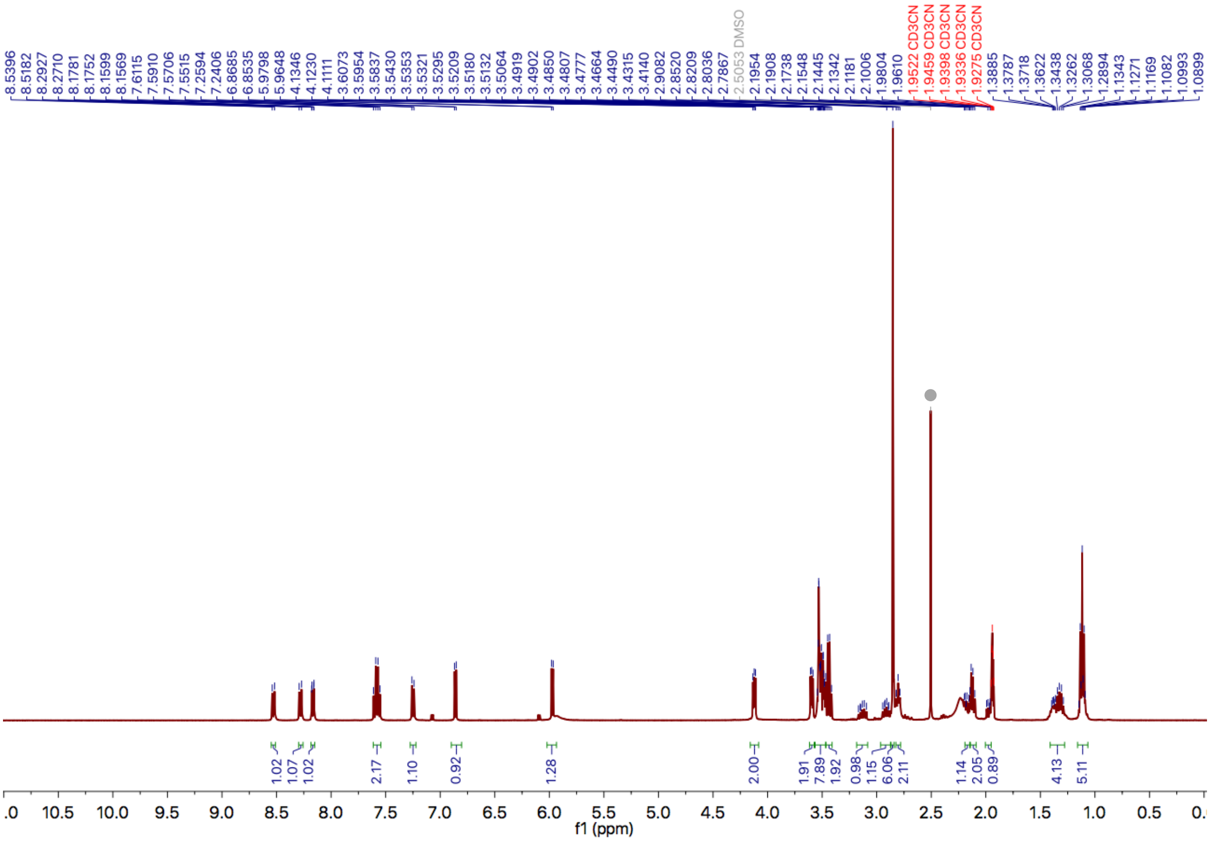

Residual DMSO solvent present from DMSO stock solution from bioconjugation reactions(●)

(^{13}C-APT,101 MHz, CD_3CN)

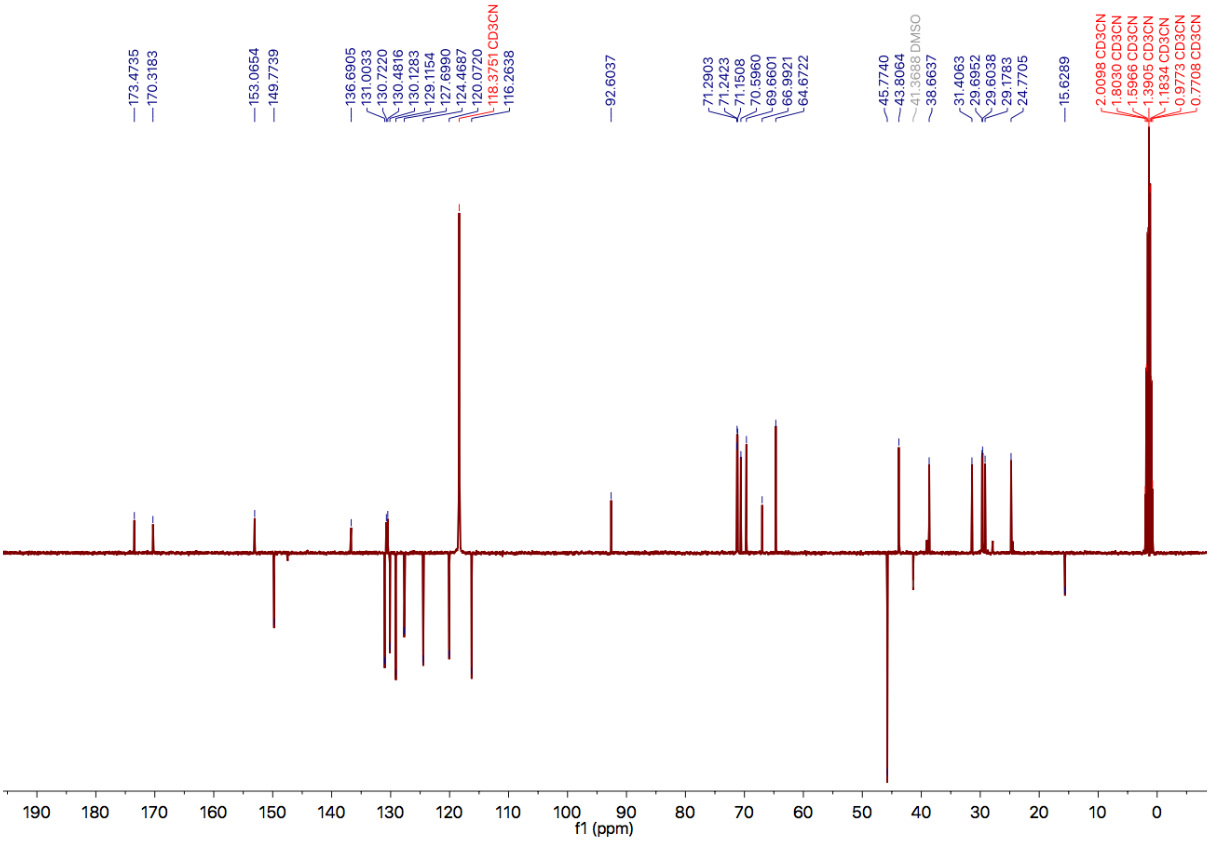

Compound 2-OMe

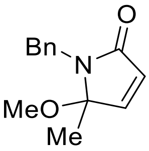

(500 MHz, $CDCl_3$)

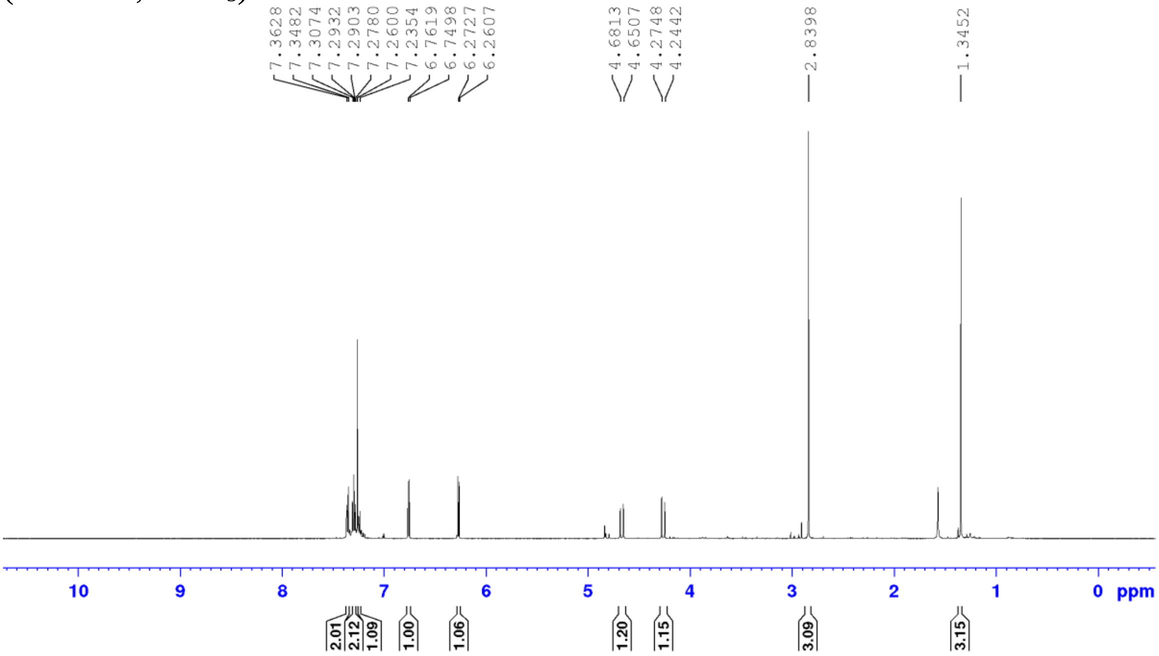

(125 MHz, $CDCl_3$)

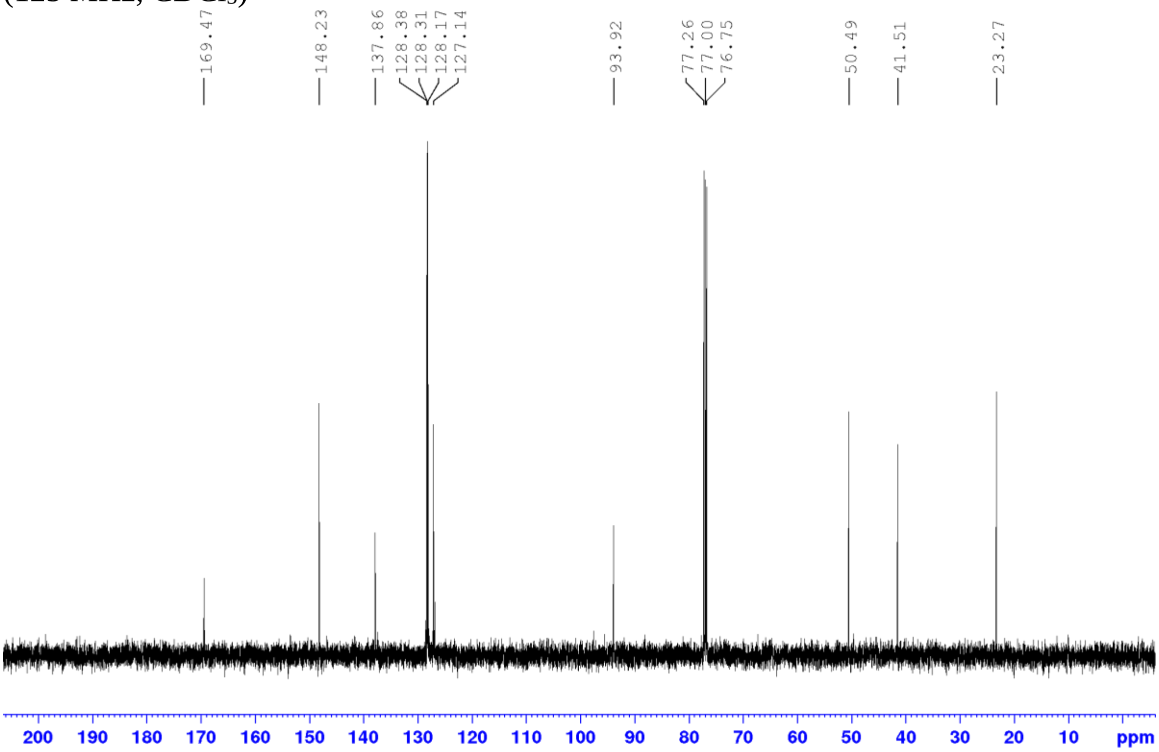

Compound 4 (minor diastereoisomer)

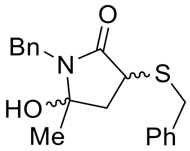

minor
less polar

(500 MHz, $CDCl_3$)

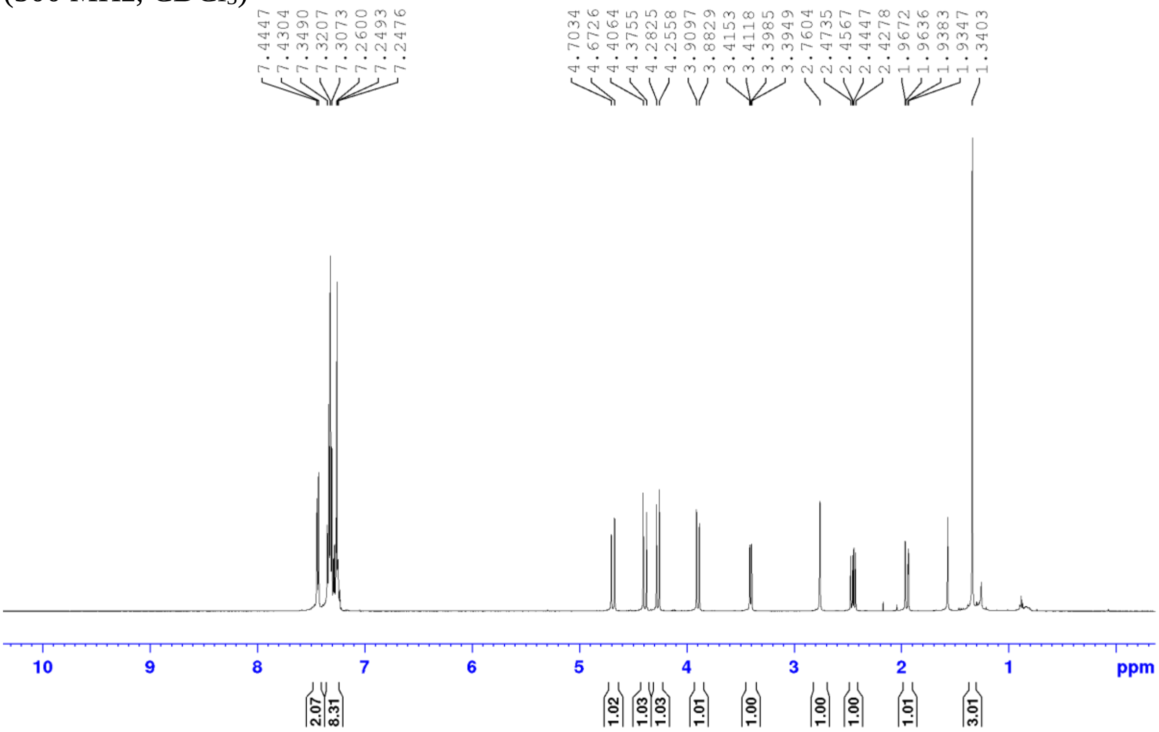

(125 MHz, $CDCl_3$)

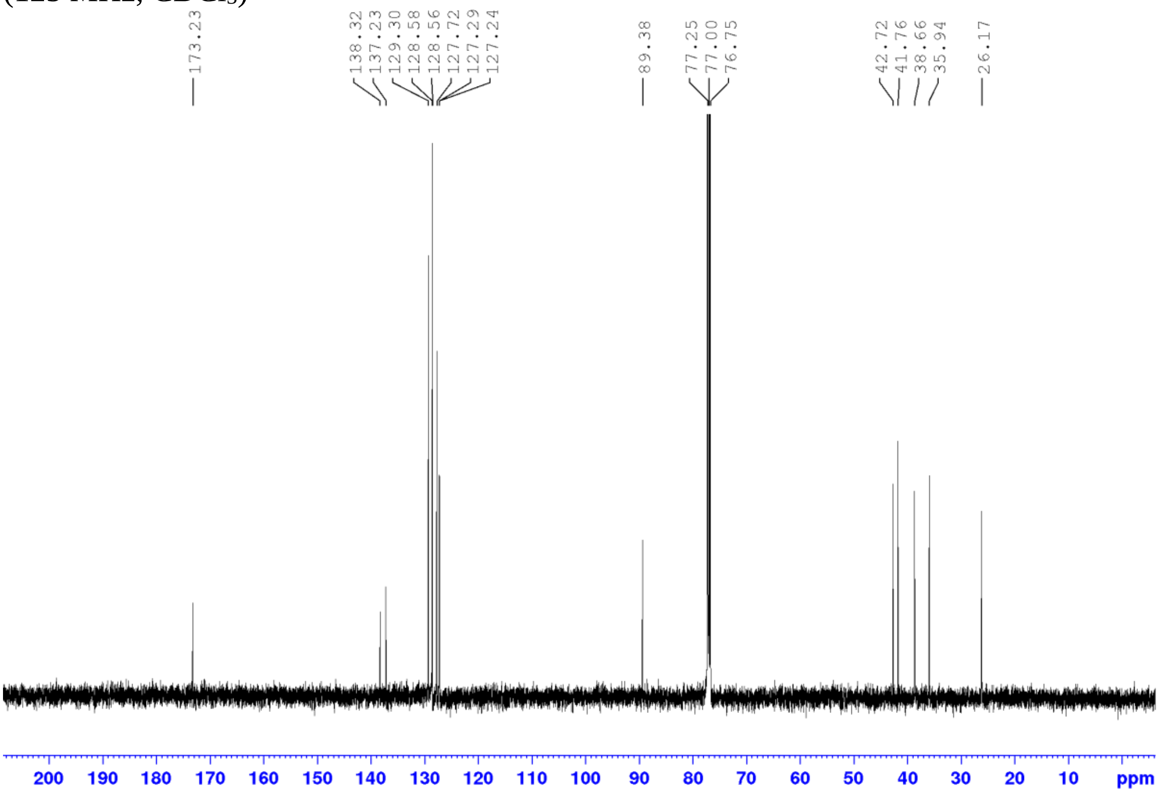

HSQC correlations of compound 4 (minor diastereoisomer)

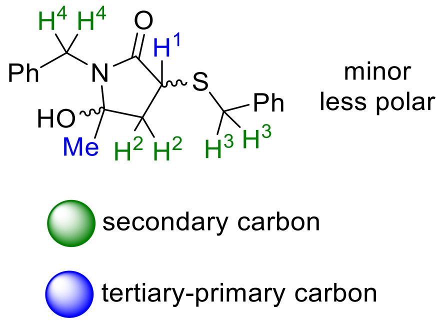

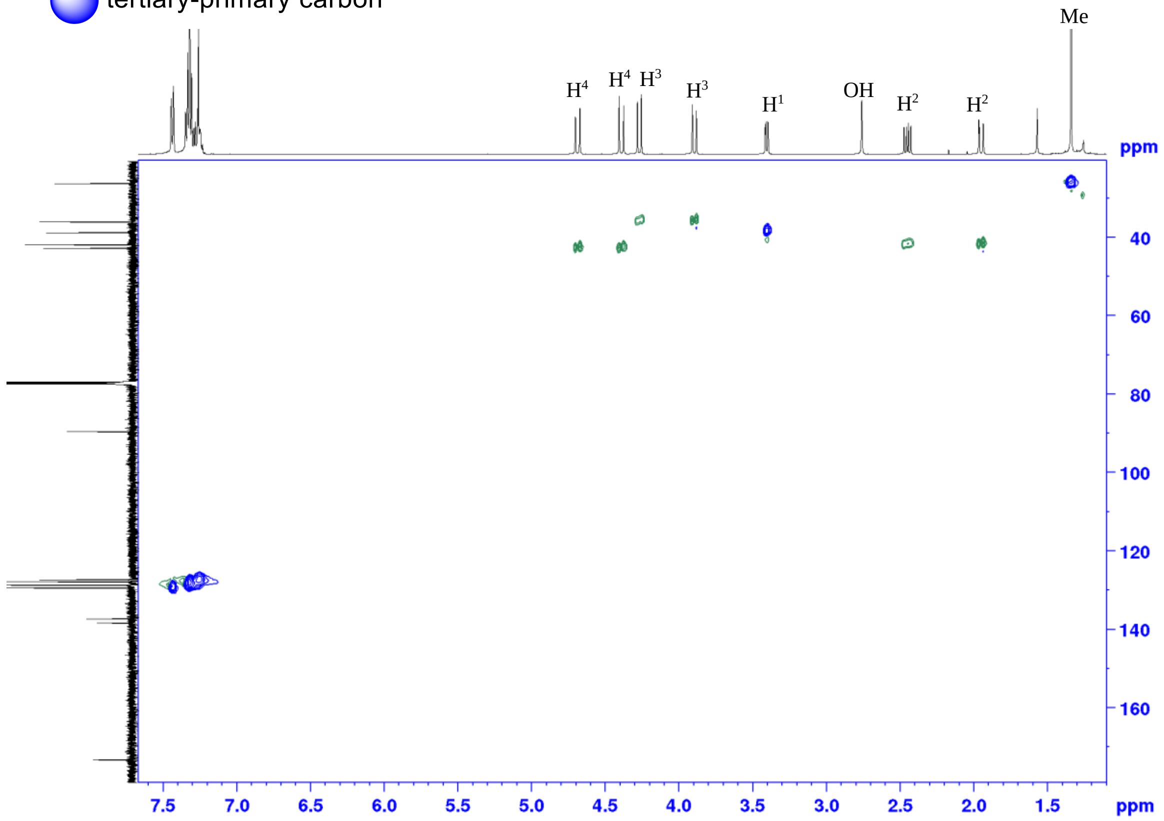

HMBC correlations of compound 4 (minor diastereoisomer)

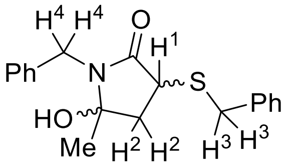

minor
less polar

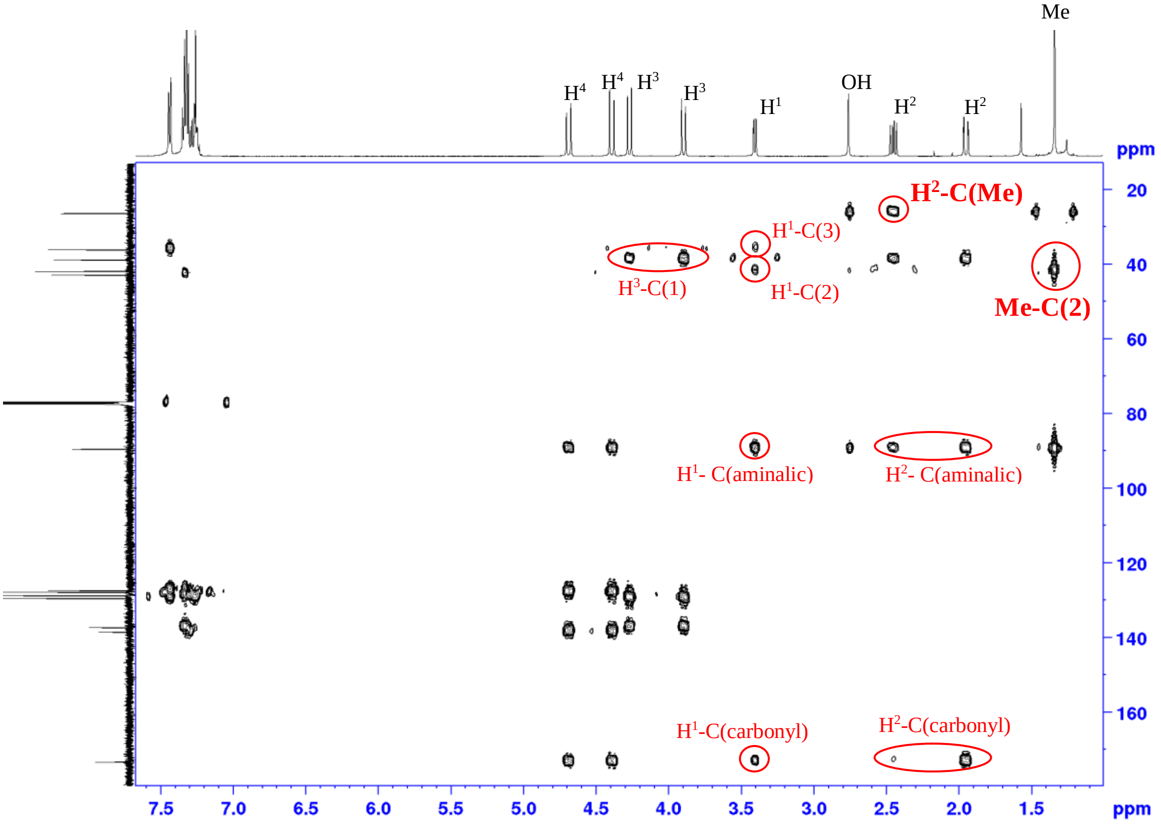

Compound 4 (major diastereoisomer)

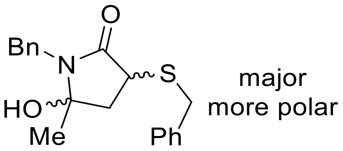

(500 MHz, $CDCl_3$)

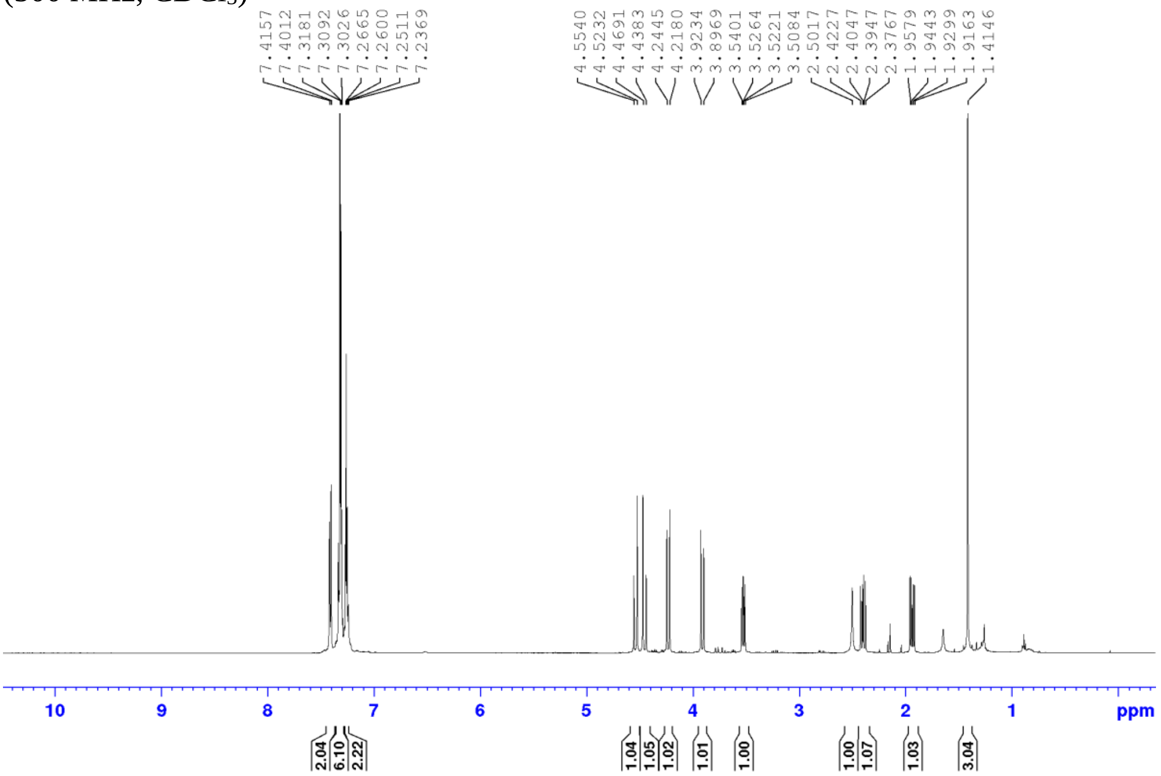

(125 MHz, $CDCl_3$)

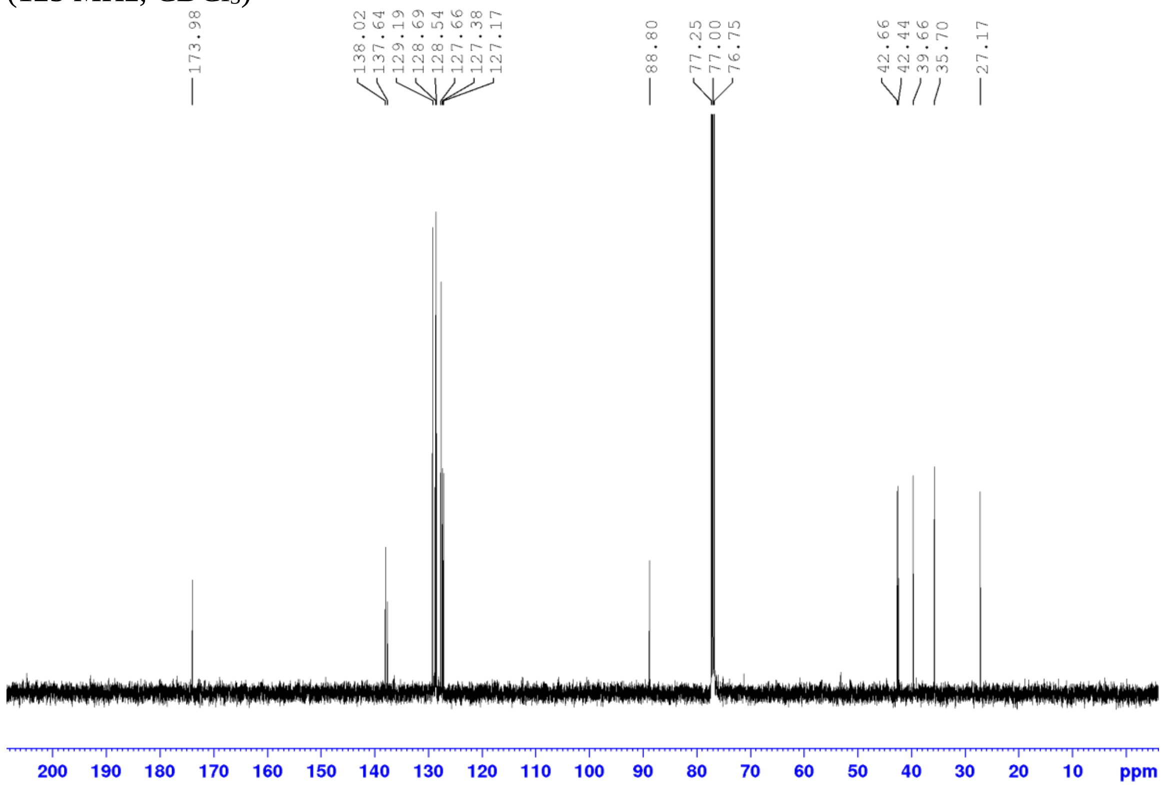

HSQC correlations of compound 4 (major diastereoisomer)

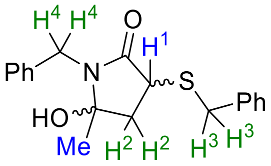

major
more polar

secondary carbon

tertiary-primary carbon

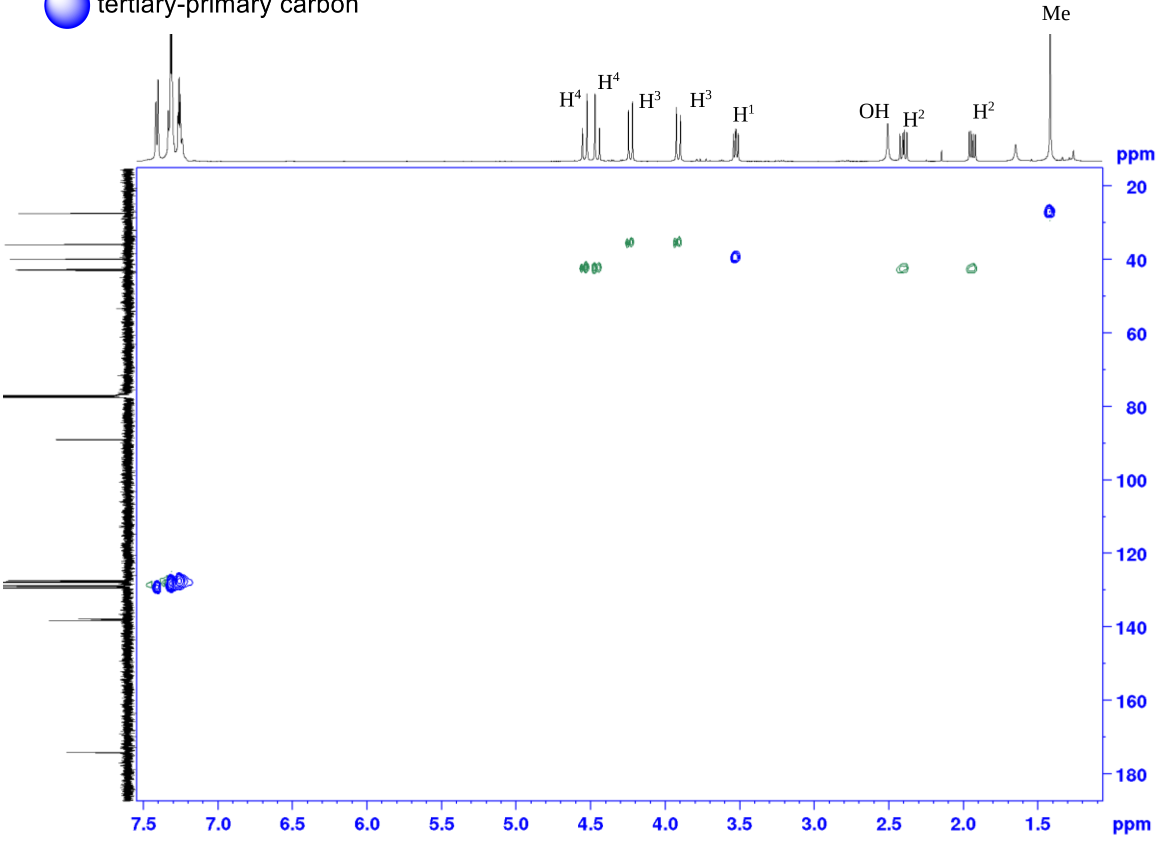

HMBC correlations of compound 4 (major diastereoisomer)

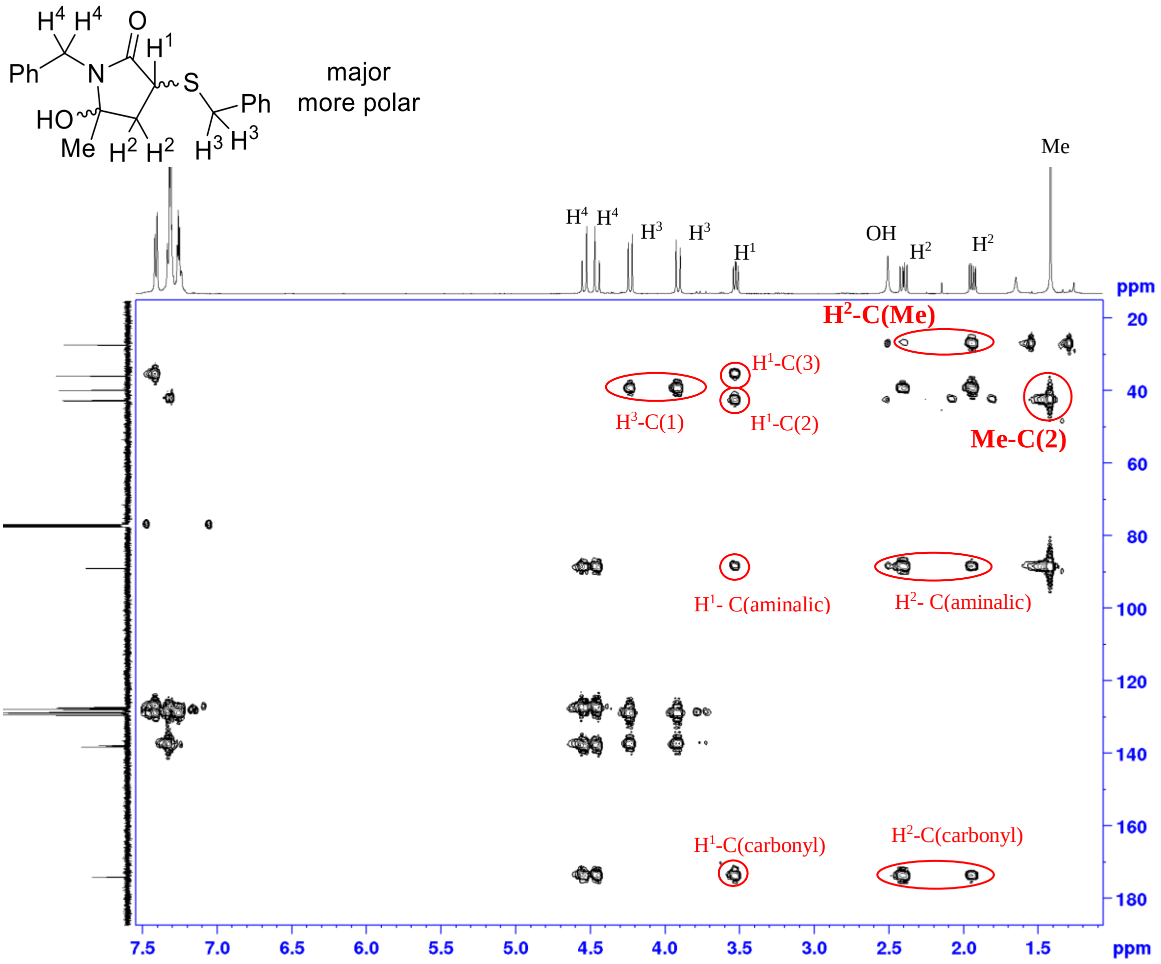

Compound 5 (major diastereoisomer)

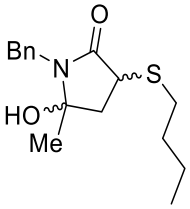

(400 MHz, CD_3CN)

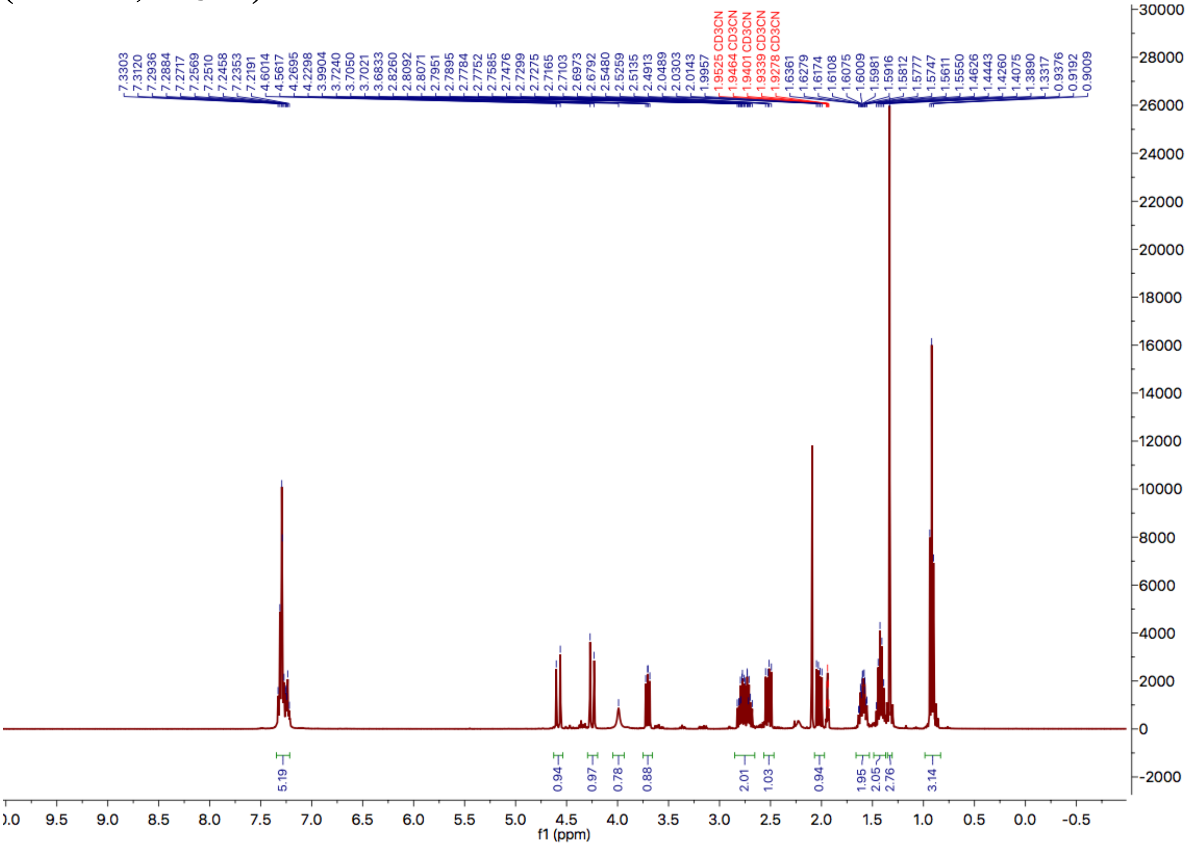

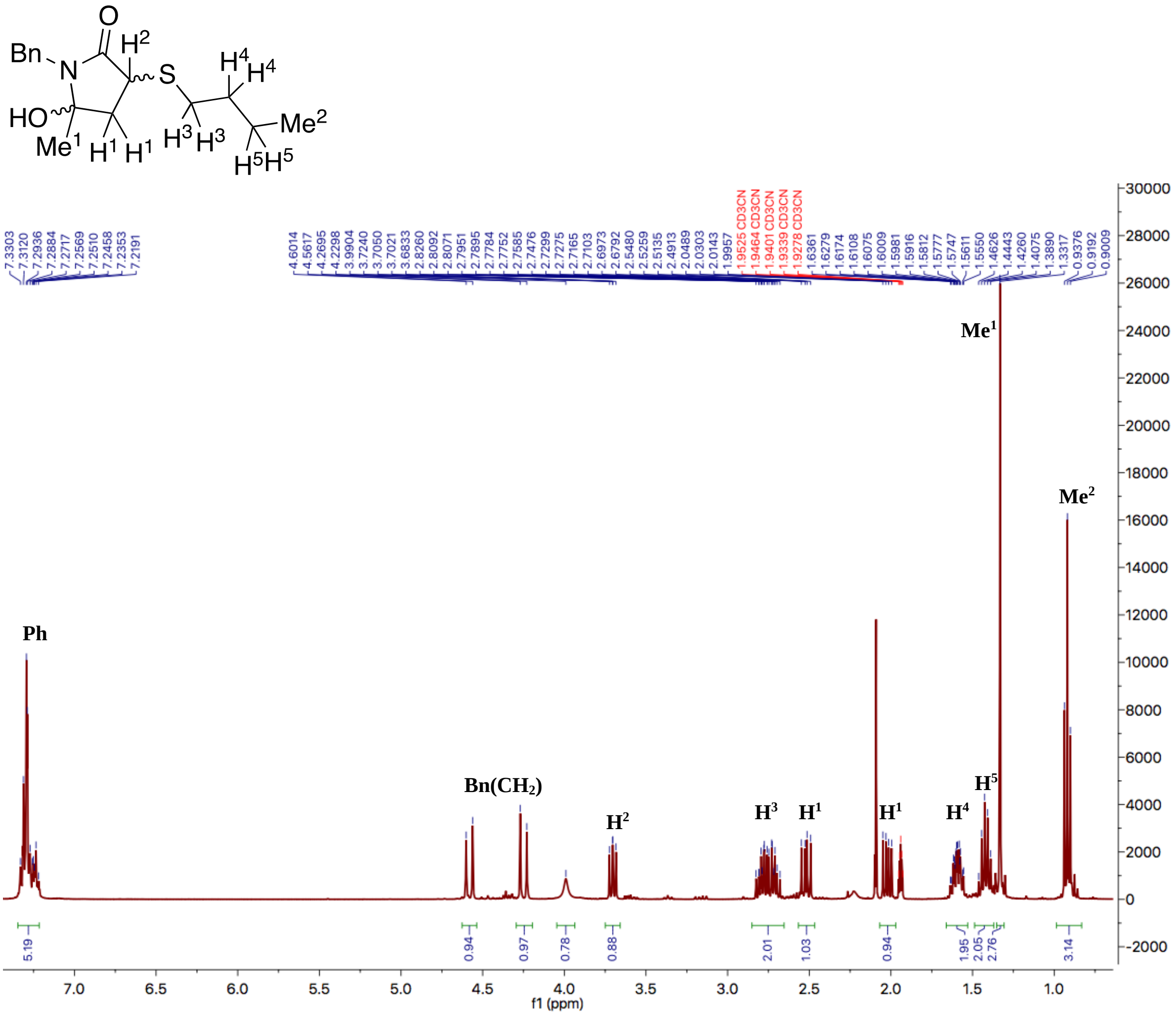

*Zoomed region of 1H-NMR of **5** (400 MHz, CD$_3$CN).*

(^{13}C-APT, 101 MHz, CD_3CN)

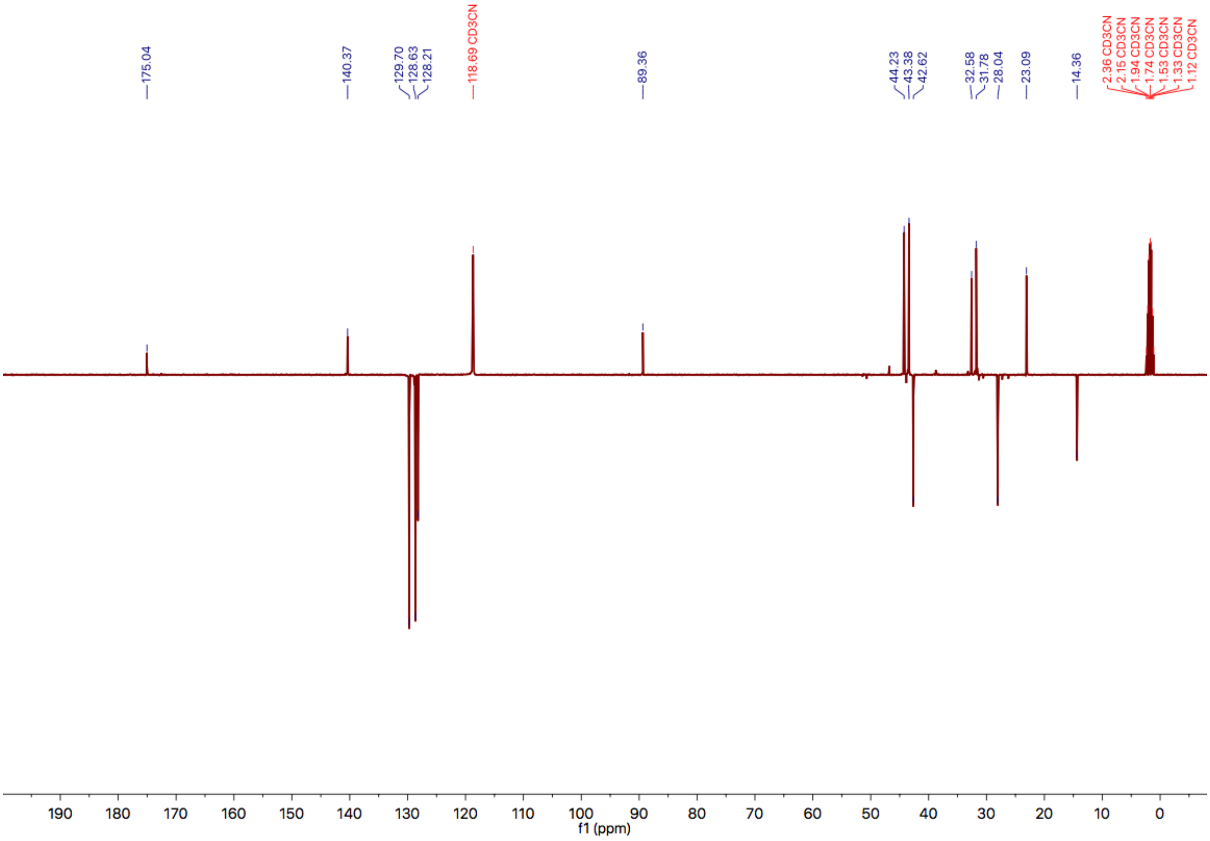

COSY correlations of compound 5 (major diastereoisomer)

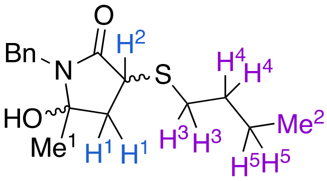

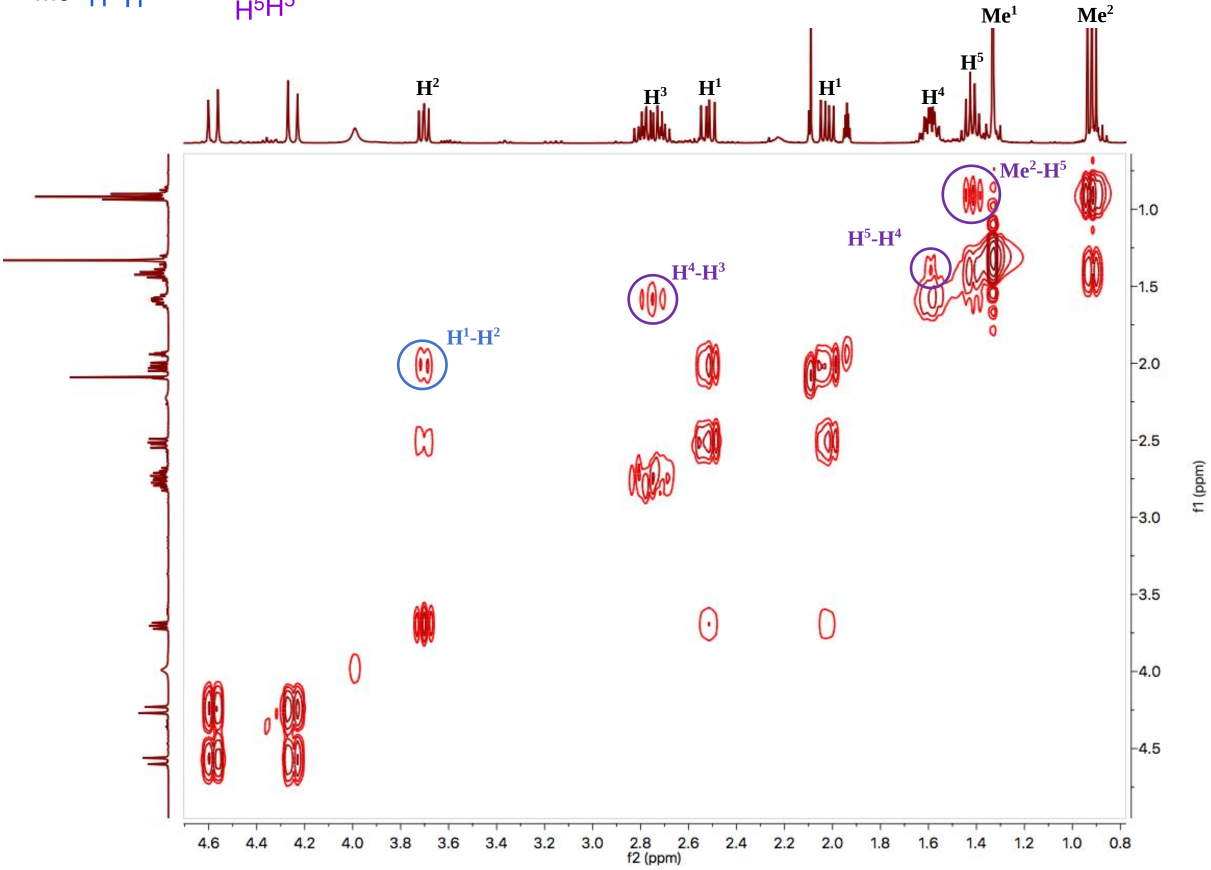

HSQC correlations of compound 5 (major diastereoisomer)

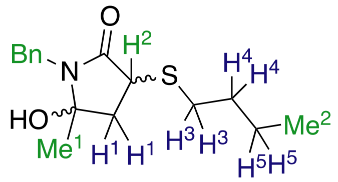

tertiary-primary carbon

secondary carbon

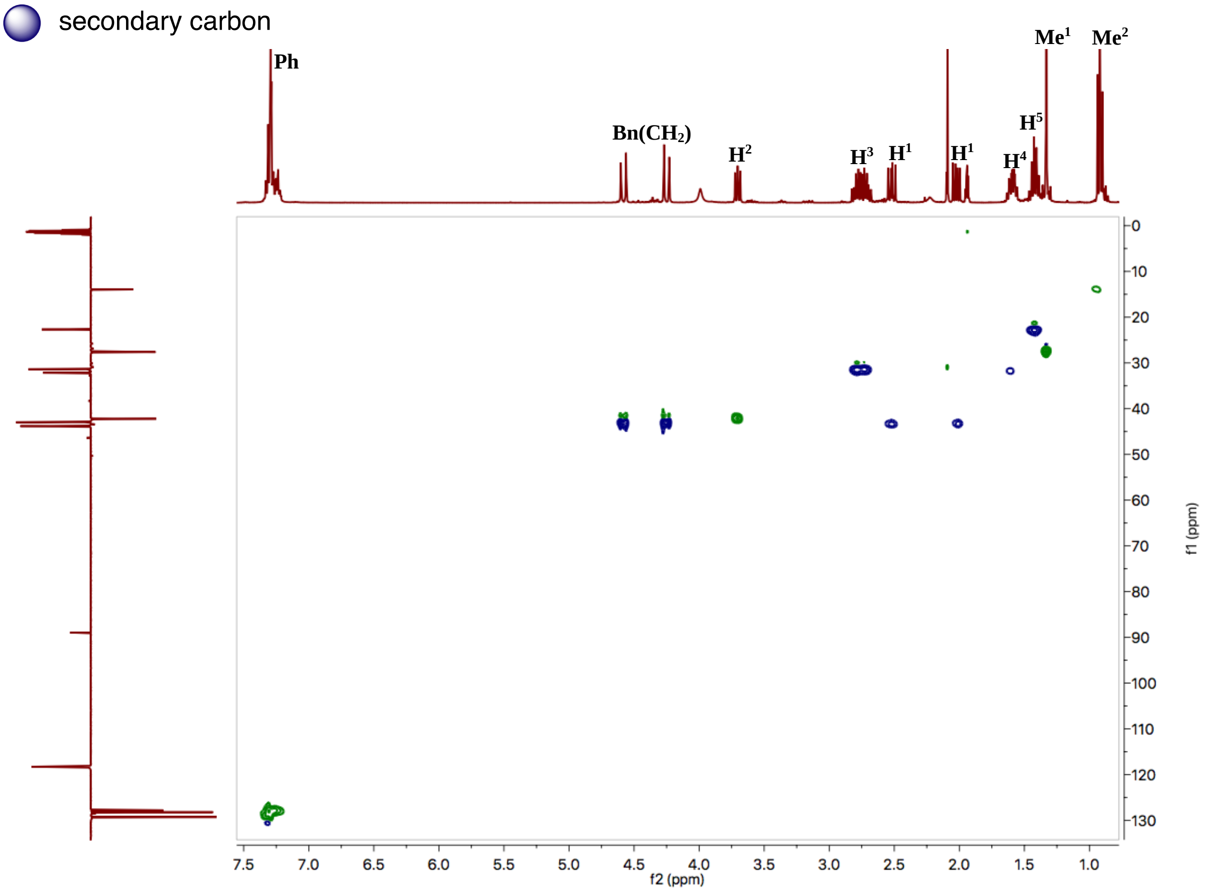

HMBC correlations of compound 5 (major diastereoisomer)

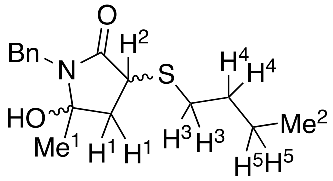

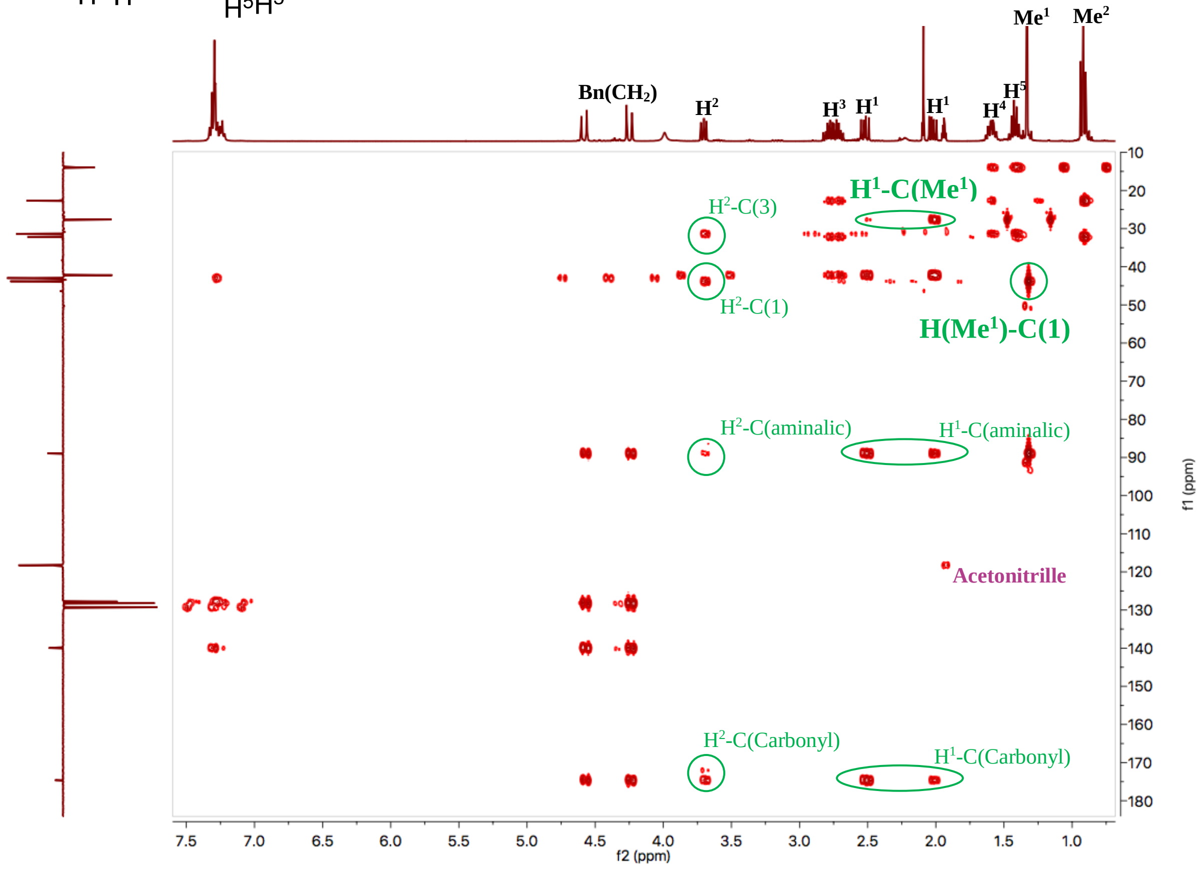

Overlay of HSQC (blue/green) and HMBC (red) NMR analysis of compound 5.

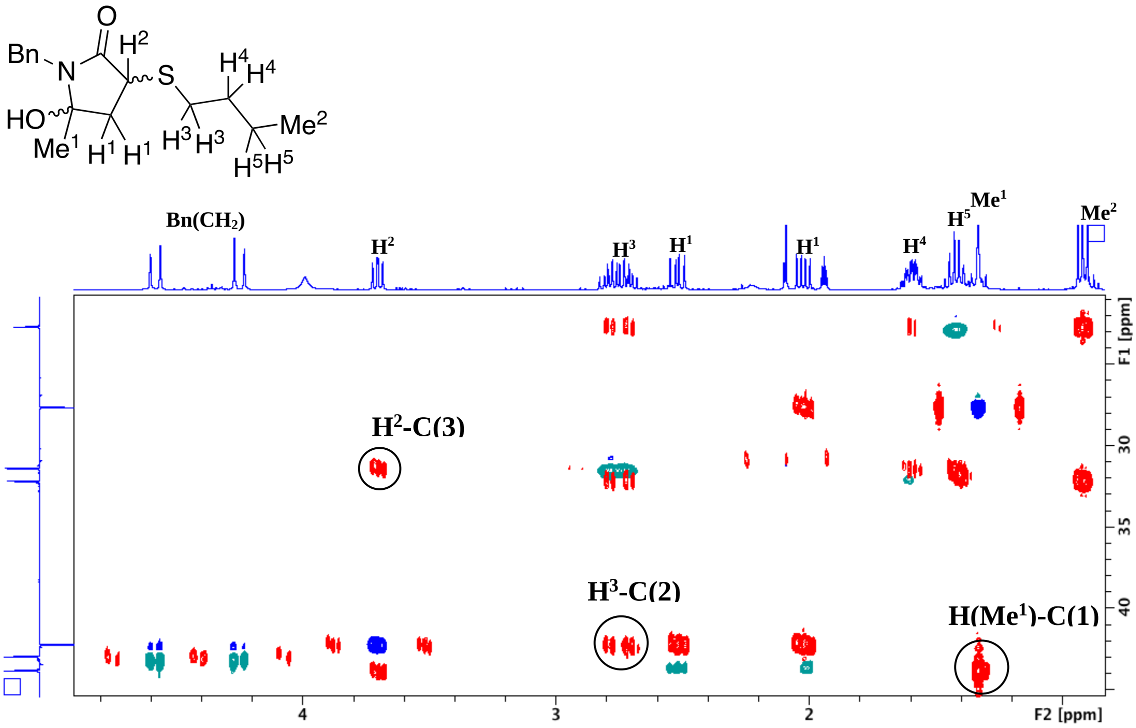

Compound 6 (major diastereoisomer)

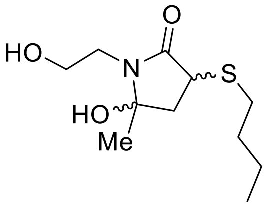

(400 MHz, DMSO-d_6)

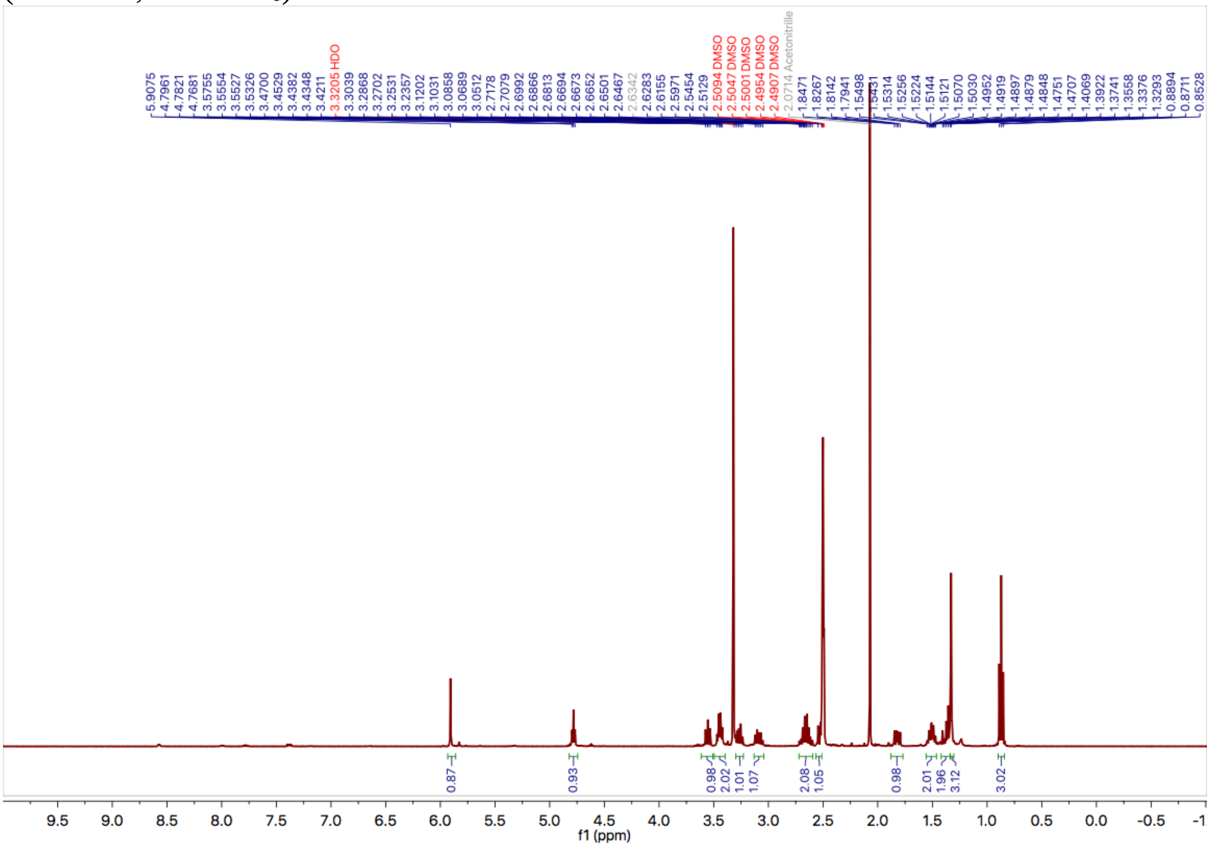

(^{13}C-APT, 101 MHz, DMSO-d_6)

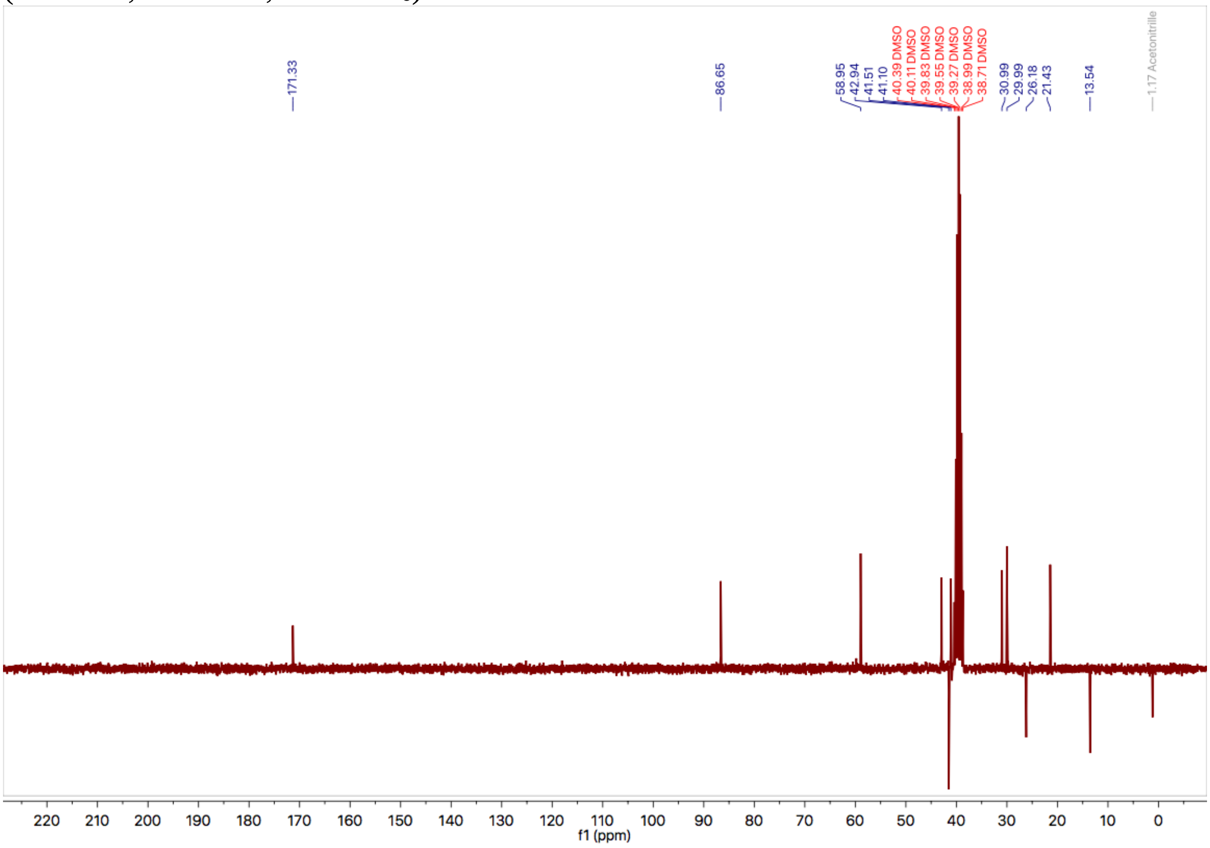

Compound 21 (minor diastereoisomer)

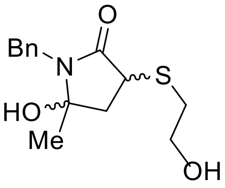

minor
less polar

(500 MHz, $CDCl_3$)

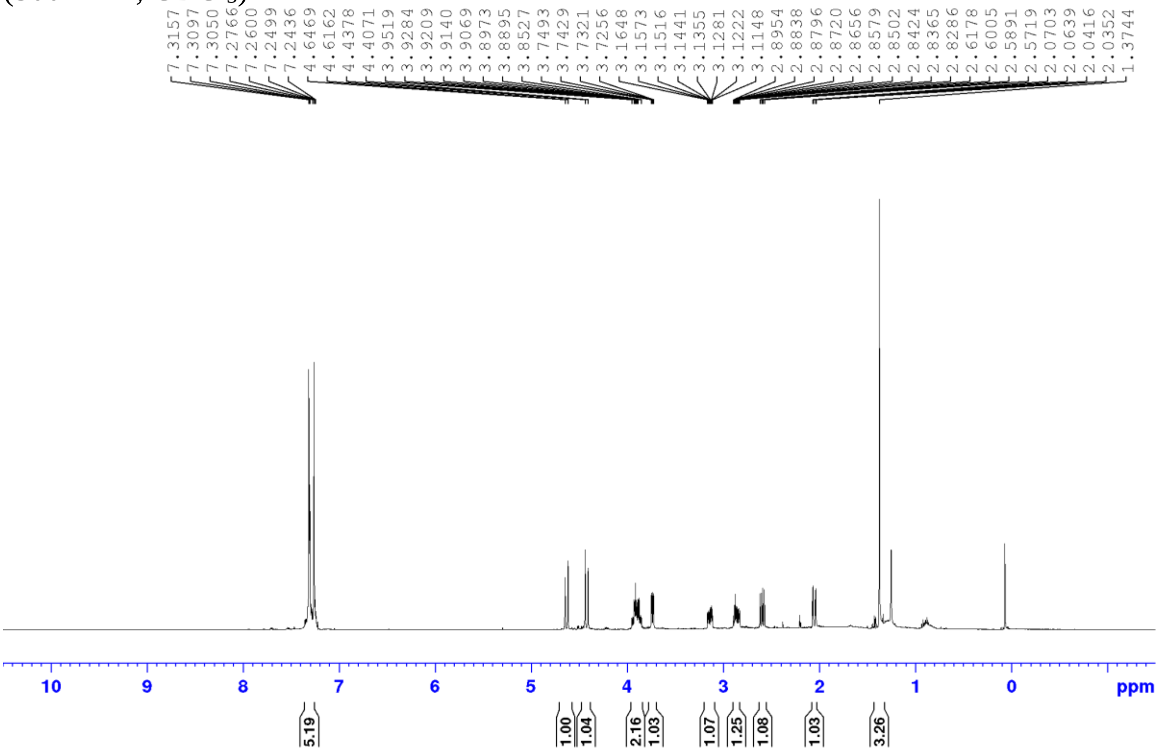

(125 MHz, $CDCl_3$)

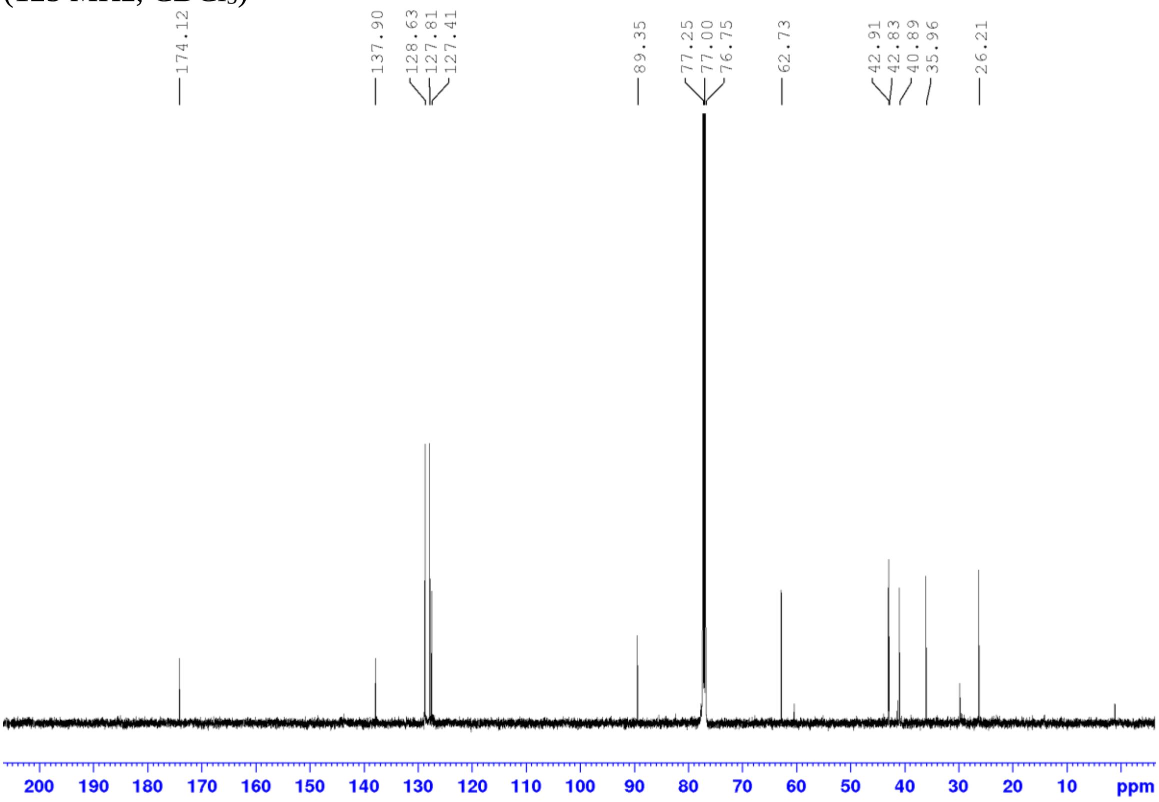

Compound 21 (major diastereoisomer)

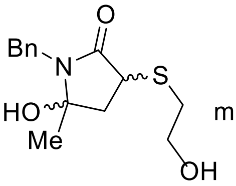

major
more polar

(500 MHz, $CDCl_3$)

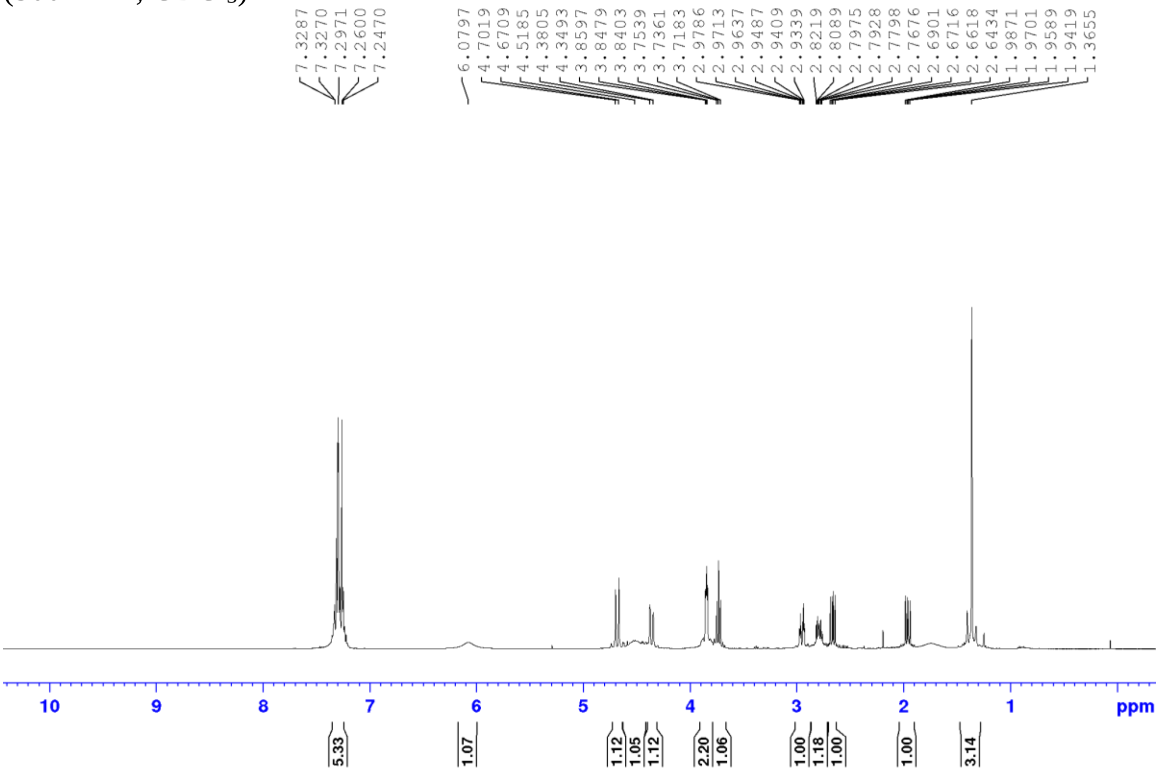

(125 MHz, $CDCl_3$)

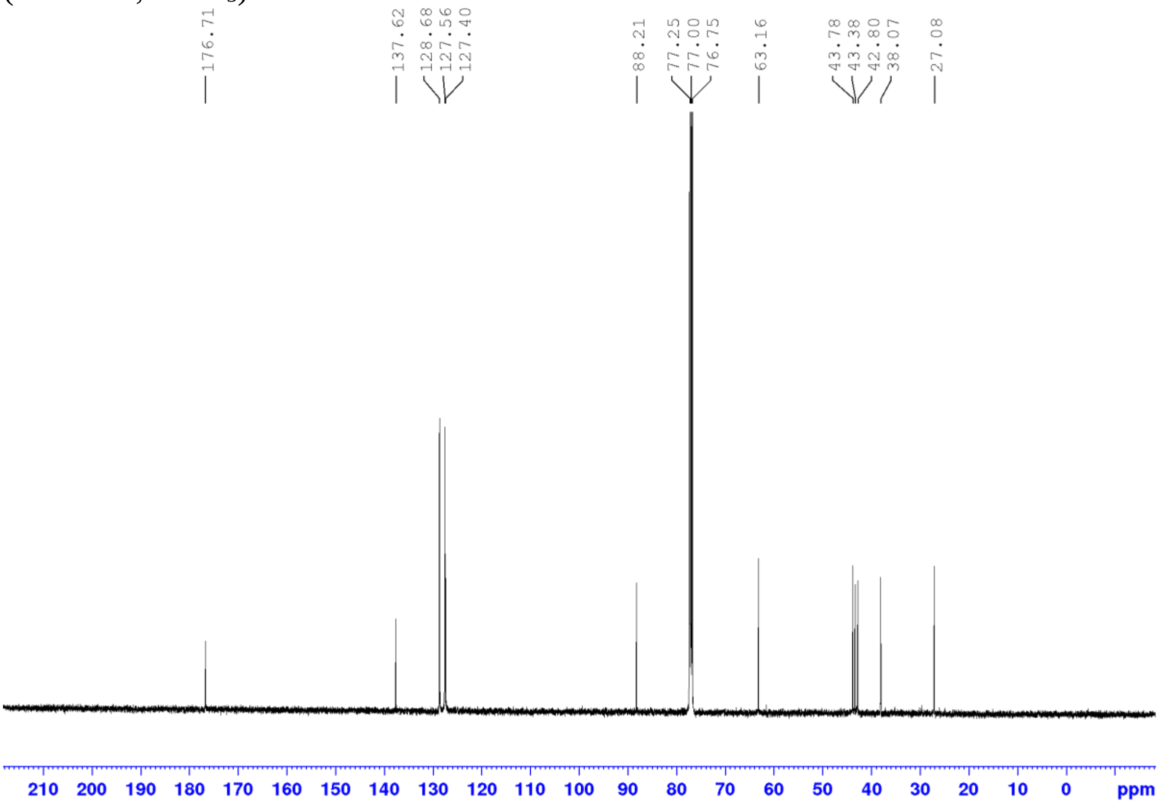

Compound 22

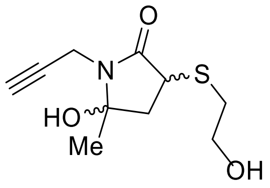

(500 MHz, $CDCl_3$)

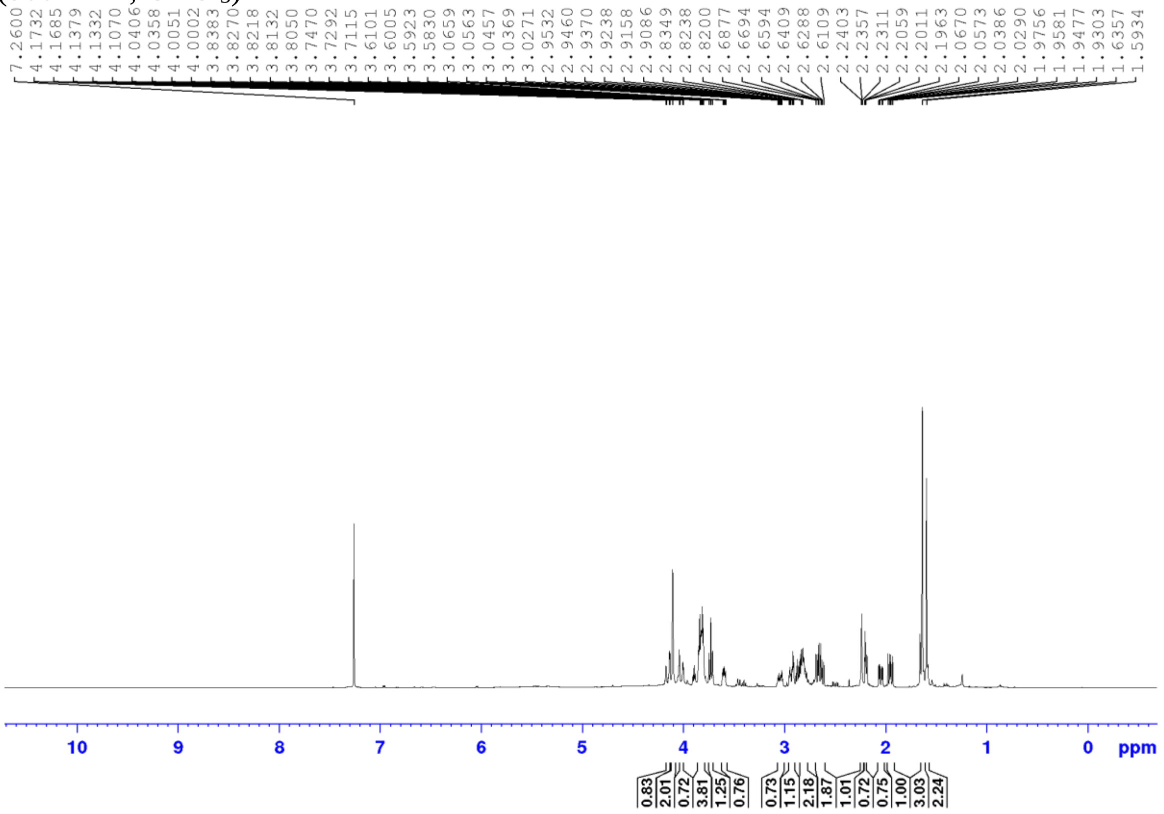

(125 MHz, $CDCl_3$)

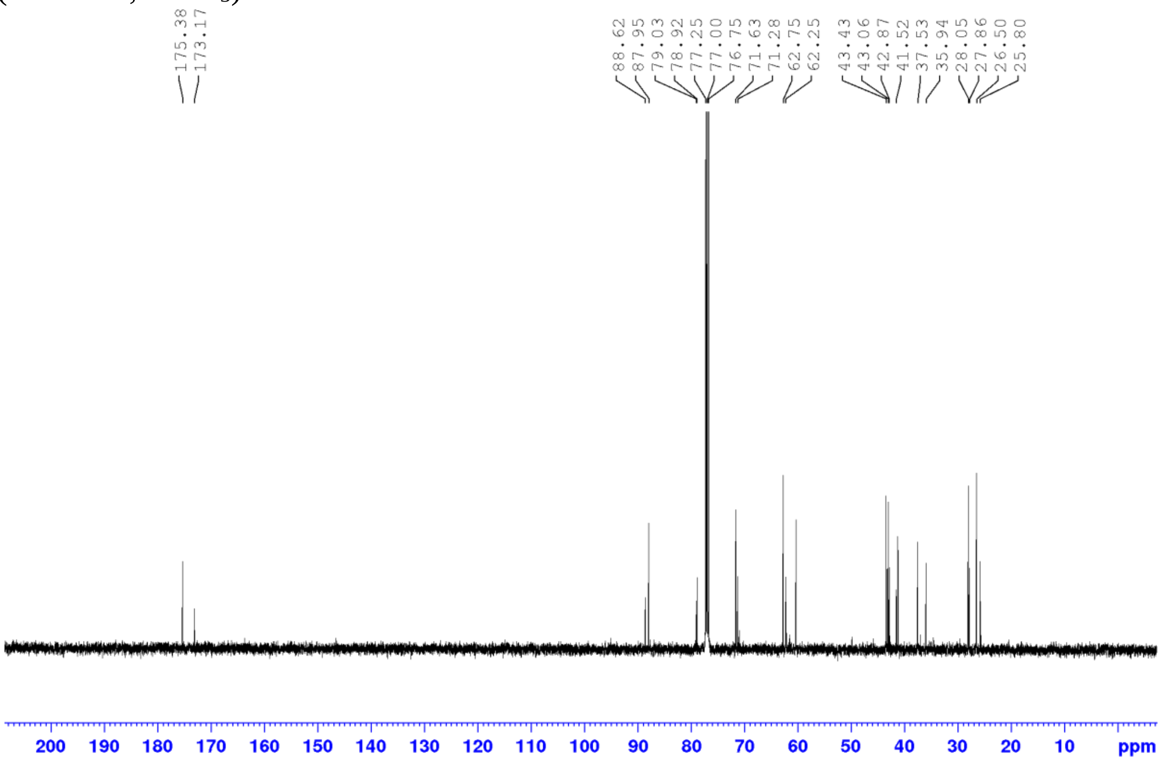

Compound 23

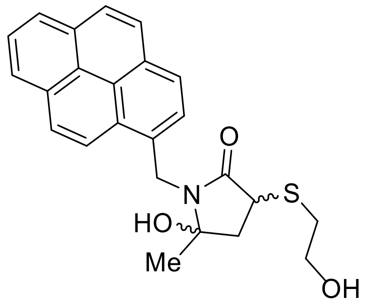

(500 MHz, $CDCl_3$)

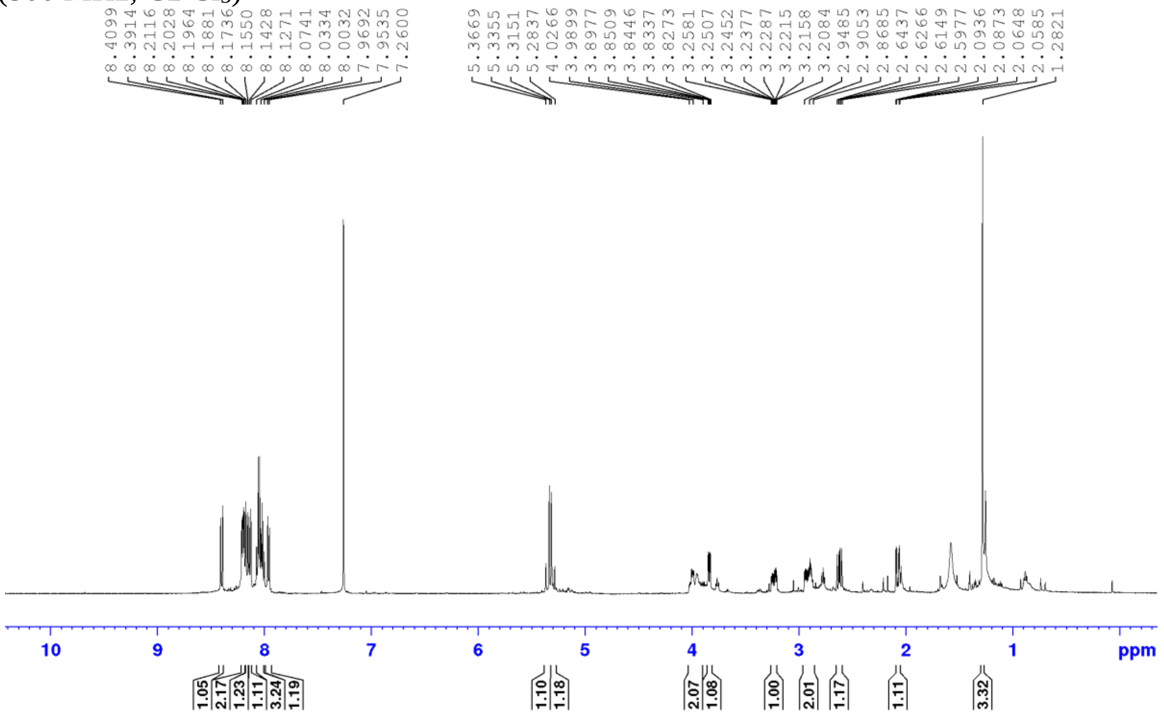

(125 MHz, $CDCl_3$)

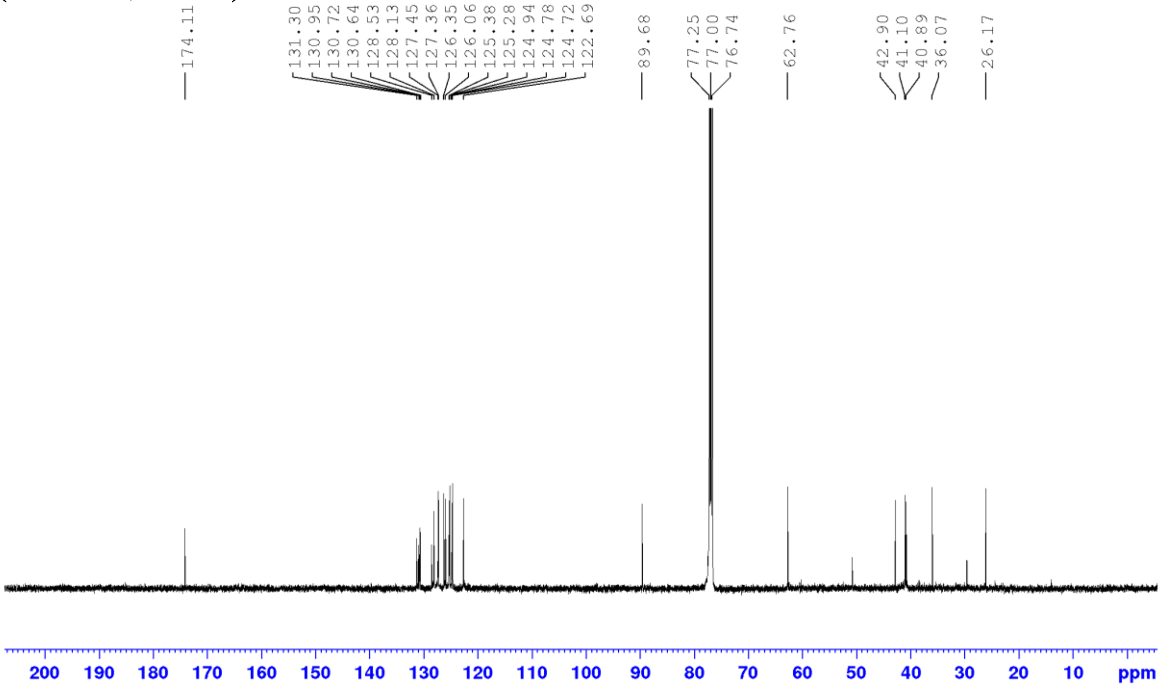

Compound 24

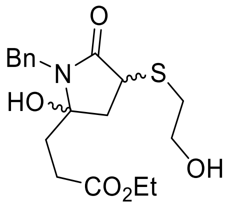

(500 MHz, $CDCl_3$)

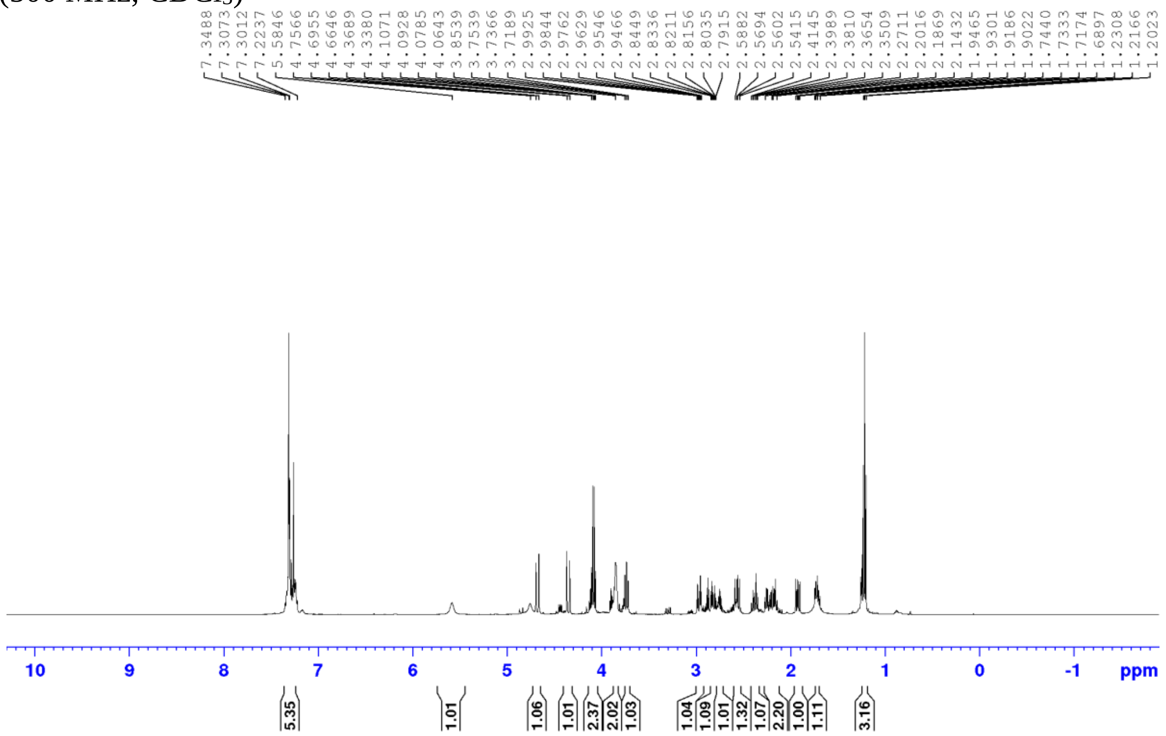

(125 MHz, $CDCl_3$)

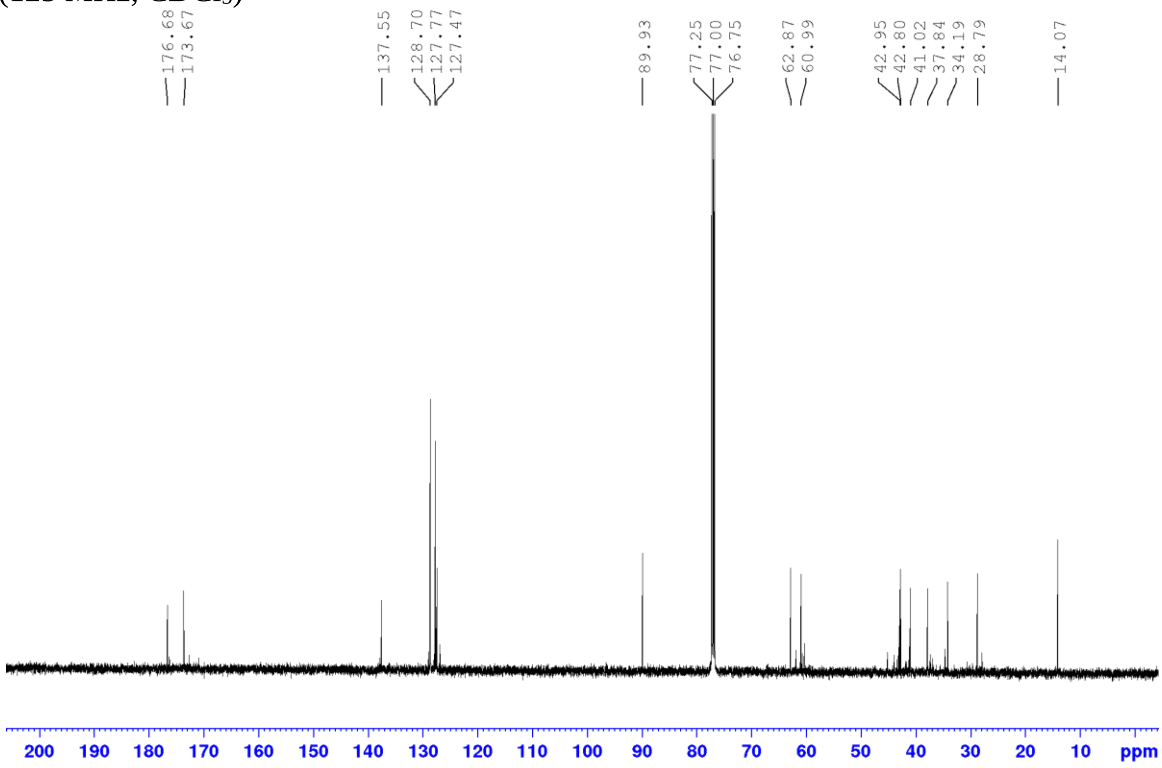

Compound 25

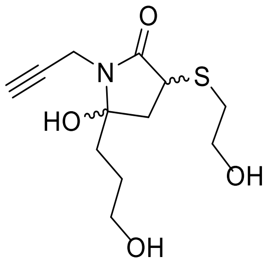

(500 MHz, CD_3OD)

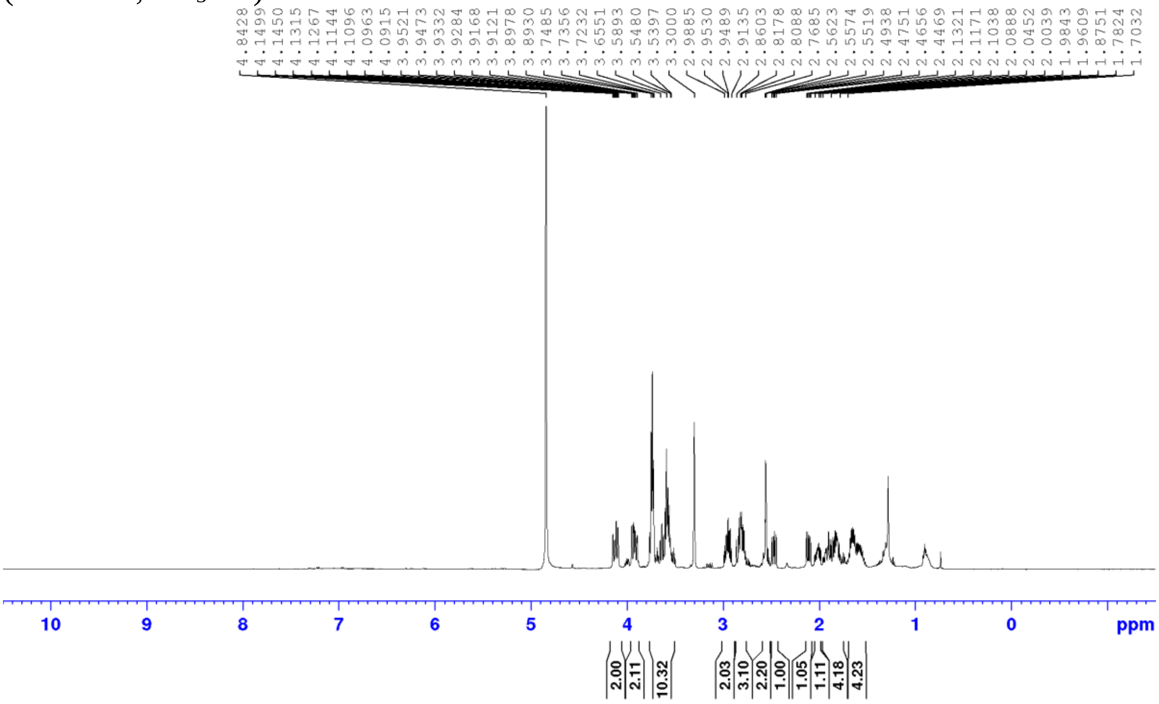

(125 MHz, CD_3OD)

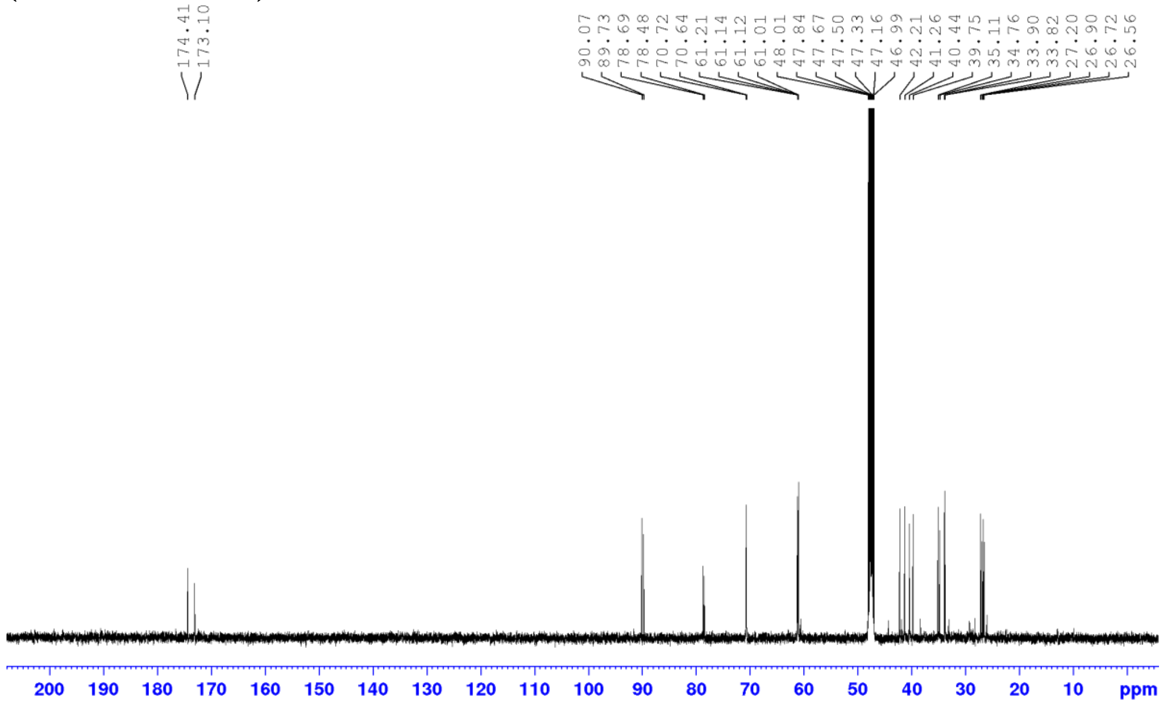

Compound 26 (more polar)

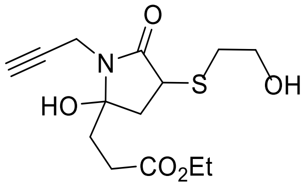

(300 MHz, $CDCl_3$)

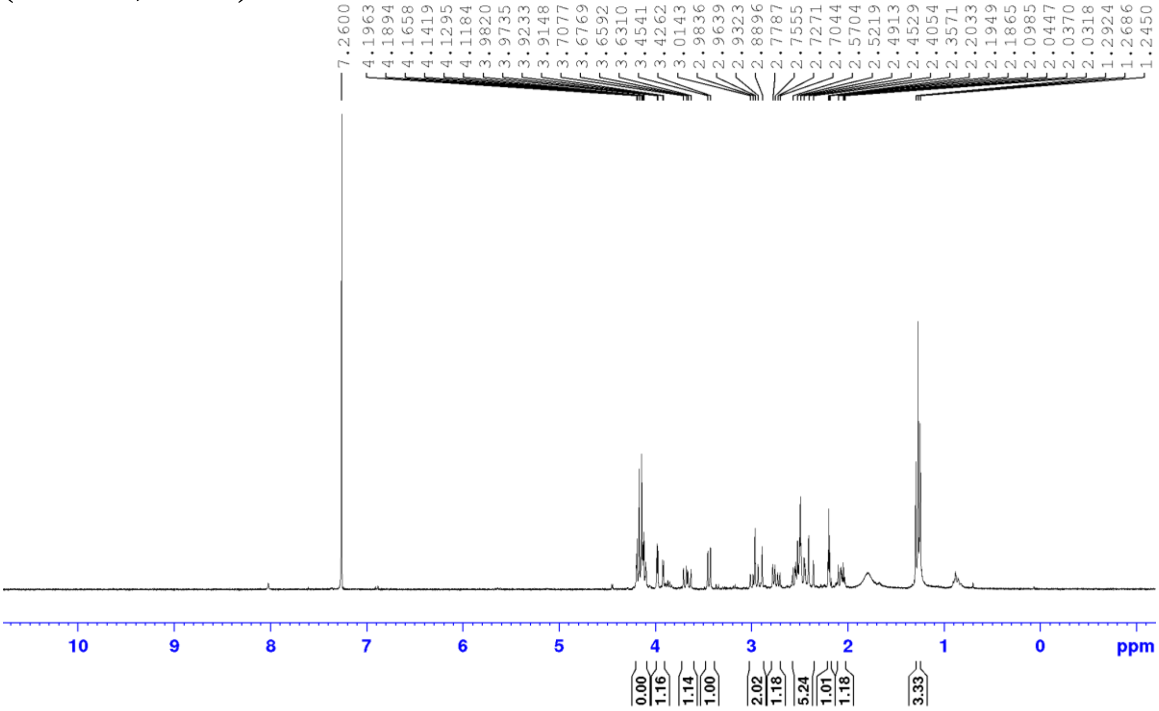

(75 MHz, $CDCl_3$)

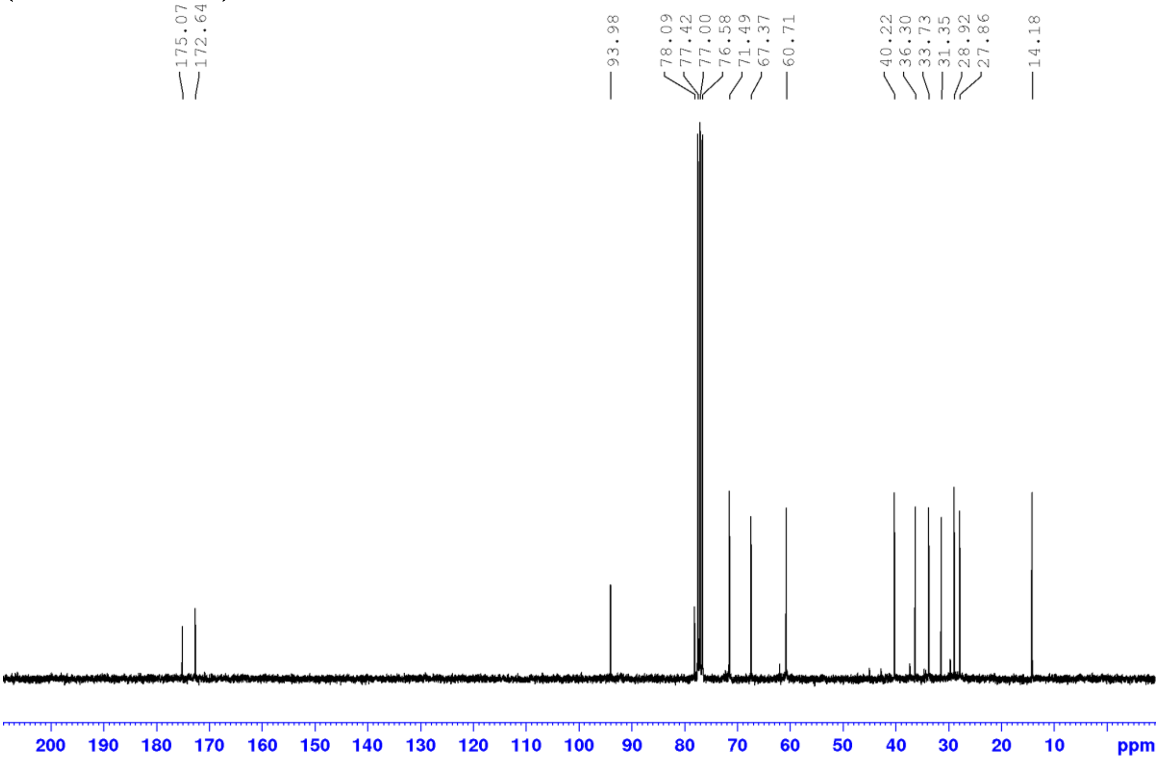
175.07
172.64
93.98
78.09
77.42
77.00
76.58
71.49
67.37
60.71
40.22
36.30
33.73
31.35
28.92
27.86
14.18
200 190 180 170 160 150 140 130 120 110 100 90 80 70 60 50 40 30 20 10 ppm

Compound 28

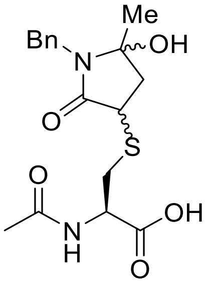

(400 MHz, DMSO-d_6)

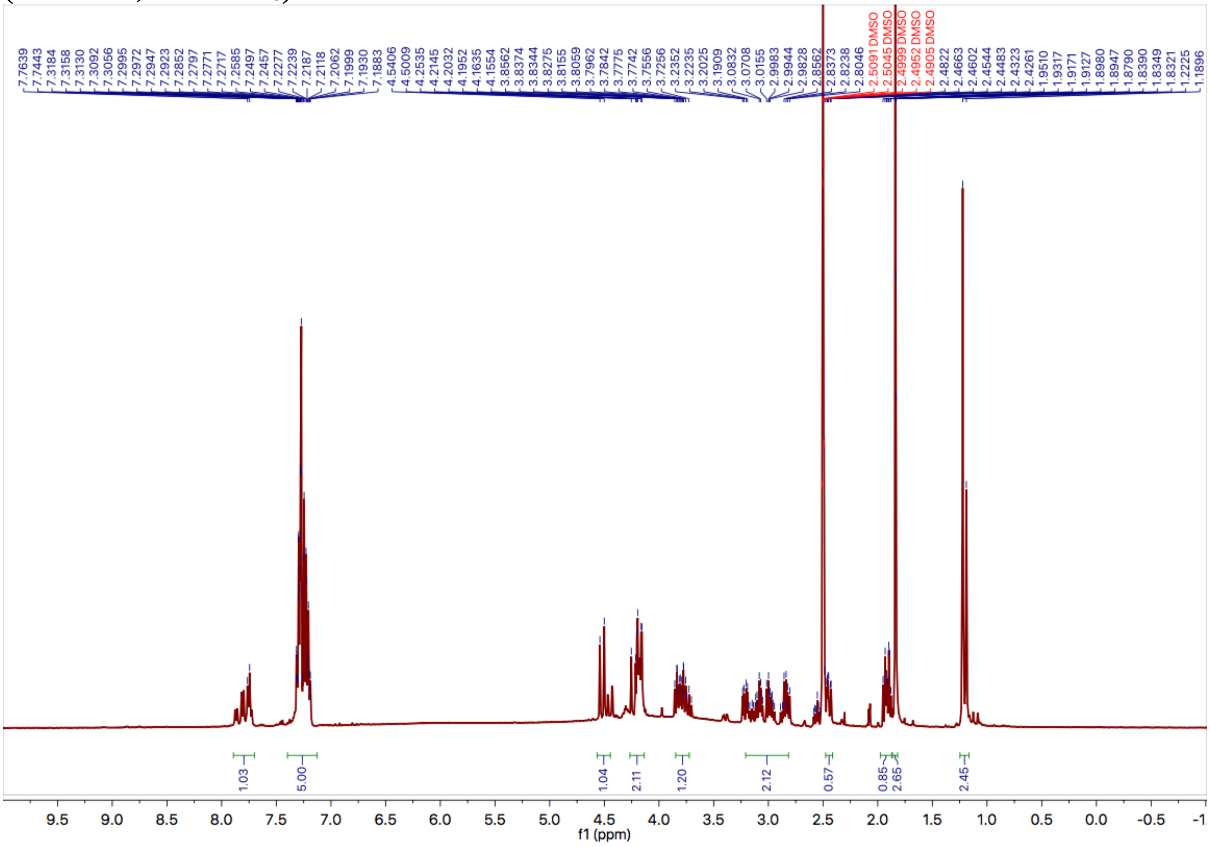

(^{13}C-APT, 101 MHz, DMSO-d$_6$)

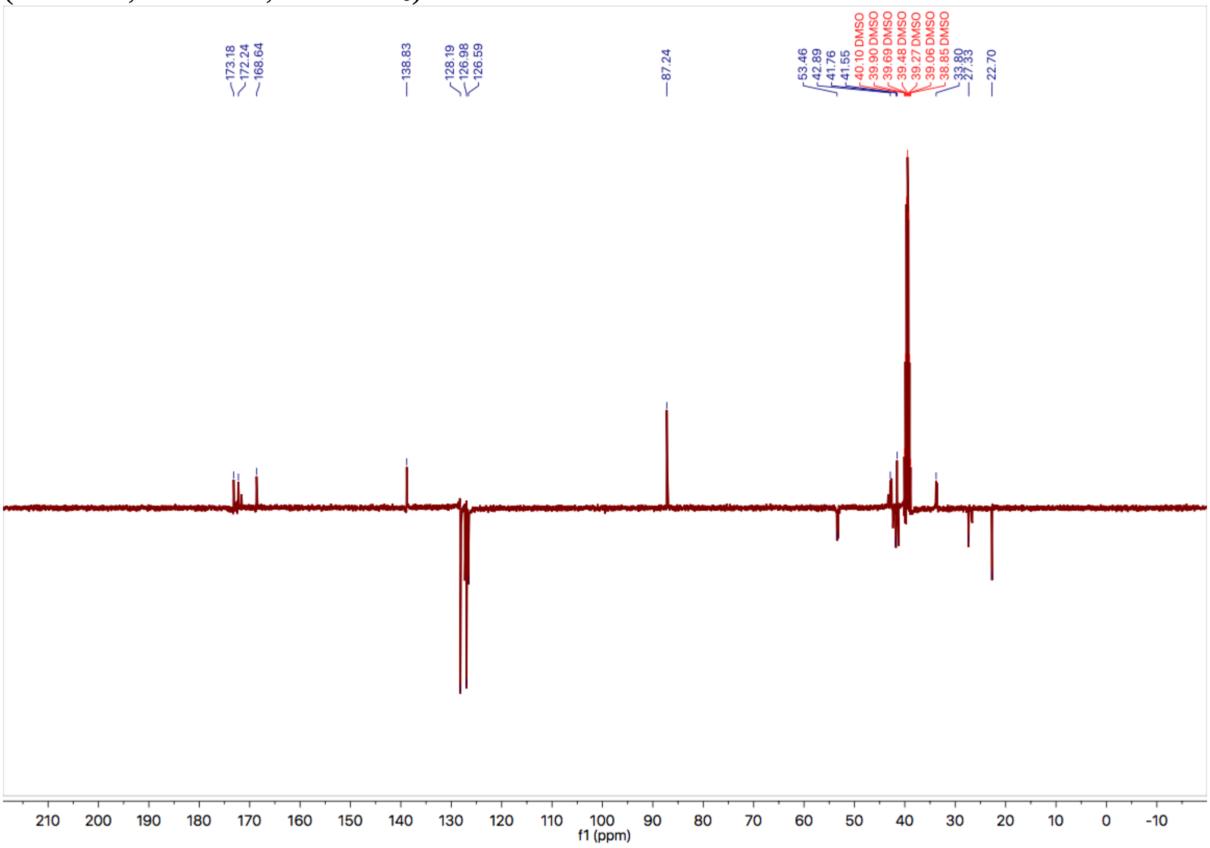

Supplement: SC-012-D0SC05881E-s001 [file SC-012-D0SC05881E-s001.pdf]
